# Efficacy and safety of anticoagulants on venous thromboembolism: a systematic review and network meta-analysis of randomized controlled trials

**DOI:** 10.3389/fphar.2024.1519869

**Published:** 2025-01-08

**Authors:** Weijie Fu, Maolin Zhao, Sheng Ding, Mei Xin, Ke Yang, Li Jiang, Fan Wu, Xiaochen Wu, Jian Wang, Jie Chen, Feng Gao, Siyi He

**Affiliations:** Department of Cardiovascular Surgery, Affiliated Hospital of Southwest Jiaotong University, The General Hospital of Western Theater Command, Chengdu, China

**Keywords:** anticoagulants, meta-analysis, systematic review, venous thromboembolism, novel oral anticoagulants

## Abstract

**Background:**

Anticoagulants are the primary means for the treatment and prevention of venous thromboembolism (VTE), but their clinical standardized application still remains controversial. The present study intends to comprehensively compare the efficacy and safety of various anticoagulants in VTE.

**Methods:**

Medline, Embase, and Cochrane Library from their inception up to August 2023 were searched to compare the efficacy and safety of various anticoagulants in VTE. We extracted data on study settings, baseline characteristics, interventions, and outcomes, applying the intention-to-treat principle. Two researchers assessed study bias using the Cochrane tool, resolving disagreements through discussion or third-party adjudication. Network meta-analyses were performed based on Bayesian generalized linear models, and a frequentist framework with multivariate random effects was used to fit the model.

**Results:**

In terms of treatment, 58 trials with 119,417 patients proved eligible, while 125 trials with 225,414 patients were included in terms of prevention. All anticoagulants were found to reduce the recurrence or incidence of VTE compared with Placebo, of which high-level evidence indicated that direct thrombin inhibitors (TIs) and novel oral anticoagulants (NOACs) were the two most effective drugs. For treatment, low molecular weight heparin (LMWH), unfractionated heparin (UFH), and vitamin K antagonists (VKAs) significantly increased the risk of major bleeding in comparison to Placebo. For prevention, only UFH (OR 2.0, 95% CI 1.2–3.3) and NOACs (OR 1.8, 95% CI 1.2–2.6) showed significant increased risks in major bleeding. Additionally, after an exhaustive analysis of NOACs, analysis showed that apixaban (RR 0.5, 95%CI 0.17–1.46) had a superior performance in major bleeding compared to rivaroxaban (RR 3.87, 95%CI 1.48–10.09).

**Conclusion:**

TIs and NOACs were superior in efficacy with minimal side effects, making them pivotal choices for both prevention and treatment of VTE. Clinical practitioners must carefully weigh drug characteristics, indications, and contraindications to optimize treatment outcomes.

**Systematic Review Registration:**

https://www.crd.york.ac.uk/PROSPERO/display_record.php?RecordID=466775.

## Introduction

Venous thromboembolism (VTE) refers to abnormal blood coagulation in the veins, including deep vein thrombosis (DVT) and pulmonary thromboembolism (PTE), ranking as the third leading global cause of mortality (Klemen et al., 2020). It affects approximately 10 million individuals annually worldwide. The incidence of VTE varies globally, with notably higher occurrences in high-income nations in comparison to low-income ones, ranging from 1-2 per 1,000 person-years in Western Countries to less than 1 per 1,000 person-years in Eastern Countries (Siegal et al., 2021). Additionally, VTE imposes a substantial economic burden, with estimated healthcare costs ranging from EUR 1.5–2.2 billion for annual hospitalizations in Europe and USD 7–10 billion in the United States (Barco et al., 2016). Survivors of VTE events face significant financial burdens, incurring costs ranging from USD 12,000–14,000 in the initial year post-event per individual (Grosse et al., 2016). Various risk factors contribute to VTE development, including progressive cancer, severe trauma or fractures, prolonged surgery, among others, with many cases being idiopathic (Khan et al., 2021a; Weiser et al., 2015; Chopard et al., 2020; Jackson et al., 2022) VTE, characterized as a chronic ailment, exhibits a notable recurrence rate and high mortality, correlating with significant hemorrhagic events attributed to anticoagulant therapy (Khan et al., 2021a; Tritschler et al., 2018). Thus, the establishment of effective treatment and prevention strategies for VTE is imperative.

The principal approach to VTE management revolves around anticoagulation therapy (Wells et al., 2014). Pharmacotherapeutic options encompass unfractionated heparin (UFH), low molecular weight heparin (LMWH), vitamin K antagonists (VKAs), antiplatelets (AP), novel oral anticoagulants (NOACs), and direct thrombin inhibitors (TIs) (Wells et al., 2014). At present, many teams have conducted comparative analysis on the efficacy and safety of these drugs, but the contradictory conclusions can not provide optimal options for VTE patients. Heparin and VKAs are classic drugs used in the clinical treatment of thrombotic diseases, and serve as the cornerstone for treating acute VTE in non-cancer patients (Ortel et al., 2020). After introduction of NOACs, comprising factor Xa inhibitors (rivaroxaban, apixaban, and edoxaban) and the thrombin inhibitor dabigatran (Tritschler et al., 2018), they have been recommended in most patients give their ease of administration, effectiveness, and favorable adverse effect profile. Meta-analyses comparing DOACs with LMWH in cancer patients suggest that DOACs are associated with a lower risk of recurrent VTE but a higher risk of major bleeding (Mulder et al., 2020). A nationwide propensity score matched study revealed higher rates of gastrointestinal bleeding with rivaroxaban compared to apixaban and dabigatran across all treatment indications (Ingason et al., 2021). Similarly, another population-based cohort study affirmed that new users of apixaban for VTE had lower rates of recurrent VTE and bleeding compared to new users of rivaroxaban (Dawwas et al., 2022). While these findings provide insights for clinicians in assessing various anticoagulation therapies for VTE, direct comparisons between DOACs remain insufficient (Ortel et al., 2020; O'Toole et al., 2023). Discrepancies in conclusions arise due to variations in retrieval strategies and participant inclusion criteria. Hence, further research is warranted to evaluate diverse anticoagulation regimens for better clinical guidance.

To date, there is a paucity of comprehensive evaluations comparing the efficacy and safety of various anticoagulants. Addressing these challenges, we conducted a systematic review and network meta-analysis of randomized controlled trials, along with subgroup analyses of DOACs, to assess relative efficacy, safety profiles, and distinctions among anticoagulants for VTE treatment and prevention respectively.

## Methods

### Data sources and searches

Our study was registered in PROSPERO (CRD42023466775). We conducted a systematic literature search on relevant topics according to a predefined protocol and strictly followed the Preferred Reporting Items for Systematic Reviews and Meta-Analyses (PRISMA) 2020 statement (Page et al., 2021). The detailed and systematic search strategy was recorded in Supplementary Appendix (page 6–7), aiming to collect all studies available in databases as Medline, Embase, and Cochrane Library (CENTRAL) from inception to 1 August 2023. We set the research type of article as RCT and the language as English, but did not restrict them by publication year or type.

### Study selection

Two researchers (WF and MZ) independently assessed potentially eligible studies, resolving any disagreements through discussion or, if necessary, through third-party adjudication. Included studies must include the following information: (1) study subjects were those with VTE or at high risk of thrombosis who required treatment or prophylactic anticoagulants; (2) a definite diagnosis of VTE in the study was made by objective examination methods; (3) the study should include one or more outcome indicators; (4) the study must be a randomized controlled trial. Studies that included the following information were excluded: (1) patients received anticoagulation therapy because of atrial fibrillation, peripherally inserted central catheter, unstable angina, acute myocardial infarction, transient ischemic attack, acute ischemic stroke, or other non-VTE diseases; (2) studies on combination drug therapy; (3) non-English literature; (4) reviews, reports, meta-analyses, animal experiments, crossover design experiments, and non-randomized controlled trials. We included studies that evaluated these interventions against with placebo or compared to each other. Finally, we divided the included articles into two parts for analysis: treatment and prevention.

### Data extraction and quality assessment

We extracted data related to study settings, baseline characteristics, interventions, and clinical outcomes by filtering titles and abstracts, and then screening the full text. In this process, results were allocated according to the intention-to-treat principle. Only results occurring during the administration of the drugs or during the observation period were included in the subsequent analysis.

To resolve any disagreements, two researchers independently assessed the risk of bias for individual studies using the Cochrane risk of bias tool (Sterne et al., 2019). This tool was used to evaluate five potential biases, including bias in the randomization process, deviation from the intended intervention, bias due to missing outcome data, bias in outcome measurement, and bias in the selection of reported results. Details of the assessment methods were provided in the Supplementary Appendix (page 12). Any differences in assessments were resolved through discussion or, if necessary, by third-party adjudication.

### Outcome measures

It was supposed that the potential beneficial outcome was a reduction in the recurrence or incidence of VTE, which was regarded as the primary outcome measure. VTE was defined as symptomatic PE involving segmental or more proximal pulmonary arteries, fatal PE, symptomatic DVT, incidental VTE detected fortuitously during imaging, or unexplained death where PE could not be ruled out. Throughout both treatment and prevention phases, the primary safety endpoint, major bleeding, remained consistent. Major bleeding was defined as bleeding that meets one of the following conditions: significant clinical symptoms, decrease in hemoglobin by 2 g/dL (i.e., 1.25 mmol/L) or more, need for transfusion of at least 2 units of whole blood or red blood cell suspension, bleeding occurring in critical sites such as intracranial, intraspinal, intraocular, pericardial, intra-articular, intramuscular with compartment syndrome, retroperitoneal, or bleeding leading to death. We also included and extracted data on secondary outcome measures, including clinically relevant non-major bleeding (CRNMB), all-cause mortality, VTE related death, fatal bleeding and adverse events. Specific definitions were detailed in Supplementary Appendix (page 8).

### Statistical analysis

In this study, the network package in STATA software (version 16.0; StataCorp LLC, United States) was used to create network plots, conduct consistency analysis between studies, and draw funnel plots. Additionally, the gemtc and ggplot packages in R software (version 4.2.1; R Foundation for Statistical Computing, Austria) were used to perform network meta-analyses based on Bayesian generalized linear models (Riaz et al., 2022), with further analysis conducted using the JAGS software. To clearly illustrate the network structure and connections between nodes, a network plot was generated for each result, followed by the creation of nodal plots for the relationships between individual drugs. For the network meta-analysis, a frequentist framework with multivariate random effects was used to fit the model, explaining the correlation of effect sizes between more than two groups of trials. The modeling of odds ratios (ORs) and 95% confidence intervals (CIs) was conducted using the Markov chain Monte Carlo method (Wang et al., 2020), We set the number of chains as 4, tuning iterations as 20,000, simulation iterations as 50,000, thinning interval as 1, inference samples as 10,000, and variance scaling factor as 2.5. The convergence effect is mainly examined by the Potential Scale Reduction Factor (PSRF). If the value of PSRF is close to 1, it indicates that the convergence is complete. We conducted the Bayesian network meta-analysis using the BUGSnet package (Liu et al., 2023), which automatically applies weakly informative priors for all model parameters, including treatment effects and intercepts. This ensures the model remains flexible and primarily data-driven, without the need for explicit prior specifications. Manual exclusion was performed prior to analysis regarding studies in comparison of the same drugs.

To demonstrate the comparison of network estimates, we used forest plots and a ranking table of relative treatment effects. The ranking was carried out based on the surface under the cumulative ranking curve (SUCRA). SUCRA, which is a metric where values nearer to 100% suggest more favorable rankings, was utilized to take into account the average certainty of one treatment with respect to another. We also employed fixed-effect and random-effects models with vague priors. The evaluation of model fitting was based on the comparison of residuals to the number of unconstrained data points and the deviance information criterion. To ensure model convergence, we used trajectory plots and convergence diagnostic plots in R software for analysis. Additionally, we compared the distribution of characteristics between the study groups categorized by treatment to assess the transitivity assumption of indirect comparisons. Heterogeneity was assessed by estimating the variance between studies (χ^2^ test and I^2^ statistic). Local inconsistency was evaluated using the node-splitting method.

The confidence in network meta-analysis (CINeMA) framework (Nikolakopoulou et al., 2020) which serves as a dedicated framework for appraising the confidence in evidence obtained from network meta-analysis, was used to evaluate the quality of randomized controlled trials, and the grading of recommendations assessment, development and evaluation (GRADE) approach (Izcovich et al., 2023) was used to assess the level of the evidence for each pairwise comparison as high, moderate, low, or very low. We calculated the absolute effects of drug therapies by comparing the relative effect sizes between drug treatment groups and placebo. This integrated approach facilitated the comprehensive assessment of the level of evidence and enabled a more accurate understanding of the effects of different treatments. To rank the treatments by effect size and level of evidence, we employed the minimally contextualized network meta-analysis method (Brignardello-Petersen et al., 2020), for better assessing the effects of different treatments and determining their order of superiority. Drugs were classified and ranked based on whether the point estimate of the effect size was above or below the null value of 1 for placebo treatment, and whether the 95% CI overlapped with this threshold. Accordingly, drugs classified as most effective had a point estimate below 1 and a 95% CI that did not overlap with the null value. Drugs classified as least effective had both a point estimate and the entire 95% CI above the null value. Finally, if serious inconsistencies were identified in the evidence, we considered the evidence with higher level between direct and indirect evidence as the best evidence.

### Subgroup analysis and sensitivity analysis

Given the widespread clinical use and increasing importance of NOACs (Chan et al., 2020), we conducted a subgroup analysis focusing solely on this class of drugs. We extracted studies where NOACs were used as the treatment group and other drug interventions as the control group. Additionally, studies that directly compared specific NOACs with placebos from the included literature were also selected for analysis. Subgroup effects were evaluated using the Meta package in R Studio 4.2.1. Furthermore, I^2^ was used to estimate the heterogeneity between studies in each pairwise comparison. For studies with substantial heterogeneity, we employed a random-effects model for incorporation. We used the one-by-one elimination method for sensitivity analysis to observe the overall stability of the studies.

## Result

**FIGURE 1:**
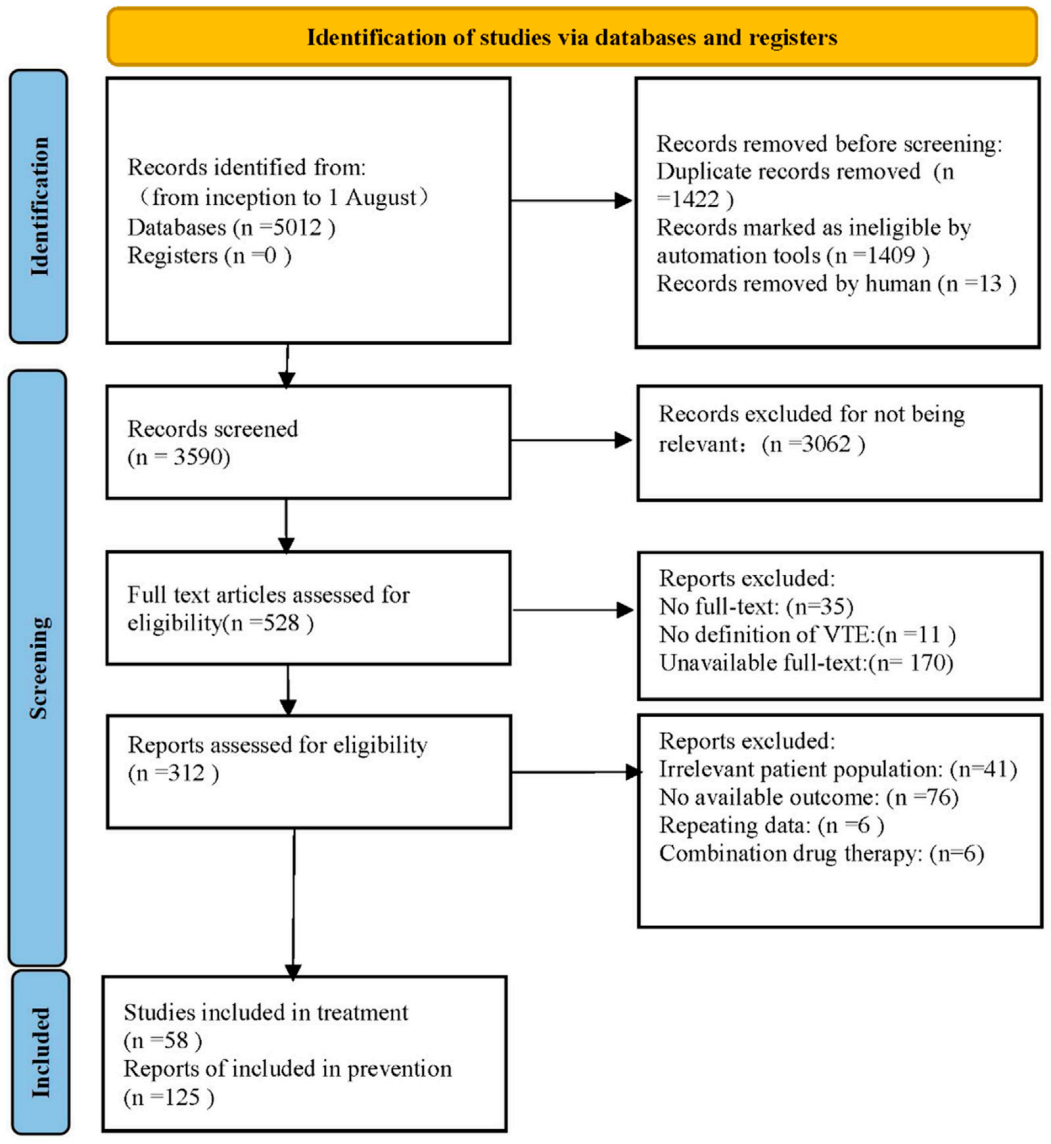
Flowchart outlining the strategy for literature search and screening used in this study.

After retrieving 5,012 articles, our team assessed the eligibility of 528 studies in full text, ultimately including 183 studies that satisfied the inclusion and exclusion criteria. PRISMA flow diagram was shown in Figure 1. Among the included trials, there comprised 58 studies (81 paired comparisons) involving 119,417 patients about VTE treatment, and 125 studies (229 paired comparisons) involving 225,414 patients about VTE prevention. The median sample size was 646 individuals (range: 48–35,756 individuals) in treatment, while the median sample size was 722 individuals (range: 76–23,257 individuals) in prevention. The full description of study baseline characteristics was listed in Supplementary Appendix (page 14–29). In studies about VTE treatment, all patients had VTE, typically defined as lower limb DVT, PTE, or both, with 12.5% of trials recruiting patients with cancer-associated thrombosis. In studies about VTE prevention, patients in 65.9% trials required hip or knee replacement surgery, patients in 8.8% trials had various types of cancer, and the remaining 25.3% trials included hospitalized patients, intensive care unit (ICU) patients, coronavirus disease 2019 (COVID-19) infected patients, and patients with acute infections. Bias risk primarily occurred in outcome measurement and randomization processes (Supplementary Appendix page 30–47). 34.3% of studies about treatment were assessed to be at low risk of bias, 59.3% at unclear risk, and 6.2% at high risk. In contrast, 38.8% of studies about prevention were assessed to be at low risk, 46.7% at unclear risk, and 14.5% at high risk.

### Primary outcomes

**FIGURE 2:**
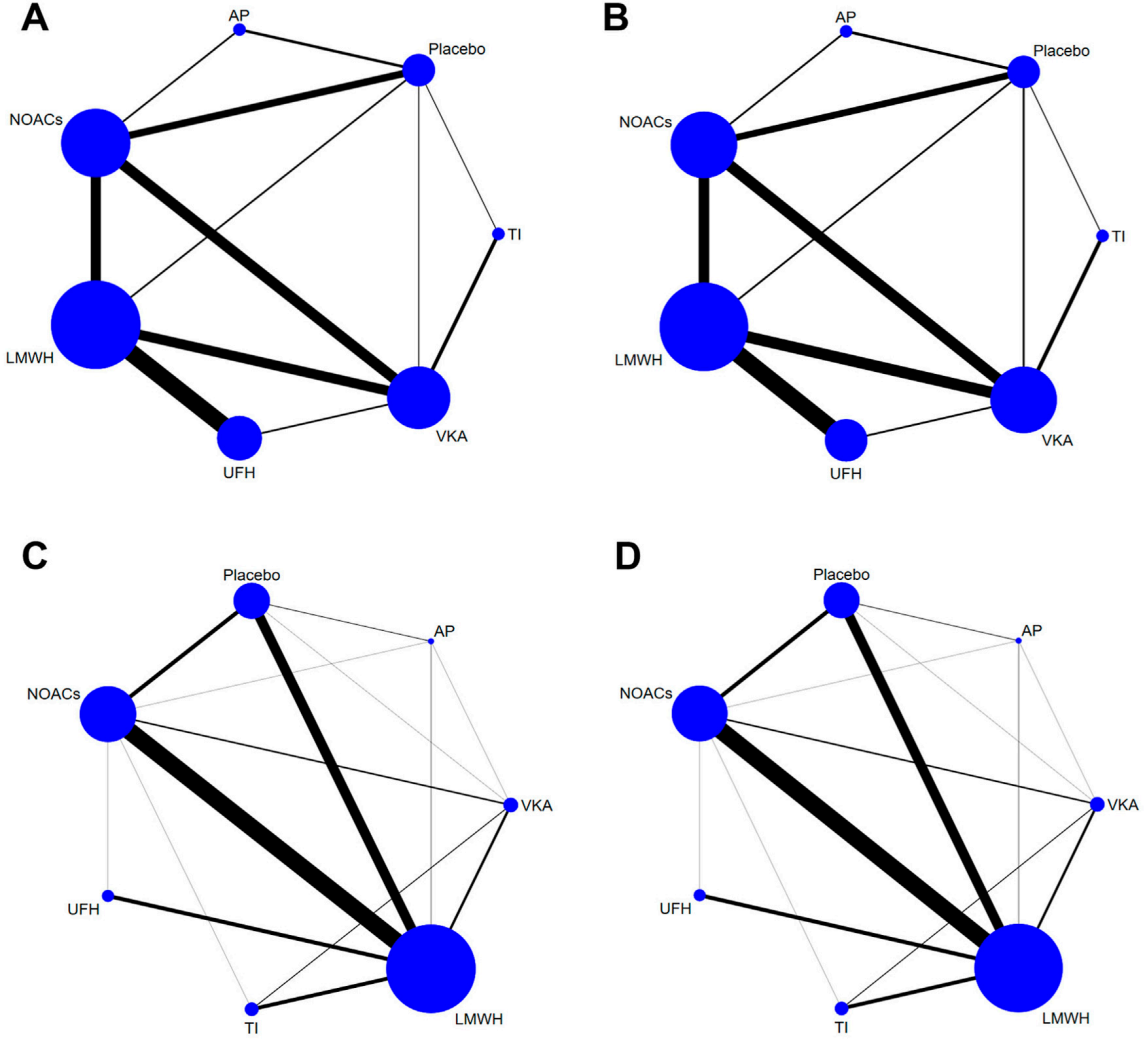
Network plots of trials evaluating various anticoagulants for VTE treatment and prevention. Network comparisons for recurrence of VTE **(A)** and major bleeding events **(B)** in studies about VTE treatment, and incidence of VTE **(C)** and major bleeding events **(D)** in studies about VTE prevention. Each node represents an anticoagulant or Placebo, with its size proportional to the number of randomized participants. Each line connecting two nodes represents a direct comparison between the two drugs, with the thickness proportional to the number of trials directly comparing the two drugs. VTE, Venous thromboembolism; LMWH, low molecular weight heparin; NOAC, novel oral anticoagulants; TI, thrombosis inhabit; VKA, Vitamin K antagonists; UFH, Unfractioned Heparin; AP, Antiplatelet drug.

**FIGURE 3:**
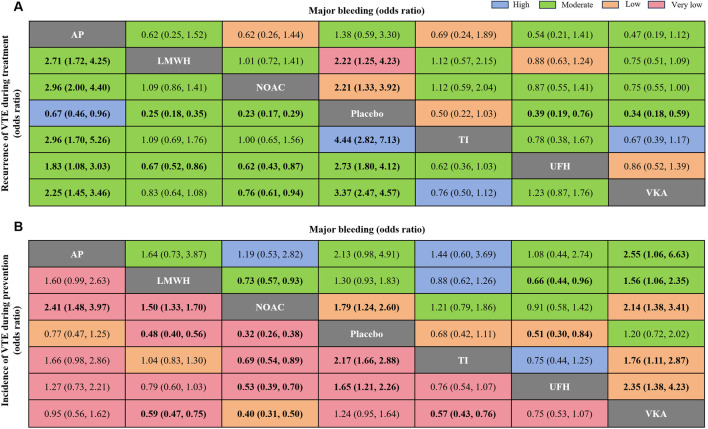
League tables of primary outcome analyses. **(A)** Recurrence of VTE and major bleeding events for treatment; **(B)** incidence of VTE and major bleeding events for prevention. The drugs are compared positively in terms of odds ratios (ORs) and 95% confidence intervals (CIs). The graph should be read from left to right: an odds ratio <1 favors the effect reduction of pharmacological intervention. To obtain the OR values for reverse comparisons, the reciprocal should be taken. The color of each cell indicates the certainty of evidence. Bold values indicate statistical significance. VTE, Venous thromboembolism; LMWH, low molecular weight heparin; NOAC, novel oral anticoagulants; TI, thrombosis inhabit; VKA, Vitamin K antagonists; UFH, Unfractioned Heparin; AP, Antiplatelet drug.

Figure 2 showed the network plot of the primary outcome, with the comparisons between VKAs and NOACs being the largest proportion in studies about treatment, followed by comparisons between LMWH and UFH. For prevention, comparisons of NOACs and LMWH were the largest proportion of studies, followed by comparisons between LMWH and Placebo. Other network plots could be found in Supplementary Appendix (page 48–59). Figure 3 showed the ranking table of network estimates for the comparison of primary outcome events (Supplementary Appendix page 84–94). If OR value was less than 1, it indicated that the drug on the left was superior to the one on the right, and vice versa for value greater than 1. The Appendix also provide GRADE assessment summary tables (Supplementary Appendix page 60–77).

**FIGURE 4:**
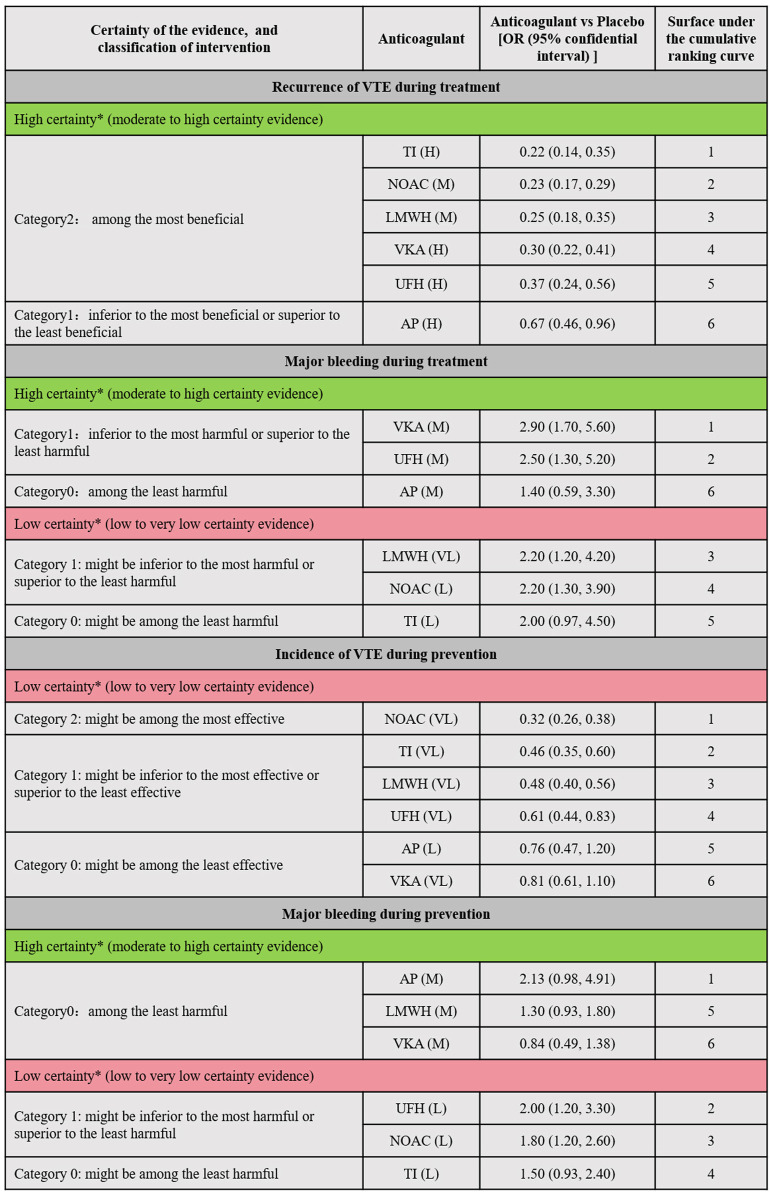
Summary of the evidence for primary outcomes. Placebo was designated as the reference group for comparison. Each anticoagulant was assessed based on whether the 95% confidence interval (CI) of its effect size intersected the decision threshold. Those showing no significant difference compared to Placebo were categorized as “Category 0,” while those demonstrating a difference to Placebo fell into “Category 1.” For efficacy outcomes (recurrence or incidence of VTE), a secondary classification was conducted based on the differences between interventions. The intervention with the smallest effect size in Category 1 served as the reference, with interventions showing greater effectiveness classified into Category 2. Conversely, for safety outcomes (major bleeding), the anticoagulant with the largest effect size in Category 1 was used as a reference, with interventions demonstrating poorer safety outcomes classified into Category 2. Interventions were further categorized into high and low reliability groups using the grading of recommendation, assessment, development, and evaluation (GRADE) approach, and the consistency of classification was verified through ranking results. This ensured that the top-ranked interventions were either the most effective or the most harmful, with categories having no impact on judgments regarding treatment and prevention. * The two major groups (high-quality evidence group and low-quality evidence group) were based on the GRADE quality of evidence for comparing anticoagulants with Placebo. High certainty (Green part of the table; includes comparisons with moderate (M) to High level (H) of evidence); Low Certainty (Red part of the table; includes comparisons with low (L) to very low (VL) quality of evidence). VTE, Venous thromboembolism; LMWH, Low Molecular Weight Heparin; NOAC, Novel Oral Anticoagulants; TI, thrombosis inhabit; VKA, Vitamin K Antagonists; UFH, Unfractioned Heparin; AP, Antiplatelet drug.

Figure 4 illustrated the minimally contextualized framework reflecting VTE associated events. This framework ranked drugs from best to worst based on efficacy and level of evidence with placebo as a control. For treatment, placebo resulted in 15 cases of VTE recurrence per 1,000 person-years. All anticoagulants significantly reduced the recurrence rate of VTE compared to placebo, with ORs and 95% CIs all less than 1. Specifically, the ORs and 95% CIs for TIs, NOACs, LMWH, UFH, and VKAs were 0.22 (0.14–0.35), 0.23 (0.17–0.29), 0.25 (0.18–0.35), 0.30 (0.22–0.41), and 0.37 (0.24–0.56) respectively. These drugs demonstrated significant superiority over AP in reducing VTE recurrence, with high-quality evidence supporting their comparisons. However, evidence level for prevention was lower. The incidence of VTE with placebo prevention was 4.3%. NOACs exhibited the highest efficacy compared to placebo, with an OR of 0.32 (95% CI 0.26–0.38), followed by TIs (OR 0.46, 95% CI 0.35–0.60), LMWH (OR 0.48, 95% CI 0.40–0.56), and UFH (OR 0.61, 95% CI 0.40–0.83). No significant differences were found between other drugs and placebo.

**FIGURE 5:**
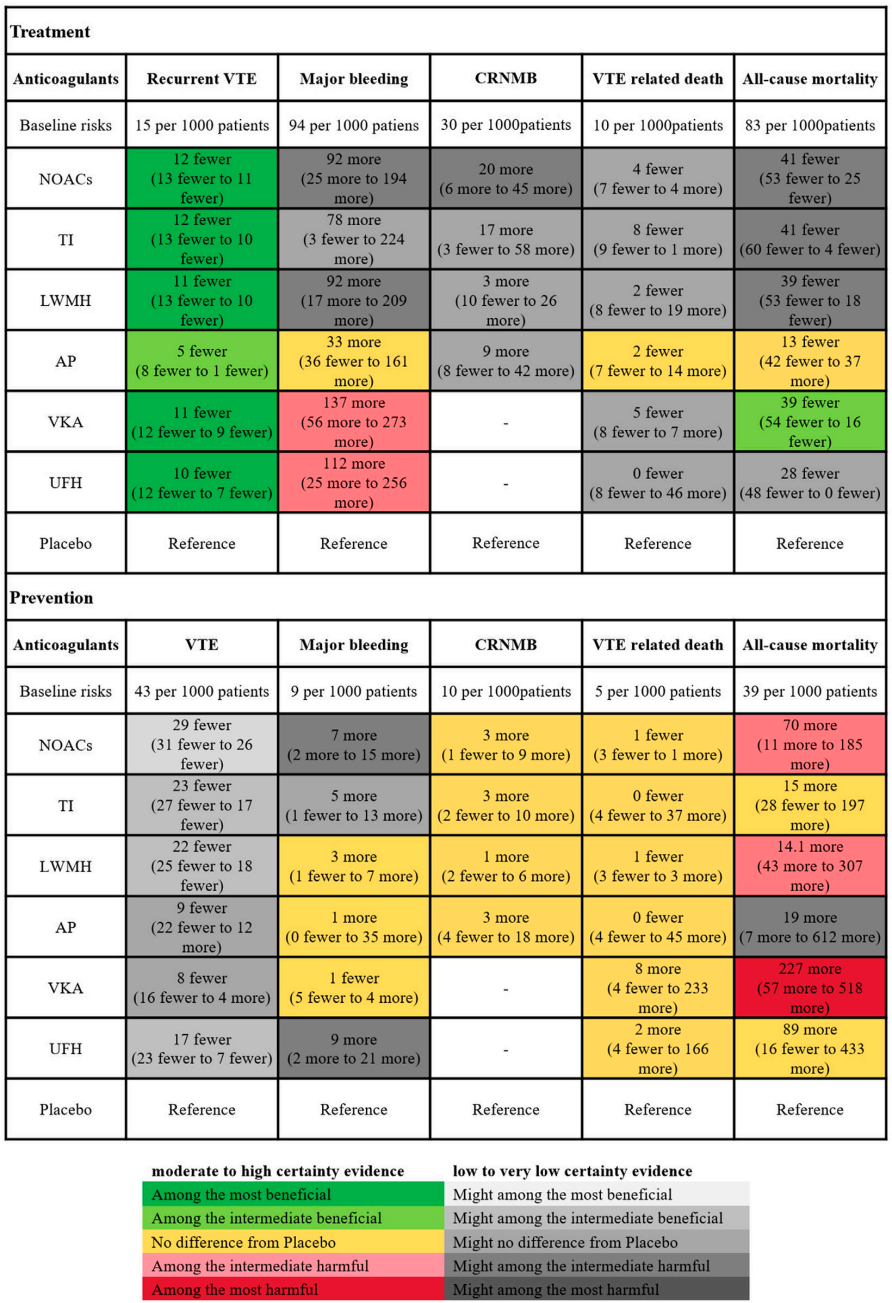
Summary of the absolute effects of anticoagulants. The certainty of evidence was assessed using the grading of recommendations assessment, development, and evaluation (GRADE) criteria. The figure illustrated the absolute benefit or harm of anticoagulant for VTE by estimating the differences in risk per 1,000 patients compared to Placebo. Anticoagulants were categorized based on their statistical significance compared to Placebo, with the categories of best, intermediate, and worst indicating the clinical significance of the effect. The certainty of evidence reflected the trustworthiness of these findings. VTE, Venous thromboembolism; CRNMB, Clinical relevant non-major bleeding; LMWH, Low Molecular Weight Heparin; NOAC, Novel Oral Anticoagulants; TI, thrombosis inhabit; VKA, Vitamin K Antagonists; UFH, Unfractioned Heparin; AP, Antiplatelet drug.

For treatment, placebo resulted in 94 major bleeding events per 1,000 person-years. In comparison to placebo, LMWH (OR 2.2, 95% CI 1.2–4.2), NOACs (OR 2.2, 95% CI 1.2–3.9), UFH (OR 2.5, 95% CI 1.3–5.2), and VKAs (OR 2.9, 95% CI 1.7–5.6) significantly increased the risk of major bleeding (evidence level ranging from low to high, as shown in Supplementary Appendix page 78–82). For prevention, the absolute risk of major bleeding associated with placebo was 0.9%. Among anticoagulants, only UFH (OR 2.0, 95% CI 1.2–3.3) and NOACs (OR 1.8, 95% CI 1.2–2.6) showed significant increased risks in bleeding events. Figure 5 showed the absolute effect sizes of anticoagulants compared to Placebo. For treatment, there were no significant differences among LMWH, NOACs, VKAs, UFH and TI in preventing VTE recurrence, while AP was the most effective in reducing major bleeding. For prevention, NOACs were the most effective in minimizing the risk of VTE, while VKAs were the most effective in reducing major bleeding events.

### Secondary outcomes

For treatment, we included 37 trials (83,270 patients) in the outcome of CRNMB, 20 trials (35,143 patients) in the outcome of VTE-related mortality, 22 trials (34,084 patients) in the outcome of fatal bleeding, and 55 trials (43,817 patients) in the outcome of all-cause mortality. Network nodes consisted of LMWH, NOACs, VKAs, UFH, AP, and Placebo. In the outcome of adverse events, 9 trials (18,503 patients) were included, and the network nodes consisted of VKAs, NOACs, TIs, LMWH, and Placebo. Compared to placebo, NOACs (OR 1.7, 95% CI 1.2–2.6) and VKAs (OR 2.1, 95% CI 1.4–3.5) may increase the incidence of CRNMB. Except for UFH and AP, all drugs may lead to a decrease in all-cause mortality, but the differences were not significant. For other outcomes, no statistically significant differences could be found between any comparison of the drugs.

For prevention, we included 92 trials (89,824 patients) in the outcome of CRNMB, and the network nodes consisted of NOACs, TIs, LMWH, AP, and Placebo. We included 18 trials (44,619 patients) in the outcome of VTE-related mortality, 86 trials (96,348 patients) in the outcome of all-cause mortality analysis, and the network nodes included LMWH, NOACs, VKAs, UFH, AP, and Placebo (Supplementary Appendix page 48–59). The pooled results showed no statistically significant differences in CRNMB and fatal bleeding events between drugs (low to moderate evidence level). Except for UFH and TIs, other drugs increased the incidence of all-cause mortality (Supplementary Appendix page 94–97).

### Subgroup analysis

In terms of treatment, subgroup analyses showed that (Supplementary Appendix page 127–142), NOACs had respective relative risk (RR) values of 0.840 (0.723, 0.977), 0.268 (0.174, 0.413), and 0.319 (0.215, 0.473) compared to VKAs, Placebo, and AP, indicating better efficacy in reducing the recurrence of VTE. However, NOACs showed higher heterogeneity (I^2^) compared to Placebo, with a P-value less than 0.1. Compared to Placebo, NOACs increased the incidence of CRNMB but reduced the incidence of all-cause mortality (RR values did not exceed the null line of 1). For other outcomes, there were no statistically significant differences between NOACs and AP. Furthermore, by analyzing NOACs in detail, we only found that apixaban was superior to rivaroxaban in the outcome of major bleeding (P < 0.05).

In terms of prevention, NOACs reduced the incidence of VTE (RR 0.602, 0.459–0.789) compared to placebo, but increased the incidence of CRNMB (1.371, 1.011–1.858). By analyzing the outcomes of VTE incidence and VTE related death, we found that NOACs were more effective than VKAs, but the risk of major bleeding was still higher. The remaining comparisons showed no significant differences. In 12 trials (28,371 patients) about prophylactic use of NOACs, differences in preventing VTE occurred among apixaban, rivaroxaban, and TTP889 (factor IX antagonist) (P < 0.05 between subgroups). Results suggested that the efficacy of TTP889 was inferior to that of the other two drugs.

### Heterogeneity analysis and sensitivity analysis

Apart from studies on the incidence of VTE in prevention, where the heterogeneity (Supplementary Appendix page 98–113) *p*-value was less than 0.05, the rest of the *p*-values were all greater than 0.1, indicating good heterogeneity in the other studies. For treatment, sensitivity analysis (Supplementary Appendix page 143–147) showed that the results for the outcomes of major bleeding and CRNMB were unstable, mainly due to the study by Weycker et al., while the remaining results were relatively stable. Egger’s test for the analysis of CRNMB had a *p*-value less than 0.05. For prevention, publication bias existed in the outcome of VTE incidence and CRNMB, for the *p*-values less than 0.05 from Egger’s test. The *p*-values from both the Egger’s and Begg’s tests for the remaining outcome events were all greater than 0.05.

## Discussion

In our review, the network meta-analysis approach was employed to analyze 183 studies involving 344,831 participants, enabling the comprehensive evaluation of the efficacy of different anticoagulants in the treatment and prevention of VTE. For treatment, moderate to high-level evidence suggested that NOACs, LMWHs, and TIs had strong efficacy in reducing VTE recurrence, while other anticoagulants could also lower the risk of thrombus formation, although with relatively lower levels of evidence regarding bleeding risks. For prevention, the evidence for thrombus formation improvement was weaker, but other outcomes were similar to treatment. Evidence for VTE-related mortality, bleeding-related mortality, and adverse events was low and inconclusive. Most preventive medications may increase mortality rates, but other studies indicated that anticoagulants could reduce mortality during the prevention process (O'Toole et al., 2023). Our subgroup analysis also indicated that NOACs and VKAs did not increase mortality, with wide confidence intervals possibly due to underlying conditions or limited outcome data.

NOACs and TIs are currently widely used due to their rapid onset, predictable efficacy, and fixed dosing without the need for routine coagulation monitoring (Gelosa et al., 2018). Consistent with previous research (Konstantinides et al., 2020; Schulman et al., 2020), they can be viable options, showing comparable effectiveness in treatment and prevention by significantly reducing the risk of thrombosis without causing major adverse events. However, VKAs, in contrast, require monitoring (Bauman et al., 2022) and pose a higher bleeding risk, as confirmed by meta-analyses (Khan et al., 2021b). Besides the observation that NOACs outperformed VKAs in fatal bleeding events, there were no significant differences identified in other outcomes across the drugs. This might be attributed to VKAs being non-selective oral anticoagulants with multiple targets (II, VII, IX, X). VKAs inhibit not only the production of thrombin by acting on upstream coagulation factors during the blood clotting “waterfall reaction,” but also impede factor II activation to acquire activity (Heestermans et al., 2022).

LMWH may be superior to NOACs in terms of bleeding, a slight variation from previous studies by Chen et al. (2021), which could be attributed to differences in participant selection and baseline data. However, the method of administration for LMWH may introduce certain inconveniences. UFH, as a traditional anticoagulant, has fewer adverse reactions but weaker thrombus reduction. Despite discrepancies with prior research (Matar et al., 2018), through mechanistic analysis and in conjunction with other studies (Wells et al., 2014; Hogwood et al., 2023), we believe LMWH holds an advantage due to its sustained activity relationship, allowing for once-daily dosing without coagulation monitoring (Spadarella et al., 2020). APs did not significantly improve thrombus formation nor increase the risk of adverse reactions, likely because they mainly affect platelet activation rather than interfering with the coagulation system (Abubakar et al., 2023), thereby hardly leading to severe bleeding events.

In our analysis, NOACs exhibited exceptional efficacy in both the treatment and prevention of VTE. By targeting factors IIa and Xa and affecting both intrinsic and extrinsic coagulation pathways, NOACs represent promising therapeutic alternatives, notably mitigating thrombosis risk (Konstantinides et al., 2020; Schulman et al., 2020). However, NOACs may also elicit variable adverse reactions. As Ingason et al. (2021) elucidated, apixaban and dabigatran are associated with a lower incidence of gastrointestinal bleeding compared to rivaroxaban. Likewise, our subgroup analysis indicated that while apixaban did not confer superior efficacy over rivaroxaban in preventing VTE recurrence, rivaroxaban carried a heightened risk of major bleeding. Therefore, it is advised that patients with a high risk of gastrointestinal bleeding avoid rivaroxaban as well as high doses of dabigatran and edoxaban (Loffredo et al., 2015). The primary etiology of major bleeding stems from the fact that NOACs serve as substrates of permeability glycoprotein, which is abundantly present along the gastrointestinal tract, and facilitates the efflux of drugs and toxins into the intestinal lumen (Wolking et al., 2015). Consequently, the concurrent use of NOACs with potent permeability glycoprotein inhibitors is not recommended (Mar et al., 2022; Steffel et al., 2021). Thus, the prudent utilization of NOACs necessitates consideration of patients’ characteristics, including age, renal and hepatic function, pregnancy status, concurrent medications, as well as weighing the associated benefits and risks (Schwarb and Tsakiris, 2016). While some clinicians aim to minimize these adverse reactions, comparative decision-making might be helpful in fully informing patients about the benefits and risks of anticoagulant therapy.

### Benefits and limitations

Previous reports may have limitations, such as focusing solely on specific diseases or only addressing treatment or prevention measures (Eck et al., 2022; Kirkilesis et al., 2020; Feng et al., 2021). Our study comprehensively evaluated the comparisons between all anticoagulants and antiplatelet medications, along with subgroup analyses of anticoagulants compared to placebos. Furthermore, we found that anticoagulants had opposite effects on the impact of all-cause mortality between treatment and prevention aspects. We conducted a subgroup analysis of NOACs alone, compared the direct results with the network results, and combined other studies to comprehensively compare the efficacy between NOACs and other drugs, leading to more reliable results. For the included trials, we calculated the absolute treatment effects and the range of the control event rates, and graded the level of evidence based on these absolute treatment effects rather than network ORs. Finally, we provided a transparent framework (Izcovich et al., 2023) for evidence summaries and a minimally contextualized framework (Brignardello-Petersen et al., 2020), further enhancing the credibility and visual display of drug efficacy results, providing a systematic approach for clinical practice recommendations.

However, our review also had some limitations. Many anticoagulants included lacked direct comparative research, which could introduce bias. Secondly, due to the lack of integrated individual patient data and the selection of subgroups just from studies included in the network, the accuracy of the results of subgroup effects was affected, and the inconsistency of VTE incidence outcomes was also influenced. Moreover, our study excluded non-English language studies, potentially impacting the comprehensiveness of the included researches. Baseline data were missing in some included trials, and there were variations in population characteristics, drug treatment times, and follow-up times in the studies, resulting in uncertainty in the CIs. Although sensitivity analysis showed that different interventions, follow-up times, and basic patient characteristics did not significantly impact the pooled results, literature also indicated that the use of anticoagulants like NOACs requires careful consideration of tumor-related factors, concurrent therapies, and age-related renal function decline to mitigate bleeding risks (Beyer-Westendorf and Ageno, 2015). Publication bias in CRNMB and VTE incidence (Egger’s test, *p* < 0.05) may arise from selective reporting, limited studies, and exclusion of gray literature. Methods like trim-and-fill or Bayesian modeling can adjust for missing studies, improving analysis reliability. Addressing heterogeneity through sensitivity analyses, subgroup analyses, or covariate adjustment can identify variability sources and enhance result robustness.

## Conclusion

In summary, network and subgroup analyses have underscored the efficacy of TIs and NOACs in reducing thrombosis with minimal side effects, making them pivotal choices for both prevention and treatment of VTE. While LMWHs offer advantages over UFH, personalized treatment regimens are still needed based on patient condition. Adverse events associated with UFH and VKAs often stem from monitoring complexity and dosing challenges. Clinical practitioners must carefully weigh drug characteristics, indications, and contraindications to optimize treatment outcomes. Majority of our findings are based on moderate to high evidence level, thus serving as a reliable reference and source of information for the clinical decision-making process. In future exploration, head-to-head trials, especially those regarding novel oral anticoagulants (NOACS), are of utmost importance. Through such trials, it will be expected to further optimize anticoagulation strategies to prevent and treat VTE, reduce mortality, and effectively manage bleeding risks.

## Data Availability

The original contributions presented in the study are included in the article/Supplementary Material, further inquiries can be directed to the corresponding authors.

## Abbreviations

VTE, venous thromboembolism; DVT, deep vein thrombosis; PTE, pulmonary thromboembolism; CINeMA, confidence in network meta-analysis; GRADE, grading of recommendations assessment, development and evaluation; NOAC, novel oral anticoagulant; TI, thrombin inhibitor; LMWH, low molecular weight heparin; UFH, unfractionated heparin; VKA, vitamin K antagonist; AP, antiplatelet; CRNMB, clinically relevant non-major bleeding; PRISMA, Preferred Reporting Items for Systematic Reviews and Meta-Analyses.
