# Supplementary material for: Efficacy and safety of anticoagulants on venous thromboembolism: a systematic review and network meta-analysis of randomized controlled trials
Source: Front Pharmacol. 2025 Jan 8;15:1519869. doi: 10.3389/fphar.2024.1519869 (PMC11750681; doi:10.3389/fphar.2024.1519869)
Supplement: Supplementary file 1 [file DataSheet1.pdf]

# Table of contents


**APPENDIX 1: SEARCH STRATEGY** ........ **3**

**APPENDIX 2: SUPPLEMENTARY OF METHODS** ........ **5**

2.1 Definitions of outcomes ........ 5
2.2 Method for subgroup analyses ........ 8
2.3 Details of the sensitivity analyses ........ 8
2.4 Details of the publication bias assessments ........ 9
2.5 Details of the Cochrane Risk-of-Bias Tool for Randomized Trials (RoB2) ........ 9
2.6 Details of GRADE Minimal Contextualized Framework and the presentation tool ........ 10

**APPENDIX 3: CHARACTERISTICS OF INCLUDED STUDIES** ........ **11**

3.1 List of included studies ........ 11
*3.11 Baseline data for the treatment of venous thromboembolism ........ 11*
*3.12 Baseline data for the prevention of venous thromboembolism ........ 16*

**APPENDIX 4: RISK OF BIAS ASSESSMENTS** ........ **27**

4.1 Treatment ........ 27
4.2 Prevention ........ 30

**APPENDIX 5: OTHER MAIN RESULTS** ........ **45**

5.1 Network plots ........ 45
5.2 Summary of findings tables ........ 57
*5.2-1 AP vs. Placebo be used for treatment ........ 57*
*5.2-2 LMWH vs. Placebo be used for treatment ........ 58*
*5.2-3 NOACs vs. Placebo be used for treatment ........ 59*
*5.2-4 TI vs. Placebo be used for treatment ........ 60*

*5.2-5 UFH vs. Placebo be used for treatment* ... *61*
*5.2-6 VKA vs. Placebo be used for treatment* ... *62*
*5.2-7 AP vs. Placebo be used for prevention* ... *63*
*5.2-8 LMWH vs. Placebo be used for prevention* ... *64*
*5.2-9 NOACs vs. Placebo be used for prevention* ... *65*
*5.2-10 TI vs. Placebo be used for prevention* ... *66*
*5.2-11 UFH vs. Placebo be used for prevention* ... *67*
*5.2-12 VKA vs. Placebo be used for prevention* ... *68*
5.3 MINIMALLY CONTEXTUALIZED FRAMEWORK ... 75
*5.31 Treatment* ... *75*
*5.32 Prevention* ... *78*
5.4 NETWORK ESTIMATES (LEAGUE TABLES) ... 80
5.5 FOREST PLOTS ... 91
5.6 HETEROGENEITY ... 95
5.7 COMPARISON-ADJUSTED FUNNEL PLOTS ... 111
5.8 SUBGROUP ANALYSIS ... 124
5.9 SINGLE-DRUG ANALYSIS ... 134
5.10 SENSITIVE ANALYSIS ... 140
5.11 SUCRA RANKING DIAGRAM ... 145

**APPENDIX 6 REFERENCE ... 151**

6.1 TREATMENT ... 151
6.2 PREVENTION ... 152

## Appendix 1: Search strategy

**MEDLINE n=907**

#1 Search: ("Venous Thromboembolism"[Mesh] OR "Thromboembolism"[Mesh]) 64578

#2 Search: ((("Anticoagulants"[MeSH Terms] OR "anticoagulant drug"[Title/Abstract] OR "drug anticoagulant"[Title/Abstract] OR "anticoagulant agents"[Title/Abstract] OR "agents anticoagulant"[Title/Abstract] OR "anticoagulation agents"[Title/Abstract] OR "agents anticoagulation"[Title/Abstract] OR "anticoagulant drugs"[Title/Abstract] OR "drugs anticoagulant"[Title/Abstract] OR "anticoagulant agent"[Title/Abstract] OR "agent anticoagulant"[Title/Abstract] OR "Anticoagulant"[Title/Abstract] OR "indirect thrombin inhibitors"[Title/Abstract] OR (("antagonists and inhibitors"[MeSH Subheading] OR ("antagonists"[All Fields] AND "Inhibitors"[All Fields]) OR "antagonists and inhibitors"[All Fields] OR "Inhibitors"[All Fields] OR "inhibitor"[All Fields] OR "inhibitor s"[All Fields]) AND "indirect thrombin"[Title/Abstract]) OR "thrombin inhibitors indirect"[Title/Abstract]))) 125522

#3 Search: "Randomized Controlled Trial"[Publication Type] OR "Randomized Controlled Trials as Topic"[MeSH] OR "randomized controlled study"[Title/Abstract] OR "randomized controlled trial"[Title/Abstract] OR "randomized study"[Title/Abstract] OR "randomized trial"[Title/Abstract] OR "randomized placebo-controlled study"[Title/Abstract] OR "randomized placebo-controlled trial"[Title/Abstract] OR "randomized placebo controlled"[Title/Abstract] OR "randomized placebo-controlled"[Title/Abstract] OR "randomized double-blind"[Title/Abstract] OR "randomized double blind"[Title/Abstract] OR (randomized[Title/Abstract] AND double-blind[Title/Abstract]) OR (randomized[Title/Abstract] AND placebo-controlled[Title/Abstract]) AND (randomized controlled trial[Filter]) Filters: Randomized Controlled Trial 601631

#4 #1 AND #2 AND #3 907

**EMBASE n=2015**

#1 exp Venous Thromboembolism/ 5004

#2 (‘Anticoagulants’/exp OR ‘Anticoagulant Drug’/exp OR ‘Drug, Anticoagulant’/exp OR ‘Anticoagulant Agents’/exp OR ‘Agents, Anticoagulant’/exp OR ‘Anticoagulation Agents’/exp OR ‘Agents, Anticoagulation’/exp OR ‘Anticoagulant Drugs’/exp OR ‘Drugs, Anticoagulant’/exp OR ‘Anticoagulant Agent’/exp OR ‘Agent, Anticoagulant’/exp OR ‘Anticoagulant’/exp OR ‘Indirect Thrombin Inhibitors’/exp OR ‘Inhibitors, Indirect Thrombin’/exp OR ‘Thrombin Inhibitors, Indirect’/exp) AND ('Review'/de OR 'Clinical Trial'/de) 10899

#3 #1 and #2 2015

**Cochrane library n=1724**

#1 (Venous Thromboembolism OR Thromboembolism):ab,ti,kw 9171

#2 (Anticoagulants OR Anticoagulant Drug OR Drug, Anticoagulant OR Anticoagulant Agents OR Agents, Anticoagulant OR Anticoagulation Agents OR Agents, Anticoagulation OR Anticoagulant Drugs OR Drugs, Anticoagulant OR Anticoagulant Agent OR Agent, Anticoagulant OR Anticoagulant OR Indirect Thrombin Inhibitors OR Inhibitors, Indirect Thrombin OR Thrombin Inhibitors, Indirect):ab,ti,kw 13235

#3 #1 AND #2 in Trials 2905

#4 Randomized Controlled Trials as Topic 82019

#5 (“randomized controlled study” OR “randomized controlled trial” OR “randomized study” OR “randomized trial” OR “randomized placebo-controlled study” OR “randomized placebo-controlled trial” OR “randomized placebo controlled” OR “randomized placebo-controlled” OR “randomized double-blind” OR “randomized double blind”):ti,ab,kw 742382

#6 #3 AND (#4 OR #5) 766791

#7 #3 AND #6 1724

Searching operation

For all databases, we used the related terms or map term to subject heading options to include the terms and all the synonyms automatically.

**Appendix 2: Supplementary of methods**

**2.1 Definitions of outcomes**

| **Outcomes** | **Definition/Description** | **Property** |
|---|---|---|
| Recurrent venous thromboembolism | VTE was either:<br>Symptoms of PE with one of the following findings:<br>A (new) intraluminal filling defect in (sub)segmental or more proximal branches on spiral computed tomography (CT) scan<br>A (new) intraluminal filling defect or an extension of an existing defect or a new sudden cut-off of vessels more than 2.5 mm in diameter on the pulmonary angiogram<br>A (new) perfusion defect of at least 75% of a segment with a local normal ventilation result (high-probability) on ventilation/perfusion lung scintigraphy (V/Q scan)<br>Inconclusive spiral CT, pulmonary angiography or lung scintigraphy with demonstration of deep vein thrombosis (DVT) in the lower extremities by compression ultrasound or venography<br>Fatal PE based on autopsy or objective diagnostic testing prior to death<br>Death that could not be attributed to a documented cause and for which PE/DVT could not be ruled out (unexplained death),<br>or Symptoms of DVT with one of the following findings:<br>Abnormal compression ultrasound where compression had been normal or, if non-compressible at screening or baseline, a substantial increase (≥4 mm) in diameter of the thrombus during full compression<br>An extension of an intraluminal filling defect, or a new intraluminal filling defect or an extension of non-visualization of veins in the presence of a sudden cut-off on venography | Binary variable/ objective outcome |
| VTE related death | PE<br>Unexplained death and VTE could not be ruled out | Binary variable/ objective outcome |
| Clinically relevant bleeding | The composite of major or clinically relevant non-major bleeding | Binary variable/ objective outcome |
| Major bleeding | Major bleeding was defined as clinically overt and:<br>Associated with a fall in hemoglobin of 2 g/dL (i.e. 1.25 mmol/L) or more, or<br>Leading to a transfusion of ≥2 units of packed red blood cells or whole blood. A red cell unit was defined as the quantity of red cells obtained from or | Binary variable/ objective outcome |

| | | |
|---|---|---|
| | corresponding to approximately 500 mL of whole blood, or<br>In a critical site: intracranial, intraspinal, intraocular, pericardial, intra-articular, intramuscular with compartment syndrome, retroperitoneal, or<br>Contributing to death | |
| Clinically relevant non-major bleeding | Clinically relevant nonmajor bleeding was defined as overt bleeding not meeting the criteria for major bleeding but associated with medical intervention, an unscheduled contact (visit or telephone call) with a physician, (temporary) cessation of study treatment, or associated with discomfort for the patient such as pain or impairment of activities of daily life including:<br>Any bleeding compromising hemodynamics, or<br>Any bleeding leading to hospitalization, or<br>Subcutaneous (skin) hematoma if the size is >25 cm2 or >100 cm2if provoked, or<br>Intramuscular hematoma, or<br>Epistaxis lasting for more than 5 minutes, if the episode was repetitive (i.e. two or more episodes of true bleeding, i.e. not spots on a handkerchief, within 24 hours), or led to an intervention (packing, electrocoagulation etc.), or<br>Gingival bleeding if it occurred spontaneously (i.e. unrelated to tooth brushing or eating), or if it<br>lasted for more than 5 minutes, or<br>Hematuria if it was macroscopic, and either spontaneous or lasting for more than 24 hours after<br>instrumentation (e.g. catheter placement or surgery) of the urogenital tract, or<br>Macroscopic gastrointestinal hemorrhage: at least one episode of melena/hematemesis, if clinically apparent and hemoccult positive, or<br>Rectal blood loss, if more than a few spots on toilet paper, or<br>Hemoptysis, if more than a few speckles in the sputum and not occurring within the context of PE, or<br>Any other bleeding type that was considered to have clinical consequences for a patient<br>All other overt bleeding events not meeting the criteria for clinically relevant nonmajor bleeding were classified as trivial bleeding. | Binary variable/ objective outcome |
| All-cause mortality | VTE related death:<br>o PE<br>o Unexplained death and VTE could not be ruled out<br>· Cardiovascular cause:<br>o Myocardial infarction | Binary variable/ objective outcome |

| | | |
|---|---|---|
| | o Heart failure<br>o Ischemic stroke<br>o Other cardiac/vascular event<br>· Other cause of death:<br>o Cancer<br>o Bleeding<br>o Infectious disease<br>· Other | |
| Fatal bleeding | bleeding contribute to death | Binary variable/<br>objective outcome |
| Venous thromboembolism | Composite of total VTE,comprising VTE detected at mandatory bilateral venography, plus symptomatic DVT or pulmonary embolism (PE), and death due to any cause | Binary variable/<br>objective outcome |
| Adverse Events | Treatment-emergent serious adverse events were defined as severe if they were grade 3 or greater | Binary variable/<br>objective outcome |

## 2.2 Method for subgroup analyses

To gain a more comprehensive understanding of the benefits and drawbacks of NOACs, we conducted a subgroup analysis comparing them directly with VKAs, LMWH, antiplatelet agents, and placebos. This analysis covered all pertinent outcomes identified in the systematic review. As we strive to further assess the efficacy of NOACs, we've decided, based on peer reviewers' suggestions, to undertake a study comparing novel oral anticoagulants with placebos via subgroup analysis. Our study delved into outcomes such as recurrent venous thromboembolism, major bleeding events, clinically relevant non-major bleeding events, and all-cause mortality in the treatment of venous thromboembolism, encompassing all preventive outcome measures. Employing the meta package in R Studio, We conducted a thorough analysis comparing the efficacy of novel oral anticoagulants with several anticoagulant medications, as well as the comparison between apixaban and rivaroxaban with placebos. Subgroup analysis was employed to discern differences in efficacy among various novel oral anticoagulants and placebos, aiming to deepen our understanding of medication effectiveness.

## 2.3 Details of the sensitivity analyses

We conducted a sensitivity analysis to further explore the robustness of our results. In order to identify any studies that may disproportionately influence the overall treatment effect, we systematically removed one study at a time. We assessed whether the original meta-analysis results were significantly altered by the exclusion of certain studies. Funnel plots of treatment effects against study precision were created for the primary outcomes to investigate potential publication bias, a technique that may help determine the feasibility of additional small studies. As shown in the figures, when the majority of the literature was excluded, the pooled results from the remaining studies were not statistically significant (95% confidence intervals included or did not include 1), indicating that the original meta-analysis results remained stable with changes in study numbers, demonstrating good robustness.

## 2.4 Details of the publication bias assessments

We used a comparison-adjusted funnel plot to explore publication bias for all direct comparisons sorted by p-score. Additionally, we employed Egger's regression test and Begg's rank test to assess asymmetry as per the requirements of the paper.

## 2.5 Details of the Cochrane Risk-of-Bias Tool for Randomized Trials (RoB2)

We used the Cochrane tool to assess the quality of the selected literature, covering various aspects including bias in the randomization process, deviations from the intended intervention, bias due to missing outcome data, bias in outcome measurement, and bias in selective reporting of results. Each trial may face multiple issues within each domain, typically with five response options: "Yes（Y）," "Probably Yes (PY)," "Probably No (PN)," "No," or "No Information (NI)." Individual questions do not allow for selection of "NI." Some issues are logically linked, meaning that selecting an option for a preceding issue may lead to skipping subsequent issues; if an issue is skipped due to this logical flow, it is marked as "Not Applicable (NA)."

When assessing the risk of bias for a particular study, reviewers need to treat responses of Y, PY, N, PN, NI, NA as the same nature of answer for the question. Based on reviewers' responses to the issues, the bias risk in each domain can be classified into three levels: "low risk of bias," "some concerns," and "high risk of bias." If all domains are assessed as "low risk of bias," the overall risk of bias is considered "low risk." If some domains are assessed as "some concerns" and no domain is rated as "high risk," then the overall bias risk is categorized as "some concerns." If any domain is rated as "high risk of bias," the overall bias risk is classified as "high risk." Two reviewers conducted independent assessments, resolving discrepancies through consensus.

### 2.6 Details of GRADE Minimal Contextualized Framework and the presentation tool

We used the Confidence in Network Meta-Analysis (CINeMA) framework and web application to rate the quality of treatment effect estimates compared to placebo. The framework distinguishes six domains (within-study bias, reporting bias, indirectness, imprecision, heterogeneity, and incoherence). Relative effect estimates below 0.400 and above 2.500 were considered clinically important for assessing imprecision, heterogeneity, and inconsistency domains. For evaluation, we used the average risk of bias to assess bias in studies and the indirectness of each study. We then used GRADEpro tool to evaluate the average rates of each outcome measure (per 1000 patient-years of xx cases) for all placebo groups and calculated the absolute risk differences per 1000 patients treated yearly per 1000 patients. An array of icon arrays displaying absolute event rates showed the benefits and harms of secondary thrombosis prevention with different medications. We calculated the probability that each medication was the most effective option by iterating Markov chain with the highest odds ratio for each medication, calculating the absolute effect value of the smallest significant difference between the intervention group and placebo. Evidence recommendation levels can be divided into two categories: strong recommendation and weak recommendation. After analyzing the point estimate and interval estimate of drug efficacy for each outcome, they can be categorized as "most beneficial," "less beneficial than most beneficial and more beneficial than least beneficial," or "potentially least beneficial" for other outcome measures based on efficacy. By integrating the SUCRA (which is defined as a metric for ranking treatments, with higher values, closer to 100%, signifying better rankings) cumulative ranking probability plot, a distinct and unambiguous ranking of therapeutic drugs can be achieved. This aids in elucidating the average level of certainty associated with one treatment in comparison to another. Finally, by evaluating local inconsistency between direct and indirect results using node-splitting method, a comprehensive understanding of the robustness and consistency of network meta-analysis was achieved, providing strong support for future research and decision-making.

## Appendix 3: Characteristics of included studies
## 3.1 List of included studies
### 3.11 Baseline data for the treatment of venous thromboembolism

| study | year | Study Participants | dose_t | dose_c | Age | | sex(m/f) | | Interventions | | | | Treatment duration | length of follow-up |
|---|---|---|---|---|---|---|---|---|---|---|---|---|---|---|
| | | | | | | | | | T group | | Control group | | | |
| | | | | | T | C | T | C | n | drug | n | drug | | |
| Raskob | 2016 | VTE | 60mg/d 30mg/d CC 30-50ml/min | 2.0--3.0 | 56.0(43.0-68.5) | 56.0(43.0-68.0) | 588/488 | 597/487 | 1076 | Edoxaban | 1084 | Warfarin | 3-6M | 12M |
| Raskob | 2016 | VTE | 60mg/d 30mg/d CC 30-50ml/min | 2.0-3.0 | 57.0(44.0-67.0) | 56.0(42.0-67.0) | 534/362 | 497/354 | 896 | Edoxaban | 851 | Warfarin | 6-12M | 12M |
| Raskob | 2016 | VTE | 60mg/d 30mg/d CC 30-50ml/min | 2.0-3.0 | 57.0(44.0-67.0) | 56.0(44.0-68.0) | 1012/649 | 987/672 | 1661 | Edoxaban | 1659 | Warfarin | 12M | 12M |
| Schulman | 2009 | VTE | 150 mg twice daily | 2.0-3.0 | 55.0/15.8 | 54.4/16.2 | 738/535 | 746/520 | 1274 | Dabigatran | 1265 | Warfarin | 6M | 6M |
| Raskob | 2018 | DVT WITH CANCER | 60mg/d 30mg/d CC 30-50ml/min | 200IU/KG*d for 30d (<18000IU)follow 150IU/kg | 64.3/11.0 | 63.7/11.7 | 277/522-277 | 263/524-263 | 522 | Edoxaban | 524 | Dalteparin | 6-12M | 9-12M |
| Agnelli | 2013 | DVT OR PTE | 2.5mg twice daily | - | 56.6/15.3 | 57.1/15.2 | 487/840-487 | 468/829-468 | 840 | Apixaban | 829 | Placebo | 12M | 1M |
| Agnelli | 2013 | DVT OR PTE | 5mg twice daily | - | 56.4/15.6 | 57.1/15.2 | 469/813-469 | 468/829-468 | 813 | Apixaban | 829 | Placebo | 12M | 1M |
| Koopman | 1996 | DVT | 5000IU+1250/h | 8000IU<50kg<br>12300IU/50-70kg<br>18400IU>70kg | 62.0/16.0 | 59.0/17.0 | 96/102 | 107/95 | 198 | StHep | 202 | LMWH | 3M | 24W |
| Medina | 2017 | DVT(oupatients) | 60mg/d 30mg/d CC 30-50ml/min | 2--3 | 54.9/15.2 | 54.2/15.3 | 436/288 | 413/277 | 724 | Edoxaban | 690 | Warfarin | 3-12M | 12M |
| Medina | 2017 | DVT(Hospitalized) | 60mg/d 30mg/d CC 30-50ml/min | 2--3 | 56.0/16.7 | 56.2/16.5 | 1066/820 | 1073/850 | 1886 | Edoxaban | 1923 | Warfarin | 3-12M | 12M |
| Buller | 2007 | DVT OR PE | 2.5mg/week<br>1.5mg/week<br>CC<30ml | - | 60.2/15.2 | 59.9/15.4 | 317/277 | 326/295 | 594 | idraparinux | 621 | Placebo | 6M | 6M |
| Planquette | 2022 | DVT | 15mg twice daily for 3w<br>20mg for 3m | 200IU/kg for 1M<br>150u/kg for 2M | 68.6/(62.9-77.8) | 70.7(62.7-78.7) | 37/37 | 40/44 | 74 | Rivaroxaban | 84 | Dalteparin | 3M | 3M |
| Righini | 2016 | DVT | 171 IU/kg*d | - | 52/17 | 53/17 | 62/64 | 70/63 | 126 | Nadroparin | 133 | Placebo | 42D | 90D |
| Findik | 2002 | PTE | 5000IU followed by 1000IU/h | 100U/kg twice daily | 49/15 | 51/18 | 15/15 | 14/15 | 30 | UFH | 29 | Enoxaparin | 5-10D | 90D |

| | | | | | | | | | | | | | | |
|---|---|---|---|---|---|---|---|---|---|---|---|---|---|---|
| Girolami | 2004 | DVT OR PE | 4000u12500u/<50kg<br>50000u15000u/50-70 kg<br>6000u17500u/>70kg | 85U/kg twice daily | 65.7/15.6 | 67.0/14.8 | 158/202 | 167/193 | 360 | UFH | 360 | LMWH | 12W | 1-3M |
| Belcaro | 1999 | DVT | 5000IU+32ml/h(20000u in 500ml dextrose solution) | 0.1ml/kg twice daily | 53.0/10.0 | 54.0/11.0 | 57/40 | 54/44 | 97 | StHep | 98 | LMWH | 2W | 2W-3M |
| Belcaro | 1999 | DVT | 12500IU twice daily | 0.1ml/kg twice daily | 54./9.0 | 54.0/11.0 | 52/47 | 54/44 | 99 | SCHhep | 98 | LMWH | 2W | 2W-3M |
| Levine | 1996 | DVT | 1ml/kg twice daily | 5000IU+32ml/h(20000u in 500ml dextrose solution) | 57/17 | 59/15 | 153/94 | 148/105 | 247 | LMWH | 253 | StHep | 3M | 90D |
| Romera | 2009 | DVT OR PE/bleeding | 3mg followed 2.0-3.0 | 175IU/kg | 61.3/16.2 | 58.9/17.6 | 70/52 | 64/55 | 122 | Acenocoumarol | 119 | LMWH | 6M | 1-12M |
| Romera | 2009 | DVT WITH CANCER | 3mg followed 2.0-3.0 | 175IU/kg | 64.7/15.2 | 59.8/15.5 | 20/13 | 18/18 | 33 | Acenocoumarol | 36 | LMWH | 6M | 1-12M |
| Ridker | 2003 | VTE | - | 1.5-2.0(<10mg/d) | 53(47-64) | 53(46-65) | 133/120 | 135/120 | 253 | Placebo | 255 | Warfarin | 28D | 2.1Y |
| Kearon | 2006 | DVT | 333U/kg<br>followed by<br>250 U/kg twice daily | 100u/kg | 60/17 | 60/16 | 182/173 | 206/147 | 355 | UFH | 353 | LMWH | 3M | 3D-3M |
| Monreal | 1999 | DVT | 10000IU/d | 2.0-3.0 | 72/18 | 59/15 | 92/118 | 122/122 | 220 | LMWH | 244 | Coumarin | 3M | 3-6M |
| Monreal | 1999 | DVT | 10000IU/d | 2.0-3.0 | 60/14 | 59/15 | 103/87 | 122/122 | 190 | LMWH | 244 | Coumarin | 3M | 3-6M |
| Riess | 2003 | DVT | 5000IU followed by 20IU/kg/h | 8000U twice daily | 60.8/15.0 | 60.6/14.7 | 322/261 | 345/282 | 593 | UFH | 627 | Certoparin | 5-14D | 190D |
| Simonneau | 1997 | PE | 50IU/KG followed by 500IU/kg/d | 2.0-3.0 | 67/16 | 67/16 | 139/169 | 133/169 | 308 | UFH | 304 | LMWH | 3M | 90D |
| Kang | 2019 | DVT | 15mg twice daily for 3w<br>20mg for 6M | 1.0mg/kg/2.0-3.0 | 57.3/11.11 | 60.1/10.9 | 15/20 | 13/19 | 35 | Rivaroxaban | 32 | Enoxaparin | 6M | 21-180D |
| Ramacciotti | 2004 | DVT | 1.5mg/kg | 5000IU+500IU/kg/d | 46/19 | 44/18 | 34/70 | 35/62 | 104 | Edoxaparin | 97 | UFH | 5-10D | 3M |
| Bynum | 1979 | PE | 5000U/12h | 2.5-17.5mg/d | 42.6 | 45.9 | 13.0/11 | 10.0/14 | 24 | Heparin | 24 | Warfarin | 6M | 18M |
| Marshall | 2020 | DVT | 20mg/d | - | 68(32-87) | 68(30-87) | 29/17 | 18/28 | 46 | Rivaroxaban | 46 | Placebo | 6M | 12M |
| Merli | 2001 | DVT | 1.0mg/kg twice daily | 2.0-3.0 | 60.7(18-92) | 60.9(18-91) | 181/131 | 150/140 | 312 | Edoxaparin | 290 | UFH | 3M | 3M |
| Merli | 2001 | DVT | 1.5mg/kg | 2.0-3.0 | 60.7(19-91) | 60.9(18-91) | 161/137 | 150/140 | 298 | Edoxaparin | 290 | UFH | 3M | 3M |

| | | | | | | | | | | | | | | |
|---|---|---|---|---|---|---|---|---|---|---|---|---|---|---|
| Gonzalez | 1999 | DVT | 4000IU/d | 5mg/1.5-2.0 | 62.7(19-83) | 58.3(20-82) | 41/44 | 46/34 | 85 | Edoxaparin | 80 | Coumarin | 3M | 3M |
| Wells | 2005 | DVT OR PE | 200IU/kg | 175IU/kg | 58.5/17.2 | 57.1/17.2 | 140/111 | 133/121 | 251 | Dalteparin | 254 | Tinzaparin | 3M | 1W-3M |
| Raskob | 2016 | DVT OR PE WITH CANCER | 60mg/d 30mg/d CC 30-50ml/min | 2.0-3.0 | 66/13 | 67.0/12 | 181/197 | 206/187 | 378 | Edoxaban | 393 | Warfarin | 3-12M | 12M |
| Raskob | 2016 | DVT OR PE WITH ACTIVE CANCER | 60mg/d 30mg/d CC 30-50ml/min | 2.0-3.0 | 66/12 | 65/12 | 54/55 | 60/39 | 109 | Edoxaban | 99 | Warfarin | 3-12M | 12M |
| Raskob | 2016 | DVT OR PE WITH ACTIVE CANCER | 60mg/d 30mg/d CC 30-50ml/min | 2.0-3.0 | 67/10 | 66/12 | 48/37 | 50/27 | 85 | Edoxaban | 77 | Warfarin | 3-12M | 12M |
| Lopaciuk | 1999 | DVT OR PE | 85IU/kg tiwce daily | 85IU/kg | 56.6/16.2 | 57.8/14.6 | 45/53 | 59/46 | 98 | Nadroparin | 95 | Acenocoumar ol | 3M | 12M |
| Simes | 2014 | DVT OR PE | - | 100mg/d | 57/16 | 57/16 | 343/265 | 361/255 | 608 | Placebo | 616 | Aspirin | 6-9M | 2-4Y |
| Meyer | 2002 | DVT OR PE WITH CANCER | 1.5mg/kg | 6-10mg/2.0-3.0 | 65/13 | 66/11 | 28/43 | 37/38 | 71 | Edoxaparin | 75 | Warfarin | 3M | 3M |
| Schrag | 2023 | VTE WITH CANCER | - | 2-3 | 64(56-70) | 62(56-68) | 181/149 | 172/136 | 330 | DOAC | 308 | Fondaparinux | 14D | 6M |
| Agnelli | 2020 | DVT WITH CANCER | 10mg twice daily for 10 day followed 5mg twice daily | 200IU/kg/d FOR 1M 150IU/kg/d | 67.2/11.3 | 67.2/10.9 | 292/284 | 276/303 | 576 | Apixaban | 579 | Dalteparin | 6M | 6M |
| Schulman | 2014 | VTE | 150mg twice daily | 2-3 | 54.7/17.6 | 55.1/16.3 | 781/499 | 776/512 | 1280 | Dabigatran | 1288 | Warfarin | 6M | 6M |
| Nisio | 2019 | VTE WITH ACTIVE CANCER | 60mg/d 30mg/d CC 30-50ml/min | 200IU/kg for 30d followed by 150iu/kg | 62.4/10.8 | 64.2/11.6 | 121/107 | 116/135 | 228 | Edoxaban | 251 | Dalteparin | <6M | 1-12M |
| Nisio | 2019 | VTE WITH ACTIVE CANCER | 60mg/d 30mg/d CC 30-50ml/min | 200IU/kg for 30d followed by 150iu/kg | 64.4/11.1 | 63.3/11.7 | 156/138 | 147/126 | 294 | Edoxaban | 273 | Dalteparin | >6M | 1-12M |
| Becattini | 2011 | DVT OR PE | 100mg/d | - | 61.9/15.3 | 62.1/15.1 | 135/70 | 122/75 | 205 | Aspirin | 197 | Placebo | 2Y | 2Y+6M |
| Breddin | 2001 | DVT | 5000IU followed by 1250iu/h | 7000u 35-45kg 8000u 46-60kg 12600u 60kg twice daily | 58.6/0.83 | 58.6/0.78 | 206/169 | 214/174 | 375 | UFH | 388 | Reviparin | 5-7D | 4-21D |
| Breddin | 2001 | DVT | 5000iu followed by 1250IU/h | 7000u 35-45kg 8000u 46-60kg 12600u 60kg/d | 58.6/0.83 | 58.5/0.80 | 206/169 | 201/173 | 375 | UFH | 374 | Reviparin | 28/2D | 4-21D |

| | | | | | | | | | | | | | | |
|---|---|---|---|---|---|---|---|---|---|---|---|---|---|---|
| Buller | 1997 | DVT OR PE | 6300IU twice daily >60kg 4200IU twice daily 46-60kg 3500IU twice daily | 5000iu followed by 1250iu/h | 59.4/17.2 | 61.6/16.6 | 258/252 | 267/244 | 510 | Reviparin | 511 | UFH | >5D | 14D-12 W |
| Bauersach s | 2018 | DVT OR PE GFR<60ml/min /1.73m3 | 175OIU/kg | 2.0-3.0 | 58(18-89) | 58(18-89) | - | - | 355 | Tinzaparin | 378 | Warfarin | 3M | 6M |
| Bauersach s | 2018 | DVT OR PE GFR>60ml/min /1.73m3 | 175IU/kg | 2.0-3.0 | 65(38-87) | 65(38-87) | - | - | 69 | Tinzaparin | 62 | Warfarin | 3M | 6M |
| Prins | 2018 | VTE | 20mg | - | 59.0/14.5 | 57.2/15.4 | - | - | 598 | Rivaroxaban | 590 | Placebo | 6-12M | 12M |
| Prins | 2018 | VTE | 20mg | - | - | - | - | - | 1107 | Rivaroxaban | 590 | Placebo | 6-12M | 12M |
| Prins | 2018 | VTE | 10mg | - | - | - | - | - | 1127 | Rivaroxaban | 590 | Placebo | 6-12M | 12M |
| Prins | 2018 | VTE | - | 100mg | - | - | - | - | 590 | Placebo | 1131 | Aspirin | 6-12M | 12M |
| Jimenez | 2007 | PE | 1mg/kg twice daily | 1mg/kg/d | 69/16 | 67/16 | 75/106 | 95/104 | 181 | Edoxaparin | 199 | Acenocoumar ol | 6M | 3M |
| Eriksson | 2005 | DVT OR PE | 24mg twice daily | - | 56/15 | 58/15 | 332/281 | 313/298 | 612 | Ximelagatran | 611 | Placebo | 18M | 18M |
| Veiga | 2000 | DVT | 5000IU followed by 1000IU/h | 2.0-3.0 | 80.9 | 79.6 | 17/33 | 24/26 | 50 | Edoxaparin | 50 | Acenocoumar ol | 6M | 3W-6M |
| Nakamura | 2015 | DVT OR PE | 60mg/d 30mg/d CC 30-50ml/min | 2.0-3.0 | 61.1/15.5 | 62.6/15 | 274/289 | 273/264 | 563 | Edoxaban | 538 | Warfarin | 5D | 5-60D |
| Nakamura | 2015 | DVT OR PE | 60mg/d 30mg/d CC 30-50ml/min | 2.0-3.0 | 54.8/16.2 | 54.9/16.1 | 2086/1469 | 2083/1501 | 3555 | Edoxaban | 3584 | Warfarin | 5D | 5-60D |
| Cirujeda | 2006 | DVT | 30mg twice daily | 2mg/d | 67.7 | 66.1 | 45/35 | 47/28 | 75 | Sulodexide | 75 | Acenocoumar ol | 3M | 3M |
| Perez | 2010 | PE | 2.0-3.0 | 175IU/kg | 72.1(24-91) | 72.4(25-93) | 25/25 | 28/24 | 50 | Acenocoumaro l | 52 | Tinzaparin | 6M | 1-6M |
| Wells | 2016 | DVT OR PE | 20mg/d | - | 58.2/15.6 | 58.4/16.0 | 354/248 | 339/255 | 602 | Rivaroxaban | 594 | Placebo | 6-12M | 12M |
| Weitz | 2017 | DVT OR PE | 20mg | 100mg | 57.9/14.7 | 58.8/14.7 | 602/505 | 643/488 | 1107 | Rivaroxaban | 1131 | Aspirin | 6M | 12M |
| Weitz | 2017 | DVT OR PE | 10mg | 100mg | 58.8/14.7 | 58.8/14.7 | 620/507 | 643/488 | 1127 | Rivaroxaban | 1131 | Aspirin | 6M | 12M |
| Buller | 2011 | DVT | 3.0mg | 2.5mg/d | 55.6/17.2 | 58.2/16.3 | 228/158 | 213/158 | 386 | Idrabiotaparinu x | 371 | Idraparinux | 6M | 6M |

| | | | | | | | | | | | | | | |
|---|---|---|---|---|---|---|---|---|---|---|---|---|---|---|
| Lee | 2003 | DVT OR PE | 200IU/kg(<18000IU) for 1M 150IU/kg | 1.5-2.5 | 62/12 | 63/13 | 159/179 | 169/169 | 338 | Dalteparin | 338 | DOAC | 6M | 6M |
| Harenberg | 2000 | DVT | 8000IU twice daily | 5000IU+20IU/kg/h | 61.0/13.6 | 63.0/13.7 | 152/113 | 116/157 | 265 | Certoparin | 273 | UFH | 6M | 6M |
| Schulman | 2013 | DVT OR PE | 150mg twice daily | 2.0-3.0 | 55.4/15.0 | 53.9/15.3 | 871/559 | 869/555 | 1430 | Dabigatran | 1426 | Warfarin | 18M | 6M |
| Schulman | 2013 | DVT OR PE | 150mg twice daily | 2.0-3.0 | 56.1/15.5 | 55.5/15.1 | 381/300 | 364/298 | 681 | Dabigatran | 662 | Warfarin | 18M | 6M |
| Harenberg | 2001 | DVT | 8000IU | 5000IU 20IU/kg/h | 60.6/14.0 | 62.3/13.8 | 216/177 | 227/177 | 393 | Certoparin | 404 | UFH | 12D | 7-15D |
| Weycker | 2018 | DVT OR PE | - | - | 60.0/16.0 | 60.0/16.0 | 9282/8595 | 9279/8599 | 17878 | Apixaban | 17878 | Warfarin | 1M | 6M |
| Wysokinski | 2019 | VTE | 60mg/d 30mg/d CC 30-50ml/min | 5000IU 20IU/kg/h | 64/12 | 62/12 | 117/107 | 209/154 | 224 | Apixaban | 363 | Enoxaparin | 3M | 3M |
| Wysokinski | 2019 | VTE | 60mg/d 30mg/d CC 30-50ml/min | 5000IU 20IU/kg/h | 62/13 | 62/12 | 82/81 | 209/154 | 163 | Rivaroxaban | 363 | Enoxaparin | 3M | 3M |
| Harenberg | 2003 | DVT | 8000IU twice daily | 5000IU 20IU/kg/h | 60.7/14.4 | 61.5/14.6 | 55.8 | 56.4 | 893 | Certoparin | 865 | UFH | 10-14D | 6M |
| Young | 2018 | DVT OR PE | 200IU/kg for 1M 150IU/kg | 15mg twice daily for 3W 20mg/d for 6M | 67（34-87） | 67（22-87） | 98/105 | 116/87 | 203 | Dalteparin | 203 | Rivaroxaban | 6M | 3-24M |
| Hull | 1979 | DVT | 5000u/12h | 10mg/d | - | - | 13/22 | 16/17 | 35 | Heparin | 33 | Warfarin | 6-12W | 12W |
| Beyer | 2017 | VTE | 10mg/d | 2.5mg/d | 61(51-73) | 61(50-70) | 100 | 87 | 236 | Rivaroxaban | 236 | Fondaparinux | 45D | 90D |

**3.12 Baseline data for the prevention of venous thromboembolism**

| study | year | Study Participants | dose_t | dose_c | Age | | sex(m) | | Interventions | | | | Treatment duration | length of follow-up |
|---|---|---|---|---|---|---|---|---|---|---|---|---|---|---|
| | | | | | | | | | T group | | Control group | | | |
| | | | | | T | C | T | C | n | drug | n | drug | | |
| Wang | 2023 | SARS-CoV-2 | 2.5mg twice daily | - | 54（44-64） | 54（44-64） | 311/299 | 303/304 | 610 | Apixaban | 607 | Placebo | 30D | 30D |
| Vadhan | 2020 | Malignancies | 10mg/d | - | 66.0（41.0-87.0） | 65.0（39.0-87.0） | 79/56 | 77/61 | 135 | Rivaroxaban | 138 | Placebo | 180D | 180D |
| Turpie | 2009 | scheduled for total knee arthroplasty | 10mg/d | 30mg twice daily | 64.4/9.7 | 64.7/9.7 | 519/1007 | 541/426 | 1526 | Rivaroxaban | 967 | Enoxaparin | 10-14D | 17D |
| Eriksson | 2010 | EHA | 5mg/d | 40mg/d | 60.2(34-82) | 58.1(22-85) | 68/90 | 80/86 | 158 | YM150 | 166 | Enoxaparin | 5W | 6-9D |
| Eriksson | 2010 | EHA | 10mg/d | 40mg/d | 60.3(29-84) | 58.1(22-85) | 73/88 | 80/86 | 161 | YM150 | 166 | Enoxaparin | 5W | 6-9D |
| Eriksson | 2010 | EHA | 30mg/d | 40mg/d | 57.9(23-85) | 58.1(22-85) | 77/79 | 80/86 | 156 | YM150 | 166 | Enoxaparin | 5W | 6-9D |
| Eriksson | 2010 | EHA | 60mg/d | 40mg/d | 61.3(24-84) | 58.1(22-85) | 73/90 | 80/86 | 163 | YM150 | 166 | Enoxaparin | 5W | 6-9D |
| Eriksson | 2010 | EHA | 120mg/d | 40mg/d | 60.2(26-83) | 58.1(22-85) | 77/79 | 80/86 | 156 | YM150 | 166 | Enoxaparin | 5W | 6-9D |
| Fisher | 2007 | THR OR TKR | 5mg/d | 40mg/d 30mg twice daily | 66(26-86) | 66（27-83） | 88/144 | 101/135 | 232 | Rivaroxaban | 236 | Enoxaparin | 5-9D | 30-60D |
| Fisher | 2007 | THR OR TKR | 10mg/d | 40mg/d 30mg twice daily | 65(31-86) | 66（27-83） | 100/138 | 101/135 | 238 | Rivaroxaban | 236 | Enoxaparin | 5-9D | 30-60D |
| Fisher | 2007 | THR OR TKR | 20mg/d | 40mg/d 30mg twice daily | 66(43-93) | 66（27-83） | 90/146 | 101/135 | 236 | Rivaroxaban | 236 | Enoxaparin | 5-9D | 30-60D |
| Fisher | 2007 | THR OR TKR | 40mg/d | 40mg/d 30mg twice daily | 66(35-92) | 66（27-83） | 82/150 | 101/135 | 232 | Rivaroxaban | 236 | Enoxaparin | 5-9D | 30-60D |
| Fisher | 2007 | THR OR TKR | 60mg/d | 40mg/d 30mg twice daily | 66(39-87 | 66（27-83） | 59/84 | 101/135 | 143 | Rivaroxaban | 236 | Enoxaparin | 5-9D | 30-60D |
| Kolluri | 2016 | CABG surgery | 2.5mg/d | - | 64.4/8.9 | 62.0/8.9 | 30/11 | 27/10 | 41 | Fondaparinux | 37 | Placebo | 9D | 35D |
| Bergqvist | 1996 | EHA | 40mg/d | - | 70（44-87） | 70（44-87） | 56/75 | 57/74 | 131 | Enoxaparin | 131 | Placebo | 19-23D | 21D |
| Kakkar | 2004 | CANCER | 5000IU | - | 65（53.8-68.4） | 60.9（52.4-69.4） | 77/113 | 84/100 | 190 | Dalteparin | 184 | Placebo | 1Y | 1Y |
| Eriksson | 2007 | EHR | 5mg/d | 40mg twice daily | 64(31-86) | 64(30-92) | 29/47 | 74/88 | 76 | Rivaroxaban | 162 | Enoxaparin | 5-9D | 30-60D |
| Eriksson | 2007 | EHR | 10mg/d | 40mg twice daily | 67(42-84) | 64(30-92) | 29/51 | 74/88 | 80 | Rivaroxaban | 162 | Enoxaparin | 5-9D | 30-60D |
| Eriksson | 2007 | EHR | 20mg/d | 40mg twice daily | 65(39-89) | 64(30-92) | 24/44 | 74/88 | 68 | Rivaroxaban | 162 | Enoxaparin | 5-9D | 30-60D |
| Eriksson | 2007 | EHR | 40mg/d | 40mg twice daily | 66(41-84) | 64(30-92) | 38/50 | 74/88 | 88 | Rivaroxaban | 162 | Enoxaparin | 5-9D | 30-60D |

| | | | | | | | | | | | | | | |
|---|---|---|---|---|---|---|---|---|---|---|---|---|---|---|
| Eriksson | 2007 | EHR | 20mg twice daily | 40mg twice daily | 66(32-84) | 64(30-92) | 32/45 | 74/88 | 77 | Rivaroxaban | 162 | Enoxaparin | 5-9D | 30-60D |
| Eriksson | 2007 | EHR | 30mg twice daily | 40mg twice daily | 64(30-87) | 64(30-92) | 34/40 | 74/88 | 74 | Rivaroxaban | 162 | Enoxaparin | 5-9D | 30-60D |
| Fuji | 2008 | THA | 20mg | - | 63.3/10.4 | 62.0/10.3 | 10/71 | 11/75 | 81 | Enoxaparin | 86 | Placebo | 14D | 90D |
| Fuji | 2008 | THA | 40mg | - | 60.6/9.9 | 62.0/10.3 | 6/74 | 11/75 | 80 | Enoxaparin | 86 | Placebo | 14D | 90D |
| Fuji | 2008 | THA | 20mg twice daily | - | 63.0/9.3 | 62.0/10.3 | 15/75 | 11/75 | 90 | Enoxaparin | 86 | Placebo | 14D | 90D |
| Fuji | 2008 | TKA | 20mg | - | 68.8/9.0 | 68.7/9.5 | 15/63 | 15/64 | 78 | Enoxaparin | 79 | Placebo | 14D | 90D |
| Fuji | 2008 | TKA | 40mg | - | 70.0/9.4 | 68.7/9.5 | 11/63 | 15/64 | 74 | Enoxaparin | 79 | Placebo | 14D | 90D |
| Fuji | 2008 | TKA | 20mg twice daily | - | 68.3/8.7 | 68.7/9.5 | 5/79 | 15/64 | 84 | Enoxaparin | 79 | Placebo | 14D | 90D |
| Eriksson | 2003 | THA | 2.5mg | 40mg | 79(17-97) | 79(19-101) | 187/644 | 224/618 | 831 | Fondaparinux | 842 | Enoxaparin | 3w | 1M |
| Eriksson | 2003 | THA | 2.5mg | 40mg | 79(23-94) | 79(28-96) | 92/235 | 98/231 | 327 | Fondaparinux | 329 | Enoxaparin | 3w | 1M |
| Haas | 2012 | CANCER(breas t) | 3000IU/d | - | 54.6/10.3 | 56.6/11.0 | NA | NA | 174 | Certoparin | 178 | Placebo | 6M | 6M |
| Haas | 2012 | CANCER(breas t) | 3000IU/d | - | 60.8/9.5 | 60.3/10.0 | NA | NA | 273 | Certoparin | 273 | Placebo | 6M | 6M |
| Verhamme | 2021 | TKA | 30mg/d | 40mg | 67（49-81） | 67（45-79） | 13/89 | 20/81 | 102 | Abelacimab | 101 | Enoxaparin | 8-12D | 8-12D |
| Verhamme | 2021 | TKA | 75mg | 40mg | 67（41-81） | 67（45-79） | 19/80 | 20/81 | 99 | Abelacimab | 101 | Enoxaparin | 8-12D | 8-12D |
| Verhamme | 2021 | TKA | 150mg | 40mg | 68（49-80） | 67（45-79） | 21/77 | 20/81 | 98 | Abelacimab | 101 | Enoxaparin | 8-12D | 8-12D |
| Selby | 2015 | fractures | 5000IU/d | - | 48.8/16.3 | 48.3/15.5 | 66/68 | 73/58 | 134 | Dalteparin | 131 | Placebo | 14D | 3M |
| Eikelboom | 2016 | Surgery | 200mg 2-4h followed by 100mg for 30 d | - | 68.6/10.3 | 68.6/10.3 | 2597/2401 | 2687/2325 | 4998 | Aspirin | 5012 | Placebo | 30D | 30D |
| Eriksson | 2007 | TKA | 220mg/d | 40mg/d | 67.0/9 | 68.0/9 | 238/441 | 216/478 | 679 | Dabigatran | 694 | Enoxaparin | 6-10D | 3M |
| Eriksson | 2007 | TKA | 150mg/d | 40mg/d | 68.0/9 | 68.0/9 | 262/441 | 216/478 | 703 | Dabigatran | 694 | Enoxaparin | 6-10D | 3M |
| Weitz | 2021 | TKA | 25mg twice daily | 40mg | 69(51-87) | 68（50-90） | 37/92 | 81/171 | 129 | Milvexian | 252 | Enoxaparin | 10-14D | 6W |
| Weitz | 2021 | TKA | 50mg twice daily | 40mg | 68(51-87) | 68（50-90） | 35/89 | 81/171 | 124 | Milvexian | 252 | Enoxaparin | 10-14D | 6W |
| Weitz | 2021 | TKA | 100mg twice daily | 40mg | 67(50-86) | 68（50-90） | 46/88 | 81/171 | 134 | Milvexian | 252 | Enoxaparin | 10-14D | 6W |
| Weitz | 2021 | TKA | 200mg twice daily | 40mg | 69(50-86) | 68（50-90） | 42/89 | 81/171 | 131 | Milvexian | 252 | Enoxaparin | 10-14D | 6W |
| Weitz | 2021 | TKA | 25mg/d | 40mg | 67(55-77) | 68（50-90） | 18/10 | 81/171 | 28 | Milvexian | 252 | Enoxaparin | 10-14D | 6W |
| Weitz | 2021 | TKA | 50mg/d | 40mg | 68(50-88) | 68（50-90） | 35/92 | 81/171 | 127 | Milvexian | 252 | Enoxaparin | 10-14D | 6W |

| | | | | | | | | | | | | | | |
|---|---|---|---|---|---|---|---|---|---|---|---|---|---|---|
| Weitz | 2021 | TKA | 200mg/d | 40mg | 68(50-88） | 68（50-90） | 35/88 | 81/171 | 123 | Milvexian | 252 | Enoxaparin | 10-14D | 6W |
| Becattini | 2022 | CANCER(colorectal) | 10mg/d | - | 65.8/11.3 | 64.5/11.1 | 163/124 | 141/141 | 287 | Rivaroxaban | 282 | Placebo | 3W | 4W |
| Agnelli | 2007 | THR OR TKR | 25mg/d | 40mg | 63.4(44-75) | 63.5(42-75) | 10/22 | 38/52 | 32 | LY517717 | 90 | Enoxaparin | 5-9D | 1M |
| Agnelli | 2007 | THR OR TKR | 50mg | 40mg | 62.4(33-75) | 63.5(42-75) | 15/19 | 38/52 | 34 | LY517717 | 90 | Enoxaparin | 5-9D | 1M |
| Agnelli | 2007 | THR OR TKR | 75mg | 40mg | 64.4(45-74) | 63.5(42-75) | 11/21 | 38/52 | 32 | LY517717 | 90 | Enoxaparin | 5-9D | 1M |
| Agnelli | 2007 | THR OR TKR | 100mg | 40mg | 62.4(31-75) | 63.5(42-75) | 50/56 | 38/52 | 106 | LY517717 | 90 | Enoxaparin | 5-9D | 1M |
| Agnelli | 2007 | THR OR TKR | 125mg | 40mg | 61.6(32-75） | 63.5(42-75) | 57/53 | 38/52 | 110 | LY517717 | 90 | Enoxaparin | 5-9D | 1M |
| Agnelli | 2007 | THR OR TKR | 150mg | 40mg | 62.1(33-75） | 63.5(42-75) | 49/54 | 38/52 | 103 | LY517717 | 90 | Enoxaparin | 5-9D | 1M |
| Carrier | 2019 | CANCER | 2.5mg twice daily | - | 61.2/12.4 | 61.7/11.3 | 121/170 | 119/164 | 291 | Apixaban | 283 | Placebo | 6M | 7M |
| Planes | 1999 | THR | 40mg | 4500IU | 64.0/11 | 65.0/11 | 111/137 | 108/143 | 248 | Enoxaparin | 251 | Tinzaparin | 15D | 12-14D |
| Woller | 2012 | fracture | 1.8-2.5 | 600mg<br>325mg twice daily for 1M | | | | | 129 | Warfarin | 152 | Aspirin | 4-6W | 90D |
| Fuji | 2014 | THA | 15mg/d | 20mg twice daily | 61.3/10.3 | 58.9/10.7 | 15/63 | 15/59 | 78 | Edoxaban | 74 | Enoxaparin | 11-14D | 15D |
| Fuji | 2014 | THA | 30mg | 20mg twice daily | 60.6/9.6 | 58.9/10.7 | 3/69 | 15/59 | 72 | Edoxaban | 74 | Enoxaparin | 11-14D | 15D |
| Eriksson | 2006 | THR | 2.5mg twice daily | 40mg | 66(26-86) | 65(27-82) | 47/85 | 54/78 | 132 | BAY 59-7939 | 132 | Enoxaparin | 5-9D | 5-9D |
| Eriksson | 2006 | THR | 5mg twice daily | 40mg | 64(31-84) | 65(27-82) | 63/73 | 54/78 | 136 | BAY 59-7939 | 132 | Enoxaparin | 5-9D | 5-9D |
| Eriksson | 2006 | THR | 10mg twice daily | 40mg | 65(43-93) | 65(27-82) | 53/80 | 54/78 | 133 | BAY 59-7939 | 132 | Enoxaparin | 5-9D | 5-9D |
| Eriksson | 2006 | THR | 20mg twice daily | 40mg | 65(35-85) | 65(27-82) | 51/83 | 54/78 | 134 | BAY 59-7939 | 132 | Enoxaparin | 5-9D | 5-9D |
| Eriksson | 2006 | THR | 30mg twice daily | 40mg | 67(51-87) | 65(27-82) | 16/21 | 54/78 | 37 | BAY 59-7939 | 132 | Enoxaparin | 5-9D | 5-9D |
| Perry | 2010 | CANCER | 5000IU | - | 57(30-81) | 55(26-77) | 61/38 | 50/37 | 99 | Dalteparin | 87 | Placebo | 2-4W | 6M |
| Colwell | 2005 | TKA | 36mg twice daily | 1.8-3.0 | 66.9/9.4 | 67.1/9.4 | 446/705 | 415/733 | 1151 | Ximelagatran | 1148 | Warfarin | 7-12D | 12D |
| Ramacciotti | 2022 | COVID-19 | 10mg/d | - | 57.8/14.8 | 56.4/15.6 | 97/62 | 94/65 | 159 | Rivaroxaban | 159 | Placebo | 35D | 35D |
| Ageno | 2021 | specific acute medical illnesses | 10mg | - | 56.6 | 57.7 | - | - | 3853 | Rivaroxaban | 3872 | Placebo | 3-10D | 3M |
| Ageno | 2021 | specific acute medical illnesses | 7.5mg | - | 44.2 | 42.9 | - | - | 2154 | Rivaroxaban | 2140 | Placebo | 3-10D | 3M |

| | | | | | | | | | | | | | | |
|---|---|---|---|---|---|---|---|---|---|---|---|---|---|---|
| Levine | 2012 | CANCER | 5mg | - | 57(41-67) | 59(20-82) | 15/17 | 15/15 | 32 | Apixaban | 30 | Placebo | 74D | 12W |
| Levine | 2012 | CANCER | 10mg | - | 60(39-76) | 59(20-82) | 13/17 | 15/15 | 30 | Apixaban | 30 | Placebo | 74D | 12W |
| Levine | 2012 | CANCER | 20mg | - | 64(25-86) | 59(20-82) | 20/13 | 15/15 | 33 | Apixaban | 30 | Placebo | 74D | 12W |
| Carlo | 1999 | CANCER | 300mg/d | 5000IU/d | 63/9.6 | 63/10.2 | 233/185 | 205/219 | 418 | Dermatan sulphate | 424 | Heparin | 7D | 4W |
| Eriksson | 2011 | THA | 220mg/d | 40mg/d | 62.0/12 | 62.0/11 | 469/541 | 501/502 | 1010 | Dabigatran | 1003 | Enoxaparin | 28-35D | 3M |
| Francis | 2003 | TKR | 36mg | 1.8-3.0 | 68.5/9.5 | 67.8/9.6 | 267/492 | 300/459 | 769 | Ximelagatran | 759 | Warfarin | 7-12D | 7-12d |
| Francis | 2003 | TKR | 24mg | 1.8-3.0 | 67.7/9.7 | 67.8/9.6 | 294/465 | 300/459 | 757 | Ximelagatran | 759 | Warfarin | 7-12D | 7-12d |
| Samama | 2020 | surgery | 10mg | 40mg | 41(29-54) | 41(29-54) | 1194/615 | 1149/646 | 1809 | Rivaroxaban | 1795 | Enoxaparin | 15D-3M | 45D-90 D |
| Heit | 1997 | TKR | 25IU/kg twice daily | 2.0-3.0 | 68.1/9.4 | 69.0/9.3 | 64/72 | 115/164 | 136 | Ardeparin | 279 | Warfarin | 5-14D | 5-14D |
| Heit | 1997 | TKR | 35IU/kg twice daily | 2.0-3.0 | 68.6/10 | 69.0/9.3 | 60/81 | 115/164 | 141 | Ardeparin | 279 | Warfarin | 5-14D | 5-14D |
| Heit | 1997 | TKR | 50IU/kg twice daily | 2.0-3.0 | 48.8/9.3 | 69.0/9.3 | 112/165 | 115/164 | 277 | Ardeparin | 279 | Warfarin | 5-14D | 5-14D |
| Eriksson | 2003 | THR OR TKR | 2mg followed by 24mg twice daily | 40mg | 67(24-88) | 67(20-89) | 509/868 | 542/845 | 1377 | Ximelagatran | 1387 | Enoxaparin | 8-14D | 4-6W |
| Colwell | 1995 | surgery | 5000IU/8h | 30mg twice daily | - | - | 91/134 | 107/121 | 225 | Heparin | 228 | Enoxaparin | 4-14D | 3M |
| Lassen | 2012 | KR OR HR OR HF | 40mg/d | 20mg od | 59(20-90) | 60(19-90) | 626/529 | 612/541 | 1155 | Enoxaparin | 1153 | Semuloparin | 7-10D | 7-10D |
| Lassen | 2012 | KR | 30mg twice daily | 20mg od | 64(22-88) | 65(28-88) | 408/160 | 402/171 | 568 | Enoxaparin | 573 | Semuloparin | 7-10D | 7-10D |
| Lassen | 2010 | THR | 2.5mg twice daily | 40mg | 60.9(19-92) | 60.6(19-93) | 1278/1430 | 1248/1451 | 2708 | Apixaban | 2699 | Enoxaparin | 32-38D | 95D |
| Samama | 2002 | THR | 4200IU/d | 2.0-3.0 | 66.0/11 | 65.0/12 | 316/328 | 322/323 | 644 | Reviparin | 645 | Acenocoum arol | 2-3D | 6-9W |
| Menzin | 1994 | HR | 30mg twice daily | 5000IU/8h | 65.8/11.0 | 65.7/10.7 | 97/95 | 101/108 | 192 | Enoxaparin | 209 | UFH | 7D | 7-14D |
| Menzin | 1994 | HR | 40mg/d | 5000IU/8h | 65.0/11.3 | 65.7/10.7 | 99/103 | 101/108 | 202 | Enoxaparin | 209 | UFH | 7D | 7-14D |
| Wirth | 2001 | EKA | 1750IU | - | 37.6/13.0 | 38.5/11.6 | 81/36 | 98/24 | 117 | Reviparin | 122 | Placebo | 7-10D | 7-10D |
| Burrows | 2001 | pregnancy | 2500IU | - | 31.7/4.8 | 31.3/5.5 | | | 39 | Dalteparin | 37 | Placebo | 5D | 2-6W |
| Leizorovic z | 2004 | acute medical condition | 5000IU | - | 68.5/11.1 | 68.5/11.7 | 884/964 | 888/945 | 1848 | Dalteparin | 1833 | Placebo | 14D | 90D |
| Kleber | 2003 | respiratory disease or heart failure | 40mg/d | 5000IU third times | 70/14 | 70/14 | 160/172 | 183/150 | 332 | Enoxaparin | 333 | UFH | 8-12D | 9-13D |

| | | | | | | | | | | | | | | |
|---|---|---|---|---|---|---|---|---|---|---|---|---|---|---|
| Heit | 2000 | THR OR TKR | 100IU/kg(<10000IU) | - | 65.0/11.3 | 66.0/11 | 265/342 | 275/313 | 607 | Ardeparin | 588 | Placebo | 6W | 12W |
| Cho | 2013 | TKA | 2.5mg | - | 68.5/6.0 | 68.5/5.5 | 5/69 | 7/67 | 74 | Fondaparinux | 74 | Placebo | 5D | 90D |
| Ginsberg | 2009 | TKA | 220mg/d | 30mg twice daily | 66.2/9.5 | 66.3/9.6 | 371/486 | 364/504 | 857 | Dabigatran | 868 | Enoxaparin | 12-15D | 3M |
| Ginsberg | 2009 | TKA | 150mg/d | 30mg twice daily | 65./9.5 | 66.3/9.6 | 364/507 | 364/504 | 871 | Dabigatran | 868 | Enoxaparin | 12-15D | 3M |
| Song | 2018 | esophageal carcinoma | 2850IU | 2.5mg | 63.1/8.7 | 63/6.5 | 30/27 | 33/26 | 57 | Nadroparin | 59 | Fondaparinux | 7D | 7D |
| Fuji | 2008 | TKR | 0.75mg | - | 71.4/8.7 | 70.4/7.9 | 15/71 | 15/72 | 86 | Fondaparinux | 87 | Placebo | 10-14D | 11-17D |
| Fuji | 2008 | TKR | 1.5mg | - | 70.5/8.0 | 70.4/7.9 | 18/67 | 15/72 | 85 | Fondaparinux | 87 | Placebo | 10-14D | 11-17D |
| Fuji | 2008 | TKR | 2.5mg | - | 71.2/7.8 | 70.4/7.9 | 17/67 | 15/72 | 84 | Fondaparinux | 87 | Placebo | 10-14D | 11-17D |
| Fuji | 2008 | TKR | 3.0mg | - | 71.5/7.6 | 70.4/7.9 | 10/74 | 15/72 | 84 | Fondaparinux | 87 | Placebo | 10-14D | 11-17D |
| Fuji | 2008 | THR | 0.75mg | - | 60.8/9.8 | 62.3/12.4 | 11/69 | 18/64 | 80 | Fondaparinux | 82 | Placebo | 10-14D | 11-17D |
| Fuji | 2008 | THR | 1.5mg | - | 60.9/10.1 | 62.3/12.4 | 20/60 | 18/64 | 80 | Fondaparinux | 82 | Placebo | 10-14D | 11-17D |
| Fuji | 2008 | THR | 2.5mg | - | 61.5/10.8 | 62.3/12.4 | 7/74 | 18/64 | 81 | Fondaparinux | 82 | Placebo | 10-14D | 11-17D |
| Fuji | 2008 | THR | 3.0mg | - | 62.7/11.4 | 62.3/12.4 | 17/66 | 18/64 | 83 | Fondaparinux | 82 | Placebo | 10-14D | 11-17D |
| Eriksson | 2007 | THR | 220mg | 40mg/d | 65.0/10 | 64.0/11 | 506/636 | 503/651 | 1146 | Dabigatran | 1154 | Enoxaparin | 28-35D | 29-36D |
| Eriksson | 2007 | THR | 150mg | 40mg/d | 36.0/11 | 64.0/11 | 496/667 | 503/651 | 1163 | Dabigatran | 1154 | Enoxaparin | 28-35D | 29-36D |
| Eriksson | 2005 | THR OR TKR | 50mg twice daily | 40mg/d | 66.1(31-88) | 65.0(20-86) | 166/223 | 151/241 | 389 | Dabigatran | 392 | Enoxaparin | 6-10D | 6-10D |
| Eriksson | 2005 | THR OR TKR | 150mg twice daily | 40mg/d | 65.9(34-89) | 65.0(20-86) | 138/252 | 151/241 | 390 | Dabigatran | 392 | Enoxaparin | 6-10D | 6-10D |
| Eriksson | 2005 | THR OR TKR | 300mg/d | 40mg/d | 66.5(21-88) | 65.0(20-86) | 139/246 | 151/241 | 385 | Dabigatran | 392 | Enoxaparin | 6-10D | 6-10D |
| Eriksson | 2005 | THR OR TKR | 225mg twice daily | 40mg/d | 65.9(33-93) | 65.0(20-86) | 164/229 | 151/241 | 393 | Dabigatran | 392 | Enoxaparin | 6-10D | 6-10D |
| Schellong | 2010 | acute medical condition | 3000IU/d | 7500IU twice daily | 70.2/12.2 | 71.0/12.4 | 77/86 | 83/91 | 163 | Certoparin | 174 | UFH | 8-12D | 83-97D |
| Eriksson | 2008 | Surgery | 300mg | - | 74(26-94) | 73(19-95) | 39/90 | 30/102 | 129 | TTP889 | 132 | Placebo | 3W | 7W |
| Eriksson | 2004 | fracture | 2.5mg | - | - | - | - | - | 208 | Fondaparinux | 220 | Placebo | 7D | 25-32D |
| Weitz | 2019 | EKA | 0.3mg/kg | 2.5mg | 66/7.5 | 65/7.0 | 17/21 | 21/42 | 38 | JNJ_9375 | 63 | Apixaban | 10-14D | 18W |
| Weitz | 2019 | EKA | 0.6mg/kg | 2.5mg | 66/8.1 | 65/7.0 | 15/25 | 21/42 | 40 | JNJ_9375 | 63 | Apixaban | 10-14D | 18W |
| Weitz | 2019 | EKA | 1.2mg/kg | 2.5mg | 67/6.4 | 65/7.0 | 7/35 | 21/42 | 42 | JNJ_9375 | 63 | Apixaban | 10-14D | 18W |

| | | | | | | | | | | | | | | |
|---|---|---|---|---|---|---|---|---|---|---|---|---|---|---|
| Weitz | 2019 | EKA | 1.8mg/kg | 2.5mg | 67/8.8 | 65/7.0 | 23/99 | 21/42 | 122 | JNJ_9375 | 63 | Apixaban | 10-14D | 18W |
| Abbas | 2016 | ICU | 3500IU/d | 40mg/d | 72.5/6.5 | 70.9/5.4 | 30/20 | 32/18 | 50 | Bemiparin | 50 | Enoxaparin | 2W | 2M |
| Spyropoulos | 2018 | HOSPITALIZED FOR 3-10D | 10mg-CC>50ml 7.5mg-CC 30-50mlcc | - | 69.7 | 69.7 | 3130/2877 | 3154/2858 | 6007 | Rivaroxaban | 6012 | Placebo | 45D | 75D |
| Agnelli | 2009 | CANCER | 3800IU | - | 62.1/10.3 | 63.7/9.2 | 372/397 | 183/198 | 769 | Nadroparin | 381 | Placebo | 110-130D | 120-140 D |
| Marlovits | 2007 | surgery | 40mg | - | 29.9/7.4 | 30.2/6.9 | 55/32 | 53/35 | 87 | Enoxaparin | 88 | Placebo | 20D | 23-28D |
| Oliveira | 2022 | gynecological cancer surgery | 40mg | 10mg | 56(20-82) | 54(23-87) | - | - | 114 | Enoxaparin | 114 | Rivaroxaban | 1M | 2M |
| Lechler | 1996 | high risk for VTE | 40mg | 5000IU third times | 74/13 | 74/13 | 183/294 | 178/304 | 477 | Enoxaparin | 482 | Heparin | 10-17D | 6W |
| Bruntink | 2017 | fracture at least 4w | 2850IU/d | - | 47.7/16.4 | 44.5/17.2 | 39/53 | 38/56 | 92 | Nadroparin | 94 | Placebo | 40D | 40D |
| Bruntink | 2017 | fracture at least 4w | 2.5mg/d | - | 49.7/17.3 | 44.5/17.2 | 41/51 | 38/56 | 92 | Fondaparinux | 94 | Placebo | 40D | 40D |
| Samama | 1999 | inpatient | 20mg | - | 72.9/10.1 | 74.1/10.6 | 187/177 | 192/179 | 364 | Enoxaparin | 371 | Placebo | 6-14D | 83-110D |
| Samama | 1999 | inpatient | 40mg | - | 73.1/10.8 | 74.1/10.6 | 171/196 | 192/179 | 367 | Enoxaparin | 371 | Placebo | 6-14D | 83-110D |
| Agnelli | 2009 | THR surgery for hip fracture | 3 mg followed by 24mg twice daily subcutaneously 4–8 hours after the end of surgery and twice daily for up to 2 days postoperatively, followed by 24 mg | 40mg | 67.4(24-89) | 63.9(21-89) | 229/250 | 211/268 | 479 | Ximelagatran | 479 | Enoxaparin | 32-38D | 180D |
| Zhao | 2023 | pulmonary nodular seg_x005f mentectomy | 10mg | 38u/kg for 3d increase 57u/kg | 61.7/10.1 | 60.7/9.1 | 107/93 | 96/107 | 200 | Rivaroxaban | 203 | Nadroparin | 30D | 30D |
| Kakkar | 1993 | surgery | 2500IU | 5000IU third times | - | - | 649/1245 | 665/1250 | 1894 | LMWH | 1915 | Heparin | 10D | 4-8W |
| Michot | 2002 | arthroscopic knee surgery | 2500IU followed 2500IU<70kg 5000iu>70kg-30d | - | 42.0/14.7 | 46.5/13.2 | 40/26 | 46/18 | 66 | Dalteparin | 64 | Placebo | 1M | 1M |
| Shorr | 2012 | THR 1/2CKD | 15mg tiwce daily | 40mg | 60(18-82) | 59(27-79） | 311/42 | 318/35 | 353 | Enoxaparin | 353 | Desirudin | 8-12D | 8-12D |
| Shorr | 2012 | CKD3A | 15mg tiwce daily | 40mg | 66(36-83) | 66(45-86） | 85/284 | 112/283 | 369 | Enoxaparin | 395 | Desirudin | 8-12D | 8-12D |

| | | | | | | | | | | | | | | |
|---|---|---|---|---|---|---|---|---|---|---|---|---|---|---|
| Shorr | 2012 | CKDB | 15mg tiwce daily | 40mg | 74(47-87) | 74(57-90) | 15/283 | 17/262 | 298 | Enoxaparin | 279 | Desirudin | 8-12D | 8-12D |
| Fitzgerald | 2001 | TKA | 7.5mg/2.0-3.0 | 30mg | - | - | - | - | 176 | Warfarin | 173 | Enoxaparin | 2-5D | 2-5D |
| Francis | 2002 | TKA | 24mg | 5mg-1.8-3.0 | 67.8/10.1 | 67.7/10.4 | 218/127 | 212/118 | 345 | Ximelagatran | 330 | Warfarin | 3-4D | 6W |
| Raskob | 2010 | KA OR HA | 2.5mg twice daily | 40mg | 65.6/9.9 | 65.9/9.8 | 439/1089 | 402/1127 | 1528 | Apixaban | 1529 | Enoxaparin | 10-14D | 30D |
| Raskob | 2010 | KA OR HA | 2.5mg twice daily | 40mg | 60.9/11.8 | 60.6/11.8 | 1288/1420 | 1248/1451 | 2708 | Apixaban | 2699 | Enoxaparin | 32-38D | 60D |
| Veen | 2021 | TKA | 150mg or 220mg/d | 10mg | 66/7.9 | 65/7.5 | 19/26 | 19/30 | 45 | Dabigatran | 49 | Rivaroxaban | 42D | 42D |
| Veen | 2021 | TKA | 2850IU | 10mg | 67.0/10 | 65/7.5 | 14/31 | 19/30 | 45 | Nadroparin | 49 | Rivaroxaban | 42D | 42D |
| Haas | 2006 | THR OR TKR | 4200IU | 7500IU | 66.1/9.3 | 66.9/9.8 | 337/676 | 350/655 | 1013 | Reviparin | 1005 | UFH | 11-14D | 1-14D |
| Wang | 2022 | surgery | 10mg | | 53.87/7.66 | 54.10/6.46 | 18/29 | 21/26 | 47 | Rivaroxaban | 47 | Placebo | 7-10D | 1M |
| Colwell | 1999 | THA | 30mg twice daily | 7.5mg-2.0-3.0 | 63.9/13.17 | 64.1/13.21 | 678/838 | 659/836 | 1516 | Enoxaparin | 1495 | Warfarin | 7D | 3M |
| Prandoni | 2002 | TKA | 2.0-3.0 | - | 68(48-82) | 69(44-87) | 83/101 | 79/97 | 184 | Warfarin | 176 | Placebo | 3M | 1-4W |
| Adrichem | 2017 | surgery | 2850IU/2500IU | - | 48.1/12.8 | 49.1/12.3 | 414/317 | 396/324 | 731 | LMWH | 720 | Placebo | 8D | 3M |
| Adrichem | 2017 | surgery | 2850IU/2500IU | - | 46.5/16.5 | 45.6/16.4 | 347/372 | 369/347 | 719 | LMWH | 716 | Placebo | 8D | 3M |
| Cohen | 2006 | acutely ill patients with congestive heart failure class III/IV | 2.5mg | - | 75.0/8.3 | 74.4/8.3 | 174/255 | 186/234 | 429 | Fondaparinux | 420 | Placebo | 6-14D | 15D |
| Lassen | 2007 | TKR | 2.5mg twice daily | 1.8-3.0 | 67.6(46-88) | 66.8(43-85) | 104/49 | 90/63 | 153 | Apixaban | 153 | Warfarin | 10-14D | 20-28D |
| Lassen | 2007 | TKR | 5mg/d | 1.8-3.0 | 66.9(31-87) | 66.8(43-85) | 102/55 | 90/63 | 157 | Apixaban | 153 | Warfarin | 10-14D | 20-28D |
| Lassen | 2007 | TKR | 5mg twice daily | 1.8-3.0 | 66.4(46-84) | 66.8(43-85) | 103/50 | 90/63 | 157 | Apixaban | 153 | Warfarin | 10-14D | 20-28D |
| Lassen | 2007 | TKR | 10mg/d | 1.8-3.0 | 67.2(28-86) | 66.8(43-85) | 92/64 | 90/63 | 156 | Apixaban | 153 | Warfarin | 10-14D | 20-28D |
| Lassen | 2007 | TKR | 10mg twice daily | 1.8-3.0 | 66.4(37-87) | 66.8(43-85) | 95/59 | 90/63 | 154 | Apixaban | 153 | Warfarin | 10-14D | 20-28D |
| Lassen | 2007 | TKR | 20mg/d | 1.8-3.0 | 65.8(35-90) | 66.8(43-85) | 101/55 | 90/63 | 156 | Apixaban | 153 | Warfarin | 10-14D | 20-28D |
| Lassen | 2007 | TKR | 30mg twice daily | 1.8-3.0 | 66.8(43-85) | 66.8(43-85) | 94/58 | 90/63 | 152 | Apixaban | 153 | Warfarin | 10-14D | 20-28D |
| Mahe | 2005 | inpatient | 7500IU/d | - | 76.1 | 76.5 | 421/188 | 39/1205 | 1230 | Nadroparin | 1244 | Placebo | 21D | 7-21D |
| Ishi | 2013 | high risk for DVT/PE | 5000IU twice daily | 40mg/d | 50.9/21.2 | 57.9/18.7 | 29/16 | 23/18 | 45 | UFH | 41 | LMWH | 4D | 4D |

| | | | | | | | | | | | | | | |
|---|---|---|---|---|---|---|---|---|---|---|---|---|---|---|
| Verhamme | 2013 | THR | 25mg | 10mg/d | 61(24-88) | 62(23-85) | 90/117 | 95/112 | 207 | TB402 | 207 | Rivaroxaban | 35D | 70-90D |
| Verhamme | 2013 | THR | 50mg | 10mg/d | 62(20-85) | 62(23-85) | 91/117 | 95/112 | 208 | TB402 | 207 | Rivaroxaban | 35D | 70-90D |
| Riess | 2010 | acute medical illness | 3000IU | 5000IU third times | 79.0/6.2 | 78.7/6.6 | 669/955 | 655/960 | 1624 | Certoparin | 1615 | UFH | 8-20D | 1M |
| Turpie | 2005 | TKR | 2.5mg twice daily | 30mg twice daily | 66(48-84) | 66(47-83) | 41/59 | 47/57 | 100 | BAY 59-7939 | 104 | Enoxaparin | 5-9D | 5-9D |
| Turpie | 2005 | TKR | 5mg twice daily | 30mg twice daily | 66(45-86) | 66(47-83) | 37/65 | 47/57 | 102 | BAY 59-7939 | 104 | Enoxaparin | 5-9D | 5-9D |
| Turpie | 2005 | TKR | 10mg twice daily | 30mg twice daily | 67(49-84) | 66(47-83) | 37/66 | 47/57 | 103 | BAY 59-7939 | 104 | Enoxaparin | 5-9D | 5-9D |
| Turpie | 2005 | TKR | 20mg twice daily | 30mg twice daily | 68(45-92) | 66(47-83) | 31/67 | 47/57 | 98 | BAY 59-7939 | 104 | Enoxaparin | 5-9D | 5-9D |
| Turpie | 2005 | TKR | 30mg twice daily | 30mg twice daily | 66(39-86) | 66(47-83) | 43/63 | 47/57 | 106 | BAY 59-7939 | 104 | Enoxaparin | 5-9D | 5-9D |
| Turpie | 2009 | THR | 1.5mg/d | - | 60.9/10.1 | 62.3/12.4 | 20/60 | 18/64 | 80 | Fondaparinux | 82 | Placebo | 5-9D | 5-9D |
| Turpie | 2009 | THR | 1.5mg/d | 30mg twice daily | 67(37-91) | 66(26-86) | 100/88 | 137/123 | 188 | Fondaparinux | 260 | Enoxaparin | 5-9D | 5-9D |
| McLeod | 2001 | surgery | 5000IU/8h | 40mg/d | 50/17 | 52/18 | 355/320 | 376/298 | 675 | Heparin | 674 | Enoxaparin | 10D | 9D |
| Eriksson | 2008 | THA | 10mg/d | 40mg/d | 63.1(18-91) | 63.3(18-93) | 220/1000 | 982/1242 | 1220 | Rivaroxaban | 2224 | Enoxaparin | 30-42D | 30-42D |
| Leclerc | 1996 | KA | 2.0-3.0 | 30mg twice daily | 69.2/9.2 | 68.0/9.4 | 123/211 | 124/212 | 334 | Warfarin | 336 | Enoxaparin | 14D | 14D |
| Zhang | 2021 | New Cancer | 2.5mg twice daily | - | 60.5/12.8 | 61.3/11.6 | 101/138 | 105/135 | 239 | Apixaban | 240 | Placebo | 7M | 6M |
| Zhang | 2021 | Recurrent Cancer | 2.5mg twice daily | - | 63.9/9.9 | 64.3/9.4 | 20/32 | 14/29 | 52 | Apixaban | 43 | Placebo | 7M | 6M |
| Dobesh | 2003 | fracture surgery | 2.5mg/d | - | 79(23-94) | 79(28-96) | 92/235 | 98/231 | 327 | Fondaparinux | 329 | Placebo | 19-23D | 19-24D |
| Hull | 2000 | THA | 2500IU+5000IU/d | 10mg or 5mg(>70y or <57kg) (2.0-3.0) | 64.0/12 | 63/13 | 248/248 | 242/247 | 496 | Dalteparin | 489 | Warfarin | 5.7D | 5.7D |
| Hull | 2000 | THA | 2500IU+5000IU/d | 10mg or 5mg(>70y or <57kg) (2.0-3.0) | 63.0/13 | 63/13 | 219/268 | 242/247 | 487 | Dalteparin | 489 | Warfarin | 5.7D | 5.7D |
| Colwell | 2003 | THR | 24mg | 30mg | 64.5/12.8 | 64.0/13.1 | 372/410 | 375/398 | 782 | Ximelagatran | 775 | Enoxaparin | 7-12D | 4-8W |
| Haas | 2011 | cancer | 3000IU | 5000IU third times | 79.7/6.4 | 78.3/5.8 | 83/50 | 84/57 | 133 | Certoparin | 141 | UFH | 8-20D | 8-20D |
| Goldhaber | 2011 | Inpatients | 2.5mg twice daily | 40mg | 66.8/12.0 | 66.7/12.0 | 1626/1629 | 1577/1696 | 3255 | Apixaban | 3273 | Enoxaparin | 6-30D | 30-90D |

| | | | | | | | | | | | | | | |
|---|---|---|---|---|---|---|---|---|---|---|---|---|---|---|
| Comp | 1998 | THR | 750IU followed by 750IU*2/d | 10mg-1.8-2.8 | 67(37-91) | 66(23-88) | 90/109 | 102/95 | 199 | Danaparoid | 197 | Warfarin | 1-10D | 1-3M |
| Hull | 2010 | Acutely ill medical patients | 40mg/d | - | 67.8/12.2 | 67.2/12.4 | 1081/1078 | 1076/1100 | 2159 | Enoxaparin | 2176 | Placebo | 6-14D | 3-6M |
| Hull | 2010 | Acutely ill medical patients | 40mg/d | - | 68.1/12.0 | 68.2/12.7 | 386/430 | 401/411 | 816 | Enoxaparin | 812 | Placebo | 6-14D | 3-6M |
| Planes | 1997 | THR | 40mg | - | - | - | - | - | 90 | Enoxaparin | 89 | Placebo | 21D | 21D |
| Lassen | 2010 | TKR | 2.5mg twice daily | 40mg | 67(59-73) | 67(60-73) | 429/1089 | 402/1127 | 1528 | Apixaban | 1529 | Enoxaparin | 10-14D | 12-16D |
| Borstad | 1992 | surgery | 5000IU twice daily | 2500IU | 57.1/12.7 | 56.3/10.4 | | | 70 | UFH | 71 | LMWH | 7D | 1M |
| Samama | 2014 | High risk of VTE | 2.5mg<br>1.5mg<br>CC30-50ml/min | 2850IU | 46.1/16.0 | 46.5/15.7 | 293/328 | 286/336 | 621 | Fondaparinux | 622 | Nadroparin | 21-45D | 25-29D |
| Eriksson | 2007 | HR | 3mg/d | 40mg | - | - | - | - | 35 | YM150 | 36 | Enoxaparin | 7-8D | 7-10D |
| Eriksson | 2007 | HR | 10mg/d | 40mg | - | - | - | - | 35 | YM150 | 36 | Enoxaparin | 7-8D | 7-10D |
| Eriksson | 2007 | HR | 30mg/d | 40mg | - | - | - | - | 36 | YM150 | 36 | Enoxaparin | 7-8D | 7-10D |
| Eriksson | 2007 | HR | 60mg/d | 40mg | - | - | - | - | 36 | YM150 | 36 | Enoxaparin | 7-8D | 7-10D |
| Lassen | 2008 | TKA | 10mg | 30mg twice daily | 64.4/9.7 | 64.7/9.7 | 519/1007 | 541/967 | 1526 | Rivaroxaban | 1508 | Enoxaparin | 11-15D | 30-35D |
| Piazza | 2023 | COVID-19+VTE risk factor | 10mg | - | 56.3/13.1 | 55.7/13.3 | 242/399 | 259/384 | 641 | Rivaroxaban | 643 | Placebo | 35D | 1-49D |
| Bueller | 2015 | TKA | 200mg | 40mg | 63.0/9 | 64.0/9 | 26/118 | 12/60 | 144 | FXI-ASO | 72 | Enoxaparin | 39D | 36-39D |
| Bueller | 2015 | TKA | 300mg | 40mg | 63.0/8 | 64.0/9 | 17/60 | 12/60 | 77 | FXI-ASO | 72 | Enoxaparin | 39D | 36-39D |
| Bergqvist | 2002 | surgery | - | 40mg | 65(30-87) | 66(40-90) | 104/63 | 96/69 | 167 | Placebo | 165 | Enoxaparin | 25-31D | 70-100D |
| Long | 2014 | fractures | 10mg/d | 0.2-0.4ml | 60.2/20.3 | 63.6/19.1 | 309/299 | 336/381 | 608 | Rivaroxaban | 717 | Nadroparin | 35D | 3M |
| Weitz | 2020 | TKA | 40mg | 2.5 twice daily | 67.0/8.8 | 64.9/8.4 | 21/55 | 18/65 | 76 | Enoxaparin | 83 | Apixaban | 10-13D | 150D |
| Anderson | 2018 | KA OR HA | 10mg | 81mg | 62.7/10.1 | 62.9/10.1 | 833/884 | 804/903 | 1717 | Rivaroxaban | 1707 | Aspirin | 30D | 90D |
| Fuji | 2014 | surgery | 30mg | 2000IU twice daily | 76.5/11.0 | 75.6/12.0 | 11/48 | 7/22 | 59 | Edoxaban | 29 | Enoxaparin | 11-14D | 25-35D |
| Geerts | 1996 | fracture | 5000IU twice daily | 30mg twice daily | 37.0/16.5 | 39.1/16.8 | 99/37 | 93/36 | 136 | Heparin | 129 | Enoxaparin | 10-14D | 14D |
| Eriksson | 2009 | EHR OR EKR | 10mg/d | 40mg/d | 63.8(18-93) | 64.0(18-93) | 1913/2744 | 1978/2714 | 4657 | Rivaroxaban | 4692 | Enoxaparin | 30-35D | 30-42D |
| Abbas | 2017 | at high risk for VTE | 3500IU | 40mg | 72.5/6.5 | 70.9/5.4 | 30/20 | 32/18 | 50 | Bemiparin | 50 | Enoxaparin | 60D | 60D |

| | | | | | | | | | | | | | | |
|---|---|---|---|---|---|---|---|---|---|---|---|---|---|---|
| Pai | 2018 | ICU | 5000IU | 5000IU twice daily | - | - | - | - | 60 | Dalteparin | 58 | UFH | 4-12D | 4-12D |
| Pai | 2018 | ICU | 5000IU | 5000IU twice daily | - | - | - | - | 1805 | Dalteparin | 1804 | UFH | 4-12D | 4-12D |
| Turpie | 2001 | EHR | 0.75mg | 30mg twice daily | 66(18-89) | 66(28-89) | 82/102 | 123/137 | 184 | Org31540/SR 90107A | 260 | Enoxaparin | 5-10D | 10-15D |
| Turpie | 2001 | EHR | 1.5mg | 30mg twice daily | 67(37-91) | 66(28-89) | 88/100 | 123/137 | 188 | Org31540/SR 90107A | 260 | Enoxaparin | 5-10D | 10-15D |
| Turpie | 2001 | EHR | 3.0mg | 30mg twice daily | 66(32-85) | 66(28-89) | 80/97 | 123/137 | 177 | Org31540/SR 90107A | 260 | Enoxaparin | 5-10D | 10-15D |
| Turpie | 2001 | EHR | 6.0mg | 30mg twice daily | 67(41-84) | 66(28-89) | 35/37 | 123/137 | 72 | Org31540/SR 90107A | 260 | Enoxaparin | 5-10D | 10-15D |
| Turpie | 2001 | EHR | 8.0mg | 30mg twice daily | 72(32-92) | 66(28-89) | 25/27 | 123/137 | 52 | Org31540/SR 90107A | 260 | Enoxaparin | 5-10D | 10-15D |
| Turpie | 2009 | TKR | 15mg tiwce daily | 30mg twice daily | 63(47-75) | 62(43-75) | 32/55 | 21/22 | 87 | Betrixaban | 43 | Enoxaparin | 10-14D | 4-8W |
| Turpie | 2009 | TKR | 40mg twice daily | 30mg twice daily | 65(47-75) | 62(43-75) | 32/52 | 21/22 | 84 | Betrixaban | 43 | Enoxaparin | 10-14D | 4-8W |
| Levine | 1991 | EHR | 30mg | 7500IU twice daily | 66.2/10.39 | 66.8/9.09 | 145/188 | 160/172 | 333 | LMWH | 332 | Heparin | 14D | 5-13D |
| Brandt | 2022 | cancer and a CVC | 2.5mg twice daily | - | 59.4/11.7 | 58.3/11.4 | 44/82 | 35/56 | 126 | Apixaban | 91 | Placebo | 180D | 180D |
| Yi | 2014 | ischemic stroke | 4000IU twice daily | 200mg | 70.2/10.51 | 69.3/10.22 | 375/308 | 374/309 | 683 | LMWH | 685 | Aspirin | 10D | 1-10D |
| Agnelli | 2005 | High risk of VTE | 2.5mg | 5000/2500IU | 66(31-92) | 65(17-93) | 788/645 | 796/629 | 1433 | Fondaparinux | 1425 | Dalteparin | 5-9D | 28-32D |
| Eriksson | 2006 | THR | 5mg | 40mg | 64.8(28-84) | 65.6(30-89) | 56/72 | 56/101 | 128 | Rivaroxaban | 157 | Enoxaparin | 5-9D | 1-2M |
| Eriksson | 2006 | THR | 10mg | 40mg | 64.0(27-89) | 65.6(30-89) | 53/89 | 56/101 | 142 | Rivaroxaban | 157 | Enoxaparin | 5-9D | 1-2M |
| Eriksson | 2006 | THR | 20mg | 40mg | 65.0(27-93) | 65.6(30-89) | 57/82 | 56/101 | 139 | Rivaroxaban | 157 | Enoxaparin | 5-9D | 1-2M |
| Eriksson | 2006 | THR | 30mg | 40mg | 65.4(31-86) | 65.6(30-89) | 69/73 | 56/101 | 142 | Rivaroxaban | 157 | Enoxaparin | 5-9D | 1-2M |
| Eriksson | 2006 | THR | 40mg | 40mg | 64.7(27-83) | 65.6(30-89) | 56/81 | 56/101 | 137 | Rivaroxaban | 157 | Enoxaparin | 5-9D | 1-2M |
| Connors | 2021 | SARS-CoV-2 infect | 81mg | - | 54.0(46.0-59.0) | 54.0(45.0-59.0) | 69/95 | 68/96 | 164 | Aspirin | 164 | Placebo | 45D | 75D |
| Connors | 2021 | SARS-CoV-2 infect | 2.5mg twice daily | - | 55.0(46.0-61.0) | 54.0(45.0-59.0) | 70/95 | 68/96 | 165 | Apixaban | 164 | Placebo | 45D | 75D |
| Connors | 2021 | SARS-CoV-2 infect | 5mg twice daily | - | 52.0(47.0-58.0) | 54.0(45.0-59.0) | 62/102 | 68/96 | 164 | Apixaban | 164 | Placebo | 45D | 75D |
| Planes | 1998 | THR | 40mg | 4200IU | 66.0/9 | 66.0/10 | 46/205 | 50/197 | 251 | Enoxaparin | 247 | Reviparin | 10-14D | 10-14D |
| Palumbo | 2011 | myeloma | 100mg | 40mg | 61(55-66) | 62(55-66) | 117/103 | 130/89 | 220 | Aspirin | 219 | Enoxaparin | 1-21D | 3W |
| Palumbo | 2011 | myeloma | 1.25mg | 40mg | 60(54-66) | 62(55-66) | 115/105 | 130/89 | 220 | Warfarin | 219 | Enoxaparin | 1-212D | 3W |

| | | | | | | | | | | | | | | |
|---|---|---|---|---|---|---|---|---|---|---|---|---|---|---|
| Campores e | 2016 | arthroscopy-assi sted knee surgery | 10mg | - | 44.9/12.8 | 45.9/13.9 | 78/44 | 84/35 | 122 | Rivaroxaban | 119 | Placebo | 7-8D | 3M |
| De | 2010 | critically ill patients above 40 years old | 5000IU | 40mg | 61.58(42-87) | 58.37(43-84) | 60/15 | 57/24 | 75 | Heparin | 81 | Enoxaparin | 6D | 6M |

## Appendix 4: Risk of bias assessments

### 4.1 Treatment

| study | D1 | D2 | D3 | D4 | D5 |
|---|---|---|---|---|---|
| Raskob2016 | low risk | low risk | low risk | low risk | low risk |
| Schulman2009 | low risk | low risk | low risk | low risk | low risk |
| Raskob2018 | low risk | low risk | low risk | low risk | low risk |
| Agnelli2013 | low risk | low risk | low risk | low risk | low risk |
| Koopman1996 | some concerns | some concerns | High | low risk | low risk |
| Medina2017 | low risk | low risk | some concerns | low risk | low risk |
| Buller2007 | low risk | low risk | low risk | low risk | low risk |
| Planquette2022 | low risk | low risk | some concerns | low risk | low risk |
| Righini2016 | low risk | low risk | low risk | some concerns | low risk |
| Findik2002 | some concerns | some concerns | low risk | low risk | low risk |
| Girolami2004 | some concerns | some concerns | some concerns | low risk | low risk |
| Belcaro1999 | low risk | low risk | low risk | low risk | low risk |
| Levine1996 | some concerns | some concerns | low risk | low risk | low risk |
| Romera2009 | low risk | low risk | low risk | low risk | low risk |
| Ridker2003 | some concerns | some concerns | low risk | low risk | low risk |
| Kearon2006 | some concerns | low risk | low risk | low risk | low risk |
| Monreal1999 | some concerns | low risk | some concerns | low risk | low risk |
| Riess2003 | some concerns | some concerns | some concerns | low risk | low risk |
| Simonneau1997 | low risk | low risk | some concerns | low risk | low risk |
| Fiessinger2005 | low risk | low risk | low risk | low risk | low risk |
| Kang2019 | low risk | low risk | low risk | low risk | low risk |
| Ramacciotti2004 | low risk | low risk | some concerns | low risk | low risk |
| Buller2008 | low risk | low risk | some concerns | low risk | low risk |
| Bynum1979 | low risk | low risk | some concerns | low risk | low risk |
| Marshall2020 | low risk | low risk | some concerns | low risk | low risk |

| | | | | | |
|---|---|---|---|---|---|
| Merli2001 | low risk | low risk | low risk | low risk | low risk |
| Gonzalez1999 | some concerns | low risk | High | some concerns | low risk |
| Wells2005 | some concerns | some concerns | some concerns | low risk | low risk |
| Raskob2016 | some concerns | low risk | some concerns | low risk | low risk |
| Lopaciuk1999 | low risk | low risk | some concerns | low risk | some concerns |
| Simes2014 | low risk | low risk | low risk | low risk | low risk |
| Meyer2002 | low risk | low risk | some concerns | low risk | low risk |
| Connor2020 | some concerns | some concerns | low risk | low risk | low risk |
| Schrag2023 | low risk | some concerns | some concerns | low risk | some concerns |
| Agnelli2020 | low risk | low risk | some concerns | High | low risk |
| Schulman2014 | low risk | low risk | low risk | low risk | low risk |
| Nisio2019 | low risk | low risk | some concerns | low risk | low risk |
| Becattini2011 | low risk | low risk | low risk | low risk | low risk |
| Breddin2001 | low risk | some concerns | some concerns | low risk | low risk |
| Buller1997 | low risk | some concerns | low risk | low risk | low risk |
| Bauersachs2018 | some concerns | low risk | some concerns | low risk | low risk |
| Prins2018 | some concerns | low risk | some concerns | low risk | low risk |
| Jimenez2007 | low risk | low risk | some concerns | low risk | low risk |
| Eriksson2005 | low risk | low risk | low risk | low risk | low risk |
| Veiga2000 | some concerns | some concerns | some concerns | low risk | low risk |
| Nakamura2015 | some concerns | some concerns | low risk | low risk | low risk |
| Cirujeda2006 | some concerns | some concerns | some concerns | low risk | low risk |
| Perez2010 | low risk | some concerns | low risk | low risk | low risk |
| Wells2016 | low risk | some concerns | low risk | low risk | low risk |
| Weitz2017 | low risk | low risk | some concerns | low risk | low risk |
| Hull2006 | low risk | low risk | low risk | low risk | low risk |
| Buller2011 | low risk | low risk | low risk | low risk | low risk |

| | | | | | |
|---|---|---|---|---|---|
| Lee2003 | low risk | low risk | some concerns | some concerns | low risk |
| Harenberg2000 | low risk | low risk | low risk | low risk | low risk |
| Schulman2013 | low risk | some concerns | some concerns | low risk | low risk |
| Harenberg2001 | low risk | low risk | low risk | low risk | low risk |
| Male2020 | low risk | low risk | low risk | low risk | low risk |
| Weycker2018 | some concerns | some concerns | High | some concerns | some concerns |
| Agnelli2007 | low risk | low risk | some concerns | low risk | low risk |
| Wysokinski2019 | some concerns | low risk | low risk | low risk | low risk |
| Harenberg2003 | low risk | low risk | low risk | low risk | low risk |
| Young2018 | low risk | low risk | low risk | low risk | low risk |
| Hull1979 | some concerns | low risk | low risk | low risk | low risk |
| Beyer2017 | low risk | low risk | low risk | low risk | low risk |

## 4.2 Prevention

| study | D1 | D2 | D3 | D4 | D5 |
|---|---|---|---|---|---|
| Wang2023 | some concerns | low risk | low risk | low risk | low risk |
| Vadhan2020 | some concerns | low risk | low risk | low risk | low risk |
| Turpie2009 | low risk | low risk | some concerns | low risk | low risk |
| Eriksson2010 | low risk | low risk | some concerns | low risk | some concerns |
| Fisher2007 | low risk | low risk | low risk | some concerns | low risk |
| Kolluri2016 | some concerns | some concerns | low risk | some concerns | low risk |
| Bergqvist1996 | some concerns | some concerns | low risk | low risk | low risk |
| Kakkar2004 | low risk | low risk | low risk | some concerns | low risk |
| Eriksson2007 | some concerns | some concerns | High | low risk | low risk |
| Fuji2008 | some concerns | low risk | low risk | low risk | low risk |
| Eriksson2003 | low risk | some concerns | low risk | low risk | low risk |
| Haas2012 | low risk | low risk | low risk | low risk | High |
| Verhamme2021 | low risk | low risk | low risk | low risk | low risk |
| Selby2015 | low risk | low risk | some concerns | low risk | low risk |
| Eikelboom2016 | low risk | low risk | low risk | low risk | low risk |
| Eriksson2007 | low risk | low risk | low risk | low risk | low risk |
| Weitz2021 | low risk | low risk | low risk | low risk | low risk |
| Becattini2022 | low risk | low risk | low risk | low risk | low risk |
| Agnelli2007 | some concerns | High | low risk | some concerns | low risk |
| Carrier2019 | low risk | low risk | low risk | low risk | low risk |
| Planes1999 | low risk | low risk | low risk | low risk | low risk |
| Woller2012 | some concerns | some concerns | some concerns | low risk | low risk |
| Fuji2014 | some concerns | some concerns | low risk | low risk | low risk |
| Eriksson2006 | some concerns | some concerns | low risk | low risk | low risk |
| Perry2010 | low risk | low risk | low risk | low risk | low risk |
| Colwell2005 | low risk | low risk | low risk | some concerns | low risk |

| | | | | | |
|---|---|---|---|---|---|
| Ramacciotti2022 | low risk | low risk | low risk | low risk | low risk |
| Ageno2021 | some concerns | some concerns | low risk | some concerns | low risk |
| Levine2012 | low risk | low risk | low risk | low risk | low risk |
| Carlo1999 | low risk | low risk | some concerns | low risk | low risk |
| Eriksson2011 | low risk | low risk | low risk | low risk | low risk |
| Francis2003 | low risk | low risk | low risk | some concerns | low risk |
| Samama2020 | low risk | low risk | low risk | low risk | low risk |
| Heit1997 | some concerns | low risk | low risk | low risk | low risk |
| Eriksson2003 | low risk | low risk | low risk | low risk | low risk |
| Colwell1995 | some concerns | low risk | High | low risk | low risk |
| Lassen2012 | low risk | low risk | low risk | low risk | low risk |
| Lassen2010 | low risk | low risk | low risk | low risk | low risk |
| Samama2002 | low risk | low risk | low risk | low risk | low risk |
| Menzin1994 | some concerns | low risk | High | low risk | low risk |
| Wirth2001 | some concerns | some concerns | some concerns | low risk | low risk |
| Burrows2001 | some concerns | low risk | High | some concerns | low risk |
| Leizorovicz2004 | some concerns | low risk | low risk | low risk | low risk |
| Kleber2003 | some concerns | low risk | some concerns | low risk | low risk |
| Heit2000 | low risk | low risk | some concerns | low risk | low risk |
| Cho2013 | low risk | low risk | low risk | low risk | low risk |
| Ginsberg2009 | low risk | low risk | low risk | low risk | High |
| Song2018 | low risk | low risk | low risk | low risk | low risk |
| Fuji2008 | some concerns | low risk | low risk | some concerns | some concerns |
| Eriksson2007 | low risk | low risk | low risk | low risk | low risk |
| Eriksson2005 | low risk | low risk | low risk | low risk | low risk |
| Schellong2010 | low risk | some concerns | some concerns | some concerns | low risk |
| Eriksson2008 | low risk | low risk | low risk | low risk | low risk |

| | | | | | |
|---|---|---|---|---|---|
| Eriksson2004 | some concerns | low risk | low risk | low risk | low risk |
| Weitz2019 | low risk | low risk | High | low risk | low risk |
| Abbas2016 | some concerns | low risk | some concerns | High | low risk |
| Spyropoulos2018 | low risk | low risk | low risk | low risk | low risk |
| Agnelli2009 | low risk | low risk | low risk | low risk | low risk |
| Marlovits2007 | some concerns | some concerns | low risk | low risk | low risk |
| Oliveira2022 | low risk | High | some concerns | low risk | low risk |
| Lechler1996 | some concerns | low risk | low risk | low risk | low risk |
| Bruntink2017 | low risk | low risk | High | low risk | low risk |
| Samama1999 | low risk | low risk | low risk | low risk | low risk |
| Agnelli2009 | low risk | low risk | low risk | low risk | low risk |
| Zhao2023 | low risk | low risk | High | low risk | low risk |
| Kakkar1993 | low risk | low risk | low risk | low risk | some concerns |
| Michot2002 | low risk | High | High | low risk | low risk |
| Shorr2012 | some concerns | some concerns | low risk | some concerns | low risk |
| Fitzgerald2001 | low risk | low risk | some concerns | low risk | low risk |
| Francis2002 | low risk | low risk | low risk | low risk | low risk |
| Raskob2010 | low risk | low risk | low risk | low risk | low risk |
| Veen2021 | low risk | low risk | some concerns | low risk | low risk |
| Haas2006 | low risk | low risk | low risk | low risk | low risk |
| Wang2022 | some concerns | some concerns | low risk | low risk | low risk |
| Colwell1999 | some concerns | low risk | some concerns | low risk | low risk |
| Prandoni2002 | low risk | low risk | some concerns | low risk | low risk |
| Adrichem2017 | some concerns | low risk | some concerns | low risk | low risk |
| Cohen2006 | low risk | low risk | low risk | low risk | low risk |
| Lassen2007 | low risk | low risk | low risk | low risk | low risk |
| Mahe2005 | low risk | low risk | low risk | low risk | low risk |

| | | | | | |
|---|---|---|---|---|---|
| Ishi2013 | low risk | low risk | low risk | some concerns | low risk |
| Verhamme2013 | some concerns | low risk | low risk | low risk | low risk |
| Riess2010 | low risk | low risk | low risk | low risk | low risk |
| Turpie2005 | low risk | low risk | low risk | low risk | low risk |
| Turpie2009 | some concerns | low risk | low risk | some concerns | low risk |
| McLeod2001 | low risk | low risk | low risk | low risk | low risk |
| Eriksson2008 | low risk | low risk | low risk | low risk | low risk |
| Leclerc1996 | low risk | low risk | low risk | low risk | low risk |
| Zhang2021 | some concerns | low risk | low risk | low risk | low risk |
| Dobesh2003 | some concerns | low risk | low risk | some concerns | some concerns |
| Hull2000 | low risk | low risk | low risk | low risk | low risk |
| Colwell2003 | low risk | low risk | low risk | low risk | low risk |
| Haas2011 | some concerns | low risk | low risk | low risk | low risk |
| Goldhaber2011 | low risk | low risk | low risk | low risk | low risk |
| Comp1998 | some concerns | low risk | High | low risk | low risk |
| Hull2010 | low risk | low risk | low risk | low risk | low risk |
| Planes1997 | some concerns | low risk | low risk | low risk | High |
| Lassen2010 | low risk | low risk | low risk | some concerns | low risk |
| Borstad1992 | some concerns | low risk | low risk | low risk | low risk |
| Samama2014 | low risk | low risk | High | low risk | low risk |
| Eriksson2007 | low risk | low risk | low risk | low risk | low risk |
| Lassen2008 | low risk | low risk | low risk | low risk | low risk |
| Piazza2023 | low risk | low risk | low risk | low risk | low risk |
| Bueller2015 | low risk | low risk | some concerns | low risk | low risk |
| Bergqvist2002 | low risk | low risk | low risk | low risk | low risk |
| Long2014 | some concerns | some concerns | some concerns | low risk | low risk |
| Weitz2020 | low risk | some concerns | High | low risk | low risk |

| | | | | | |
|---|---|---|---|---|---|
| Anderson2018 | low risk | low risk | low risk | low risk | low risk |
| Fuji2014 | some concerns | low risk | low risk | low risk | low risk |
| Geerts1996 | low risk | low risk | low risk | low risk | low risk |
| Eriksson2009 | some concerns | low risk | low risk | low risk | low risk |
| Abbas2017 | some concerns | low risk | some concerns | low risk | low risk |
| Pai2018 | low risk | low risk | low risk | low risk | low risk |
| Turpie2001 | some concerns | low risk | low risk | low risk | low risk |
| Turpie2009 | low risk | low risk | some concerns | low risk | low risk |
| Levine1991 | some concerns | some concerns | some concerns | low risk | low risk |
| Brandt2022 | some concerns | low risk | low risk | some concerns | some concerns |
| Yi2014 | low risk | low risk | some concerns | low risk | some concerns |
| Agnelli2005 | some concerns | low risk | low risk | low risk | low risk |
| Eriksson2006 | some concerns | low risk | low risk | low risk | low risk |
| Connors2021 | low risk | low risk | low risk | low risk | low risk |
| Planes1998 | some concerns | low risk | low risk | low risk | low risk |
| Palumbo2011 | some concerns | some concerns | some concerns | low risk | low risk |
| Camporese2016 | some concerns | low risk | low risk | low risk | low risk |
| De2010 | some concerns | High | low risk | some concerns | some concerns |

Footnotes: D1=Randomization process; D2=Deviationsf from intended interventions.; D3=Measurement of the outcome; D4=Missing outcome data; D5=Selective reporting.

**Bias between paired drugs**
**Outcome: Recurrent VTE during treatment**

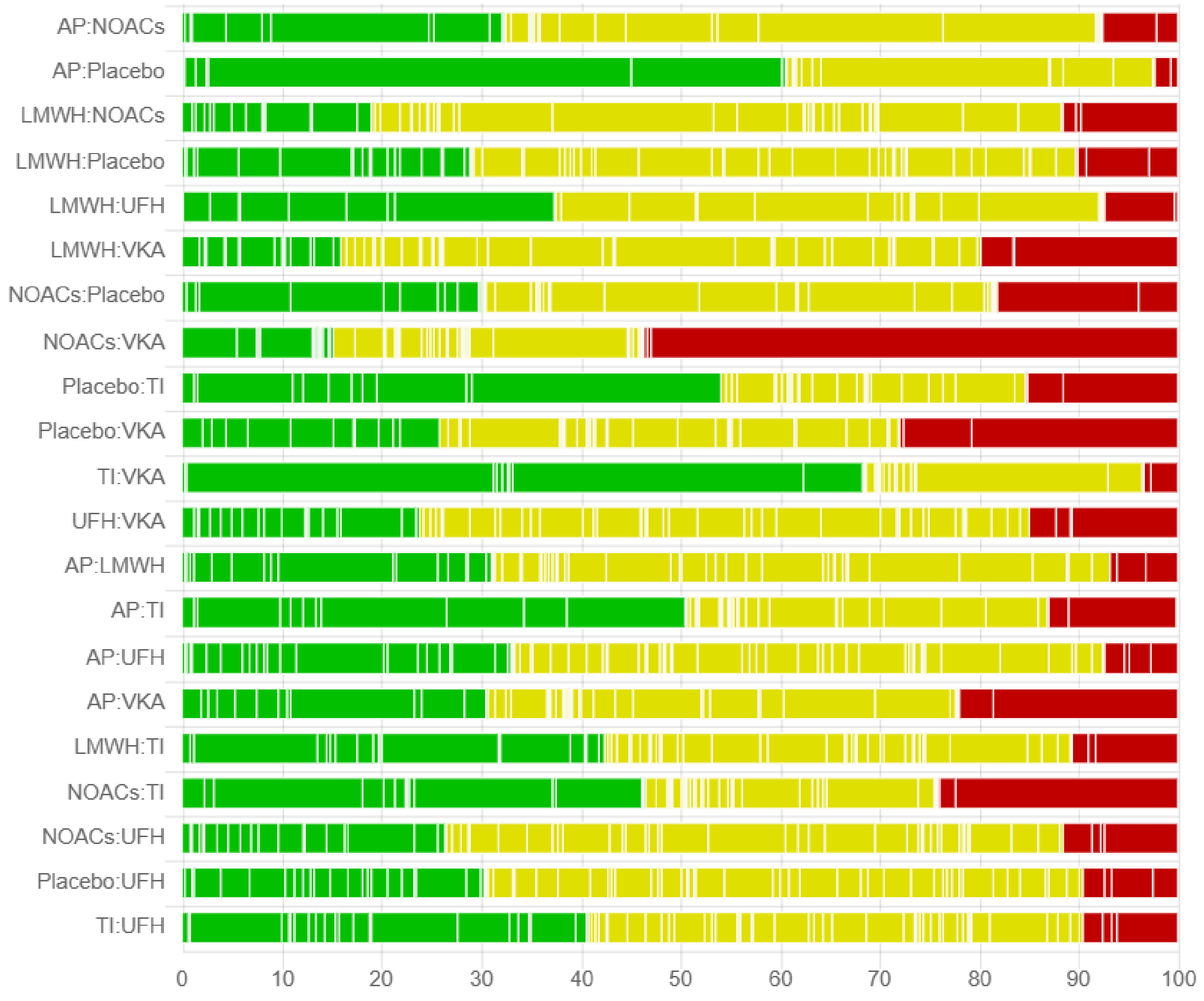

**Outcome: Major bleeding during treatment**

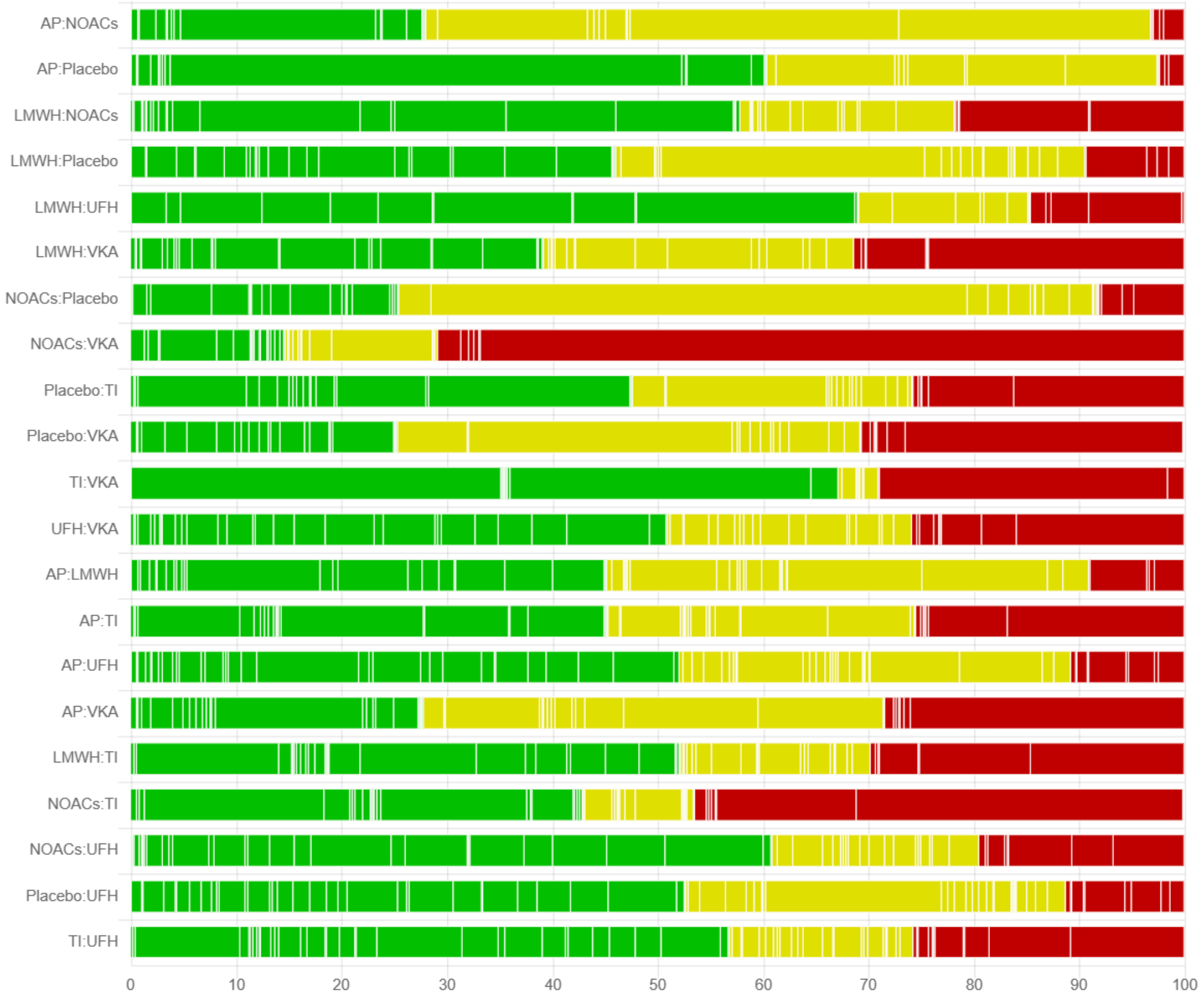

**Outcome: Clinical relevant non-major bleeding during treatment**

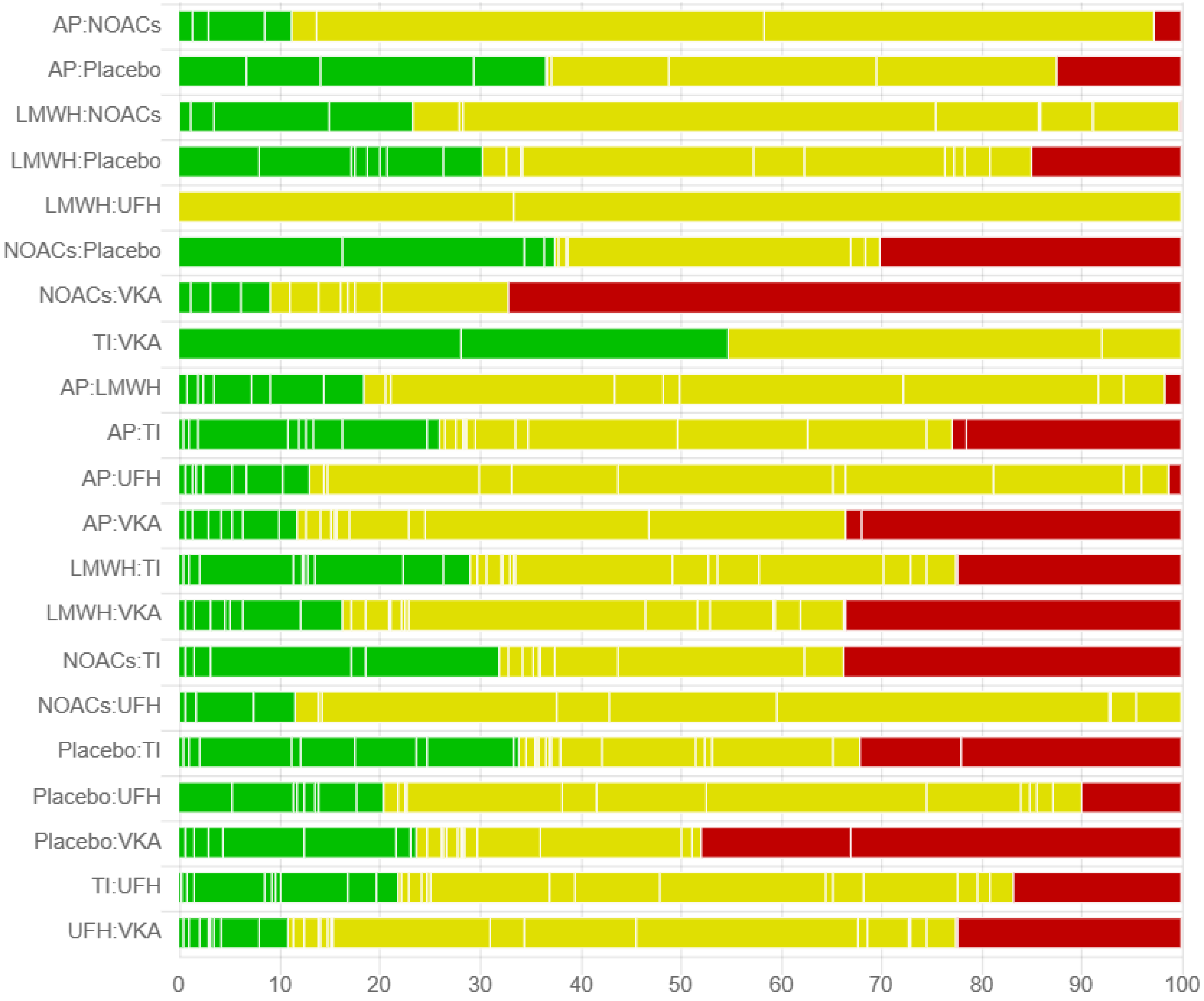

**Outcome: VTE related death during treatment**

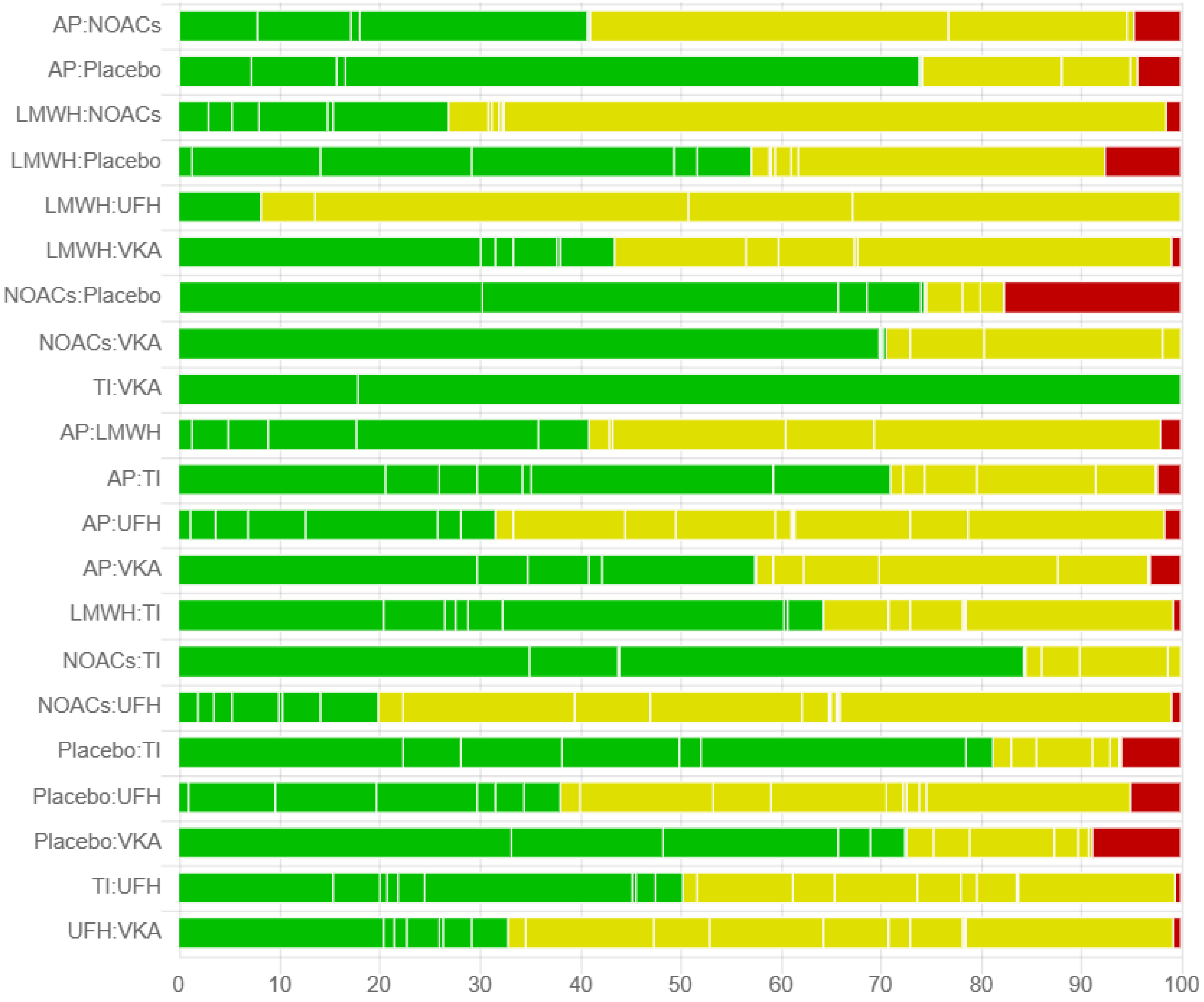

**Outcome: Fatal bleeding during treatment**

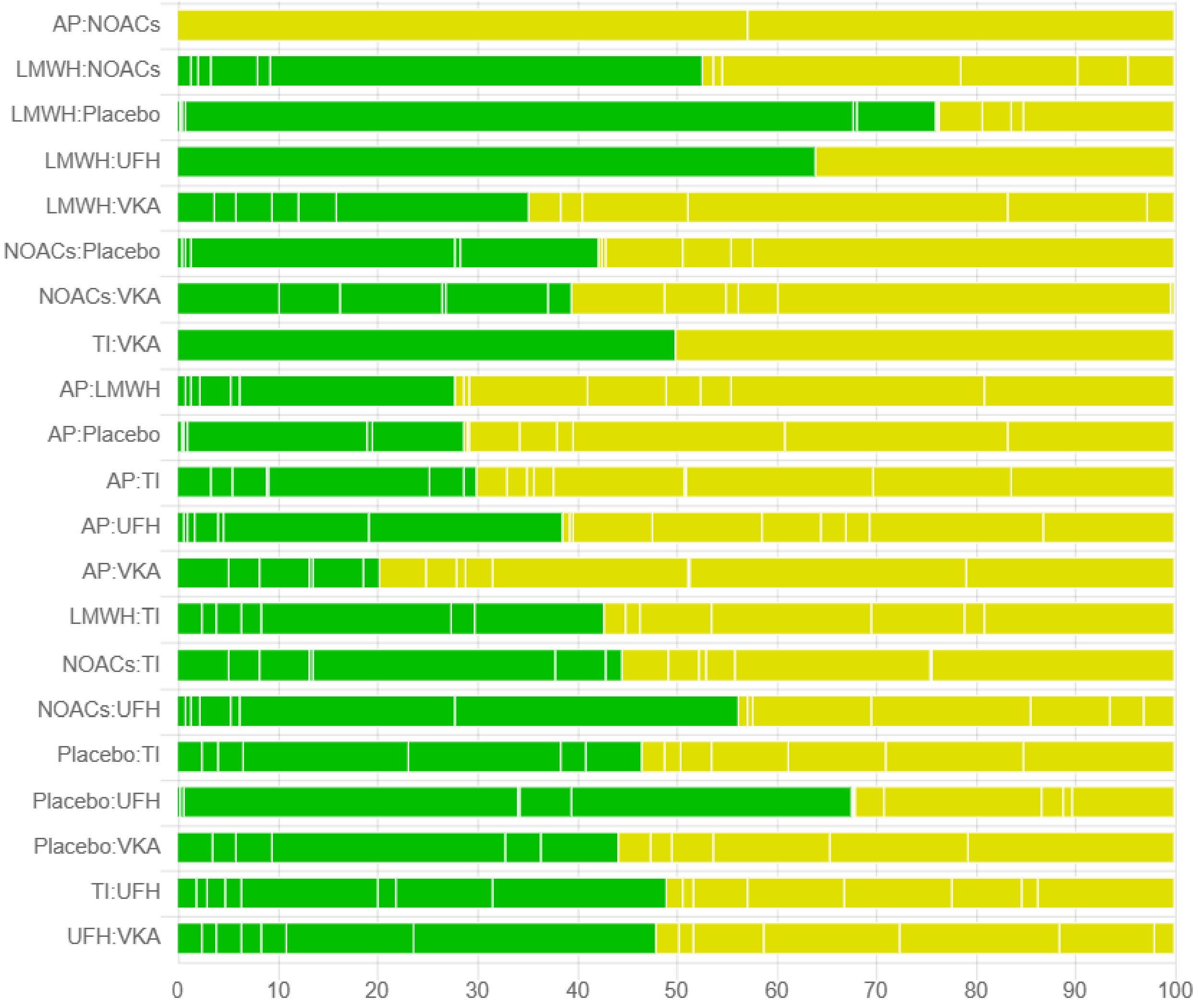

**Outcome: Adverse events during treatment**

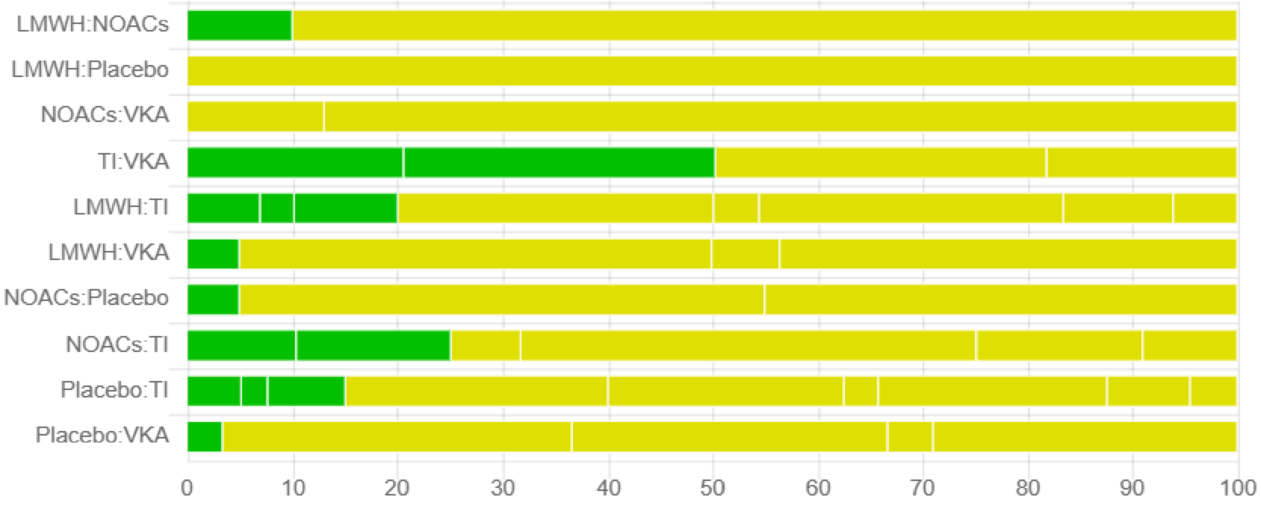


**Outcome: Clinical relevant non-major bleeding during prophylaxis**

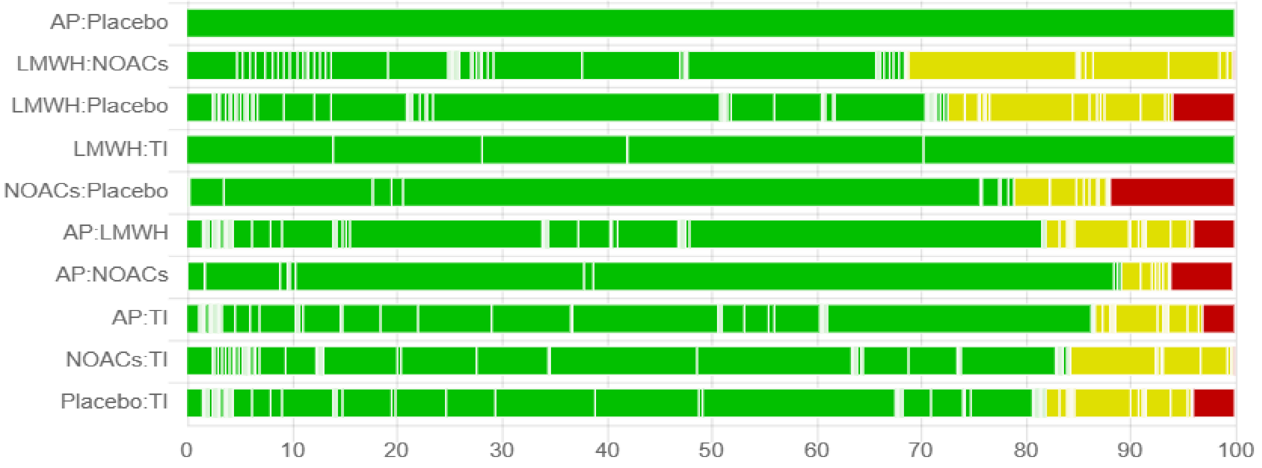

**Outcome: All-cause mortality during treatment**

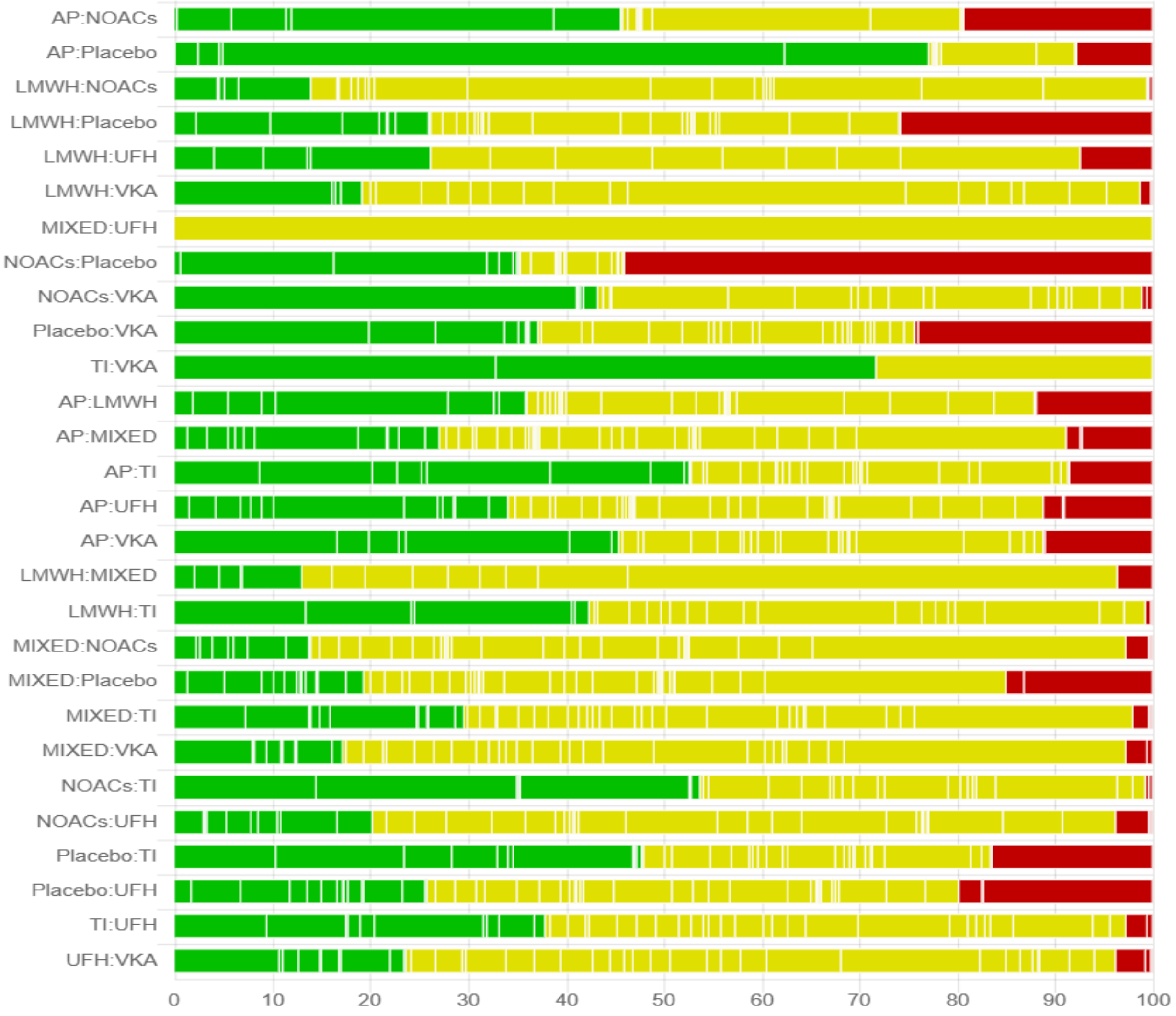

**Outcome: VTE during prophylaxis**

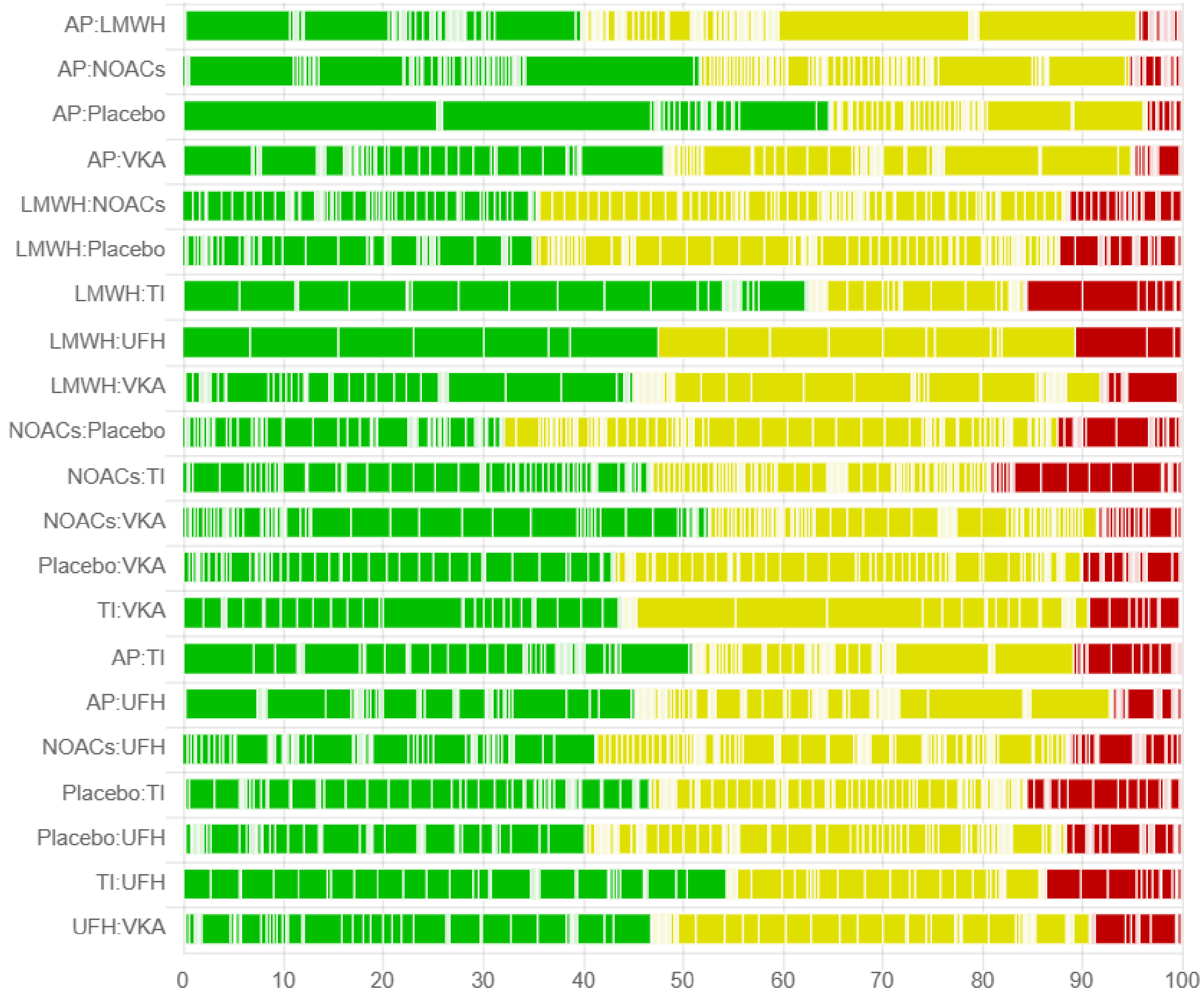

**Outcome: Major bleeding during prophylaxis**

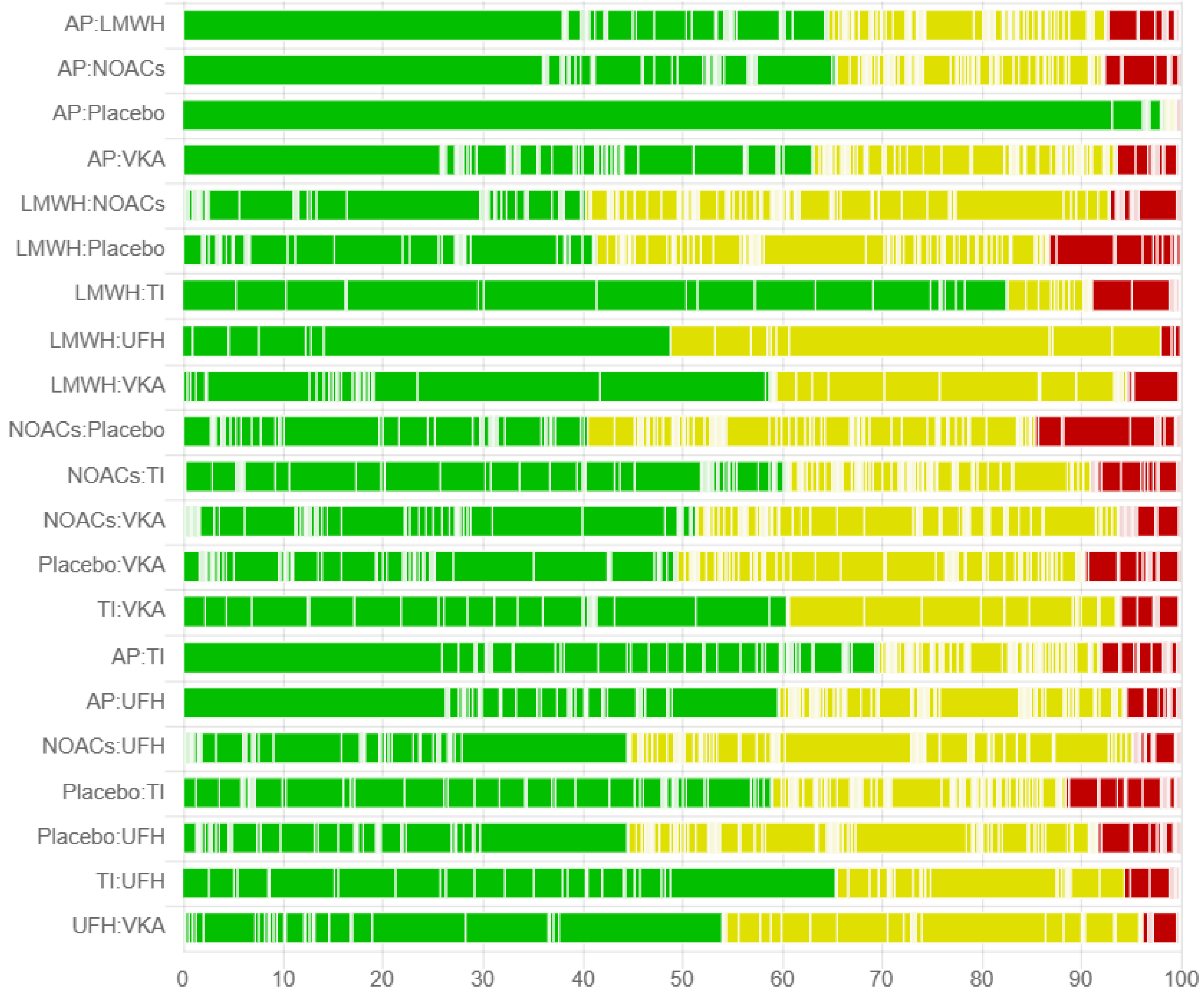

**Outcome：VTE related death during prophylaxis**

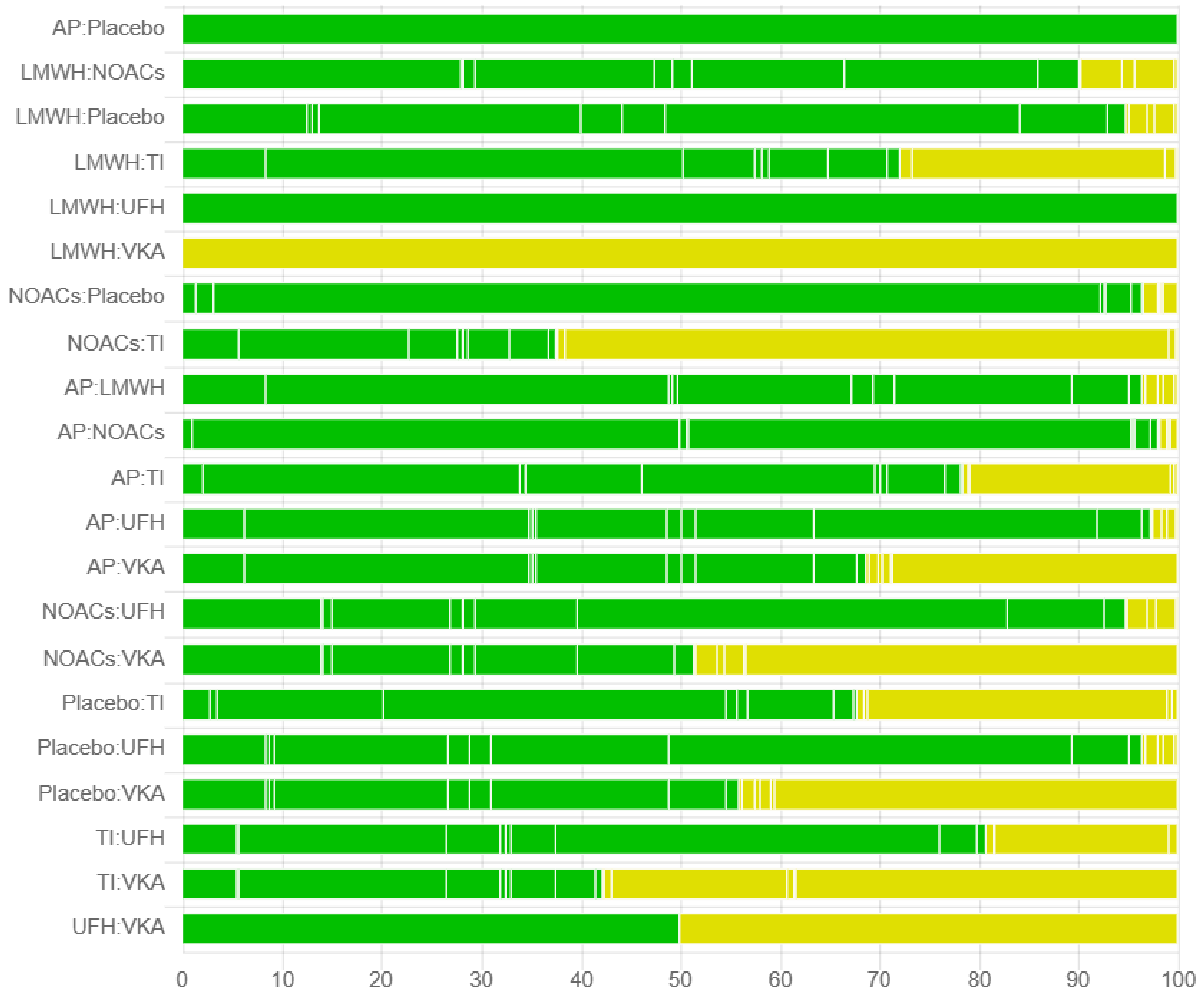

**Appendix 5: Other main results**

**5.1 Network plots**

**Outcome：Recurrent VTE during treatment**

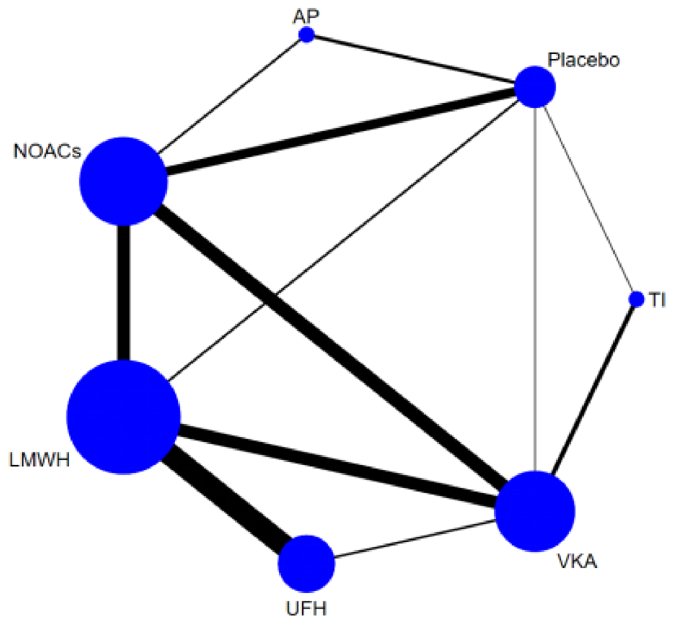


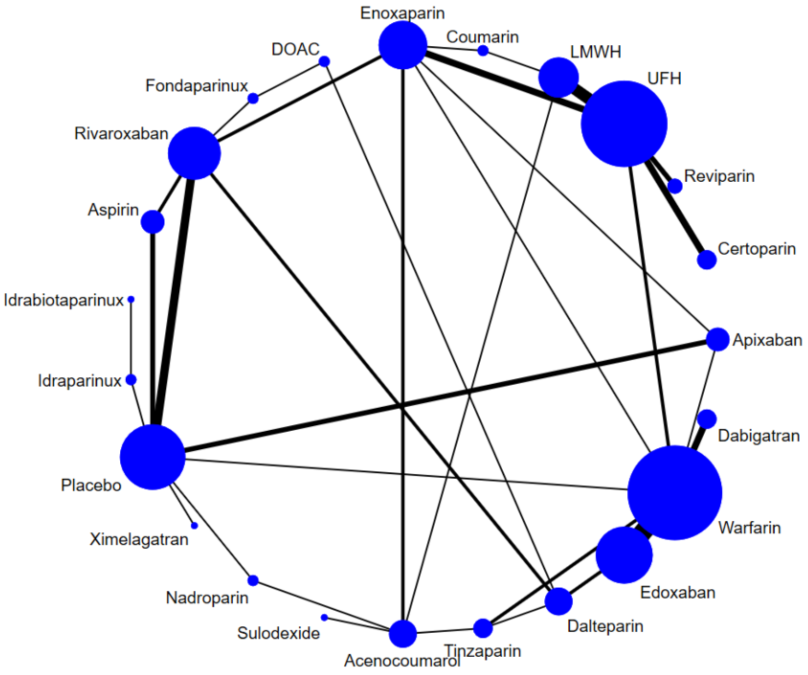

**Outcome：Major bleeding during treatment**

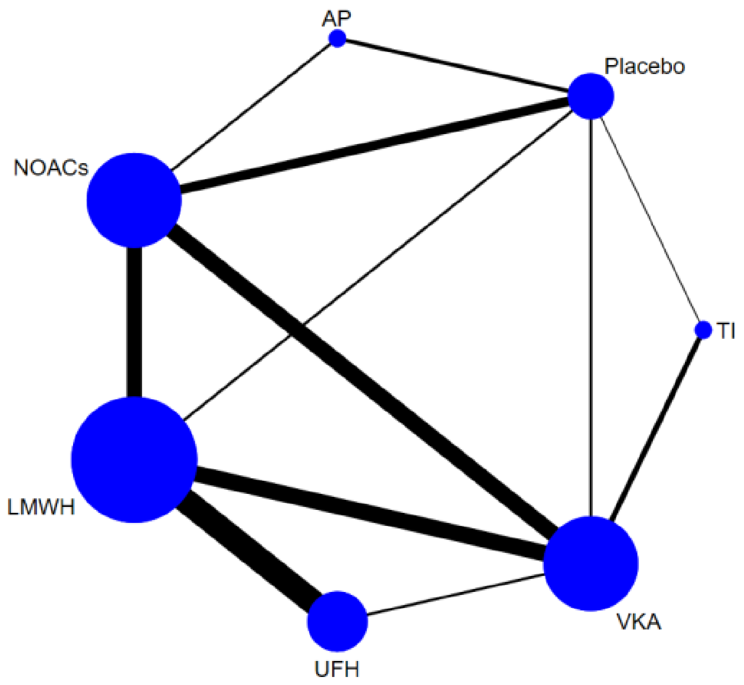


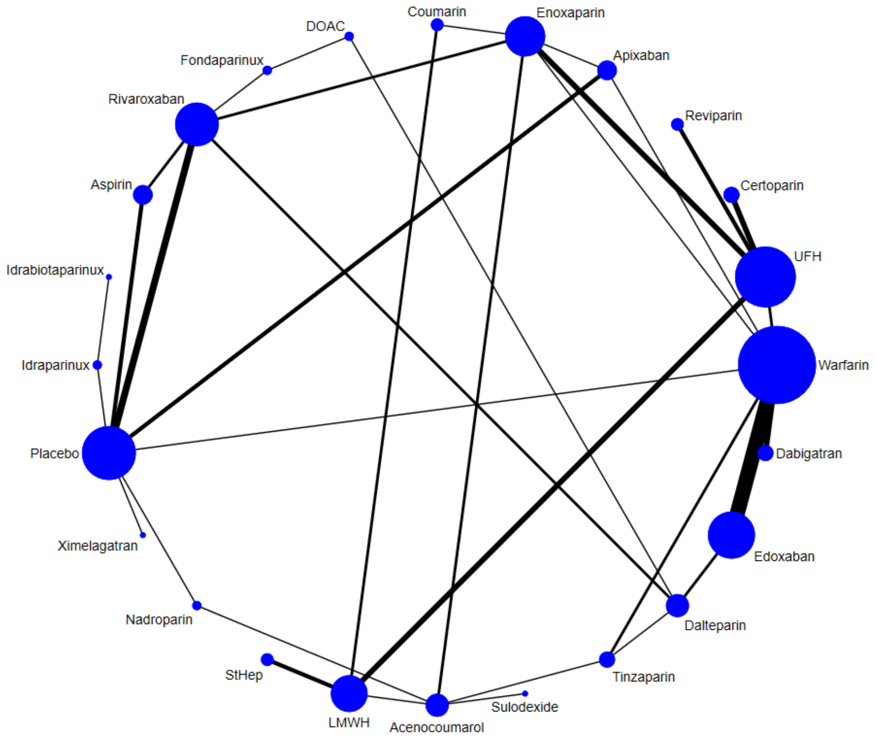

**Outcome: Clinical relevant non-major bleeding during treatment**

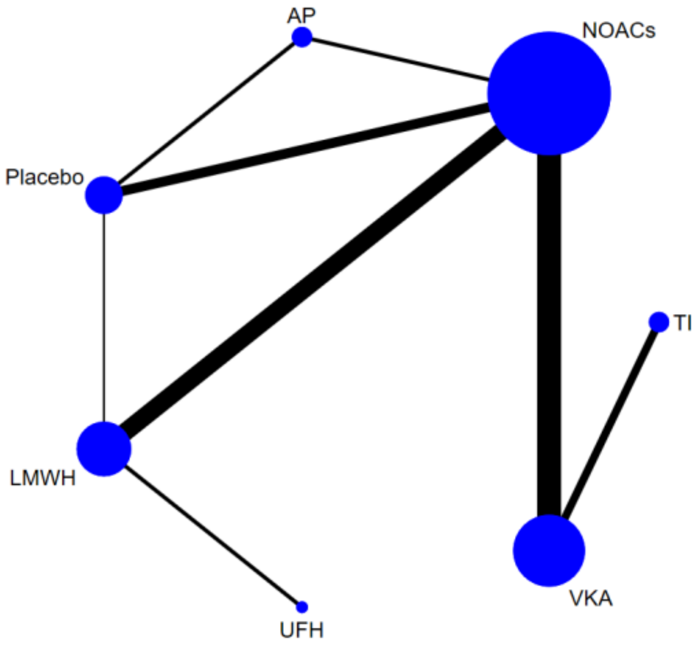


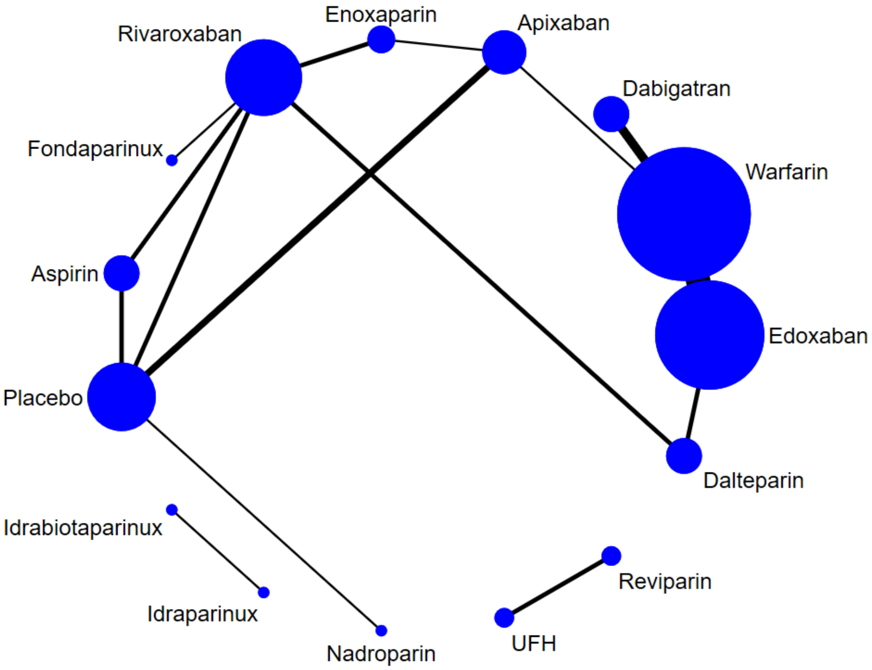

**Outcome: VTE related death during treatment**

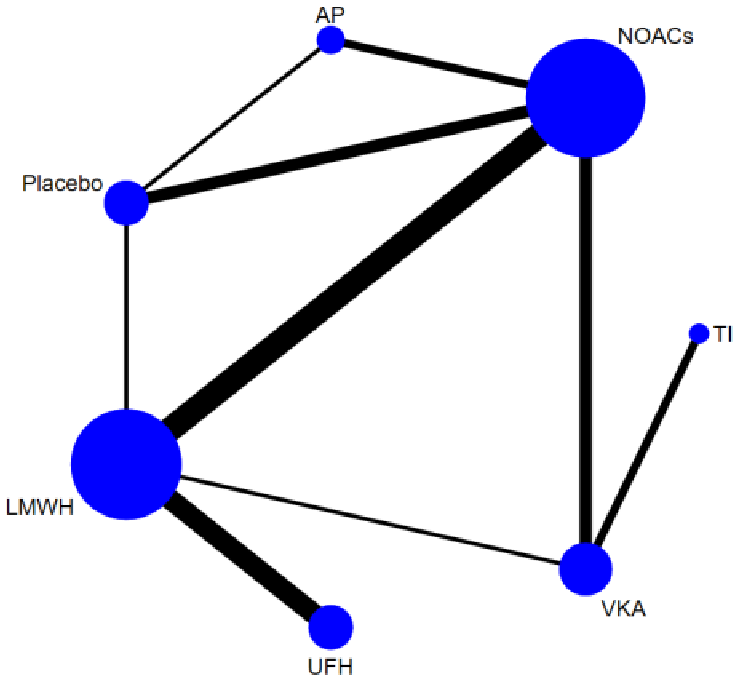


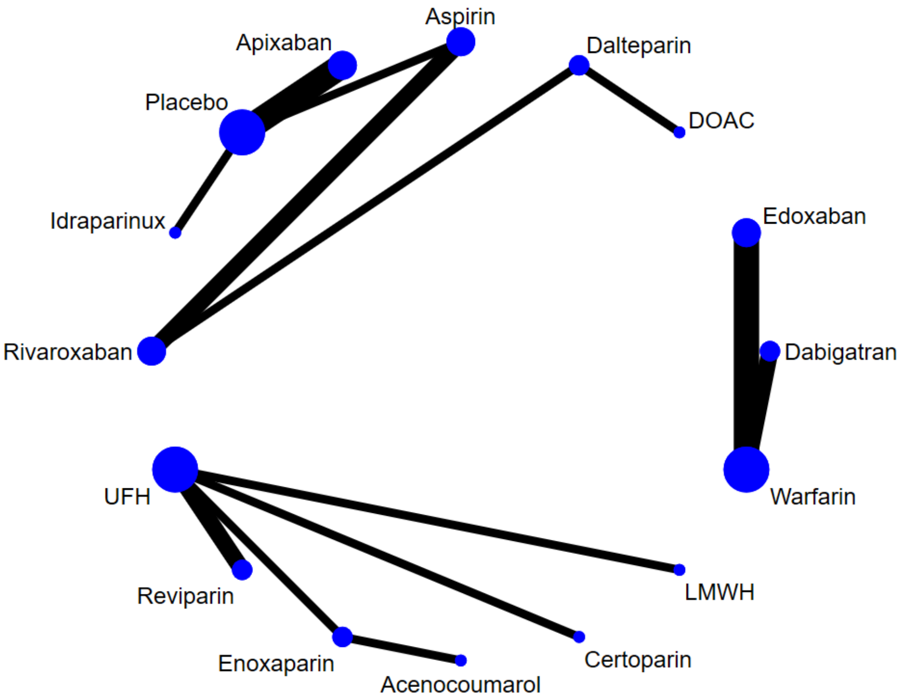

**Outcome: Fatal bleeding during treatment**

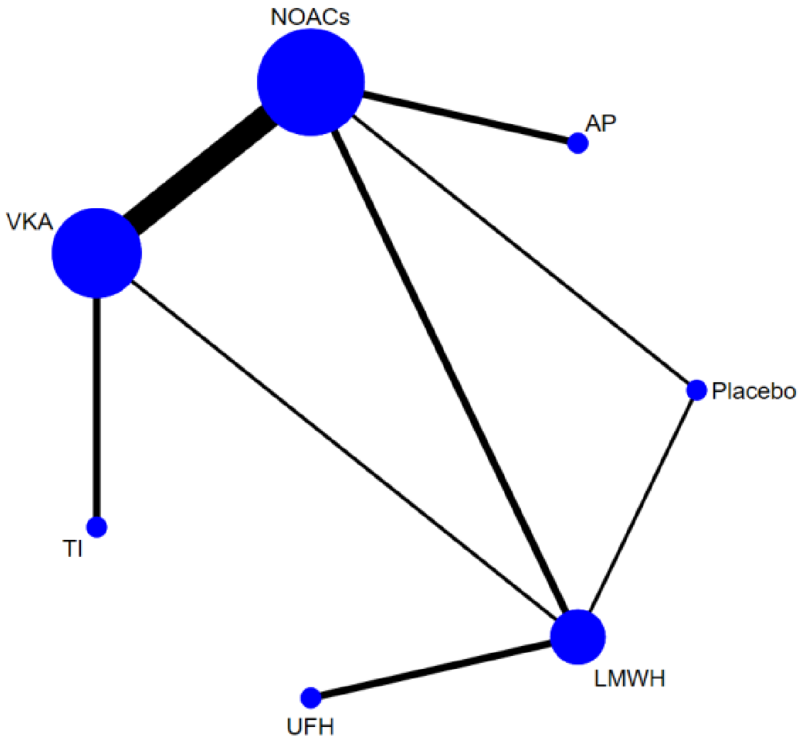


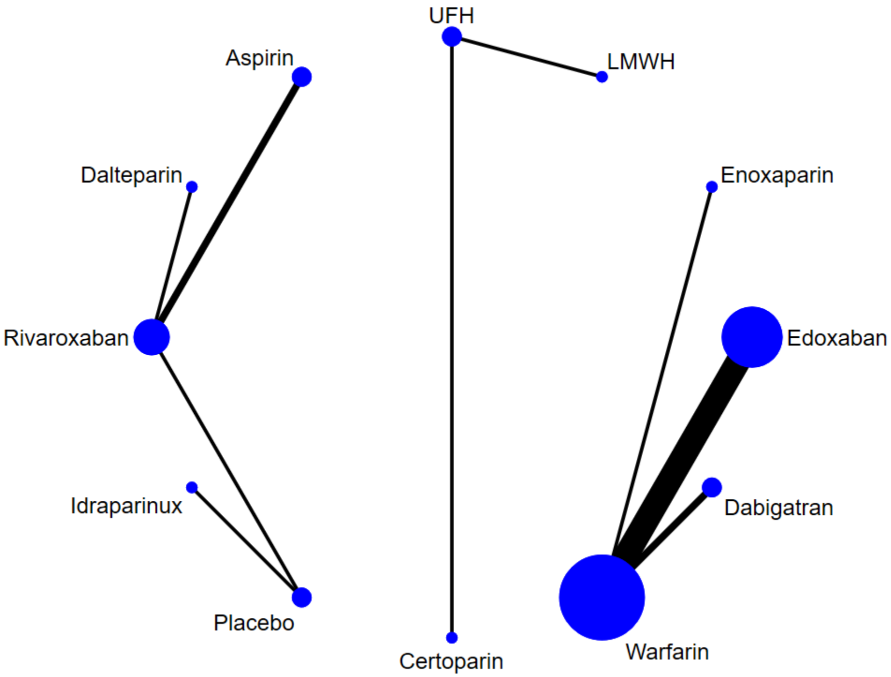

**Outcome: Adverse events during treatment**

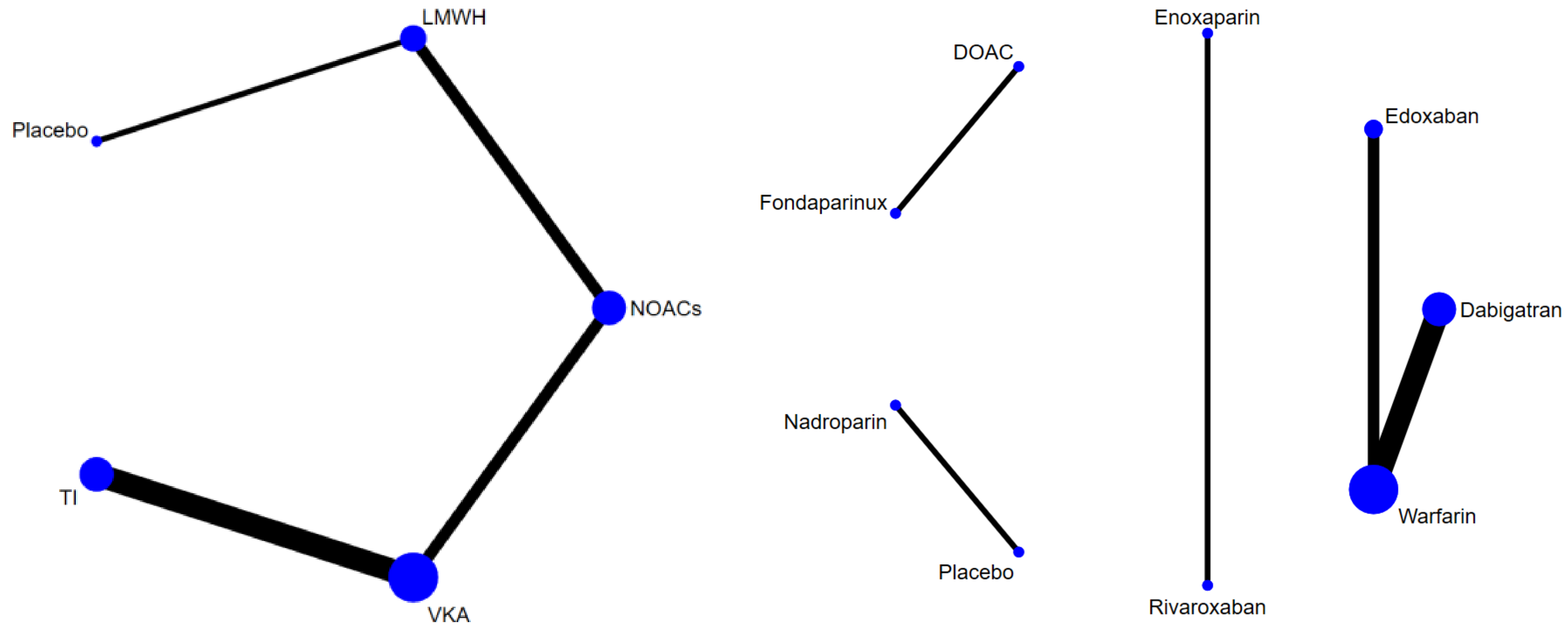

**Outcome: All-cause mortality during treatment**

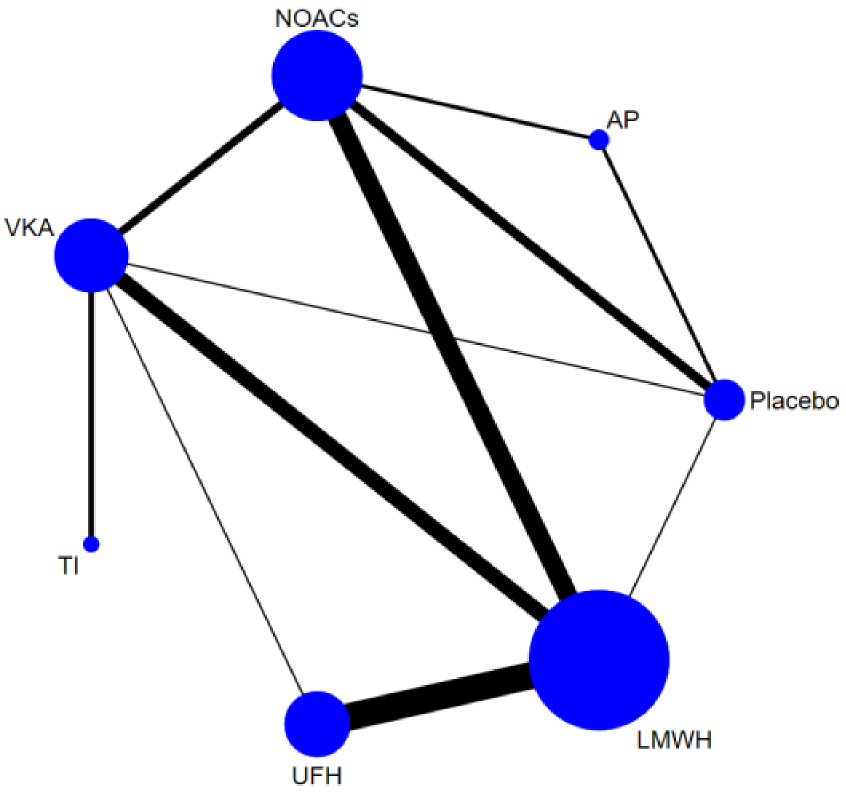


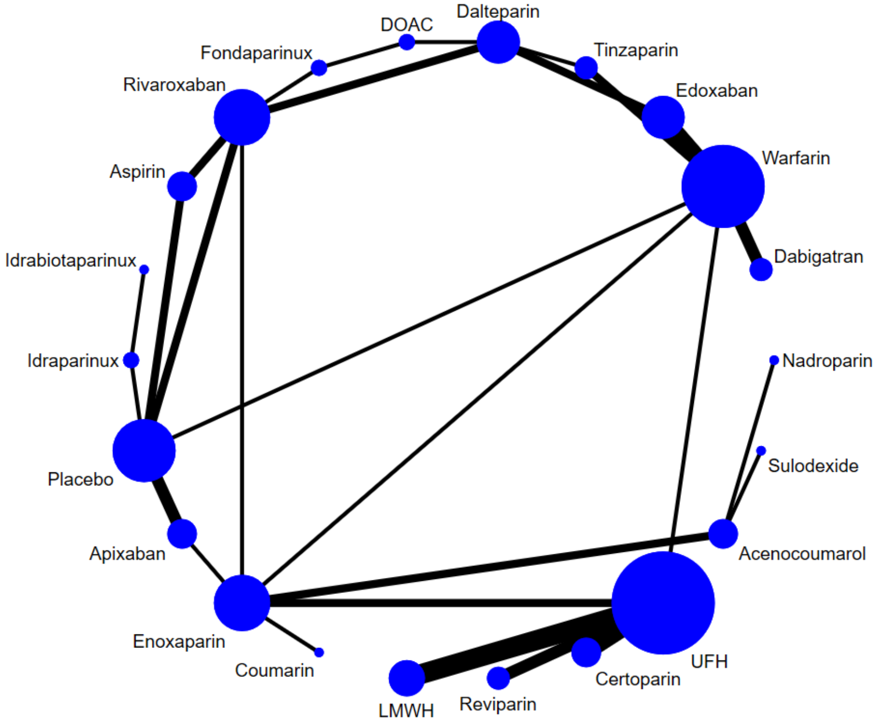

**Outcome: VTE during prophylaxis**

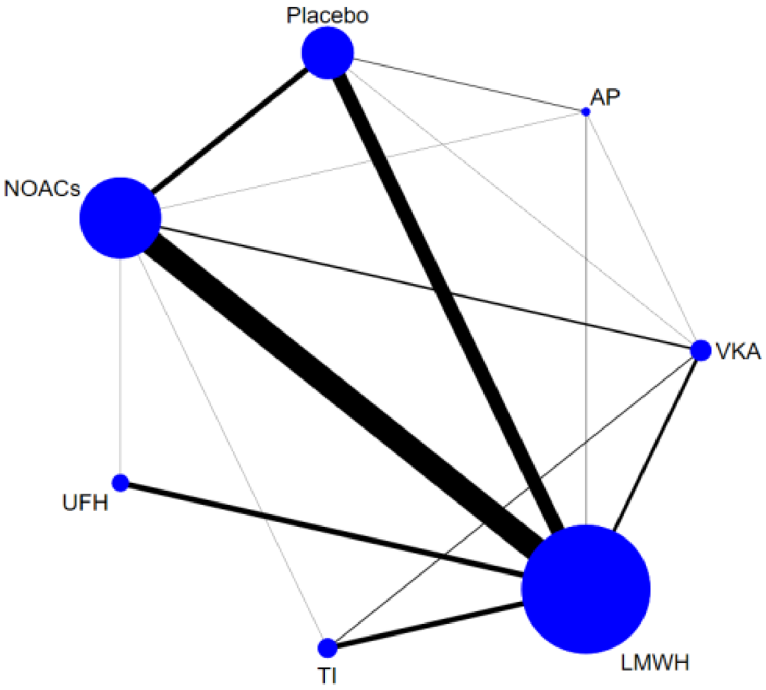


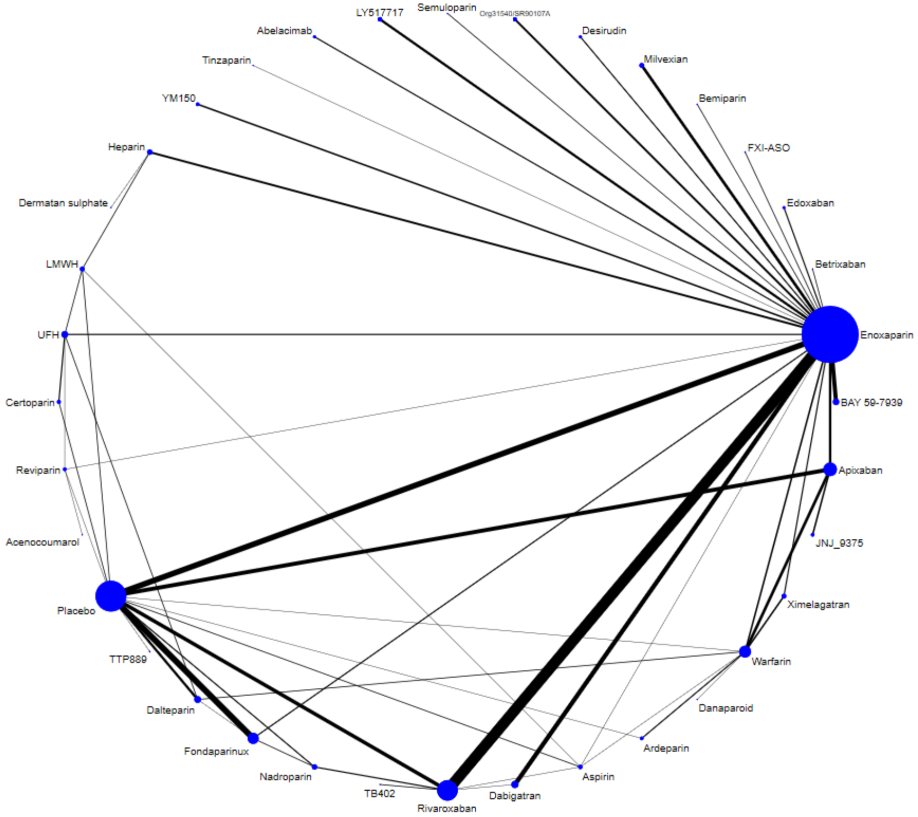

**Outcome: Major bleeding during prophylaxis**

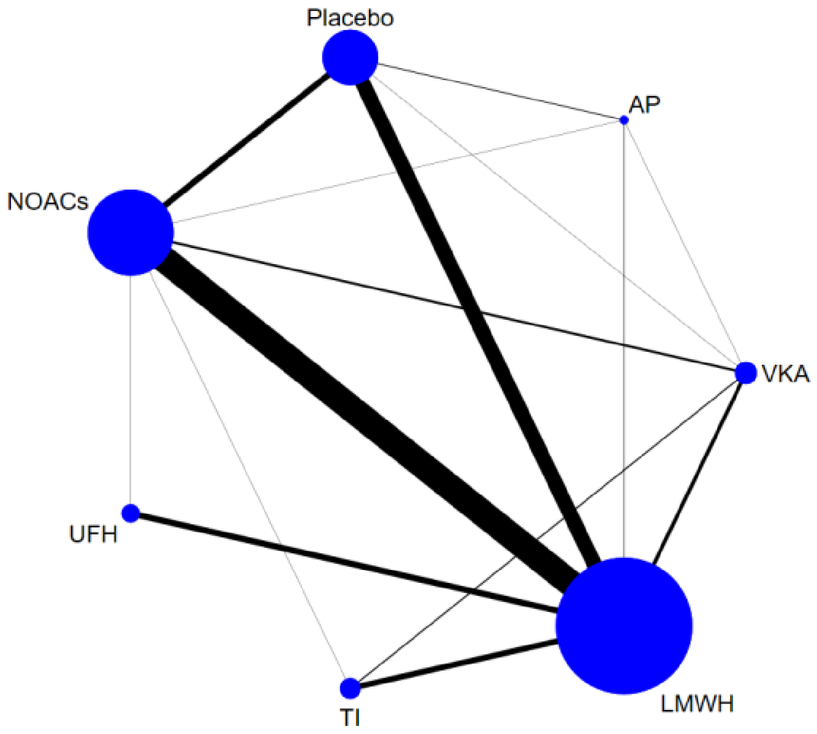


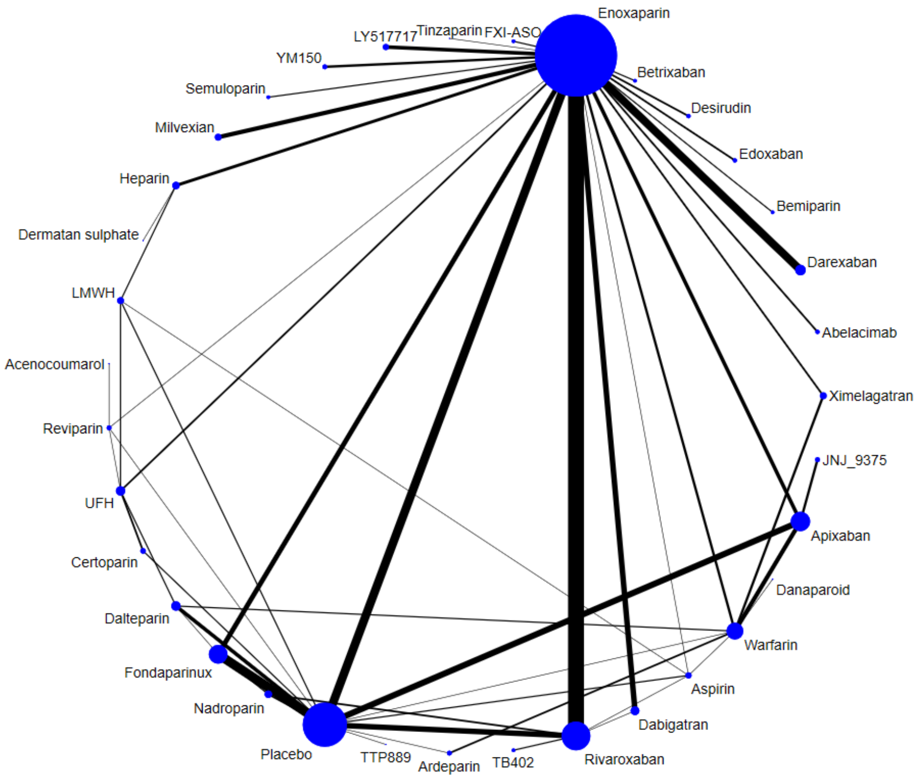

**Outcome: All-cause mortality during prophylaxis**

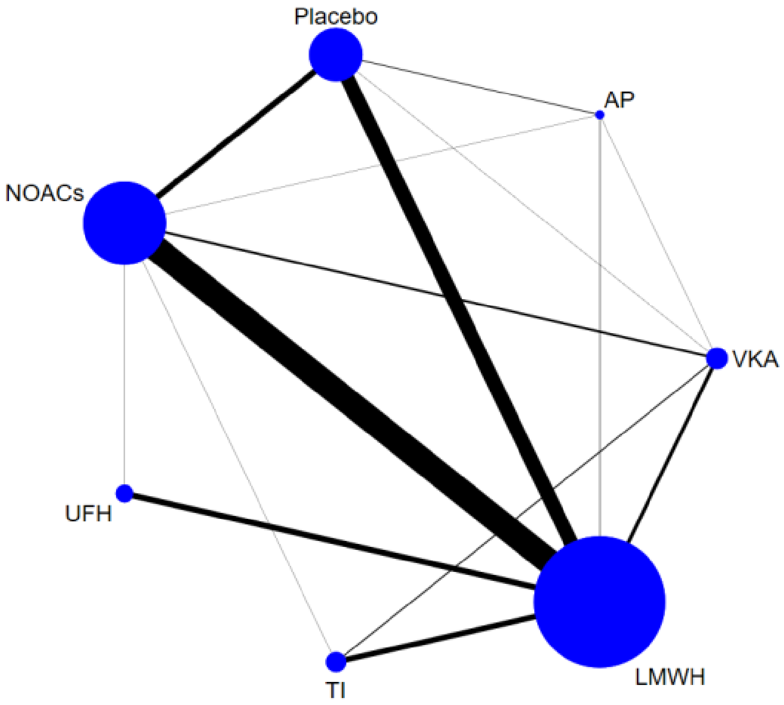


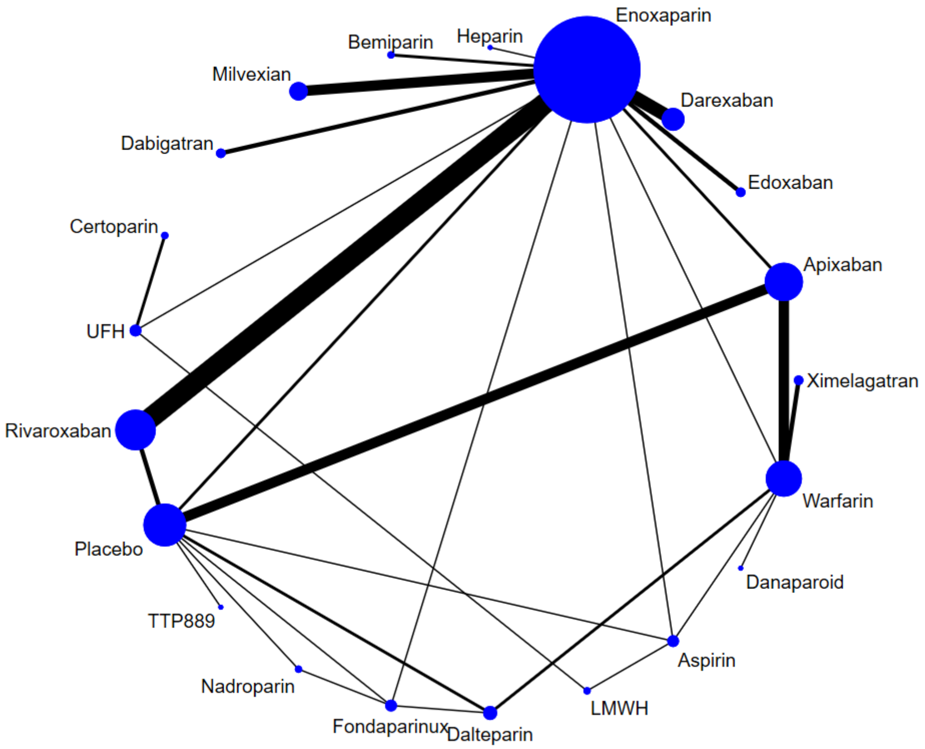

**Outcome: Clinical relevant non-major bleeding during prophylaxis**

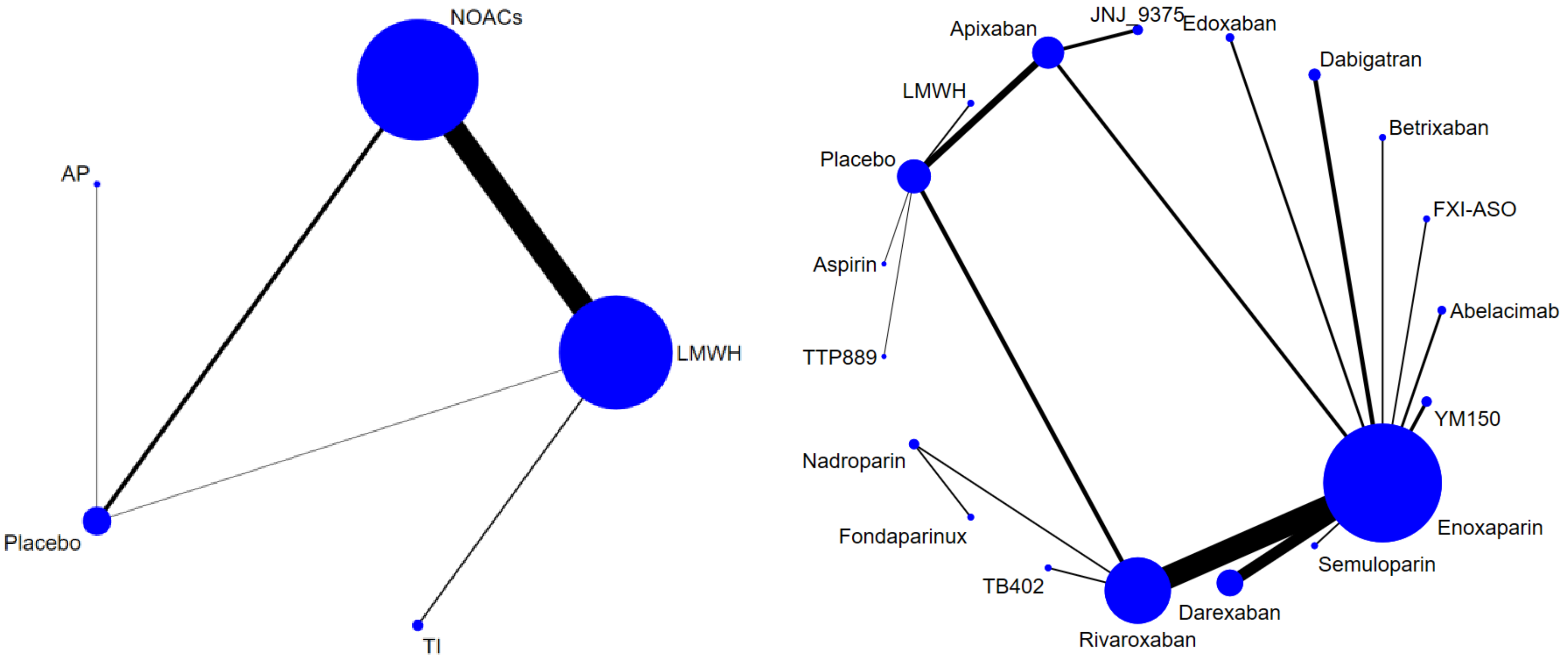

**Outcome：VTE related death during prophylaxis**

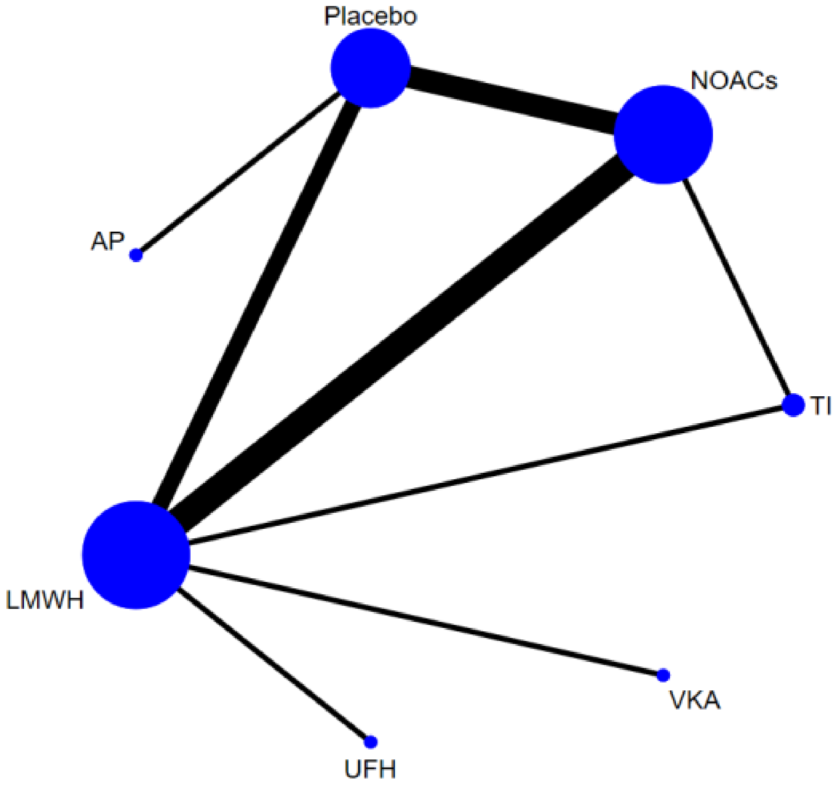


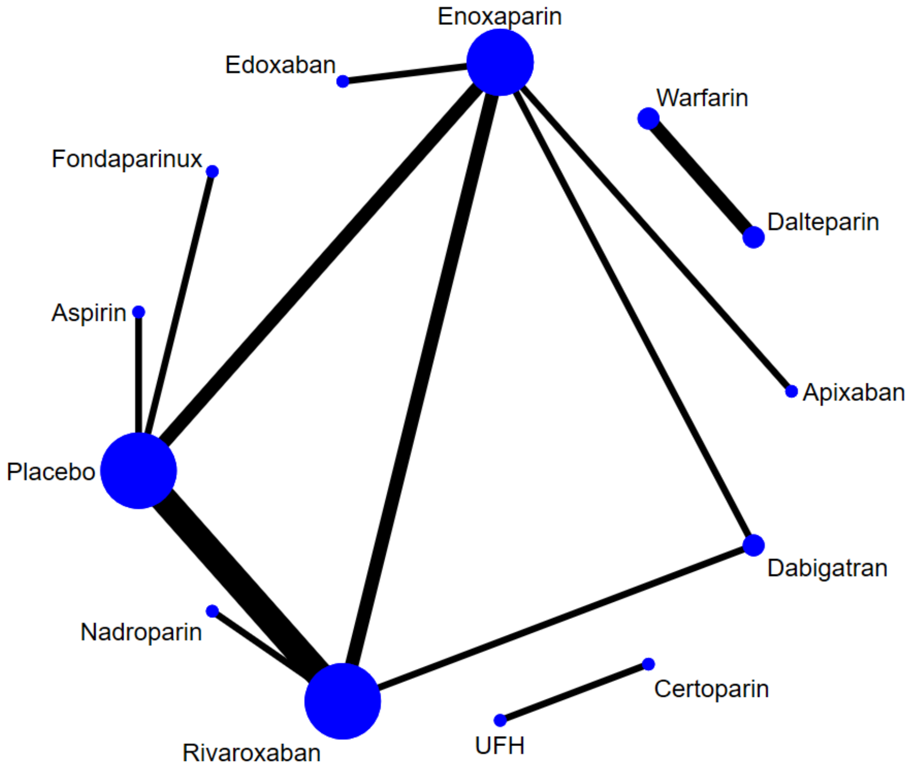

## 5.2 Summary of findings tables

### 5.2-1 AP vs. Placebo be used for treatment

Summary of findings:

AP compared to Placebo for treatment

Patient or population: VTE
Setting:
Intervention: AP
Comparison: Placebo

| Outcome<br>№ of participants<br>(studies) | Relative effect<br>(95% CI) | Anticipated absolute effects (95% CI) | | | Certainty | What happens |
|---|---|---|---|---|---|---|
| | | Placebo | AP | Difference | | |
| VTE<br>(3 RCTs) | OR 0.67<br>(0.46 to 0.96) | 1.5% | 1.0%<br>(0.7 to 1.5) | 0.5% fewer<br>(0.8 fewer to 0.1 fewer) | ⊕⊕⊕⊕<br>High | |
| Major bleeding<br>(3 RCTs) | OR 1.40<br>(0.59 to 3.30) | 9.4% | 12.6%<br>(5.7 to 25.4) | 3.3% more<br>(3.6 fewer to 16.1 more) | ⊕⊕⊕◯<br>Moderate[a] | |
| CRNMB<br>(2 RCTs) | OR 1.30<br>(0.74 to 2.50) | 3.0% | 3.9%<br>(2.3 to 7.2) | 0.9% more<br>(0.8 fewer to 4.2 more) | ⊕⊕◯◯<br>Low[a,b] | |
| VTE related death<br>(1 RCT) | OR 0.78<br>(0.25 to 2.50) | 1.0% | 0.8%<br>(0.2 to 2.4) | 0.2% fewer<br>(0.7 fewer to 1.4 more) | ⊕⊕⊕◯<br>Moderate[c] | |
| fatal bleeding<br>(0 RCTs) | OR 3.20<br>(0.26 to 52.00) | 0.0% | 0.0%<br>(0 to 0) | 0.0% fewer<br>(0 fewer to 0 fewer) | ⊕⊕◯◯<br>Low[a,c] | |
| All-cause mortality<br>(2 RCTs) | OR 0.83<br>(0.47 to 1.50) | 8.3% | 7.0%<br>(4.1 to 12) | 1.3% fewer<br>(4.2 fewer to 3.7 more) | ⊕⊕⊕◯<br>Moderate[b] | |

*The risk in the intervention group (and its 95% confidence interval) is based on the assumed risk in the comparison group and the relative effect of the intervention (and its 95% CI).

CI: confidence interval; OR: odds ratio

GRADE Working Group grades of evidence
High certainty: we are very confident that the true effect lies close to that of the estimate of the effect.
Moderate certainty: we are moderately confident in the effect estimate: the true effect is likely to be close to the estimate of the effect, but there is a possibility that it is substantially different.
Low certainty: our confidence in the effect estimate is limited: the true effect may be substantially different from the estimate of the effect.
Very low certainty: we have very little confidence in the effect estimate: the true effect is likely to be substantially different from the estimate of effect.

Explanations
a. Within-study bias
b. Heterogeneity
c. Imprecision

**5.2-2 LMWH vs. Placebo be used for treatment**

Summary of findings:

LMWH compared to Placebo for treatment

Patient or population: VTE
Setting:
Intervention: LMWH
Comparison: Placebo

| Outcome<br>№ of participants<br>(studies) | Relative effect<br>(95% CI) | Anticipated absolute effects (95% CI) | | | Certainty | What happens |
|---|---|---|---|---|---|---|
| | | Placebo | LMWH | Difference | | |
| Recurrent VTE<br>(2 RCTs) | OR 0.25<br>(0.18 to 0.35) | 1.5% | 0.4%<br>(0.3 to 0.5) | 1.1% fewer<br>(1.3 fewer to 1 fewer) | ⊕⊕⊕◯<br>Moderate[a] | |
| Major bleeding<br>(2 RCTs) | OR 2.2<br>(1.2 to 4.2) | 9.4% | 18.5%<br>(11 to 30.3) | 9.2% more<br>(1.7 more to 20.9 more) | ⊕⊕◯◯<br>Low[a,b] | |
| CRNMB<br>(1 RCT) | OR 1.10<br>(0.66 to 1.90) | 3.0% | 3.3%<br>(2 to 5.6) | 0.3% more<br>(1 fewer to 2.6 more) | ⊕⊕◯◯<br>Low[a,c] | |
| VTE related death<br>(1 RCT) | OR 0.75<br>(0.21 to 3.00) | 1.0% | 0.7%<br>(0.2 to 2.9) | 0.2% fewer<br>(0.8 fewer to 1.9 more) | ⊕⊕◯◯<br>Low[a,d] | |
| fatal bleeding<br>(1 RCT) | OR 3.50<br>(0.56 to 35.00) | 0.0% | 0.0%<br>(0 to 0) | 0.0% fewer<br>(0 fewer to 0 fewer) | ⊕⊕⊕◯<br>Moderate[d] | |
| adverse event<br>(1 RCT) | not estimable | 0.0% | 0.0%<br>(0 to 0) | 0.0% fewer<br>(0 fewer to 0 fewer) | ⊕⊕◯◯<br>Low[a,d] | |
| All-cause mortality<br>(1 RCT) | OR 0.51<br>(0.35 to 0.77) | 8.3% | 4.4%<br>(3.1 to 6.6) | 3.9% fewer<br>(5.3 fewer to 1.8 fewer) | ⊕⊕◯◯<br>Low[a,c] | |

*The risk in the intervention group (and its 95% confidence interval) is based on the assumed risk in the comparison group and the relative effect of the intervention (and its 95% CI).

CI: confidence interval; OR: odds ratio

GRADE Working Group grades of evidence
High certainty: we are very confident that the true effect lies close to that of the estimate of the effect.
Moderate certainty: we are moderately confident in the effect estimate: the true effect is likely to be close to the estimate of the effect, but there is a possibility that it is substantially different.
Low certainty: our confidence in the effect estimate is limited: the true effect may be substantially different from the estimate of the effect.
Very low certainty: we have very little confidence in the effect estimate: the true effect is likely to be substantially different from the estimate of effect.

Explanations
a. Within-study bias
b. Heterogeneity
c. Incoherence
d. Imprecision

**5.2-3 NOACs vs. Placebo be used for treatment**

Summary of findings:

NOACs compared to Placebo for treatment

Patient or population: VTE
Setting:
Intervention: NOACs
Comparison: Placebo

| Outcome № of participants (studies) | Relative effect (95% CI) | Anticipated absolute effects (95% CI) | | | Certainty | What happens |
|---|---|---|---|---|---|---|
| | | Placebo | NOACs | Difference | | |
| Recurrent VTE (8 RCTs) | OR 0.23 (0.17 to 0.29) | 1.5% | 0.4% (0.3 to 0.5) | 1.2% fewer (1.3 fewer to 1.1 fewer) | ⊕⊕⊕◯ Moderate[a] | |
| Major bleeding (7 RCTs) | OR 2.2 (1.3 to 3.9) | 9.4% | 18.5% (11.8 to 28.7) | 9.2% more (2.5 more to 19.4 more) | ⊕⊕◯◯ Low[a,b] | |
| CRNMB (5 RCTs) | OR 1.7 (1.2 to 2.6) | 3.0% | 5.0% (3.6 to 7.5) | 2.0% more (0.6 more to 4.5 more) | ⊕⊕◯◯ Low[a,b] | |
| VTE related death (3 RCTs) | OR 0.64 (0.31 to 1.40) | 1.0% | 0.6% (0.3 to 1.4) | 0.4% fewer (0.7 fewer to 0.4 more) | ⊕⊕◯◯ Low[c,d] | |
| fatal bleeding (1 RCT) | OR 2.30 (0.34 to 23.00) | 0.0% | 0.0% (0 to 0) | 0.0% fewer (0 fewer to 0 fewer) | ⊕⊕◯◯ Low[a,c] | |
| adverse event (0 RCTs) | not estimable | 0.0% | 0.0% (0 to 0) | 0.0% fewer (0 fewer to 0 fewer) | ⊕◯◯◯ Very low[a,b,e] | |
| All-cause mortality (5 RCTs) | OR 0.49 (0.35 to 0.68) | 8.3% | 4.3% (3.1 to 5.8) | 4.1% fewer (5.3 fewer to 2.5 fewer) | ⊕⊕◯◯ Low[a,e] | |

*The risk in the intervention group (and its 95% confidence interval) is based on the assumed risk in the comparison group and the relative effect of the intervention (and its 95% CI).

CI: confidence interval; OR: odds ratio

GRADE Working Group grades of evidence
High certainty: we are very confident that the true effect lies close to that of the estimate of the effect.
Moderate certainty: we are moderately confident in the effect estimate: the true effect is likely to be close to the estimate of the effect, but there is a possibility that it is substantially different.
Low certainty: our confidence in the effect estimate is limited: the true effect may be substantially different from the estimate of the effect.
Very low certainty: we have very little confidence in the effect estimate: the true effect is likely to be substantially different from the estimate of effect.

Explanations
a. Within-study bias
b. Heterogeneity
c. Imprecision
d. Reporting bias
e. Incoherence

**5.2-4 TI vs. Placebo be used for treatment**

Summary of findings:

TI compared to Placebo for treatment

Patient or population: VTE
Setting:
Intervention: TI
Comparison: Placebo

| Outcome<br>№ of participants<br>(studies) | Relative effect<br>(95% CI) | Anticipated absolute effects (95% CI) | | | Certainty | What happens |
|---|---|---|---|---|---|---|
| | | Placebo | TI | Difference | | |
| Recurrent VTE<br>(1 RCT) | OR 0.22<br>(0.14 to 0.35) | 1.5% | 0.3%<br>(0.2 to 0.5) | 1.2% fewer<br>(1.3 fewer to 1 fewer) | ⊕⊕⊕⊕<br>High | |
| Major bleeding<br>(1 RCT) | OR 2.00<br>(0.97 to 4.50) | 9.4% | 17.1%<br>(9.1 to 31.7) | 7.8% more<br>(0.3 fewer to 22.4 more) | ⊕⊕◯◯<br>Low[a,b] | |
| CRNMB<br>(0 RCTs) | OR 1.6<br>(0.9 to 3.1) | 3.0% | 4.7%<br>(2.7 to 8.8) | 1.7% more<br>(0.3 fewer to 5.8 more) | ⊕⊕◯◯<br>Lowa,b | |
| VTE related death<br>(0 RCTs) | OR 0.200<br>(0.036 to 1.100) | 1.0% | 0.2%<br>(0 to 1.1) | 0.8% fewer<br>(0.9 fewer to 0.1 more) | ⊕⊕◯◯<br>Low[c,d] | |
| fatal bleeding<br>(0 RCTs) | OR 3.8<br>(0.2 to 80.0) | 0.0% | 0.0%<br>(0 to 0) | 0.0% fewer<br>(0 fewer to 0 fewer) | ⊕⊕◯◯<br>Low[a,c] | |
| adverse event<br>(0 RCTs) | not estimable | 0.0% | 0.0%<br>(0 to 0) | 0.0% fewer<br>(0 fewer to 0 fewer) | ⊕◯◯◯<br>Very low[a,b,c,e] | |
| All-cause mortality<br>(0 RCTs) | OR 0.49<br>(0.26 to 0.95) | 8.3% | 4.3%<br>(2.3 to 8) | 4.1% fewer<br>(6 fewer to 0.4 fewer) | ⊕⊕◯◯<br>Low[a,b] | |

*The risk in the intervention group (and its 95% confidence interval) is based on the assumed risk in the comparison group and the relative effect of the intervention (and its 95% CI).

CI: confidence interval; OR: odds ratio

GRADE Working Group grades of evidence
High certainty: we are very confident that the true effect lies close to that of the estimate of the effect.
Moderate certainty: we are moderately confident in the effect estimate: the true effect is likely to be close to the estimate of the effect, but there is a possibility that it is substantially different.
Low certainty: our confidence in the effect estimate is limited: the true effect may be substantially different from the estimate of the effect.
Very low certainty: we have very little confidence in the effect estimate: the true effect is likely to be substantially different from the estimate of effect.

Explanations
a. Within-study bias
b. Heterogeneity
c. Imprecision
d. Reporting bias
e. Incoherence

**5.2-5 UFH vs. Placebo be used for treatment**

Summary of findings:

UFH compared to Placebo for treatment

Patient or population: VTE
Setting:
Intervention: UFH
Comparison: Placebo

| Outcome<br>№ of participants<br>(studies) | Relative effect<br>(95% CI) | Anticipated absolute effects (95% CI) | | | Certainty | What happens |
|---|---|---|---|---|---|---|
| | | Placebo | UFH | Difference | | |
| Recurrent VTE<br>(0 RCTs) | OR 0.37<br>(0.24 to 0.56) | 1.5% | 0.6%<br>(0.4 to 0.9) | 1.0% fewer<br>(1.2 fewer to 0.7 fewer) | ⊕⊕⊕◯<br>Moderate[a] | |
| Major bleeding<br>(0 RCTs) | OR 2.5<br>(1.3 to 5.2) | 9.4% | 20.5%<br>(11.8 to 34.9) | 11.2% more<br>(2.5 more to 25.6 more) | ⊕⊕⊕◯<br>Moderate[a] | |
| CRNMB<br>(0 RCTs) | OR 1.2<br>(0.4 to 3.8) | 3.0% | 3.6%<br>(1.2 to 10.6) | 0.6% more<br>(1.8 fewer to 7.6 more) | ⊕◯◯◯<br>Very low[a,b,c] | |
| VTE related death<br>(0 RCTs) | OR 0.97<br>(0.18 to 5.90) | 1.0% | 1.0%<br>(0.2 to 5.5) | 0.0% fewer<br>(0.8 fewer to 4.6 more) | ⊕◯◯◯<br>Very low[a,b,d] | |
| fatal bleeding<br>(0 RCTs) | OR 0.5<br>(0.5 to 100.0) | 0.0% | 0.0%<br>(0 to 0) | 0.0% fewer<br>(0 fewer to 0 fewer) | ⊕⊕◯◯<br>Low[b,c] | |
| adverse event<br>(0 RCTs) | not estimable | 0.0% | 0.0%<br>(0 to 0) | 0.0% fewer<br>(0 fewer to 0 fewer) | ⊕◯◯◯<br>Very low[a,c] | |
| All-cause mortality<br>(0 RCTs) | OR 0.64<br>(0.4 to 1.00) | 8.3% | 5.5%<br>（3.5 to 8.3） | 2.8% fewer<br>(4.8 fewer to 0 fewer) | ⊕⊕◯◯<br>Low[a,c] | |

*The risk in the intervention group (and its 95% confidence interval) is based on the assumed risk in the comparison group and the relative effect of the intervention (and its 95% CI).

CI: confidence interval; OR: odds ratio

GRADE Working Group grades of evidence
High certainty: we are very confident that the true effect lies close to that of the estimate of the effect.
Moderate certainty: we are moderately confident in the effect estimate: the true effect is likely to be close to the estimate of the effect, but there is a possibility that it is substantially different.
Low certainty: our confidence in the effect estimate is limited: the true effect may be substantially different from the estimate of the effect.
Very low certainty: we have very little confidence in the effect estimate: the true effect is likely to be substantially different from the estimate of effect.

Explanations
a. Within-study bias
b. Imprecision
c. Heterogeneity
d. Reporting bias

**5.2-6 VKA vs. Placebo be used for treatment**

Summary of findings:

VKA compared to Placebo for treatment

Patient or population: VTE
Setting:
Intervention: VKA
Comparison: Placebo

| Outcome № of participants (studies) | Relative effect (95% CI) | Anticipated absolute effects (95% CI) | | | Certainty | What happens |
|---|---|---|---|---|---|---|
| | | Placebo | VKA | Difference | | |
| Recurrent VTE (1 RCT) | OR 0.30 (0.22 to 0.41) | 1.5% | 0.5% (0.3 to 0.6) | 1.1% fewer (1.2 fewer to 0.9 fewer) | ⊕⊕⊕◯ Moderate[a] | |
| Major bleeding (2 RCTs) | OR 2.9 (1.7 to 5.6) | 9.4% | 23.1% (14.9 to 36.7) | 13.7% more (5.6 more to 27.3 more) | ⊕⊕⊕◯ Moderate[a] | |
| CRNMB (0 RCTs) | OR 2.1 (1.4 to 3.5) | 3.0% | 6.1% (4.2 to 9.8) | 3.1% more (1.2 more to 6.8 more) | ⊕⊕⊕◯ Moderate[a] | |
| VTE related death (0 RCTs) | OR 0.49 (0.15 to 1.70) | 1.0% | 0.5% (0.1 to 1.7) | 0.5% fewer (0.8 fewer to 0.7 more) | ⊕⊕◯◯ Low[b,c] | |
| fatal bleeding (0 RCTs) | OR 7.70 (0.99 to 80.00) | 0.0% | 0.0% (0 to 0) | 0.0% fewer (0 fewer to 0 fewer) | ⊕⊕◯◯ Low[a,b] | |
| adverse event (0 RCTs) | not estimable | 0.0% | 0.0% (0 to 0) | 0.0% fewer (0 fewer to 0 fewer) | ⊕◯◯◯ Very low[a,b,d,e] | |
| All-cause mortality (1 RCT) | OR 0.51 (0.33 to 0.79) | 8.3% | 4.4% (2.9 to 6.7) | 3.9% fewer (5.4 fewer to 1.6 fewer) | ⊕⊕⊕◯ Moderate[a] | |

*The risk in the intervention group (and its 95% confidence interval) is based on the assumed risk in the comparison group and the relative effect of the intervention (and its 95% CI).

CI: confidence interval; OR: odds ratio

GRADE Working Group grades of evidence
High certainty: we are very confident that the true effect lies close to that of the estimate of the effect.
Moderate certainty: we are moderately confident in the effect estimate: the true effect is likely to be close to the estimate of the effect, but there is a possibility that it is substantially different.
Low certainty: our confidence in the effect estimate is limited: the true effect may be substantially different from the estimate of the effect.
Very low certainty: we have very little confidence in the effect estimate: the true effect is likely to be substantially different from the estimate of effect.

Explanations
a. Within-study bias
b. Imprecision
c. Reporting bias
d. Heterogeneity
e. Incoherence

**5.2-7 AP vs. Placebo be used for prevention**

Summary of findings:

| AP compared to Placebo for Prevention | | | | | | |
|---|---|---|---|---|---|---|
| Patient or population: PRE<br>Setting:<br>Intervention: AP<br>Comparison: Placebo | | | | | | |
| Outcome<br>№ of participants<br>(studies) | Relative effect<br>(95% CI) | Anticipated absolute effects (95% CI) | | | Certainty | What happens |
| | | Placebo | AP | Difference | | |
| Recurrent VTE<br>(3 RCTs) | OR 0.77<br>(0.47 to 1.30) | 4.3% | 3.3%<br>(2.1 to 5.5) | 0.9% fewer<br>(2.2 fewer to 1.2 more) | ⊕⊕◯◯<br>Low[a,b] | |
| Major bleeding<br>(3 RCTs) | OR 2.10<br>(0.98 to 4.90) | 0.9% | 2.0%<br>(0.9 to 4.5) | 1.0% more<br>(0 fewer to 3.5 more) | ⊕⊕⊕◯<br>Moderate[a] | |
| All-cause mortality<br>(1 RCT) | OR 7.3<br>(1.2 to 46.0) | 3.9% | 22.9%<br>(4.6 to 65.1) | 19.0% more<br>(0.7 more to 61.2 more) | ⊕⊕◯◯<br>Low[c,d] | |
| CRNMB<br>(1 RCT) | OR 1.30<br>(0.61 to 2.90) | 1.0% | 1.3%<br>(0.6 to 2.8) | 0.3% more<br>(0.4 fewer to 1.8 more) | ⊕⊕⊕◯<br>Moderate[d] | |
| VTE related death<br>(1 RCT) | OR 0.95<br>(0.08 to 11.00) | 0.5% | 0.5%<br>(0 to 5) | 0.0% fewer<br>(0.4 fewer to 4.5 more) | ⊕⊕⊕◯<br>Moderate[d] | |

*The risk in the intervention group (and its 95% confidence interval) is based on the assumed risk in the comparison group and the relative effect of the intervention (and its 95% CI).

CI: confidence interval; OR: odds ratio

GRADE Working Group grades of evidence
High certainty: we are very confident that the true effect lies close to that of the estimate of the effect.
Moderate certainty: we are moderately confident in the effect estimate: the true effect is likely to be close to the estimate of the effect, but there is a possibility that it is substantially different.
Low certainty: our confidence in the effect estimate is limited: the true effect may be substantially different from the estimate of the effect.
Very low certainty: we have very little confidence in the effect estimate: the true effect is likely to be substantially different from the estimate of effect.

Explanations
a. Heterogeneity
b. Reporting bias
c. Within-study bias
d. Imprecision

**5.2-8 LMWH vs. Placebo be used for prevention**

Summary of findings:

LMWH compared to Placebo for Prevention

Patient or population: pre
Setting:
Intervention: LMWH
Comparison: Placebo

| Outcome<br>№ of participants<br>(studies) | Relative effect<br>(95% CI) | Anticipated absolute effects (95% CI) | | | Certainty | What happens |
|---|---|---|---|---|---|---|
| | | Placebo | LMWH | Difference | | |
| Recurrent VTE<br>(31 RCTs) | OR 0.48<br>(0.40 to 0.56) | 4.3% | 2.1%<br>(1.8 to 2.4) | 2.2% fewer<br>(2.5 fewer to 1.8 fewer) | ⊕◯◯◯<br>Very low[a,b,c] | |
| Major bleeding<br>(31 RCTs) | OR 1.30<br>(0.93 to 1.80) | 0.9% | 1.2%<br>(0.9 to 1.7) | 0.3% more<br>(0.1 fewer to 0.7 more) | ⊕⊕⊕◯<br>Moderatea | |
| All-cause mortality<br>(6 RCTs) | OR 5.4<br>(2.2 to 13.0) | 3.9% | 18.0%<br>(8.2 to 34.6) | 14.1% more<br>(4.3 more to 30.7 more) | ⊕⊕⊕⊕<br>High | |
| CRNMB<br>(2 RCTs) | OR 1.10<br>(0.74 to 1.60) | 1.0% | 1.1%<br>(0.7 to 1.5) | 0.1% more<br>(0.2 fewer to 0.6 more) | ⊕⊕⊕⊕<br>High | |
| VTE related death<br>(3 RCTs) | OR 0.71<br>(0.28 to 1.60) | 0.5% | 0.3%<br>(0.1 to 0.8) | 0.1% fewer<br>(0.3 fewer to 0.3 more) | ⊕⊕⊕◯<br>Moderate[d] | |

*The risk in the intervention group (and its 95% confidence interval) is based on the assumed risk in the comparison group and the relative effect of the intervention (and its 95% CI).

CI: confidence interval; OR: odds ratio

GRADE Working Group grades of evidence
High certainty: we are very confident that the true effect lies close to that of the estimate of the effect.
Moderate certainty: we are moderately confident in the effect estimate: the true effect is likely to be close to the estimate of the effect, but there is a possibility that it is substantially different.
Low certainty: our confidence in the effect estimate is limited: the true effect may be substantially different from the estimate of the effect.
Very low certainty: we have very little confidence in the effect estimate: the true effect is likely to be substantially different from the estimate of effect.

Explanations
a. Within-study bias
b. Heterogeneity
c. Incoherence
d. Imprecision

**5.2-9 NOACs vs. Placebo be used for prevention**

Summary of findings:

NOACs compared to Placebo for Prevention

Patient or population: pre
Setting:
Intervention: NOACs
Comparison: Placebo

| Outcome<br>№ of participants<br>(studies) | Relative effect<br>(95% CI) | Anticipated absolute effects (95% CI) | | | Certainty | What happens |
|---|---|---|---|---|---|---|
| | | Placebo | NOACs | Difference | | |
| VTE<br>(31 RCTs) | OR 0.32<br>(0.26 to 0.38) | 4.3% | 1.4%<br>(1.1 to 1.7) | 2.9% fewer<br>(3.1 fewer to 2.6 fewer) | ⊕◯◯◯<br>Low[a,b,c] | |
| Major bleeding<br>(31 RCTs) | OR 1.8<br>(1.2 to 2.6) | 0.9% | 1.7%<br>(1.1 to 2.4) | 0.7% more<br>(0.2 more to 1.5 more) | ⊕⊕◯◯<br>Low[a,c] | |
| All-cause mortality<br>(11 RCTs) | OR 3.0<br>(1.3 to 7.1) | 3.9% | 10.9%<br>(5 to 22.4) | 7.0% more<br>(1.1 more to 18.5 more) | ⊕⊕⊕⊕<br>High | |
| CRNMB<br>(14 RCTs) | OR 1.30<br>(0.93 to 1.90) | 1.0% | 1.3%<br>(0.9 to 1.8) | 0.3% more<br>(0.1 fewer to 0.9 more) | ⊕⊕⊕◯<br>Moderate[a] | |
| VTE related death<br>(4 RCTs) | OR 0.73<br>(0.31 to 1.30) | 0.5% | 0.3%<br>(0.1 to 0.6) | 0.1% fewer<br>(0.3 fewer to 0.1 more) | ⊕⊕⊕⊕<br>High | |

*The risk in the intervention group (and its 95% confidence interval) is based on the assumed risk in the comparison group and the relative effect of the intervention (and its 95% CI).

CI: confidence interval; OR: odds ratio

GRADE Working Group grades of evidence
High certainty: we are very confident that the true effect lies close to that of the estimate of the effect.
Moderate certainty: we are moderately confident in the effect estimate: the true effect is likely to be close to the estimate of the effect, but there is a possibility that it is substantially different.
Low certainty: our confidence in the effect estimate is limited: the true effect may be substantially different from the estimate of the effect.
Very low certainty: we have very little confidence in the effect estimate: the true effect is likely to be substantially different from the estimate of effect.

Explanations
a. Heterogeneity
b. Reporting bias
c. Within-study bias

**5.2-10 TI vs. Placebo be used for prevention**

Summary of findings:

TI compared to Placebo for Prevention

Patient or population: PRE
Setting:
Intervention: TI
Comparison: Placebo

| Outcome № of participants (studies) | Relative effect (95% CI) | Anticipated absolute effects (95% CI) | | | Certainty | What happens |
|---|---|---|---|---|---|---|
| | | Placebo | TI | Difference | | |
| VTE (0 RCTs) | OR 0.46 (0.35 to 0.60) | 4.3% | 2.0% (1.5 to 2.6) | 2.3% fewer (2.7 fewer to 1.7 fewer) | ⊕◯◯◯ Very low[a,b,c,d] | |
| Major bleeding (0 RCTs) | OR 1.5 (0.9 to 2.4) | 0.9% | 1.4% (0.9 to 2.2) | 0.5% more (0.1 fewer to 1.3 more) | ⊕⊕◯◯ Low[a,b] | |
| All-cause mortality (0 RCTs) | OR 1.40 (0.27 to 7.60) | 3.9% | 5.4% (1.1 to 23.6) | 1.5% more (2.8 fewer to 19.7 more) | ⊕⊕⊕◯ Moderate[e] | |
| CRNMB (0 RCTs) | OR 1.30 (0.79 to 2.10) | 1.0% | 1.3% (0.8 to 2) | 0.3% more (0.2 fewer to 1 more) | ⊕⊕⊕◯ Moderate[b] | |
| VTE related death (0 RCTs) | OR 1.10 (0.14 to 9.20) | 0.5% | 0.5% (0.1 to 4.2) | 0.0% fewer (0.4 fewer to 3.7 more) | ⊕⊕⊕◯ Moderate[e] | |

*The risk in the intervention group (and its 95% confidence interval) is based on the assumed risk in the comparison group and the relative effect of the intervention (and its 95% CI).

CI: confidence interval; OR: odds ratio

GRADE Working Group grades of evidence
High certainty: we are very confident that the true effect lies close to that of the estimate of the effect.
Moderate certainty: we are moderately confident in the effect estimate: the true effect is likely to be close to the estimate of the effect, but there is a possibility that it is substantially different.
Low certainty: our confidence in the effect estimate is limited: the true effect may be substantially different from the estimate of the effect.
Very low certainty: we have very little confidence in the effect estimate: the true effect is likely to be substantially different from the estimate of effect.

Explanations
a. Within-study bias
b. Heterogeneity
c. Incoherence
d. Reporting bias
e. Imprecision

**5.2-11 UFH vs. Placebo be used for prevention**

Summary of findings:

UFH compared to Placebo for Prevention

Patient or population: PRE
Setting:
Intervention: UFH
Comparison: Placebo

| Outcome<br>№ of participants<br>(studies) | Relative effect<br>(95% CI) | Anticipated absolute effects (95% CI) | | | Certainty | What happens |
|---|---|---|---|---|---|---|
| | | Placebo | UFH | Difference | | |
| VTE<br>(0 RCTs) | OR 0.60<br>(0.44 to 0.82) | 4.3% | 2.6%<br>(1.9 to 3.5) | 1.7% fewer<br>(2.3 fewer to 0.7 fewer) | ⊕◯◯◯<br>Very low[a,b,c,d] | |
| Major bleeding<br>(0 RCTs) | OR 2.0<br>(1.2 to 3.3) | 0.9% | 1.9%<br>(1.1 to 3) | 0.9% more<br>(0.2 more to 2.1 more) | ⊕⊕◯◯<br>Low[a,b] | |
| All-cause mortality<br>(0 RCTs) | OR 3.60<br>(0.59 to 22.00) | 3.9% | 12.8%<br>(2.3 to 47.2) | 8.9% more<br>(1.6 fewer to 43.3 more) | ⊕⊕⊕◯<br>Moderate[a] | |
| VTE related death<br>(0 RCTs) | OR 1.5<br>(0.1 to 43.0) | 0.5% | 0.7%<br>(0 to 17.1) | 0.2% more<br>(0.4 fewer to 16.6 more) | ⊕⊕⊕◯<br>Moderate[e] | |

*The risk in the intervention group (and its 95% confidence interval) is based on the assumed risk in the comparison group and the relative effect of the intervention (and its 95% CI).

CI: confidence interval; OR: odds ratio

GRADE Working Group grades of evidence
High certainty: we are very confident that the true effect lies close to that of the estimate of the effect.
Moderate certainty: we are moderately confident in the effect estimate: the true effect is likely to be close to the estimate of the effect, but there is a possibility that it is substantially different.
Low certainty: our confidence in the effect estimate is limited: the true effect may be substantially different from the estimate of the effect.
Very low certainty: we have very little confidence in the effect estimate: the true effect is likely to be substantially different from the estimate of effect.

Explanations
a. Within-study bias
b. Heterogeneity
c. Incoherence
d. Reporting bias
e. Imprecision

**5.2-12 VKA vs. Placebo be used for prevention**

Summary of findings:

VKA compared to Placebo for Prevention

Patient or population: PRE
Setting:
Intervention: VKA
Comparison: Placebo

| Outcome № of participants (studies) | Relative effect (95% CI) | Anticipated absolute effects (95% CI) | | | Certainty | What happens |
|---|---|---|---|---|---|---|
| | | Placebo | VKA | Difference | | |
| VTE (1 RCT) | OR 0.80 (0.61 to 1.10) | 4.3% | 3.4% (2.6 to 4.7) | 0.8% fewer (1.6 fewer to 0.4 more) | ⨁◯◯◯ Very low[a,b,c] | |
| Major bleeding (1 RCT) | OR 0.84 (0.49 to 1.40) | 0.9% | 0.8% (0.5 to 1.3) | 0.1% fewer (0.5 fewer to 0.4 more) | ⨁⨁⨁◯ Moderate[a] | |
| All-cause mortality (0 RCTs) | OR 8.9 (2.6 to 31.0) | 3.9% | 26.6% (9.6 to 55.7) | 22.7% more (5.7 more to 51.8 more) | ⨁⨁⨁◯ Moderate[d] | |
| VTE related death (0 RCTs) | OR 2.6 (0.2 to 65.0) | 0.5% | 1.2% (0.1 to 23.8) | 0.8% more (0.4 fewer to 23.3 more) | ⨁⨁⨁◯ Moderate[d] | |

*The risk in the intervention group (and its 95% confidence interval) is based on the assumed risk in the comparison group and the relative effect of the intervention (and its 95% CI).

CI: confidence interval; OR: odds ratio

GRADE Working Group grades of evidence
High certainty: we are very confident that the true effect lies close to that of the estimate of the effect.
Moderate certainty: we are moderately confident in the effect estimate: the true effect is likely to be close to the estimate of the effect, but there is a possibility that it is substantially different.
Low certainty: our confidence in the effect estimate is limited: the true effect may be substantially different from the estimate of the effect.
Very low certainty: we have very little confidence in the effect estimate: the true effect is likely to be substantially different from the estimate of effect.

Explanations
a. Within-study bias
b. Incoherence
c. Reporting bias
d. Imprecision

| Comparison | Grade level | Reason(s) for downgrading |
|---|---|---|
| **Recurrent VTE during treatment** | | |
| AP: NOACs | moderate | ["Within-study bias"] |
| AP: Placebo | high | [] |
| LMWH: NOACs | moderate | ["Within-study bias"] |
| LMWH: Placebo | moderate | ["Within-study bias"] |
| LMWH: UFH | moderate | ["Within-study bias"] |
| LMWH: VKA | moderate | ["Within-study bias"] |
| NOACs: Placebo | moderate | ["Within-study bias"] |
| NOACs: VKA | moderate | ["Within-study bias"] |
| Placebo: TI | high | [] |
| Placebo: VKA | moderate | ["Within-study bias"] |
| TI: VKA | high | [] |
| UFH: VKA | moderate | ["Within-study bias"] |
| AP: LMWH | moderate | ["Within-study bias"] |
| AP: TI | moderate | ["Within-study bias"] |
| AP: UFH | moderate | ["Within-study bias"] |
| AP: VKA | moderate | ["Within-study bias"] |
| LMWH: TI | moderate | ["Within-study bias"] |
| NOACs: TI | moderate | ["Within-study bias"] |
| NOACs: UFH | moderate | ["Within-study bias"] |
| Placebo: UFH | moderate | ["Within-study bias"] |
| TI: UFH | moderate | ["Within-study bias"] |
| **Major bleeding during treatment** | | |
| AP: NOACs | low | ["Within-study bias","Imprecision"] |
| AP: Placebo | moderate | ["Within-study bias"] |
| LMWH: NOACs | moderate | ["Within-study bias"] |
| LMWH: Placebo | very low | ["Within-study bias","Heterogeneity","Incoherence"] |
| LMWH: UFH | low | ["Within-study bias","Incoherence"] |
| LMWH: VKA | moderate | ["Within-study bias"] |
| NOACs: Placebo | low | ["Within-study bias","Heterogeneity"] |
| NOACs: VKA | moderate | ["Within-study bias"] |
| Placebo: TI | low | ["Within-study bias","Heterogeneity"] |
| Placebo: VKA | moderate | ["Within-study bias"] |
| TI: VKA | high | [] |
| UFH: VKA | low | ["Within-study bias","Incoherence"] |
| AP: LMWH | moderate | ["Within-study bias"] |
| AP: TI | moderate | ["Within-study bias"] |
| AP: UFH | moderate | ["Within-study bias"] |
| AP: VKA | moderate | ["Within-study bias"] |
| LMWH: TI | moderate | ["Within-study bias"] |
| NOACs: TI | moderate | ["Within-study bias"] |
| NOACs: UFH | moderate | ["Within-study bias"] |
| Placebo: UFH | moderate | ["Within-study bias"] |
| TI: UFH | moderate | ["Within-study bias"] |
| **Clinical relevant non-major bleeding during treatment** | | |
| AP: NOACs | moderate | ["Within-study bias"] |

| | | |
|---|---|---|
| AP: Placebo | low | ["Within-study bias","Heterogeneity"] |
| LMWH: NOACs | very low | ["Within-study bias","Heterogeneity","Incoherence"] |
| LMWH: Placebo | low | ["Within-study bias","Incoherence"] |
| LMWH: UFH | very low | ["Within-study bias","Imprecision","Heterogeneity"] |
| NOACs: Placebo | low | ["Within-study bias","Heterogeneity"] |
| NOACs: VKA | moderate | ["Within-study bias"] |
| TI: VKA | high | [] |
| AP: LMWH | low | ["Within-study bias","Heterogeneity"] |
| AP: TI | moderate | ["Within-study bias"] |
| AP: UFH | very low | ["Within-study bias","Imprecision","Heterogeneity"] |
| AP: VKA | low | ["Within-study bias","Heterogeneity"] |
| LMWH: TI | low | ["Within-study bias","Heterogeneity"] |
| LMWH: VKA | moderate | ["Within-study bias"] |
| NOACs: TI | moderate | ["Within-study bias"] |
| NOACs: UFH | low | ["Within-study bias","Imprecision"] |
| Placebo: TI | low | ["Within-study bias","Heterogeneity"] |
| Placebo: UFH | very low | ["Within-study bias","Imprecision","Heterogeneity"] |
| Placebo: VKA | moderate | ["Within-study bias"] |
| TI: UFH | very low | ["Within-study bias","Imprecision","Heterogeneity"] |
| UFH: VKA | low | ["Within-study bias","Imprecision"] |
| VTE related death during treatment | | |
| AP: NOACs | very low | ["Within-study bias","Reporting bias","Imprecision"] |
| AP: Placebo | low | ["Reporting bias","Imprecision"] |
| LMWH: NOACs | very low | ["Within-study bias","Reporting bias","Imprecision","Heterogeneity"] |
| LMWH: Placebo | very low | ["Within-study bias","Reporting bias","Imprecision"] |
| LMWH: UFH | very low | ["Within-study bias","Reporting bias","Imprecision"] |
| LMWH: VKA | very low | ["Within-study bias","Reporting bias","Imprecision"] |
| NOACs: Placebo | low | ["Reporting bias","Imprecision"] |
| NOACs: VKA | low | ["Reporting bias","Imprecision"] |
| TI: VKA | low | ["Reporting bias","Imprecision"] |
| AP: LMWH | very low | ["Within-study bias","Reporting bias","Imprecision"] |
| AP: TI | low | ["Reporting bias","Imprecision"] |
| AP: UFH | very low | ["Within-study bias","Reporting bias","Imprecision"] |
| AP: VKA | very low | ["Reporting bias","Imprecision","Heterogeneity"] |
| LMWH: TI | low | ["Reporting bias","Imprecision"] |
| NOACs: TI | low | ["Reporting bias","Imprecision"] |
| NOACs: UFH | very low | ["Within-study bias","Reporting bias","Imprecision"] |
| Placebo: TI | low | ["Reporting bias","Heterogeneity"] |
| Placebo: UFH | very low | ["Within-study bias","Reporting bias","Imprecision"] |
| Placebo: VKA | low | ["Reporting bias","Imprecision"] |
| TI: UFH | very low | ["Within-study bias","Reporting bias","Imprecision"] |
| UFH: VKA | very low | ["Within-study bias","Reporting bias","Imprecision"] |
| Fatal bleeding during treatment | | |
| AP: NOACs | low | ["Within-study bias","Imprecision"] |
| LMWH: NOACs | low | ["Imprecision","Heterogeneity"] |
| LMWH: Placebo | low | ["Imprecision","Heterogeneity"] |
| LMWH: UFH | moderate | ["Imprecision"] |

| | | |
|---|---|---|
| **LMWH: VKA** | **very low** | **["Within-study bias","Imprecision","Heterogeneity"]** |
| **NOACs: Placebo** | **low** | **["Within-study bias","Imprecision"]** |
| **NOACs: VKA** | **moderate** | **["Within-study bias"]** |
| **TI: VKA** | **low** | **["Within-study bias","Imprecision"]** |
| **AP: LMWH** | **low** | **["Within-study bias","Imprecision"]** |
| **AP: Placebo** | **low** | **["Within-study bias","Imprecision"]** |
| **AP: TI** | **low** | **["Within-study bias","Imprecision"]** |
| **AP: UFH** | **low** | **["Within-study bias","Imprecision"]** |
| **AP: VKA** | **low** | **["Within-study bias","Imprecision"]** |
| **LMWH: TI** | **low** | **["Within-study bias","Imprecision"]** |
| **NOACs: TI** | **low** | **["Within-study bias","Imprecision"]** |
| **NOACs: UFH** | **low** | **["Imprecision","Heterogeneity"]** |
| **Placebo: TI** | **low** | **["Within-study bias","Imprecision"]** |
| **Placebo: UFH** | **low** | **["Imprecision","Heterogeneity"]** |
| **Placebo: VKA** | **low** | **["Within-study bias","Imprecision"]** |
| **TI: UFH** | **low** | **["Within-study bias","Imprecision"]** |
| **UFH: VKA** | **low** | **["Within-study bias","Imprecision"]** |
| **Adverse events during treatment** | | |
| **LMWH: NOACs** | **low** | **["Within-study bias","Incoherence"]** |
| **LMWH: Placebo** | **very low** | **["Within-study bias","Imprecision","Heterogeneity","Incoherence"]** |
| **NOACs: VKA** | **low** | **["Within-study bias","Incoherence"]** |
| **TI: VKA** | **moderate** | **["Incoherence"]** |
| **LMWH: TI** | **low** | **["Within-study bias","Incoherence"]** |
| **LMWH: VKA** | **low** | **["Within-study bias","Incoherence"]** |
| **NOACs: Placebo** | **very low** | **["Within-study bias","Imprecision","Heterogeneity","Incoherence"]** |
| **NOACs: TI** | **low** | **["Within-study bias","Incoherence"]** |
| **Placebo: TI** | **very low** | **["Within-study bias","Imprecision","Heterogeneity","Incoherence"]** |
| **Placebo: VKA** | **very low** | **["Within-study bias","Imprecision","Heterogeneity","Incoherence"]** |
| **All-cause mortality during treatment** | | |
| **AP: NOACs** | **low** | **["Within-study bias","Imprecision"]** |
| **AP: Placebo** | **moderate** | **["Heterogeneity"]** |
| **LMWH: NOACs** | **moderate** | **["Within-study bias"]** |
| **LMWH: Placebo** | **low** | **["Within-study bias","Incoherence"]** |
| **LMWH: UFH** | **moderate** | **["Within-study bias"]** |
| **LMWH: VKA** | **moderate** | **["Within-study bias"]** |
| **NOACs: Placebo** | **low** | **["Within-study bias","Incoherence"]** |
| **NOACs: VKA** | **moderate** | **["Within-study bias"]** |
| **Placebo: VKA** | **moderate** | **["Within-study bias"]** |
| **TI: VKA** | **high** | **[]** |
| **AP: LMWH** | **low** | **["Within-study bias","Imprecision"]** |
| **AP: TI** | **low** | **["Within-study bias","Imprecision"]** |
| **AP: UFH** | **low** | **["Within-study bias","Imprecision"]** |
| **AP: VKA** | **low** | **["Within-study bias","Imprecision"]** |
| **LMWH: TI** | **moderate** | **["Within-study bias"]** |
| **NOACs: TI** | **moderate** | **["Within-study bias"]** |
| **NOACs: UFH** | **moderate** | **["Within-study bias"]** |
| **Placebo: TI** | **low** | **["Within-study bias","Heterogeneity"]** |

| | | |
|---|---|---|
| **Placebo: UFH** | **low** | **["Within-study bias","Heterogeneity"]** |
| **TI: UFH** | **low** | **["Within-study bias","Heterogeneity"]** |
| **UFH: VKA** | **moderate** | **["Within-study bias"]** |
| **VTE during prophylaxis** | | |
| **AP: LMWH** | **very low** | **["Within-study bias","Reporting bias","Heterogeneity"]** |
| **AP: NOACs** | **very low** | **["Within-study bias","Reporting bias","Heterogeneity"]** |
| **AP: Placebo** | **low** | **["Reporting bias","Heterogeneity"]** |
| **AP: VKA** | **very low** | **["Within-study bias","Reporting bias","Heterogeneity","Incoherence"]** |
| **LMWH: NOACs** | **very low** | **["Within-study bias","Reporting bias","Heterogeneity"]** |
| **LMWH: Placebo** | **very low** | **["Within-study bias","Reporting bias","Heterogeneity","Incoherence"]** |
| **LMWH: TI** | **low** | **["Within-study bias","Reporting bias"]** |
| **LMWH: UFH** | **very low** | **["Within-study bias","Reporting bias","Heterogeneity","Incoherence"]** |
| **LMWH: VKA** | **very low** | **["Within-study bias","Reporting bias","Heterogeneity"]** |
| **NOACs: Placebo** | **very low** | **["Within-study bias","Reporting bias","Incoherence"]** |
| **NOACs: TI** | **very low** | **["Within-study bias","Reporting bias","Heterogeneity","Incoherence"]** |
| **NOACs: VKA** | **low** | **["Within-study bias","Reporting bias"]** |
| **Placebo: VKA** | **very low** | **["Within-study bias","Reporting bias","Incoherence"]** |
| **TI: VKA** | **very low** | **["Within-study bias","Reporting bias","Heterogeneity"]** |
| **AP: TI** | **very low** | **["Within-study bias","Reporting bias","Heterogeneity","Incoherence"]** |
| **AP: UFH** | **very low** | **["Within-study bias","Reporting bias","Incoherence"]** |
| **NOACs: UFH** | **very low** | **["Within-study bias","Reporting bias","Heterogeneity","Incoherence"]** |
| **Placebo: TI** | **very low** | **["Within-study bias","Reporting bias","Heterogeneity","Incoherence"]** |
| **Placebo: UFH** | **very low** | **["Within-study bias","Reporting bias","Heterogeneity","Incoherence"]** |
| **TI: UFH** | **very low** | **["Within-study bias","Reporting bias","Heterogeneity","Incoherence"]** |
| **UFH: VKA** | **very low** | **["Within-study bias","Reporting bias","Heterogeneity","Incoherence"]** |
| **Major bleeding during prophylaxis** | | |
| **AP: LMWH** | **moderate** | **["Heterogeneity"]** |
| **AP: NOACs** | **high** | **[]** |
| **AP: Placebo** | **moderate** | **["Heterogeneity"]** |
| **AP: VKA** | **moderate** | **["Imprecision"]** |
| **LMWH: NOACs** | **moderate** | **["Within-study bias"]** |
| **LMWH: Placebo** | **moderate** | **["Within-study bias"]** |
| **LMWH: TI** | **high** | **[]** |
| **LMWH: UFH** | **moderate** | **["Within-study bias"]** |
| **LMWH: VKA** | **moderate** | **["Within-study bias"]** |
| **NOACs: Placebo** | **low** | **["Within-study bias","Heterogeneity"]** |
| **NOACs: TI** | **moderate** | **["Within-study bias"]** |
| **NOACs: VKA** | **low** | **["Within-study bias","Incoherence"]** |
| **Placebo: VKA** | **moderate** | **["Within-study bias"]** |
| **TI: VKA** | **low** | **["Within-study bias","Heterogeneity"]** |
| **AP: TI** | **high** | **[]** |
| **AP: UFH** | **moderate** | **["Within-study bias"]** |
| **NOACs: UFH** | **moderate** | **["Within-study bias"]** |
| **Placebo: TI** | **low** | **["Within-study bias","Heterogeneity"]** |
| **Placebo: UFH** | **low** | **["Within-study bias","Heterogeneity"]** |
| **TI: UFH** | **high** | **[]** |
| **UFH: VKA** | **moderate** | **["Within-study bias","Heterogeneity"]** |

| | | |
|---|---|---|
| **All-cause mortality during prophylaxis** | | |
| **AP: LMWH** | **low** | **["Within-study bias","Imprecision"]** |
| **AP: Placebo** | **low** | **["Within-study bias","Imprecision"]** |
| **AP: VKA** | **low** | **["Within-study bias","Imprecision"]** |
| **LMWH: NOACs** | **high** | **[]** |
| **LMWH: Placebo** | **high** | **[]** |
| **LMWH: TI** | **moderate** | **["Imprecision"]** |
| **LMWH: UFH** | **moderate** | **["Within-study bias"]** |
| **LMWH: VKA** | **moderate** | **["Imprecision"]** |
| **NOACs: Placebo** | **high** | **[]** |
| **NOACs: VKA** | **moderate** | **["Imprecision"]** |
| **TI: VKA** | **low** | **["Within-study bias","Imprecision"]** |
| **AP: NOACs** | **low** | **["Within-study bias","Imprecision"]** |
| **AP: TI** | **low** | **["Within-study bias","Imprecision"]** |
| **AP: UFH** | **low** | **["Within-study bias","Imprecision"]** |
| **NOACs: TI** | **moderate** | **["Imprecision"]** |
| **NOACs: UFH** | **high** | **[]** |
| **Placebo: TI** | **moderate** | **["Imprecision"]** |
| **Placebo: UFH** | **moderate** | **["Within-study bias"]** |
| **Placebo: VKA** | **moderate** | **["Imprecision"]** |
| **TI: UFH** | **low** | **["Within-study bias","Imprecision"]** |
| **UFH: VKA** | **low** | **["Within-study bias","Imprecision"]** |
| **Clinical relevant non-major bleeding during prophylaxis** | | |
| **AP: Placebo** | **moderate** | **["Imprecision"]** |
| **LMWH: NOACs** | **high** | **[]** |
| **LMWH: Placebo** | **high** | **[]** |
| **LMWH: TI** | **high** | **[]** |
| **NOACs: Placebo** | **moderate** | **["Heterogeneity"]** |
| **AP: LMWH** | **moderate** | **["Imprecision"]** |
| **AP: NOACs** | **moderate** | **["Imprecision"]** |
| **AP: TI** | **moderate** | **["Imprecision"]** |
| **NOACs: TI** | **high** | **[]** |
| **Placebo: TI** | **moderate** | **["Heterogeneity"]** |
| **VTE related death during prophylaxis** | | |
| **AP: Placebo** | **moderate** | **["Imprecision"]** |
| **LMWH: NOACs** | **moderate** | **["Imprecision"]** |
| **LMWH: Placebo** | **moderate** | **["Imprecision"]** |
| **LMWH: TI** | **moderate** | **["Imprecision"]** |
| **LMWH: UFH** | **moderate** | **["Imprecision"]** |
| **LMWH: VKA** | **low** | **["Within-study bias","Imprecision"]** |
| **NOACs: Placebo** | **high** | **[]** |
| **NOACs: TI** | **low** | **["Within-study bias","Imprecision"]** |
| **AP: LMWH** | **moderate** | **["Imprecision"]** |
| **AP: NOACs** | **moderate** | **["Imprecision"]** |
| **AP: TI** | **moderate** | **["Imprecision"]** |
| **AP: UFH** | **moderate** | **["Imprecision"]** |
| **AP: VKA** | **moderate** | **["Imprecision"]** |

| | | |
|---|---|---|
| **NOACs: UFH** | **moderate** | **["Imprecision"]** |
| **NOACs: VKA** | **moderate** | **["Imprecision"]** |
| **Placebo: TI** | **moderate** | **["Imprecision"]** |
| **Placebo: UFH** | **moderate** | **["Imprecision"]** |
| **Placebo: VKA** | **moderate** | **["Imprecision"]** |
| **TI: UFH** | **moderate** | **["Imprecision"]** |
| **TI: VKA** | **low** | **["Within-study bias","Imprecision"]** |
| **UFH: VKA** | **low** | **["Within-study bias","Imprecision"]** |

### 5.3 Minimally contextualized framework

**5.31 Treatment**

| Certainty of the evidence, and classification of intervention | Anticoagulant | Anticoagulant vs Placebo (OR(95%Confidence interval）） | Surface under the cumulative ranking curve |
|---|---|---|---|
| **Recurrent VTE (odds ratio; 95% confidence interval)** | | | |
| **High certainty (moderate to high certainty evidence)** | | | |
| Category2：  among the most beneficial | TI | 0.22（0.14，0.35） | 1 |
| | NOACs | 0.23（0.17，0.29） | 2 |
| | LMWH | 0.25（0.18，0.35） | 3 |
| | VKA | 0.30（0.22，0.41） | 4 |
| | UFH | 0.37（0.24，0.56） | 5 |
| Category1：inferior to the most beneficial or superior to the least beneficial | AP | 0.67（0.46，0.96） | 6 |
| | | | |
| **Major bleeding (odds ratio; 95% confidence interval)** | | | |
| **High certainty (moderate to high certainty evidence)** | | | |
| Category1：inferior to the most harmful or superior to the least harmful | VKA | 2.9（1.7，5.6） | 1 |
| | UFH | 2.5（1.3，5.2） | 2 |
| Category0：among the least harmful | AP | 1.4（0.59，3.3） | 6 |
| **Low certainty (low to very low certainty evidence)** | | | |
| Category 1: might be inferior to the most harmful or superior to the least harmful | LMWH | 2.2（1.2，4.2） | 3 |
| | NOACs | 2.2（1.3，3.9） | 4 |
| Category 0: might be among the least harmful | TI | 2.0（0.97，4.5） | 5 |
| | | | |
| **Clinical relevant non-major bleeding (odds ratio; 95% confidence interval)** | | | |
| **High certainty (moderate to high certainty evidence)** | | | |
| Category1：inferior to the most harmful or superior to the least harmful | VKA | 2.1（1.4，3.5） | 1 |
| **Low certainty (low to very low certainty evidence)** | | | |
| Category 1: might be inferior to the most harmful or superior to the least harmful | NOACs | 1.7（1.2，2.6） | 2 |
| Category 0: might be among the least harmful | TI | 1.6（0.9，3.1） | 3 |

| | | | |
|---|---|---|---|
| | AP | 1.3（0.74，2.5） | 4 |
| | UFH | 1.2（0.40，3.8） | 5 |
| | LMWH | 1.1（0.66，1.9） | 6 |
| | | | |
| **VTE related Death (odds ratio; 95% confidence interval)** | | | |
| **High certainty (moderate to high certainty evidence)** | | | |
| Category0：among the least beneficial | AP | 0.78(0.25,2.5) | 5 |
| **Low certainty (low to very low certainty evidence)** | | | |
| Category 0: might be among the least beneficial | TI | 0.20(0.036,1.1) | 1 |
| | VAK | 0.49(0.15,1.7) | 2 |
| | NOACs | 0.64(0.31,1.4) | 3 |
| | LMWH | 0.75(0.21,3.0) | 4 |
| | UFH | 0.97(0.18,5.9) | 6 |
| | | | |
| **Fatal bleeding (odds ratio; 95% confidence interval)** | | | |
| **High certainty (moderate to high certainty evidence)** | | | |
| Category0：among the least harmful | LMWH | 3.5(0.56,35.0) | 4 |
| **Low certainty (low to very low certainty evidence)** | | | |
| Category 0: might be among the least harmful | VKA | 7.7(0.99,80.0) | 1 |
| | UFH | 5.8(0.50,1.0E+02) | 2 |
| | TI | 3.8(0.20,80.0) | 3 |
| | AP | 3.2(0.26,52.0) | 5 |
| | NOACs | 2.3(0.34,23.0) | 6 |
| | | | |
| **Adverse events (odds ratio; 95% confidence interval)** | | | |
| **Low certainty (low to very low certainty evidence)** | | | |
| Category 0: might be among the least harmful | VKA | 8.2(0.90,2.3E+02) | 1 |
| | LMWH | 7.6(0.92,2.1E+02) | 2 |
| | TI | 7.3(0.78,2.1E+02) | 3 |
| | NAOCs | 7.1(0.80,1.9E+02) | 4 |

| | | | |
|---|---|---|---|
| **All-cause mortality (odds ratio; 95% confidence interval)** | | | |
| **High certainty (moderate to high certainty evidence)** | | | |
| Category1：inferior to the most beneficial or superior to the least beneficial | VKA | 0.51(0.33,0.79) | 4 |
| Category0：among the least beneficial | AP | 0.83(0.47,1.5) | 6 |
| **Low certainty (low to very low certainty evidence)** | | | |
| Category 1: might be inferior to the most beneficial or superior to the least beneficial | TI | 0.49(0.26,0.95) | 1 |
| | NOACs | 0.49(0.35,0.68) | 2 |
| | LMWH | 0.51(0.35,0.77) | 3 |
| Category 0: might be among the least beneficial | UFH | 0.64(0.40,1.0) | 5 |
| | Placebo | Reference | - |

**5.32 Prevention**

| Certainty of the evidence, and classification of intervention | Anticoagulant | Anticoagulant vs Placebo (OR(95%Credible interval）） | Surface under the cumulative ranking curve |
|---|---|---|---|
| **VTE (odds ratio; 95% confidence interval)** | | | |
| **Low certainty (low to very low certainty evidence)** | | | |
| Category 2: might be among the most effective | NOACs | 0.32(0.26,0.38) | 1 |
| Category 1: might be inferior to the most effective or superior to the least effective | TI | 0.46(0.35,0.60) | 2 |
| | LMWH | 0.48(0.40,0.56) | 3 |
| | UFH | 0.61(0.44,0.83) | 4 |
| Category 0: might be among the least effective | AP | 0.76(0.47,1.2) | 5 |
| | VKA | 0.81(0.61,1.1) | 6 |
| | | | |
| **Major bleeding (odds ratio; 95% confidence interval)** | | | |
| **High certainty (moderate to high certainty evidence)** | | | |
| Category0：among the least harmful | AP | 2.1(0.98,4.9) | 1 |
| | LMWH | 1.3(0.93,1.8) | 5 |
| | VKA | 0.84(0.50,1.4) | 6 |
| **Low certainty (low to very low certainty evidence)** | | | |
| Category 1: might be inferior to the most harmful or superior to the least harmful | UFH | 2.0(1.2,3.3) | 2 |
| | NOACs | 1.8(1.2,2.6) | 3 |
| Category 0: might be among the least harmful | TI | 1.5(0.93,2.4) | 4 |
| | | | |
| **All-cause mortality (odds ratio; 95% confidence interval)** | | | |
| **High certainty (moderate to high certainty evidence)** | | | |
| Category2： among the most harmful | VKA | 8.9(2.6,31.0) | 1 |
| Category1：inferior to the most harmful or superior to the least harmful | LMWH | 5.4(2.2,13.0) | 3 |
| | NOACs | 3.0(1.3,7.1) | 4 |
| Category0：among the least harmful | UFH | 3.6(0.59,22.0) | 5 |

| | | | |
|---|---|---|---|
| | TI | 1.4(0.27,7.6) | 6 |
| **Low certainty (low to very low certainty evidence)** | | | |
| Category 1: might be inferior to the most harmful or superior to the least harmful | AP | 7.3(1.2,43.0) | 2 |
| | | | |
| **Clinical relevant non-major bleeding (odds ratio; 95% confidence interval)** | | | |
| **High certainty (moderate to high certainty evidence)** | | | |
| Category0：among the least harmful | AP | 1.40(0.52,3.9) | 1 |
| | TI | 1.40(0.69,2.5) | 2 |
| | NOACs | 1.40(0.88,2.2) | 3 |
| | LMWH | 1.1(0.69,2.5) | 4 |
| | | | |
| **VTE related death (odds ratio; 95% confidence interval)** | | | |
| **High certainty (moderate to high certainty evidence)** | | | |
| Category0：among the least harmful | VKA | 2.6(0.20,65.0) | 1 |
| | UFH | 1.5(0.10,43.0) | 2 |
| | TI | 1.1(0.14,9.2) | 3 |
| | AP | 0.95(0.08,11.0) | 4 |
| | NAOCs | 0.73(0.31,1.3) | 5 |
| | LMWH | 0.71(0.28,1.6) | 6 |
| | Placebo | Reference | - |

### 5.4 Network estimates (league tables)

**GRADE evidence levels represented by different colors**

High Moderate Low Very low

**Outcome：Recurrent VTE during treatment**

| | | | | | | |
|---|---|---|---|---|---|---|
| AP | 0.37 (0.24, 0.58) | 0.34 (0.23, 0.5) | 1.5 (1.04, 2.16) | 0.34 (0.19, 0.59) | 0.55 (0.33, 0.93) | 0.44 (0.29, 0.69) |
| 2.71 (1.72, 4.25) | LMWH | 0.92 (0.71, 1.17) | 4.06 (2.9, 5.66) | 0.91 (0.57, 1.44) | 1.48 (1.16, 1.92) | 1.21 (0.93, 1.57) |
| 2.96 (2, 4.4) | 1.09 (0.86, 1.41) | NOACs | 4.43 (3.42, 5.76) | 1 ( 0.64, 1.53) | 1.62 (1.15, 2.32) | 1.32 (1.07, 1.64) |
| 0.67 (0.46, 0.96) | 0.25 (0.18, 0.35) | 0.23 (0.17, 0.29) | Placebo | 0.22 (0.14, 0.35) | 0.37 (0.24, 0.56) | 0.3 (0.22, 0.41) |
| 2.96 (1.7, 5.26) | 1.09 (0.69, 1.76) | 1 (0.65, 1.56) | 4.44 (2.82, 7.13) | TI | 1.62 (0.97, 2.79) | 1.32 (0.9, 1.99) |
| 1.83 (1.08, 3.03) | 0.67 (0.52, 0.86) | 0.62 (0.43, 0.87) | 2.73 (1.8, 4.12) | 0.62 (0.36, 1.03) | UFH | 0.81 (0.57, 1.16) |
| 2.25 (1.45, 3.46) | 0.83 (0.64, 1.08) | 0.76 (0.61, 0.94) | 3.37 (2.47, 4.57) | 0.76 (0.5, 1.12) | 1.23 (0.87, 1.76) | VKA |

**Outcome：Major bleeding during treatment**

| | | | | | | |
|---|---|---|---|---|---|---|
| AP | 1.62 (0.66, 4.02) | 1.61 (0.69, 3.79) | 0.72 (0.3, 1.68) | 1.44 (0.53, 4.08) | 1.84 (0.71, 4.84) | 2.14 (0.89, 5.31) |
| 0.62 (0.25, 1.52) | LMWH | 0.99 (0.71, 1.4) | 0.45 (0.24, 0.8) | 0.89 (0.46, 1.75) | 1.14 (0.81, 1.58) | 1.33 (0.92, 1.94) |
| 0.62 (0.26, 1.44) | 1.01 (0.72, 1.41) | NOACs | 0.45 (0.26, 0.75) | 0.9 (0.49, 1.69) | 1.14 (0.71, 1.82) | 1.33 (1, 1.82) |
| 1.38 (0.59, 3.3) | 2.22 (1.25, 4.23) | 2.21 (1.33, 3.92) | Placebo | 1.98 (0.97, 4.5) | 2.53 (1.31, 5.18) | 2.95 (1.69, 5.6) |
| 0.69 (0.24, 1.89) | 1.12 (0.57, 2.15) | 1.12 (0.59, 2.04) | 0.5 (0.22, 1.03) | TI | 1.28 (0.6, 2.62) | 1.49 (0.85, 2.58) |
| 0.54 (0.21, 1.41) | 0.88 (0.63, 1.24) | 0.87 (0.55, 1.41) | 0.39 (0.19, 0.76) | 0.78 (0.38, 1.67) | UFH | 1.17 (0.72, 1.94) |
| 0.47 (0.19, 1.12) | 0.75 (0.51, 1.09) | 0.75 (0.55, 1) | 0.34 (0.18, 0.59) | 0.67 (0.39, 1.17) | 0.86 (0.52, 1.39) | VKA |

**Outcome：Clinical relevant non-major bleeding during treatment**

| | | | | | | |
|---|---|---|---|---|---|---|
| AP | 0.82 (0.43, 1.61) | 1.28 (0.74, 2.23) | 0.74 (0.4, 1.35) | 1.19 (0.59, 2.51) | 0.9 (0.28, 2.99) | 1.59 (0.87, 2.9) |
| 1.22 (0.62, 2.35) | LMWH | 1.56 (1.07, 2.25) | 0.9 (0.51, 1.51) | 1.44 (0.81, 2.65) | 1.1 (0.41, 2.97) | 1.93 (1.24, 2.97) |
| 0.78 (0.45, 1.35) | 0.64 (0.44, 0.93) | NOACs | 0.58 (0.38, 0.84) | 0.92 (0.59, 1.51) | 0.7 (0.25, 2.03) | 1.24 (0.98, 1.56) |
| 1.35 (0.74, 2.53) | 1.11 (0.66, 1.94) | 1.73 (1.19, 2.63) | Placebo | 1.6 (0.9, 3.12) | 1.22 (0.4, 3.82) | 2.14 (1.38, 3.47) |
| 0.84 (0.4, 1.71) | 0.69 (0.38, 1.24) | 1.08 (0.66, 1.68) | 0.63 (0.32, 1.11) | TI | 0.76 (0.24, 2.38) | 1.34 (0.87, 1.95) |
| 1.11 (0.33, 3.62) | 0.91 (0.34, 2.46) | 1.43 (0.49, 4.08) | 0.82 (0.26, 2.5) | 1.32 (0.42, 4.23) | UFH | 1.76 (0.6, 5.15) |
| 0.63 (0.35, 1.14) | 0.52 (0.34, 0.81) | 0.81 (0.64, 1.02) | 0.47 (0.29, 0.72) | 0.75 (0.51, 1.14) | 0.57 (0.19, 1.67) | VKA |

**Outcome：VTE related death during treatment**

| | | | | | | |
|---|---|---|---|---|---|---|
| AP | 0.95<br>(0.21, 5.01) | 0.82<br>(0.26, 2.65) | 1.28<br>(0.4, 4.07) | 0.26<br>(0.04, 1.66) | 1.23<br>(0.19, 9.06) | 0.63<br>(0.14, 2.76) |
| 1.05<br>(0.2, 4.68) | LMWH | 0.86<br>(0.27, 2.59) | 1.33<br>(0.34, 4.69) | 0.27<br>(0.04, 1.62) | 1.27<br>(0.43, 3.78) | 0.66<br>(0.13, 2.68) |
| 1.21<br>(0.38, 3.85) | 1.16<br>(0.39, 3.73) | NOACs | 1.56<br>(0.71, 3.2) | 0.31<br>(0.06, 1.36) | 1.49<br>(0.32, 7.64) | 0.77<br>(0.28, 2.01) |
| 0.78<br>(0.25, 2.49) | 0.75<br>(0.21, 2.96) | 0.64<br>(0.31, 1.41) | Placebo | 0.2<br>(0.04, 1.08) | 0.97<br>(0.18, 5.92) | 0.49<br>(0.15, 1.7) |
| 3.91<br>(0.6, 26.4) | 3.75<br>(0.62, 27.46) | 3.19<br>(0.74, 15.67) | 5<br>(0.93, 27.91) | TI | 4.74<br>(0.59, 46.95) | 2.44<br>(0.78, 8.39) |
| 0.81<br>(0.11, 5.26) | 0.79<br>(0.26, 2.32) | 0.67<br>(0.13, 3.13) | 1.03<br>(0.17, 5.59) | 0.21<br>(0.02, 1.7) | UFH | 0.52<br>(0.08, 3.06) |
| 1.59<br>(0.36, 7) | 1.52<br>(0.37, 7.47) | 1.3<br>(0.5, 3.58) | 2.03<br>(0.59, 6.82) | 0.41<br>(0.12, 1.28) | 1.94<br>(0.33, 13.22) | VKA |

**Outcome：Fatal bleeding during treatment**

| | | | | | | |
|---|---|---|---|---|---|---|
| AP | 1.15<br>(0.12, 8.55) | 0.75<br>(0.12, 3.91) | 0.31<br>(0.02, 3.88) | 1.21<br>(0.06, 14.24) | 1.84<br>(0.14, 27.8) | 2.51<br>(0.32, 15.56) |
| 0.87<br>(0.12, 8.04) | LMWH | 0.67<br>(0.21, 2.1) | 0.28<br>(0.03, 1.8) | 1.06<br>(0.1, 8.72) | 1.65<br>(0.34, 9.5) | 2.17<br>(0.65, 7.66) |
| 1.33<br>(0.26, 8.31) | 1.5<br>(0.48, 4.78) | NOACs | 0.43<br>(0.04, 2.96) | 1.59<br>(0.16, 11.41) | 2.52<br>(0.34, 20.78) | 3.29<br>(1.45, 8.19) |
| 3.2<br>(0.26, 52.39) | 3.51<br>(0.56, 34.57) | 2.31<br>(0.34, 23.49) | Placebo | 3.8<br>(0.2, 79.61) | 5.83<br>(0.5, 103.24) | 7.74<br>(0.99, 80.24) |
| 0.83<br>(0.07, 16.71) | 0.95<br>(0.11, 10.48) | 0.63<br>(0.09, 6.24) | 0.26<br>(0.01, 5) | TI | 1.56<br>(0.1, 31.41) | 2.02<br>(0.37, 18.01) |
| 0.54<br>(0.04, 7.4) | 0.6<br>(0.11, 2.92) | 0.4<br>(0.05, 2.9) | 0.17<br>(0.01, 1.98) | 0.64<br>(0.03, 9.58) | UFH | 1.34<br>(0.16, 10.03) |
| 0.4<br>(0.06, 3.11) | 0.46<br>(0.13, 1.53) | 0.3<br>(0.12, 0.69) | 0.13<br>(0.01, 1.01) | 0.5<br>(0.06, 2.7) | 0.75<br>(0.1, 6.34) | VKA |

**Outcome： Adverse events during treatment**

| | | | | |
|---|---|---|---|---|
| LMWH | 0.93<br>(0.55, 1.59) | 0.13<br>(0, 1.09) | 0.95<br>(0.47, 2) | 1.07<br>(0.56, 2.11) |
| 1.08<br>(0.63, 1.82) | NOACs | 0.14<br>(0.01, 1.25) | 1.03<br>(0.63, 1.71) | 1.15<br>(0.77, 1.74) |
| 7.62<br>(0.92, 210.47) | 7.06<br>(0.8, 193.7) | Placebo | 7.31<br>(0.78, 206.01) | 8.18<br>(0.9, 228.73) |
| 1.05<br>(0.5, 2.14) | 0.97<br>(0.59, 1.59) | 0.14<br>(0, 1.28) | TI | 1.12<br>(0.84, 1.49) |
| 0.94<br>(0.47, 1.79) | 0.87<br>(0.57, 1.3) | 0.12<br>(0, 1.12) | 0.89<br>(0.67, 1.19) | VKA |

**Outcome: All-cause mortality during treatment**

| | | | | | | |
|---|---|---|---|---|---|---|
| AP | 0.62<br>(0.33, 1.17) | 0.58<br>(0.32, 1.06) | 1.2<br>(0.67, 2.13) | 0.59<br>(0.26, 1.35) | 0.76<br>(0.38, 1.51) | 0.61<br>(0.31, 1.19) |
| 1.61<br>(0.85, 3.04) | LMWH | 0.93<br>(0.75, 1.15) | 1.92<br>(1.3, 2.84) | 0.95<br>(0.54, 1.68) | 1.22<br>(0.94, 1.59) | 0.98<br>(0.74, 1.31) |
| 1.73<br>(0.94, 3.17) | 1.07<br>(0.87, 1.34) | NOACs | 2.07<br>(1.47, 2.89) | 1.02<br>(0.58, 1.81) | 1.31<br>(0.94, 1.86) | 1.05<br>(0.8, 1.42) |
| 0.83<br>(0.47, 1.5) | 0.52<br>(0.35, 0.77) | 0.48<br>(0.35, 0.68) | Placebo | 0.49<br>(0.26, 0.95) | 0.64<br>(0.4, 1.02) | 0.51<br>(0.33, 0.79) |
| 1.69<br>(0.74, 3.87) | 1.05<br>(0.59, 1.86) | 0.98<br>(0.55, 1.73) | 2.03<br>(1.05, 3.84) | TI | 1.29<br>(0.69, 2.41) | 1.04<br>(0.63, 1.7) |
| 1.31<br>(0.66, 2.6) | 0.82<br>(0.63, 1.06) | 0.76<br>(0.54, 1.07) | 1.57<br>(0.98, 2.5) | 0.78<br>(0.41, 1.45) | UFH | 0.8<br>(0.55, 1.18) |
| 1.64<br>(0.84, 3.18) | 1.02<br>(0.76, 1.35) | 0.95<br>(0.71, 1.25) | 1.96<br>(1.26, 2.99) | 0.97<br>(0.59, 1.58) | 1.24<br>(0.85, 1.82) | VKA |

**Outcome: VTE during prophylaxis**

| | | | | | | |
|---|---|---|---|---|---|---|
| AP | 0.62<br>(0.38, 1.01) | 0.41<br>(0.25, 0.68) | 1.31<br>(0.8, 2.14) | 0.6<br>(0.35, 1.02) | 0.79<br>(0.45, 1.37) | 1.05<br>(0.62, 1.77) |
| 1.6<br>(0.99, 2.63) | LMWH | 0.67<br>(0.59, 0.75) | 2.1<br>(1.78, 2.48) | 0.96<br>(0.77, 1.2) | 1.27<br>(0.97, 1.66) | 1.68<br>(1.34, 2.11) |
| 2.41<br>(1.48, 3.97) | 1.5<br>(1.33, 1.7) | NOACs | 3.15<br>(2.61, 3.82) | 1.45<br>(1.13, 1.85) | 1.9<br>(1.43, 2.55) | 2.53<br>(1.99, 3.22) |
| 0.77<br>(0.47, 1.25) | 0.48<br>(0.4, 0.56) | 0.32<br>(0.26, 0.38) | Placebo | 0.46<br>(0.35, 0.6) | 0.6<br>(0.44, 0.82) | 0.8<br>(0.61, 1.05) |
| 1.66<br>(0.98, 2.86) | 1.04<br>(0.83, 1.3) | 0.69<br>(0.54, 0.89) | 2.17<br>(1.66, 2.88) | TI | 1.32<br>(0.93, 1.87) | 1.75<br>(1.32, 2.32) |
| 1.27<br>(0.73, 2.21) | 0.79<br>(0.6, 1.03) | 0.53<br>(0.39, 0.7) | 1.65<br>(1.21, 2.26) | 0.76<br>(0.54, 1.07) | UFH | 1.33<br>(0.94, 1.88) |
| 0.95<br>(0.56, 1.62) | 0.59<br>(0.47, 0.75) | 0.4<br>(0.31, 0.5) | 1.24<br>(0.95, 1.64) | 0.57<br>(0.43, 0.76) | 0.75<br>(0.53, 1.07) | VKA |

**Outcome: Major bleeding during prophylaxis**

| | | | | | | |
|---|---|---|---|---|---|---|
| AP | 0.61 (0.26, 1.36) | 0.84 (0.35, 1.88) | 0.47 (0.2, 1.02) | 0.69 (0.27, 1.66) | 0.92 (0.37, 2.26) | 0.39 (0.15, 0.94) |
| 1.64 (0.73, 3.87) | LMWH | 1.38 (1.07, 1.76) | 0.77 (0.55, 1.08) | 1.13 (0.8, 1.61) | 1.51 (1.04, 2.25) | 0.64 (0.43, 0.94) |
| 1.19 (0.53, 2.82) | 0.73 (0.57, 0.93) | NOACs | 0.56 (0.39, 0.8) | 0.82 (0.54, 1.26) | 1.1 (0.71, 1.74) | 0.47 (0.29, 0.72) |
| 2.13 (0.98, 4.91) | 1.3 (0.93, 1.83) | 1.79 (1.24, 2.6) | Placebo | 1.48 (0.9, 2.4) | 1.97 (1.19, 3.31) | 0.84 (0.49, 1.38) |
| 1.44 (0.6, 3.69) | 0.88 (0.62, 1.26) | 1.21 (0.79, 1.86) | 0.68 (0.42, 1.11) | TI | 1.33 (0.8, 2.28) | 0.57 (0.35, 0.9) |
| 1.08 (0.44, 2.74) | 0.66 (0.44, 0.96) | 0.91 (0.58, 1.42) | 0.51 (0.3, 0.84) | 0.75 (0.44, 1.25) | UFH | 0.43 (0.24, 0.73) |
| 2.55 (1.06, 6.63) | 1.56 (1.06, 2.35) | 2.14 (1.38, 3.41) | 1.2 (0.72, 2.02) | 1.76 (1.11, 2.87) | 2.35 (1.38, 4.23) | VKA |

**Outcome: All-cause mortality during prophylaxis**

| | | | | | | |
|---|---|---|---|---|---|---|
| AP | 0.75<br>(0.14, 4.04) | 0.41<br>(0.07, 2.4) | 0.14<br>(0.02, 0.85) | 0.2<br>(0.02, 1.73) | 0.49<br>(0.05, 4.91) | 1.23<br>(0.2, 7.51) |
| 1.34<br>(0.25, 7.35) | LMWH | 0.55<br>(0.31, 1.01) | 0.18<br>(0.07, 0.45) | 0.26<br>(0.06, 1.12) | 0.66<br>(0.14, 3.13) | 1.63<br>(0.61, 4.45) |
| 2.42<br>(0.42, 13.81) | 1.81<br>(0.99, 3.24) | NOACs | 0.33<br>(0.14, 0.78) | 0.47<br>(0.1, 2.13) | 1.2<br>(0.23, 6.21) | 2.96<br>(1.09, 8.03) |
| 7.29<br>(1.18, 43.47) | 5.44<br>(2.21, 13.34) | 3.01<br>(1.29, 7.1) | Placebo | 1.42<br>(0.27, 7.63) | 3.61<br>(0.59, 21.64) | 8.94<br>(2.56, 31.24) |
| 5.11<br>(0.58, 45.09) | 3.82<br>(0.89, 16.31) | 2.11<br>(0.47, 9.75) | 0.7<br>(0.13, 3.76) | TI | 2.53<br>(0.3, 21.03) | 6.28<br>(1.46, 26.81) |
| 2.02<br>(0.2, 20.1) | 1.51<br>(0.32, 7.13) | 0.83<br>(0.16, 4.41) | 0.28<br>(0.05, 1.69) | 0.4<br>(0.05, 3.33) | UFH | 2.48<br>(0.4, 15.49) |
| 0.81<br>(0.13, 5.1) | 0.61<br>(0.22, 1.63) | 0.34<br>(0.12, 0.92) | 0.11<br>(0.03, 0.39) | 0.16<br>(0.04, 0.68) | 0.4<br>(0.06, 2.51) | VKA |

**Outcome: Clinical relevant non-major bleeding during prophylaxis**

| | | | | |
|---|---|---|---|---|
| AP | 0.84 (0.34, 1.92) | 1.02 (0.42, 2.31) | 0.76 (0.34, 1.64) | 1.01 (0.39, 2.45) |
| 1.19 (0.52, 2.92) | LMWH | 1.21 (1.06, 1.41) | 0.91 (0.64, 1.35) | 1.2 (0.88, 1.63) |
| 0.98 (0.43, 2.35) | 0.83 (0.71, 0.94) | NOACs | 0.75 (0.54, 1.08) | 0.99 (0.69, 1.38) |
| 1.31 (0.61, 2.95) | 1.1 (0.74, 1.56) | 1.34 (0.93, 1.86) | Placebo | 1.33 (0.79, 2.1) |
| 0.99 (0.41, 2.57) | 0.83 (0.61, 1.13) | 1.01 (0.73, 1.44) | 0.75 (0.48, 1.27) | TI |

**Outcome：VTE related death during prophylaxis**

| | | | | | | |
|---|---|---|---|---|---|---|
| AP | 0.73 (0.06, 9.82) | 0.75 (0.06, 9.41) | 1.06 (0.1, 12.55) | 1.17 (0.05, 28.89) | 1.73 (0.05, 85.69) | 2.74 (0.08, 138.43) |
| 1.38 (0.1, 18.14) | LMWH | 1.03 (0.45, 2.27) | 1.41 (0.63, 3.59) | 1.59 (0.21, 12.94) | 2.19 (0.17, 57.74) | 3.68 (0.34, 85.17) |
| 1.33 (0.11, 17.26) | 0.97 (0.44, 2.21) | NOACs | 1.37 (0.78, 3.19) | 1.55 (0.22, 12.63) | 2.22 (0.15, 62.84) | 3.62 (0.3, 91.73) |
| 0.95 (0.08, 10.51) | 0.71 (0.28, 1.58) | 0.73 (0.31, 1.29) | Placebo | 1.11 (0.14, 9.24) | 1.55 (0.1, 43.31) | 2.56 (0.2, 64.61) |
| 0.85 (0.03, 18.97) | 0.63 (0.08, 4.73) | 0.64 (0.08, 4.61) | 0.9 (0.11, 7.35) | TI | 1.41 (0.05, 60.7) | 2.34 (0.09, 92) |
| 0.58 (0.01, 20.67) | 0.46 (0.02, 5.79) | 0.45 (0.02, 6.69) | 0.65 (0.02, 9.72) | 0.71 (0.02, 19.95) | UFH | 1.76 (0.03, 97.68) |
| 0.36 (0.01, 12.3) | 0.27 (0.01, 2.97) | 0.28 (0.01, 3.32) | 0.39 (0.02, 4.93) | 0.43 (0.01, 11.4) | 0.57 (0.01, 35.65) | VKA |

## 5.5 Forest plots

**Footnotes: AP = Antiplatelet Drugs; LMWH= Low-molecular-weight heparin; UFH= Heparin ; NOACs=Novel oral anticoagulants; VKA= Vitamin K Inhibitors.**

### Outcome： Recurrent VTE during treatment

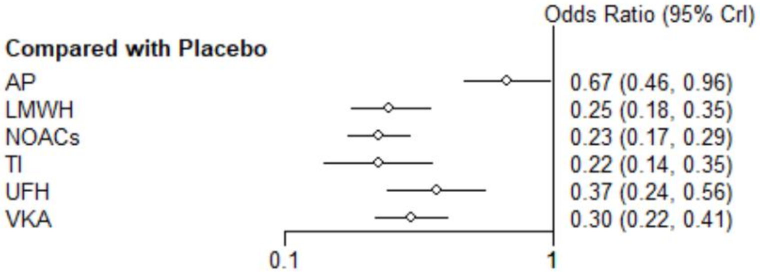


### Outcome： Major bleeding during treatment

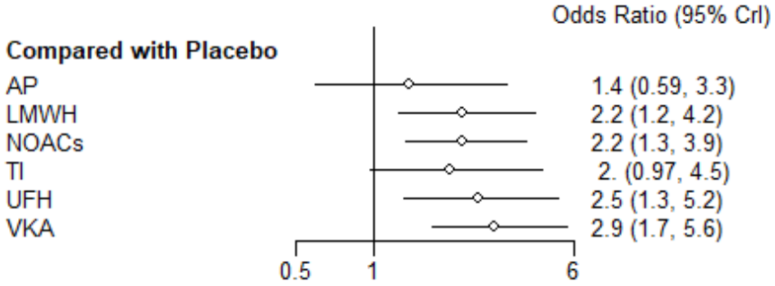


### Outcome： Clinical relevant non-major bleeding during treatment

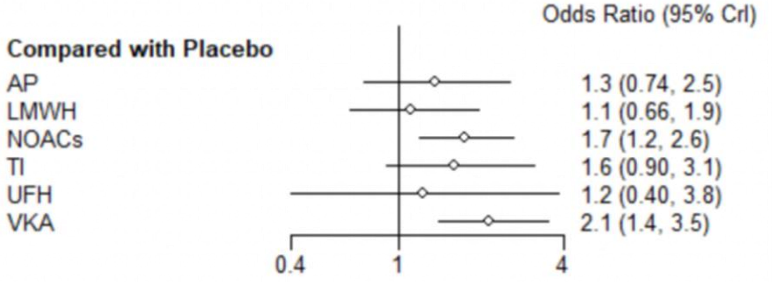

**Outcome：VTE related death during treatment**

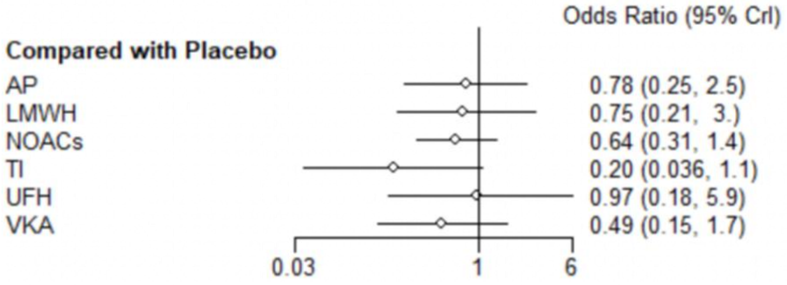


**Outcome：Fatal bleeding during treatment**

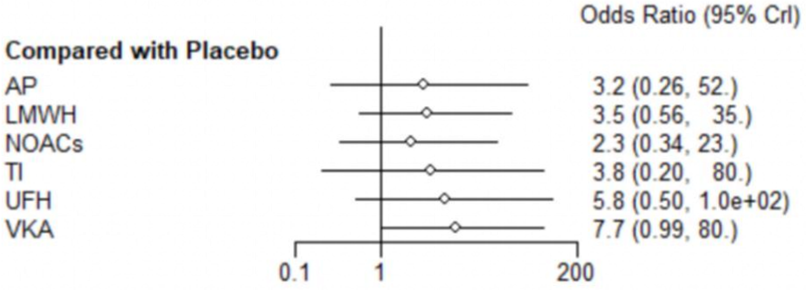


**Outcome：Adverse events during treatment**

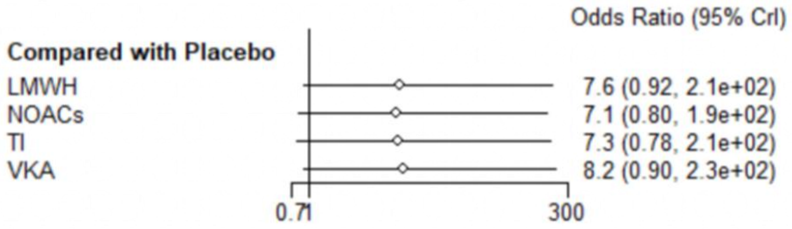

**Outcome: All-cause mortality during treatment**

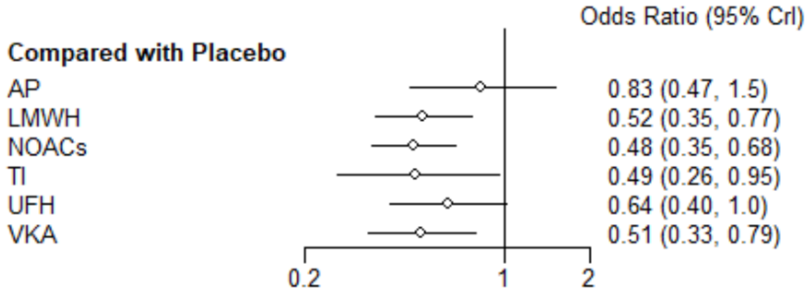


**Outcome: VTE during prophylaxis**

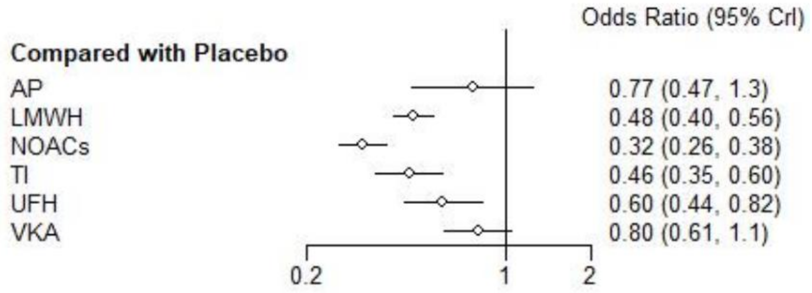


**Outcome: Major bleeding during prophylaxis**

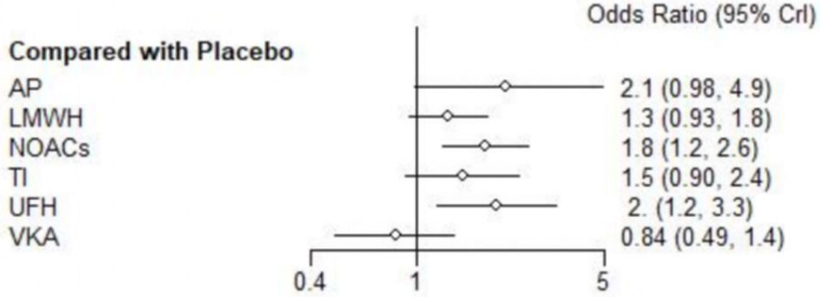

**Outcome: All-cause mortality during prophylaxis**

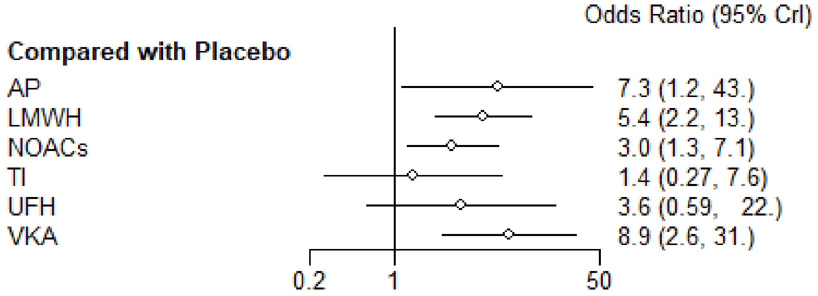


**Outcome: Clinical relevant non-major bleeding during prophylaxis**

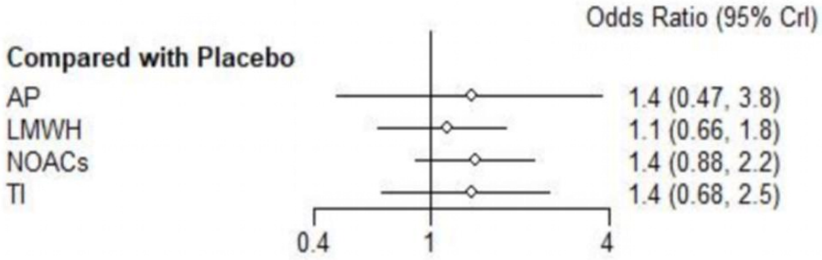


**Outcome: VTE related death during prophylaxis**

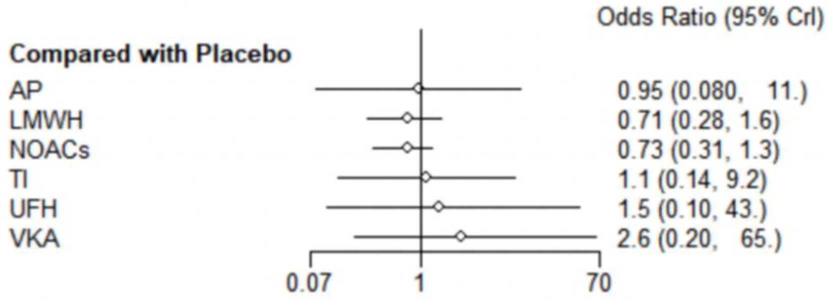

### 5.6 Heterogeneity
**Outcome: Recurrent VTE during treatment**

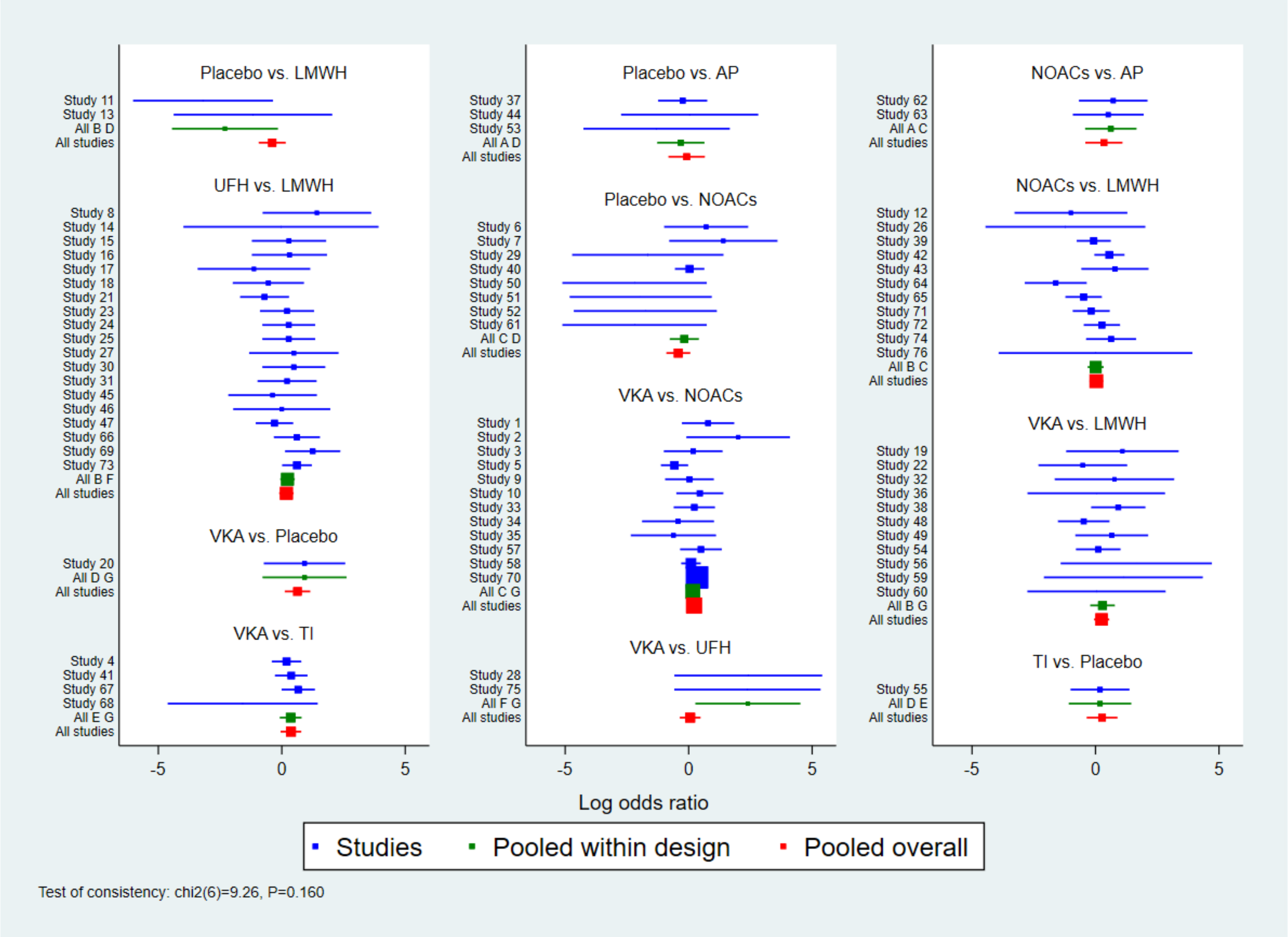

**Outcome: Major bleeding during treatment**

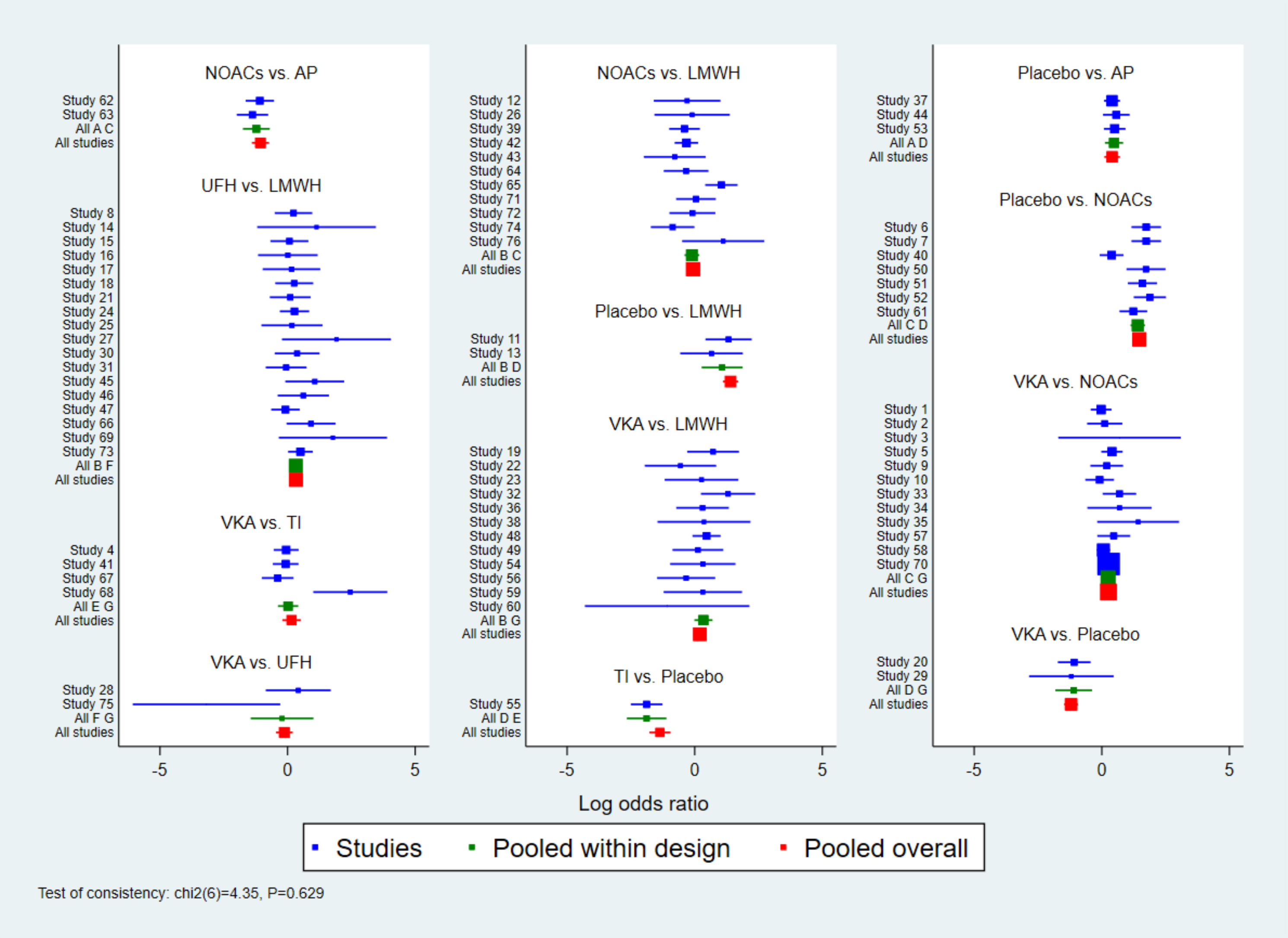

**Outcome: Clinical relevant non-major bleeding during treatment**

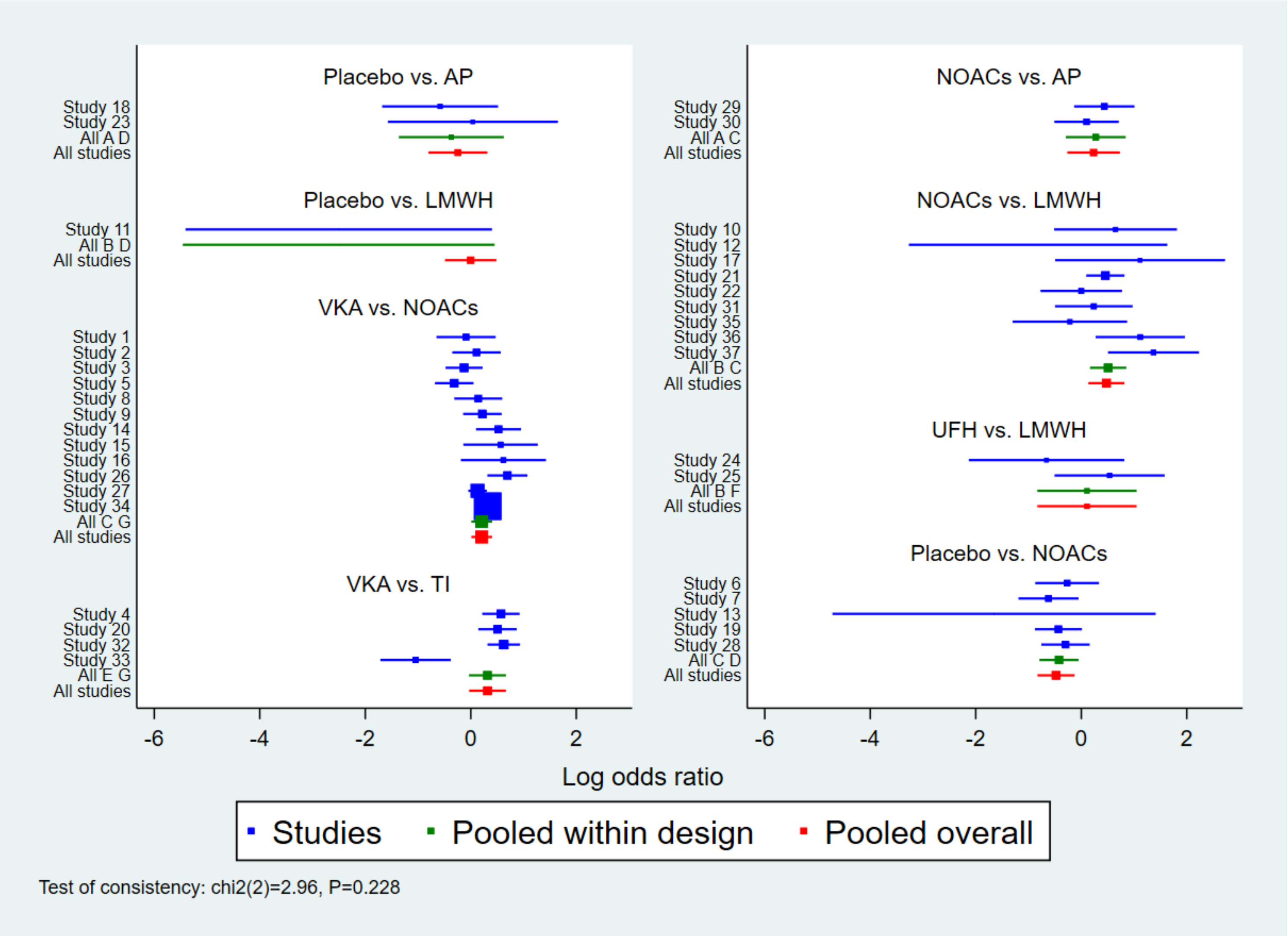

**Outcome：VTE related death during treatment**

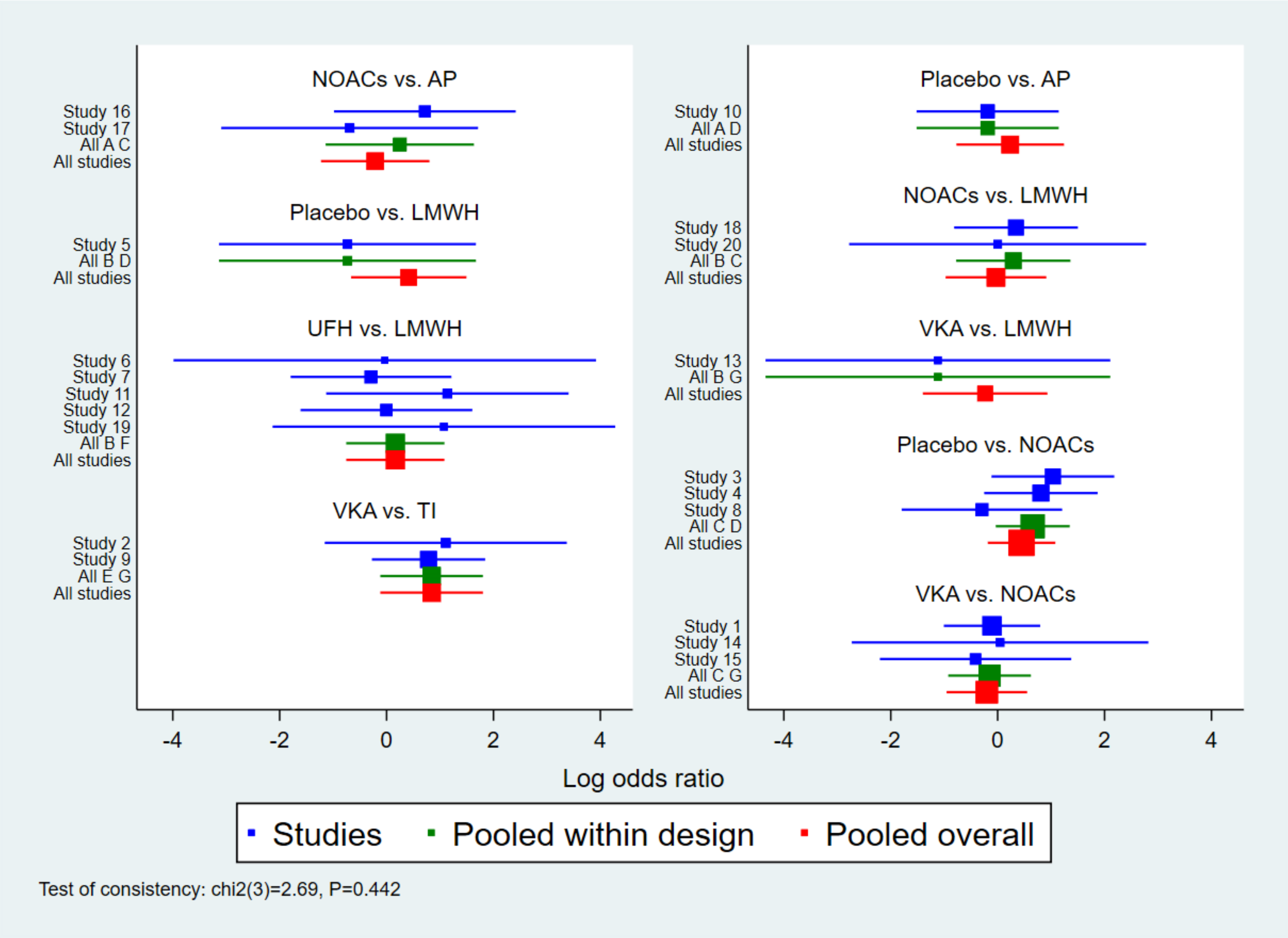

**Outcome: Fatal bleeding during treatment**

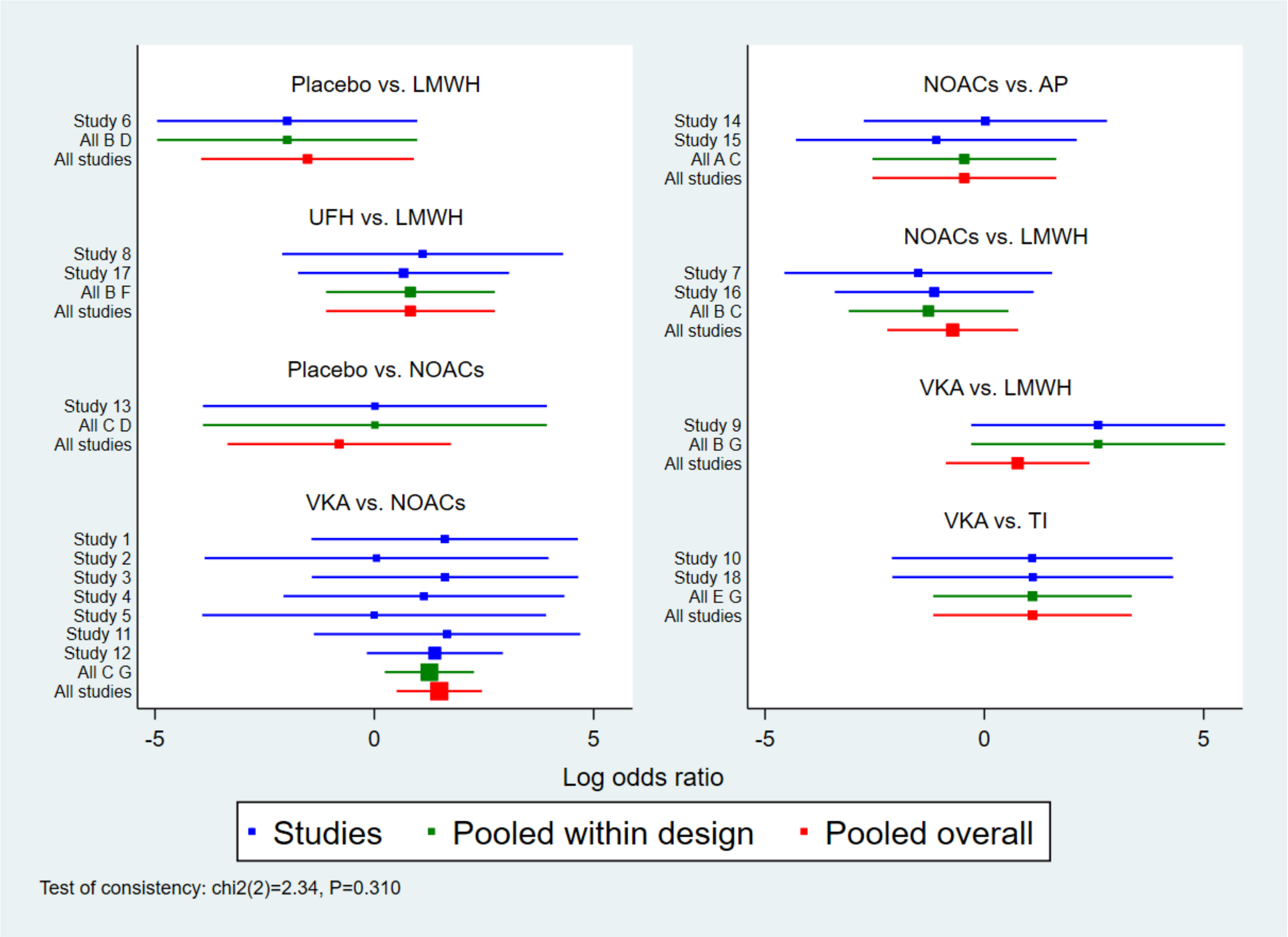

**Outcome：Adverse events during treatment**

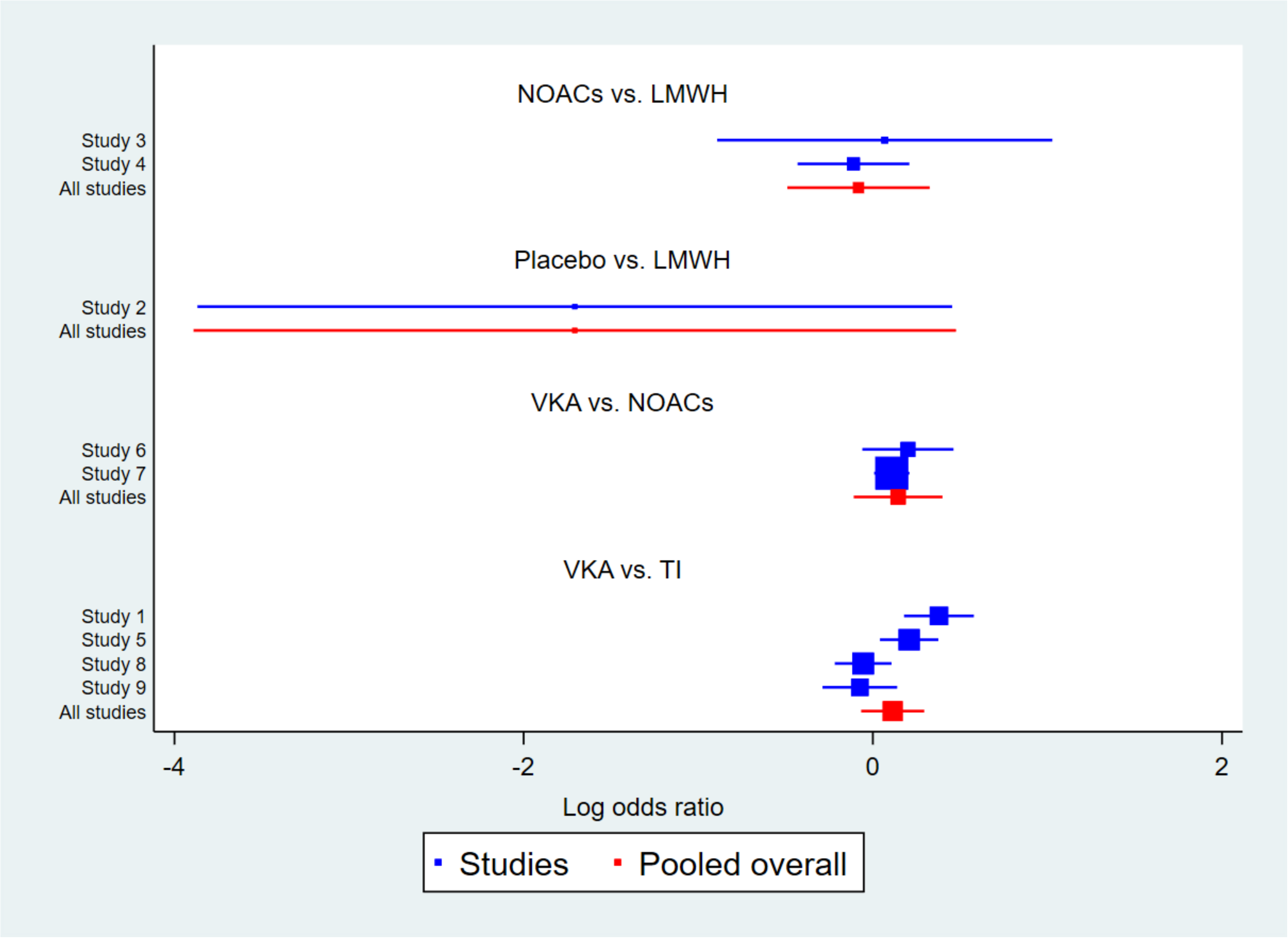

**Outcome: All-cause mortality during treatment**

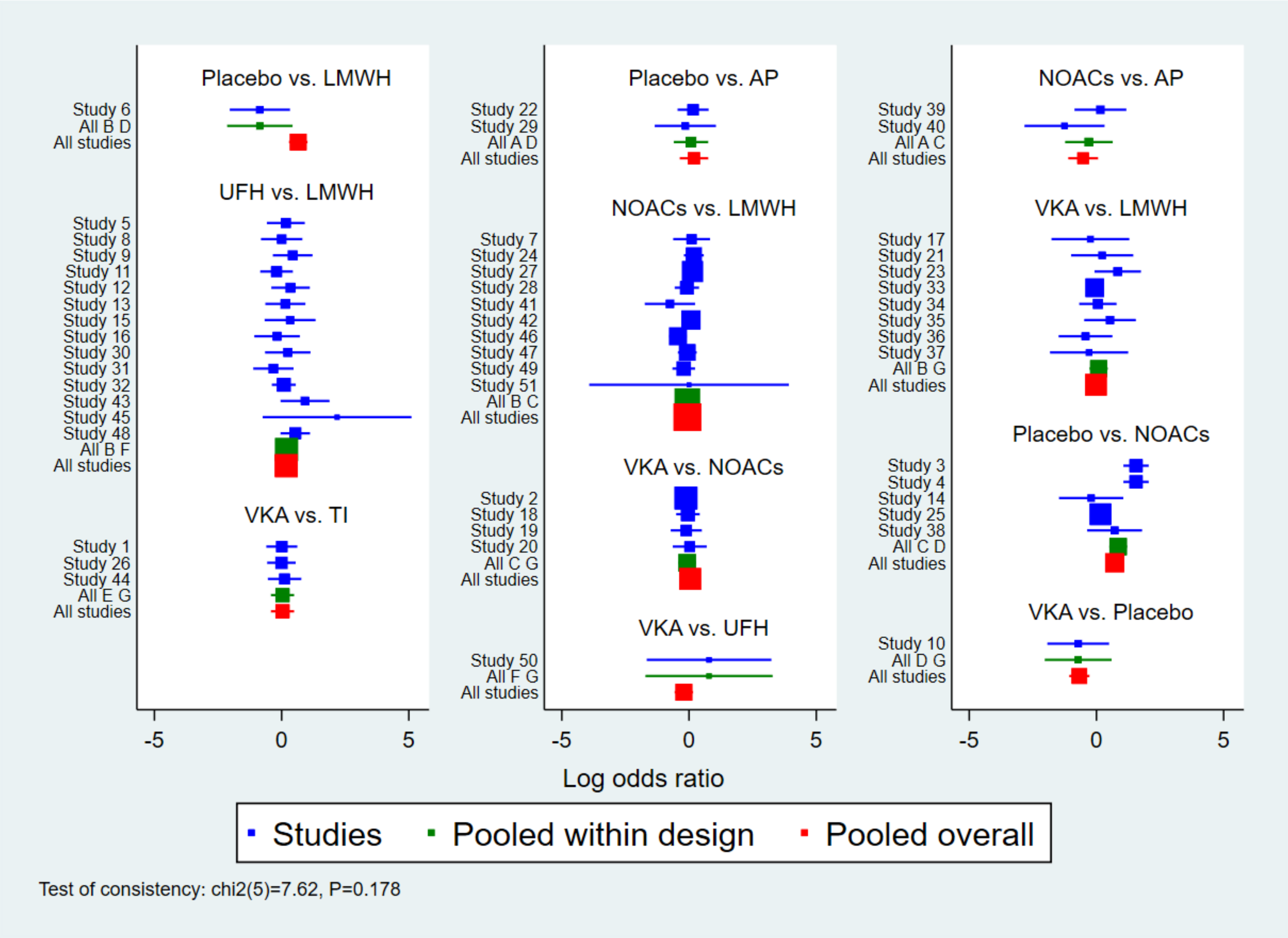

**Outcome: VTE during prophylaxis**

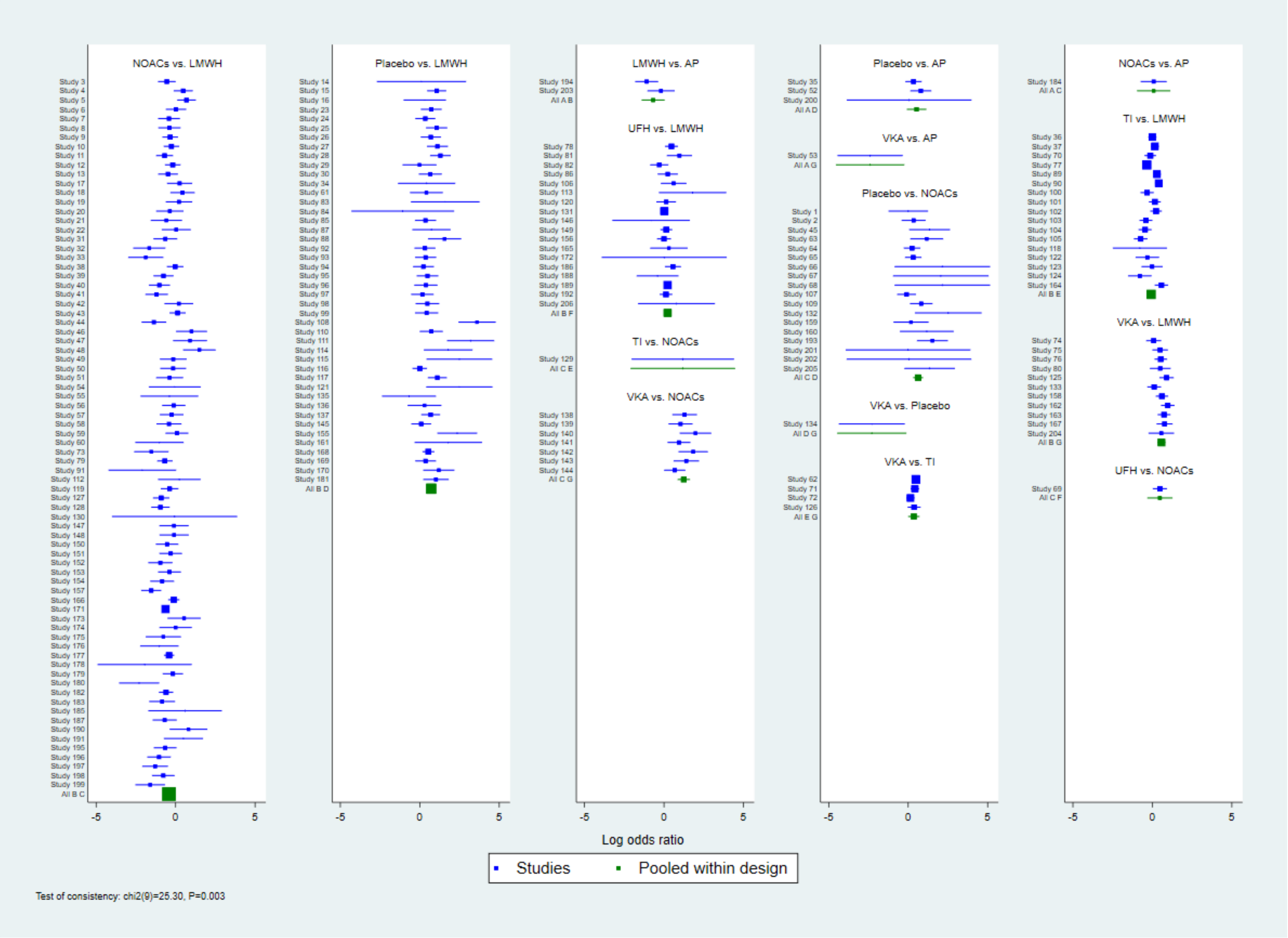

**Outcome: Major bleeding during prophylaxis**

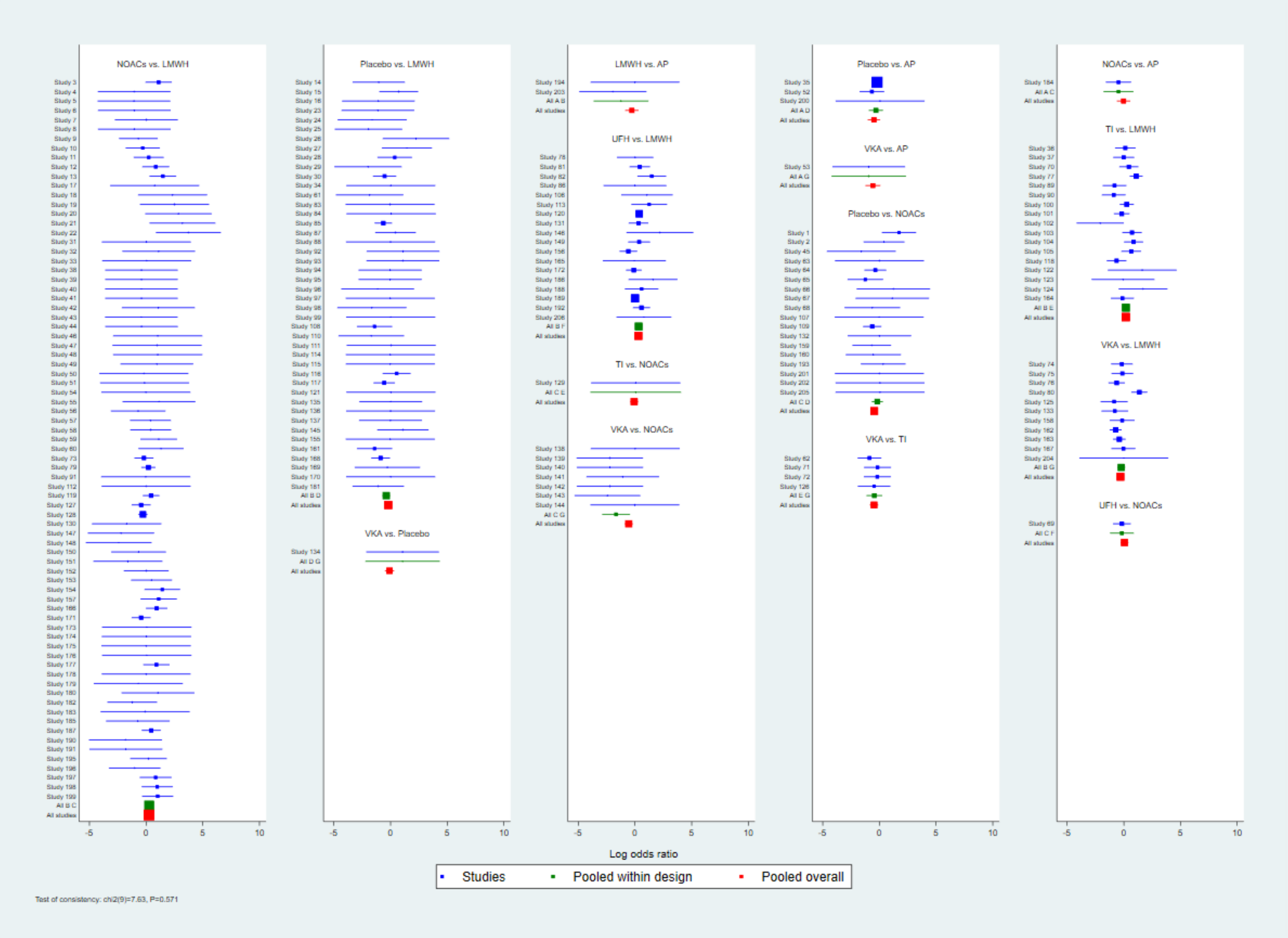

**Outcome: All-cause mortality during prophylaxis**

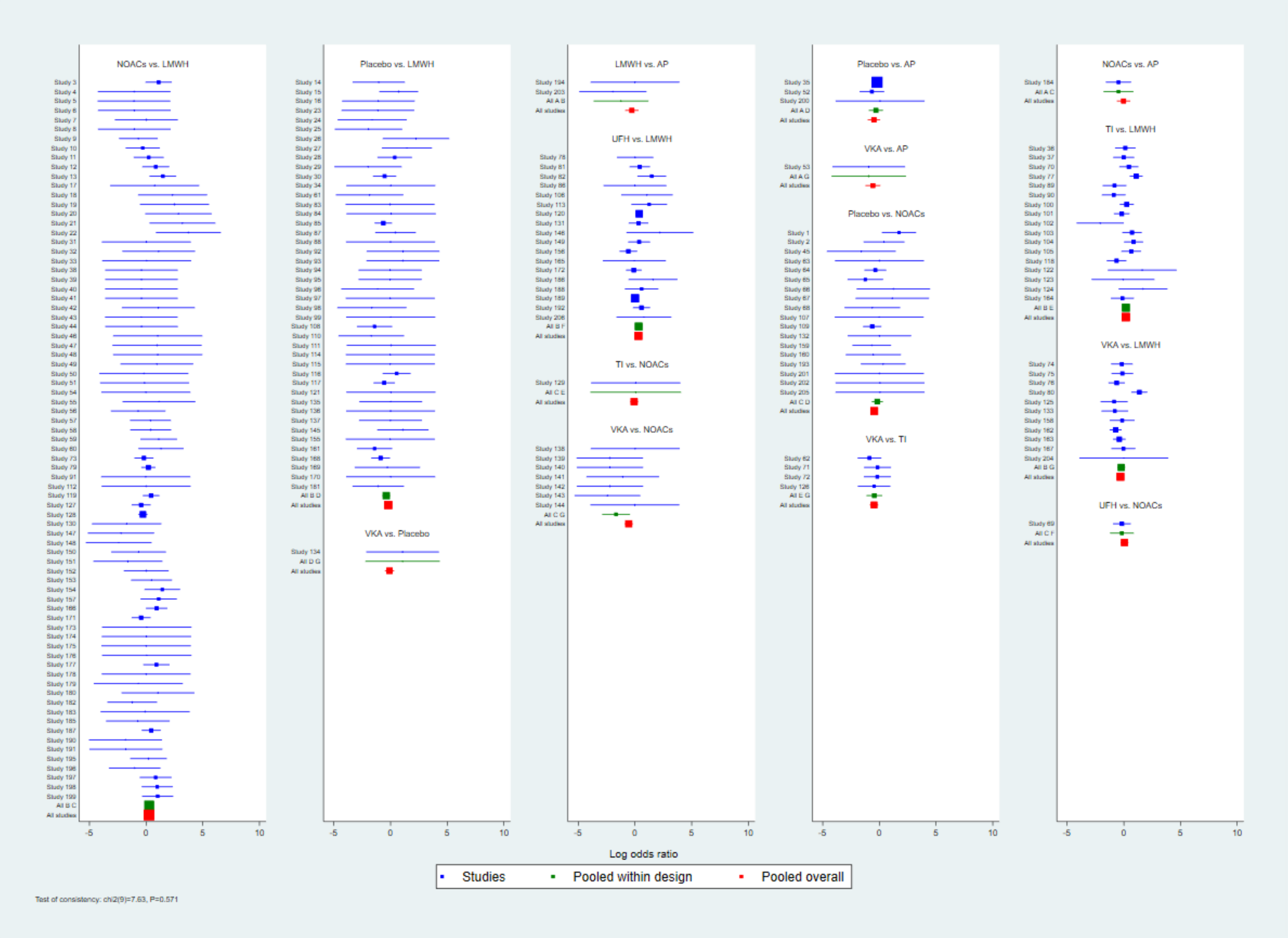

**Outcome: Clinical relevant non-major bleeding during prophylaxis**

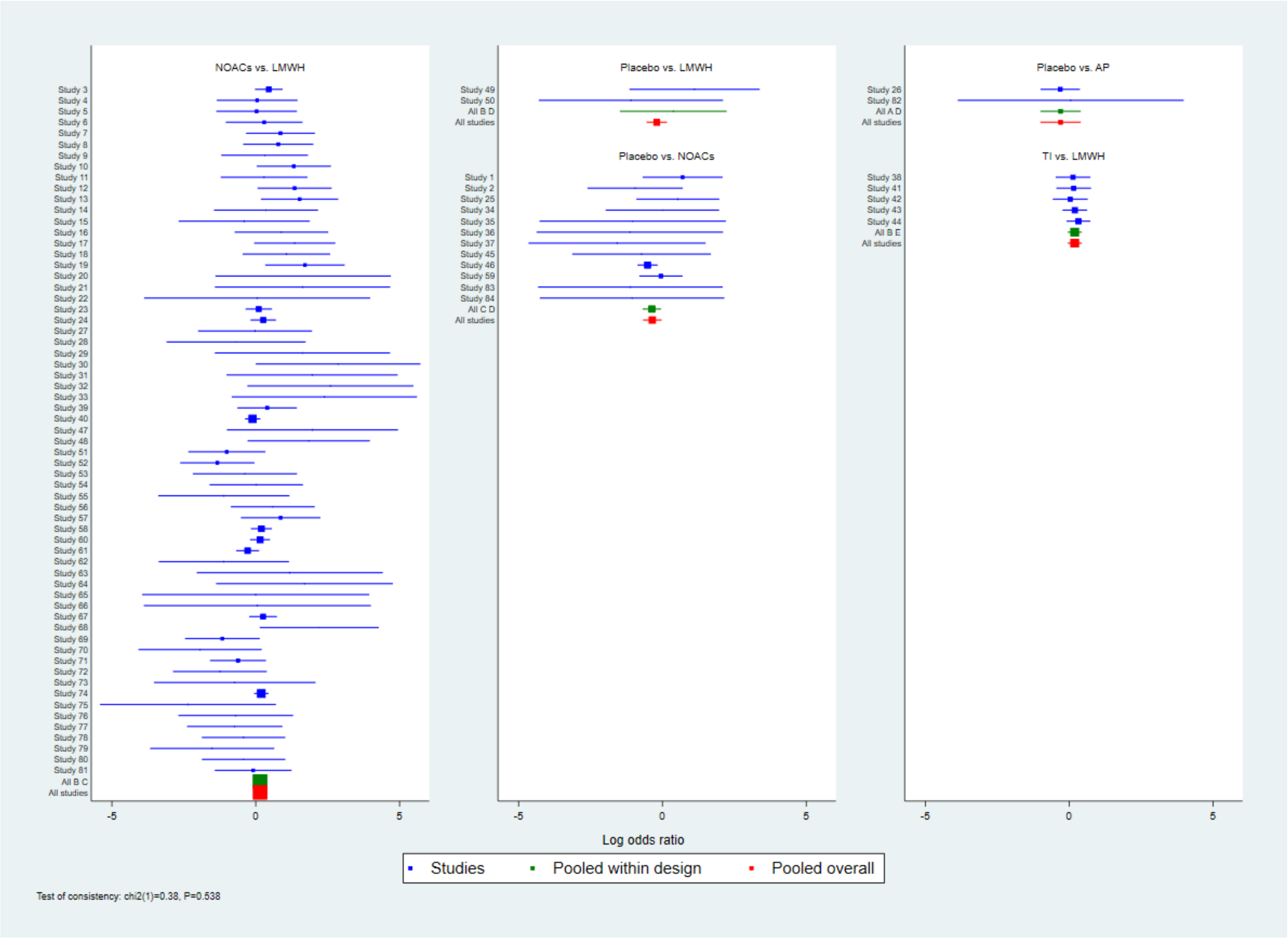

**Outcome: VTE related death during prophylaxis**

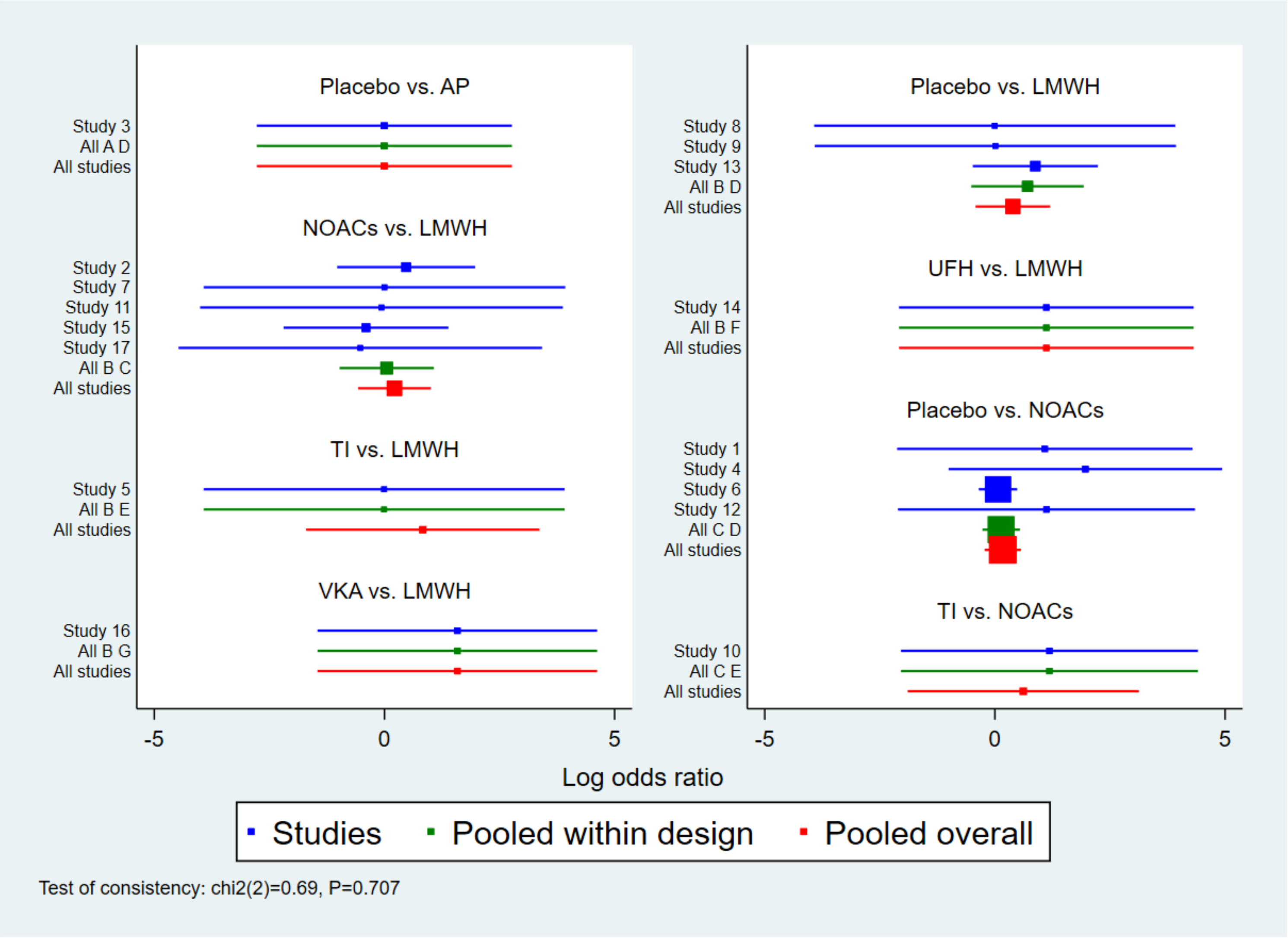

**Inconsistency (incoherence) assessments**
**Treatment**

| Comparison | Direct estimate Certainty | Indirect estimate Certainty | Network estimate Certainty | P Value |
|---|---|---|---|---|
| **Recurrent VTE during treatment** | | | | |
| NOACs vs AP | 1.9(0.61,6.5) | 1.3(0.39,4.6) | 1.6(0.69,3.8) | 0.67 |
| Placebo vs AP | 0.62 (0.20, 1.8) | 0.90 (0.24, 3.4) | 0.72(0.31,1.6) | 0.67 |
| NOACs vs LMWH | 0.95 (0.62,1.4) | 1.1 (0.62, 2.0) | 1.0 (0.72, 14) | 0.66 |
| Placebo vs LMWH | 8.9e-10 (1.3e-22, 0.016) | 0.63 (0.34,1.1) | 0.45 (0.24, 0.80) | 7.50E-04 |
| UFH vs LMWH | 1.3 (0.90, 1.8) | 1.7e-10 (5.9e-31, 0.050) | 1.1 (0.81, 1.6) | 0 |
| VKA vs LMWH | 1.4 (0.81, 2.6) | 1.3(0.78,2.1) | 1.3 (0.94, 2) | 0.77 |
| Placebo vs NOACs | 0.59 (0.30, 1.1) | 0 28 (0.12, 0.67) | 0.45 (0 25, 0.75) | 0.18 |
| VKAvs NOACs | 1.2 (0.87, 1.7) | 1.8 (0.98, 3.3) | 1.3 (1.0, 1.8) | 0.28 |
| TI vs Placebo | 1.2 (0.27, 5.6) | 2.4 (1.0, 6.3) | 2.(0.99, 4.5) | 0.44 |
| VKA vs Placebo | 2.7 (0.45, 25.0) | 3.0(1.7, 5.9) | 3.(1.7, 5.7) | 0.94 |
| VKA vs TI | 1.4 (0 73, 2.5) | 2.7 (0.52, 14) | 1.5 (0.85, 2.6) | 0.45 |
| VKA vs UFH | 3.7e+09 (27, 3.Oe+31) | 0.94 (0.58, 1.5) | 1.2 (0.73, 2) | 0 |
| **Major bleeding during treatment** | | | | |
| NOACs vs AP | 0.29 (0.15, 0.53) | 0.38 (0.22, 0.64) | 0.38 (0.22, 0.64) | 0.507525 |
| Placebo vs AP | 1.6 (1 0, 2 5) | 1.2 (0.62, 2.4) | 1.5 (1.0, 2.2) | 0.496775 |
| NOACs vs LMWH | 0.90 (0 65, 1.2) | 0.93 (0.62, 1.4) | 0.92 (0.71, 1.2) | 0.8792 |
| Placebo vs LMWH | 3.1 (1 3, 7.7) | 4.3(3.0,6.2) | 4.1(2.9,5.7) | 0.500475 |
| UFH vs LMWH | 1.4(1.1,1.9) | 3.0 (0.99, 9.8) | 1.5(1.2,1.9) | 0.2056 |
| VKA vs LMWH | 1.4 (0.96, 2.1) | 1.1 (0.74, 1.5) | 1. 2 (0 93, 1 6) | 0.265 |
| Placebo VS NOACs | 4.2(3.1,5.9) | 4.9 (3.1, 7.6) | 4.4(3.4, 5.8) | 0.612275 |
| VKA vs NOACs | 1.3 (1.0, 1.7) | 1.4 (0.91, 2.0) | 1.3(11,16) | 0.892925 |
| T1 vs Placebo | 0.15 (0.060, 0.35) | 0.26 (0.15 0.45) | 0.23(0.14, 0.35) | 0.26505 |
| VKA vs Placebo | 0.32(0.14, 0.72) | 0.29 (0.21, 0.41) | 0.30 (0.22, 0.41) | 0.818125 |
| VKA vs TI | 0.2 (0.78, 1.9) | 2.1 (0.85, 5.5) | 1.3(0.90,2.0) | 0.257225 |
| VKA vs UFH | 0.42 (0.13, 1.2) | 0.87 (0.60, 1.3) | 0.81 (0.57, 1.2) | 0.214475 |
| **Clinical relevant non-major bleeding during treatment** | | | | |

| | | | | |
|---|---|---|---|---|
| NOACs vs AP | 1.3(0.69,2.6) | 1.2 (0.36, 3.6) | 1.3(0.74,2.2) | 0.834625 |
| Placebo VS AP | 0.68 (0.23, 1 9) | 0.77 (0.34, 1.7) | 0.74 (0.40, 1.3) | 0.85185 |
| NOACs vs LMWH | 1 7 (1.2, 2.4) | 3.2e-08 (6.5e-17, 0.091) | 1.6 (1.1, 2.3) | 3.00E-04 |
| Placebo vs LMWH | 1.7e-11 (28e-51, 0.053) | 1.1(062,1 8) | 0.91 (0.52, 1.5) | 5.00E-05 |
| Placebo vs NOACs | 0.64 (0.42, 0.98) | 0.28 (0.090, 0.82) | 0.58 (0.38, 0.84) | 0.15712 |
| **VTE related death during treatment** | | | | |
| NOACs vs AP | 1.3 (0.27, 5.7) | 0.46 (0.077, 3.0) | 0.83 (0.24, 2.7) | 0.39695 |
| Placebo vs AP | 0.78 (0.14, 4.2) | 2.0(0.34, 12) | 1.3(0.39,4.1) | 0.408825 |
| NOACs vs LMWH | 1.3 (0.36, 5.2) | 0.18 (0.0058, 2.1) | 0.86 (0.28, 2.5) | 0.15555 |
| Placebo vs LMWH | 0.43 (0.013, 5.2) | 1.9(0.41,7.9) | 1.3 (0.36, 4.5) | 0.3293 |
| VKAvs LMWH | 3.9e-05 (4.9e-16, 0.66) | 0.80 (0.17, 3.8) | 0.66(0.15, 2.6) | 0.04845 |
| Placebo VS NOACs | 1.9 (0.84, 4.6) | 0.51 (0.085, 2.9) | 1.5 (0.70, 3.2) | O. 165 |
| VKA vs NOACs | 0.84 (0.30, 2.3) | 1.1e-05 (7.9e-15, 1.6) | 0.76 (0.29, 1.9) | 0.079475 |
| **Fatal bleeding during treatment** | | | | |
| NOACs vs LMWH | 0.39 (0.059, 1.8) | 1.7(0.25, 2.0) | 0.69 (0.20, 2.2) | 0.23835 |
| Placebo vs LMWH | 0.18 (0.0045, 1.6) | 0.84 (0.015, 5.5) | 0.31 (0.032, 1.8) | 0.479675 |
| VKAvs LMWH | 9.9 (1.3，2.9e+02) | 0.96 (0.15, 4.7) | 2.2 (0.64, 8.5) | 0.084775 |
| Placebo vs NOACs | 1.1 (0.023, 45) | 0.23 (0.0062, 3.3) | 0.45 (0.042，3.3) | 0.474 |
| VKA vs NOACs | 2.5 (1.1，6.4) | 28. (2.1, 1.2e+03) | 3.2 (1.4，8.3) | 0.083125 |
| **All-cause mortality during treatment** | | | | |
| NOACs vs AP | 0.68 (0.26, 1.7) | 0.51 (0.23, 1.1) | 0.58 (0.32, 1.1) | 0.637825 |
| Placebo VS AP | 1.1 (0.52, 2.2) | 1.5 (0.53, 3.9) | 1.2 (0.67, 2.1) | 0.625575 |
| NOACs vs LMWH | 0.93 (0.72, 1.2) | 0.94 (0.57, 1.5) | 0.93 (0.75, 1.2) | 0.9817 |
| Placebo vs LMWH | 0.40(0.097,1.5) | 2.2 (1.5, 3.3) | 1.9 (1.3, 2.8) | 0.0143 |
| UFH vs LMWH | 1.2 (0.96, 1.6) | 0.36 (0.0090, 5.3) | 1.2 (0.95, 1.6) | 0.36515 |
| VKA vs LMWH | 1.1 (0.76, 1.6) | 0.85 (0.55, 1 3) | 0.98(0.74,1.3) | 0.37475 |
| Placebo vs NOACs | 2.4 (1.7, 3.5) | 1.1 (0.55, 2.4) | 2.1 (1.5, 2.9) | 0.072075 |
| VKA vs NOACs | 0.93 (0.63, 1.4) | 1.2 (0.83, 1.9) | 1.1 (0.80, 1.4) | 0.29957 |
| VKA vs Placebo | 0.47(0.11,1.8) | 0.51 (0.3, 0.82) | 0.51 (0.34, 0.79) | 0.906825 |
| VKA vs UFH | 2.7(0.20, 9.3) | 0.78 (0.53, 1.2) | 0.80 (0.55, 1.2) | 0.3583 |

**Prevention**

| **Comparison** | **Direct estimate Certainty** | **Indirect estimate Certainty** | **Network estimate Certainty** | **P Value** |
|---|---|---|---|---|
| **VTE during prophylaxis** | | | | |
| LMWH vs AP | 0.51 (0.24, 1.1) | 0.76 (0.44, 1.3) | 0.67 (0.43, 1.0) | 0.523725 |
| NOACs vs AP | 1.1 (0.36, 3.4) | 0.41 (0.25, 0.67) | 0.48 (0.31, 0.75) | 0.088975 |
| Placebo VS AP | 1.7(0.88, 3.3) | 1.1(0.53, 1.7) | 1.2(0.80,1.9) | 0.3204 |
| VKA vs AP | 0.064 (0.0023, 0.50) | 1.4 (0.78, 2.4) | 1.1 (0.62, 1.8) | 0.0028 |
| NOACs vs LMWH | 0.64 (0.56, 0.73) | 0.79 (0.59, 1.1) | 0.67 (0.59, 0.75) | 0.212225 |
| Placebo VS LMWH | 2.2 (1.9, 2.7) | 1.7 (1.2, 2.4) | 2.1 (1.8, 2.5) | 0.138525 |
| Tl vs LMWH | 0.91 (0.71, 1.2) | 1.3 (0.76, 2.2) | 0.96 (0.77, 1.2) | 0.23545 |
| UFH vs LMWH | 1.3 (0.97, 1.7) | 1.1 (0.39, 2.9) | 1.3 (0.97, 1.7) | 0.72245 |
| VKA vs LMWH | 1.8 (1.3, 2.4) | 1.6 (1.1, 22) | 1.7 (1.3, 2.1) | 0.598825 |
| Placebo VS NOACs | 2.1(1.5,3.0) | 3.7(2.9,4.6) | 3.1 (2.6, 3.8) | 0.0099 |
| Tl vs NOACs | 1.2e+09 (1.8, 1.0e+25) | 1.4 (1.1, 1.8) | 1.4 (1.1, 1.9) | 0.04365 |
| UFH vs NOACs | 1.6 (0.59, 4.4) | 1.9 (1.4, 2.6) | 1.9 (1.4,2.6) | 0.728 |
| VKA vs NOACs | 3.7 (2.3, 5.8) | 2.2 (1.6, 2.9) | 2.5 (2, 3.2) | 0.0571 |
| VKA VS Placebo | 0.072 (0.0024, 0.58) | 0.84 (0.64, 1.1) | 0.80 (0.61, 1.1) | 0.019175 |
| VKA VS Tl | 1.4 (0.89, 2.3) | 2.0 (1.4, 2.8) | 1.7(1.3,2.3) | 0.288025 |
| **Major bleeding during prophylaxis** | | | | |
| LMWH vs AP | 1.1e-14 (2.0e-49, 0.064) | 0.73(0.31,1.7) | 0.61 (0.26, 1.4) | 0.00635 |
| NOACs vs AP | 0.60 (0.11, 3.0) | 0.92 (0.34, 2.3) | 0.84 (0.36, 1.9) | 0.6522 |
| Placebo VS AP | 0.68 (0.26, 1.7) | 0.19 (0.040, 0.79) | 0.47 (0.21, 1.0) | 0.146425 |
| VKAvs AP | 1.3e-17 (3.1e-57, 0.18) | 0.41 (0.16, 1.0) | 0.39 (0.15, 0.94) | 0.036075 |
| NOACs vs LMWH | 1.4 (1.0, 1.8) | 1.4 (0.79, 2.5) | 1 4 (1.1, 1.8) | 0.94345 |
| Placebo VS LMWH | 0.60 (0.40, 0.89) | 1.3 (0.73, 2.4) | 0.77 (0.55, 1.1) | 0.0294 |
| Tl vs LMWH | 1.2 (0.79, 1.7) | 0.99 (0.37, 2.6) | 1.1 (0.79, 1.6) | 0.760925 |
| UFH vs LMWH | 1 5 (1.0，2.3) | 1.1 (0.27, 4.8) | 1.5 (1.0, 2.2) | 0.679775 |
| VKA vs LMWH | 0.79 (0.50, 1.2) | 0.37 (0.17,0.78) | 0.64 (0.43, 0.94) | 0.087675 |
| Placebo VS NOACs | 0.85 (0.47, 1.6) | 0.43 (0.27, 0.68) | 0.56 (0.38, 0.80) | 0.078375 |
| Tl vs NOACs | 4.9e-08 (7.7e-26, 9.9e+19) | 0.82 (0.53, 1.3) | 0.82 (0.54, 1.3) | 0.643775 |

| | | | | |
|---|---|---|---|---|
| UFH vs NOACs | 0.84 (0.20, 3.4) | 1.1(0.71, 1.8) | 1.1(0.71, 1.7) | 0.68035 |
| VKA vs NOACs | 3.3e-11(1.1e-21,0.011) | 0.61 (0.38, 0.98) | 0.47 (0.29, 0.72) | 0 |
| VKA VS Placebo | 7.8e+10(0.96，6.2e+47) | 0.81 (0.48, 1.3) | 0.83 (0.50, 1.4) | 0.044725 |
| VKA VS Tl | 0.63 (0.26, 1.5) | 0.54 (0.30, 0.95) | 0.57 (0.35, 0.91) | 0.77075 |
| **All-cause mortality during prophylaxis** | | | | |
| LMWH vs AP | 0.88 (0.077, 9.5) | 0.63 (0 054, 7 5) | 0.74 (0 13, 4 1) | 0.85265 |
| Placebo vs AP | 1.9 (0.055, 1.2e+02) | 0.055 (0 0065, 046) | 0.055 (0 0065, 046) 0.14 (0022, 085) | 0.09255 |
| VKA vs AP | 0.11 (0.0039, 2.7) | 3.8 (044, 34) | 1.2 (0 20, 7 5) | 0.0704 |
| NOACs vs LMWH | 0.80 (0.42, 1.5) | 0.14 (0039, 048) | 0.56 (0 31, 1 0) | 0.01375 |
| Placebo vs LMWH | 0.028 (0.0064, 0.11) | 0.61 (0 20, 1 9) | 0.18 (0074, 045) | 8.00E-04 |
| TI vs LMWH | 0.20 (0.027, 1.4) | 0.39 (0 041, 3 7) | 0.26 (0 062, 1 1) | 0.65815 |
| VKA vs LMWH | 2.4(0.48, 13) | 1.3 (0.38, 4.8) | 1.6(0.61,4.5) | 0.563525 |
| Placebo VS NOACs | 0.71 (0.25, 2.0) | 0.077 (0.017, 0.32) | 0.33 (0.14,0.77) | 0.01385 |
| VKA vs NOACs | 4.1 (1.0, 18) | 2.1 (0.49, 8.8) | 3.0(1.1,8.0) | 0.50475 |
| VKA vs TI | 4.7 (0 65, 34.0) | 9.1 (0.99, 87) | 6.2 (1.4, 27) | 0.65505 |
| **VTE related death during prophylaxis** | | | | |
| LMWH vs AP | 0.88 (0.077, 9.5) | 0.63 (0 054, 7 5) | 0.74 (0 13, 4 1) | 0.85265 |
| Placebo vs AP | 1.9 (0.055, 1.2e+02) | 0.055 (0 0065, 046) | 0.055 (0 0065, 046) 0.14 (0022, 085) | 0.09255 |
| VKA vs AP | 0.11 (0.0039, 2.7) | 3.8 (044, 34) | 1.2 (0 20, 7 5) | 0.0704 |
| NOACs vs LMWH | 0.80 (0.42, 1.5) | 0.14 (0039, 048) | 0.56 (0 31, 1 0) | 0.01375 |
| Placebo vs LMWH | 0.028 (0.0064, 0.11) | 0.61 (0 20, 1 9) | 0.18 (0074, 045) | 8.00E-04 |
| TI vs LMWH | 0.20 (0.027, 1.4) | 0.39 (0 041, 3 7) | 0.26 (0 062, 1 1) | 0.65815 |
| VKA vs LMWH | 2.4(0.48, 13) | 1.3 (0.38, 4.8) | 1.6(0.61,4.5) | 0.563525 |
| Placebo VS NOACs | 0.71 (0.25, 2.0) | 0.077 (0.017, 0.32) | 0.33 (0.14,0.77) | 0.01385 |
| VKA vs NOACs | 4.1 (1.0, 18) | 2.1 (0.49, 8.8) | 3.0(1.1,8.0) | 0.50475 |
| VKA vs TI | 4.7 (0 65, 34.0) | 9.1 (0.99, 87) | 6.2 (1.4, 27) | 0.65505 |

**5.7 Comparison-adjusted funnel plots**
**Footnote：A :AP；B:LMWH；C：NOACs；D：Placebo；E：TI；F：UFH；G：VKA。**
**Outcome：Recurrent VTE during treatment**

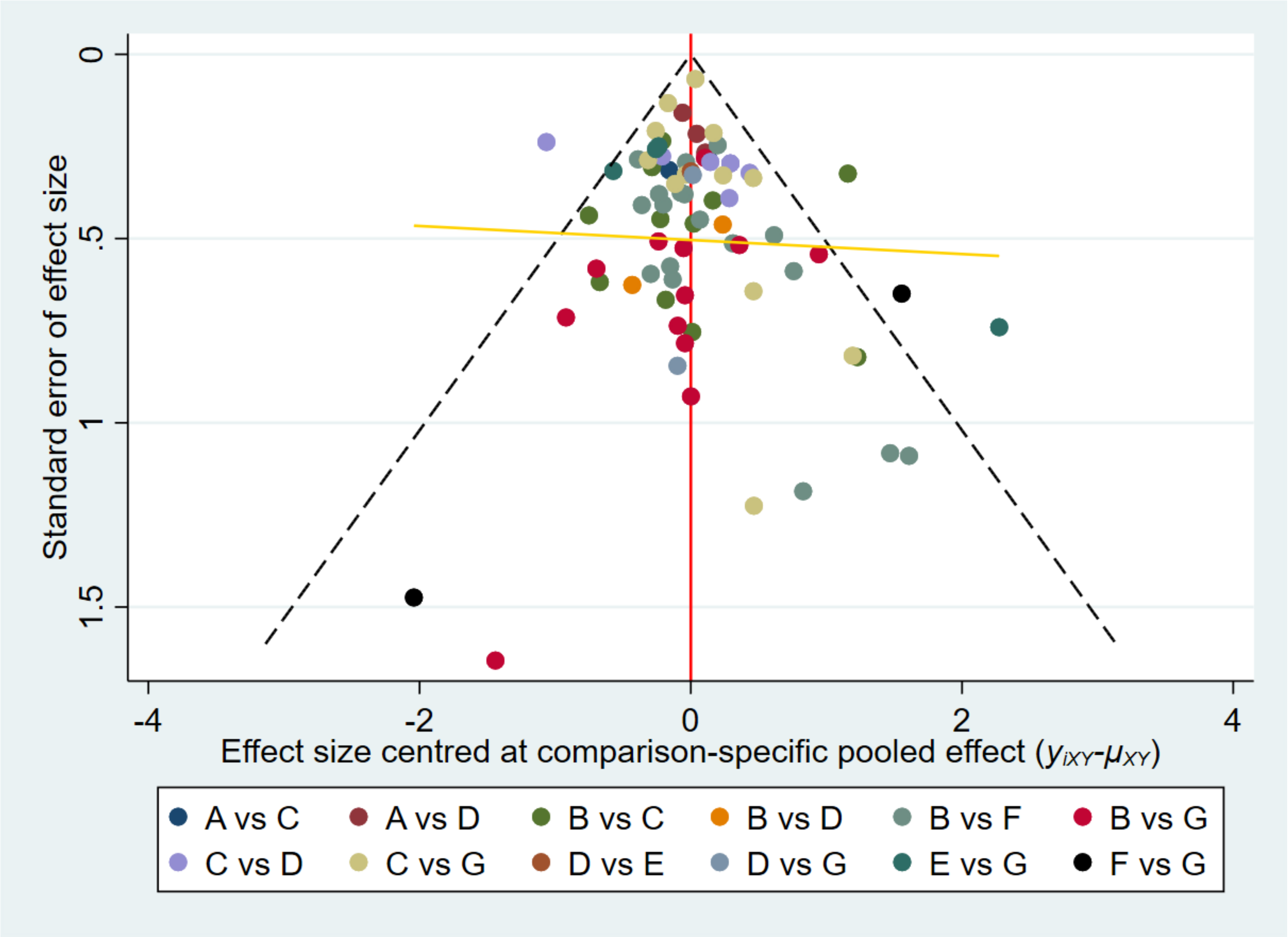

**Outcome：Major bleeding during treatment**

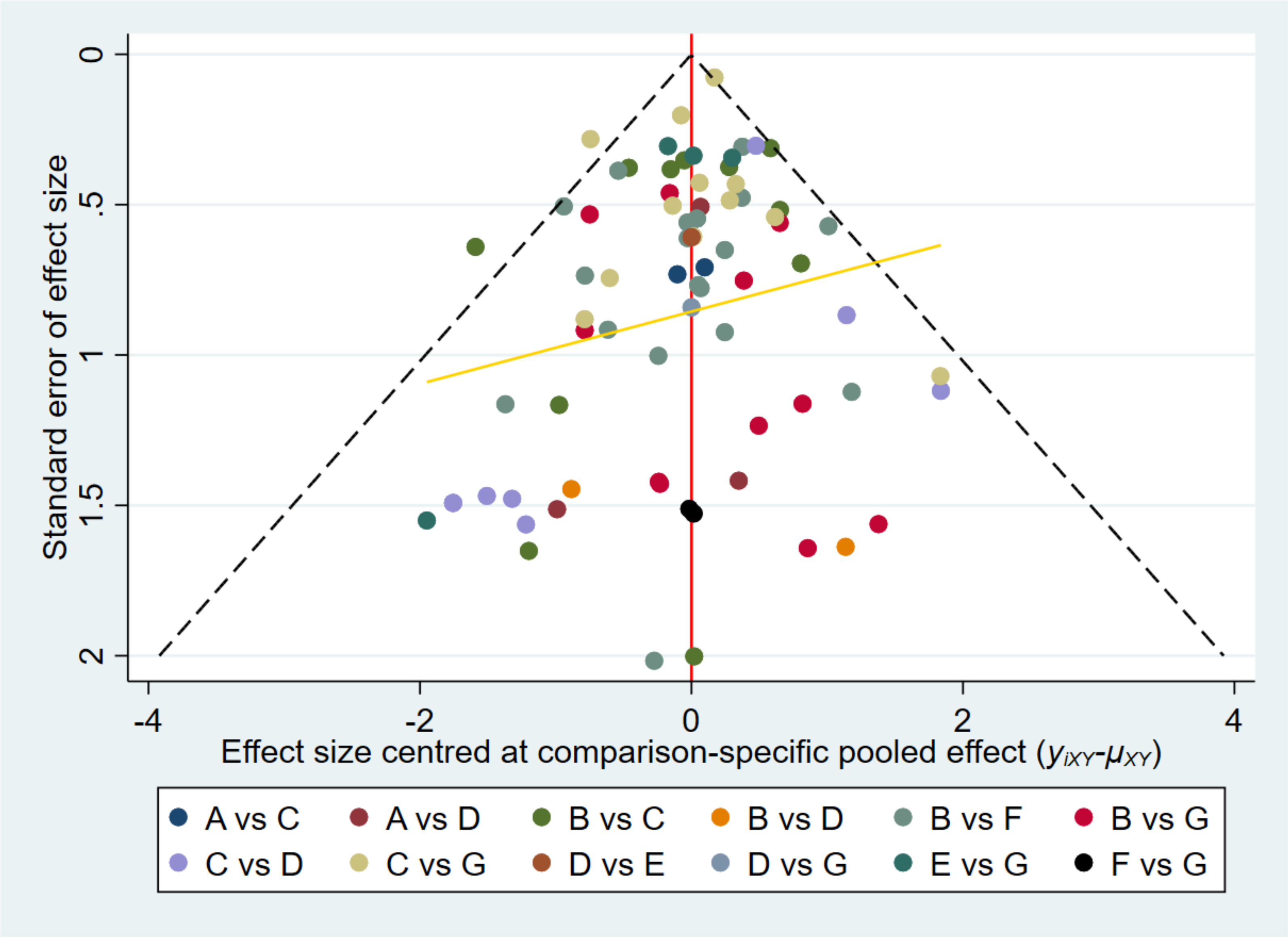

Outcome：Clinical relevant non-major bleeding during treatment

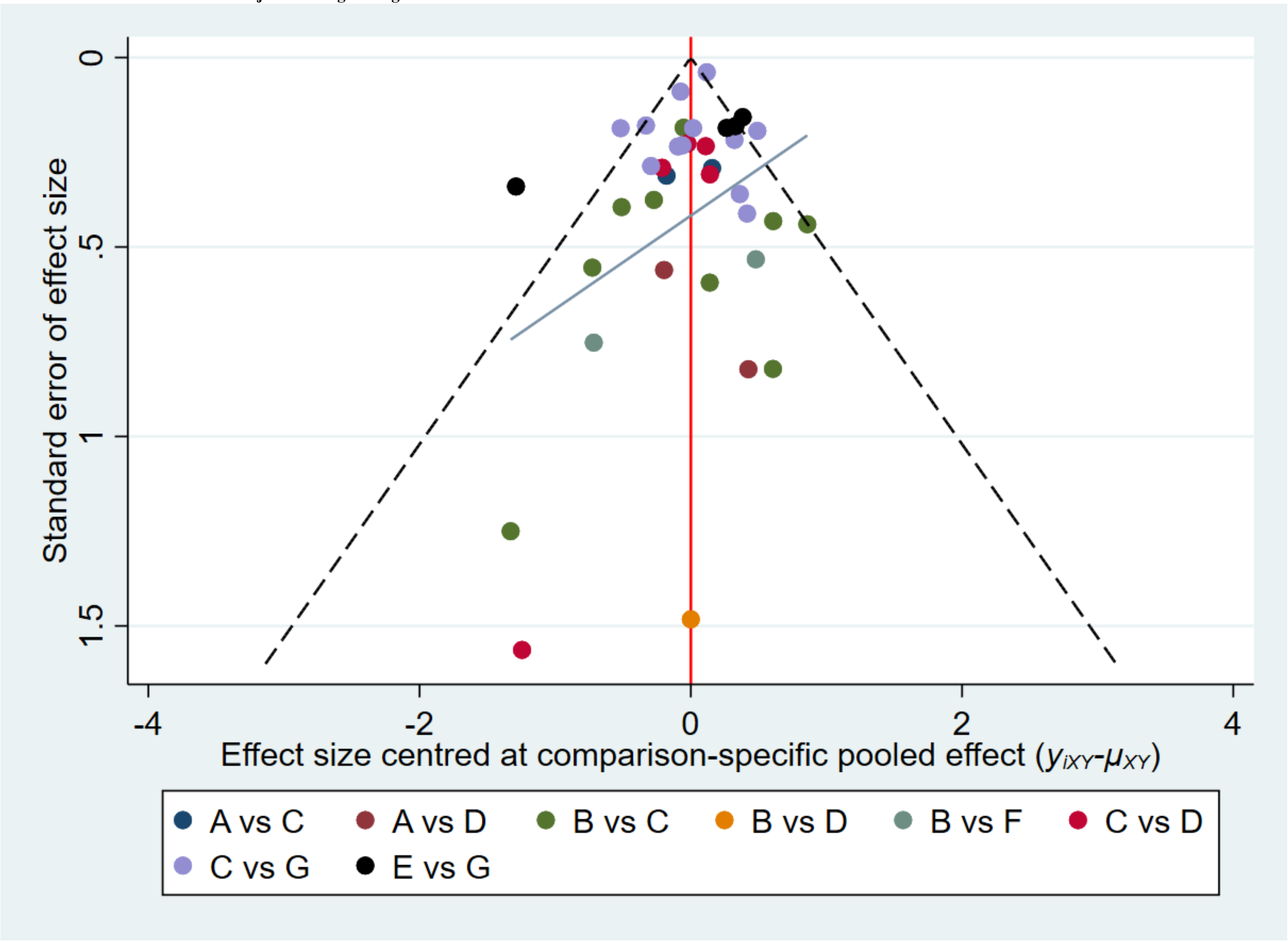

**Outcome：VTE related death during treatment**

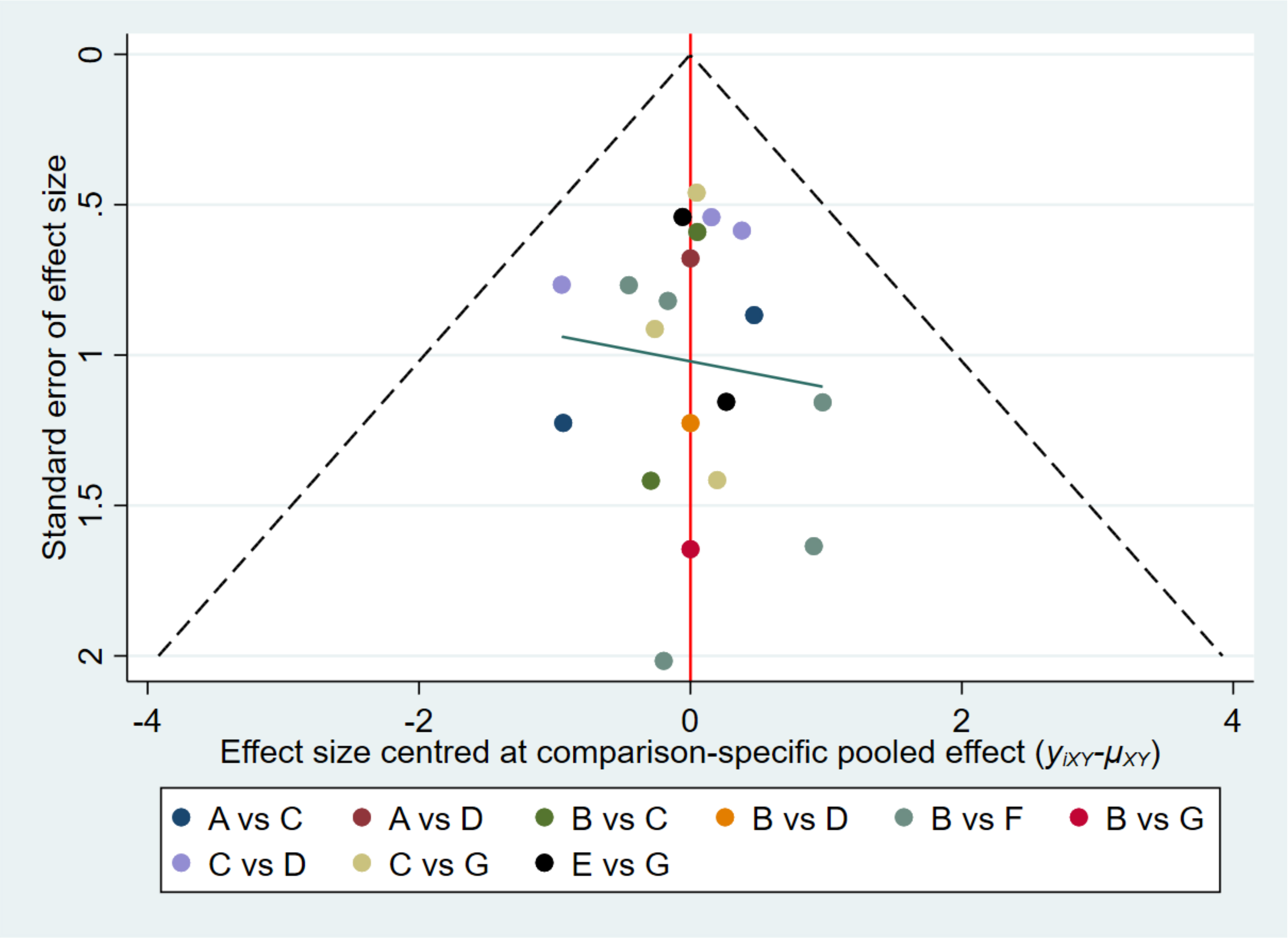

**Outcome：Fatal bleeding during treatment**

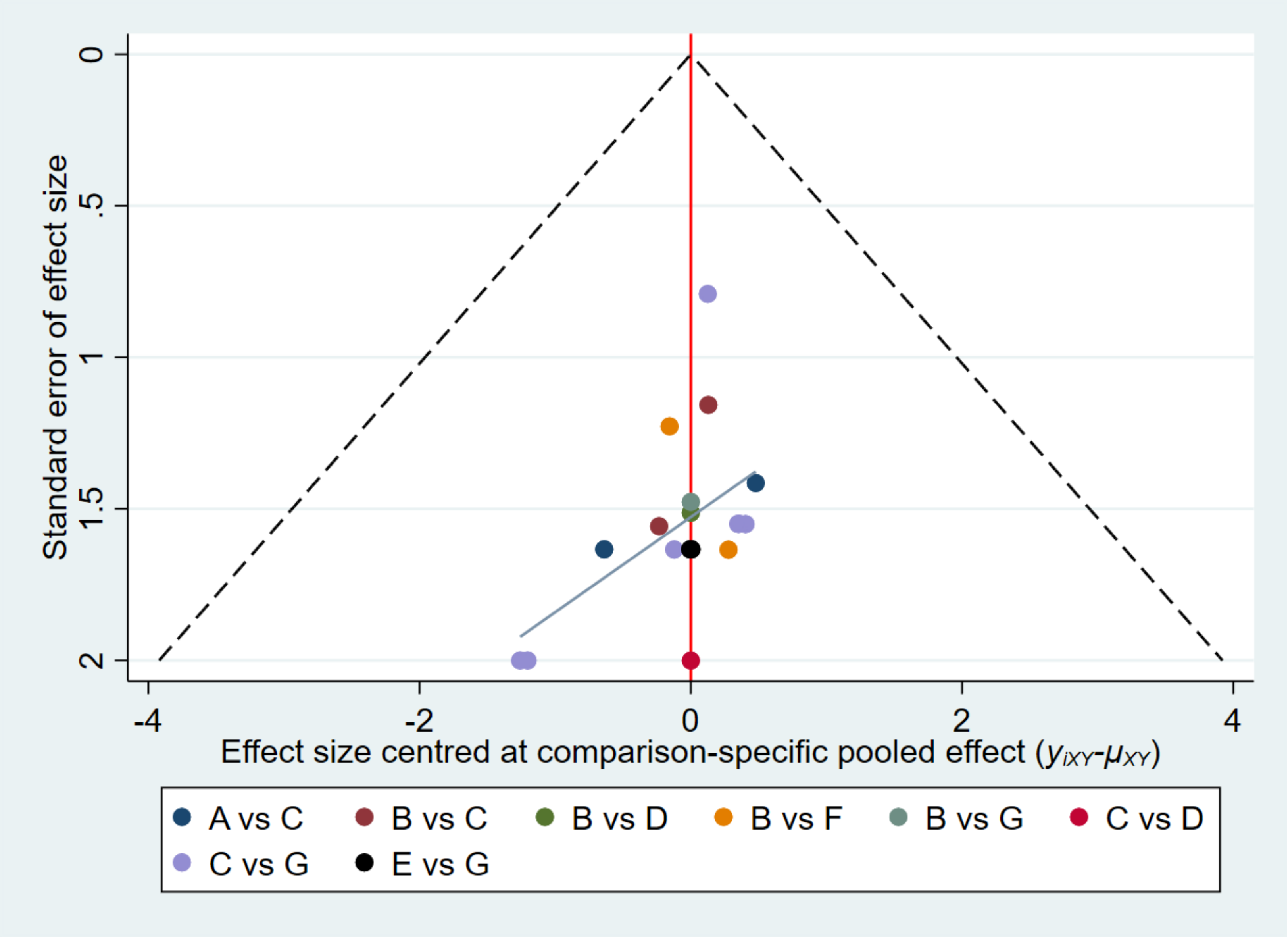

**Outcome：Adverse events during treatment**

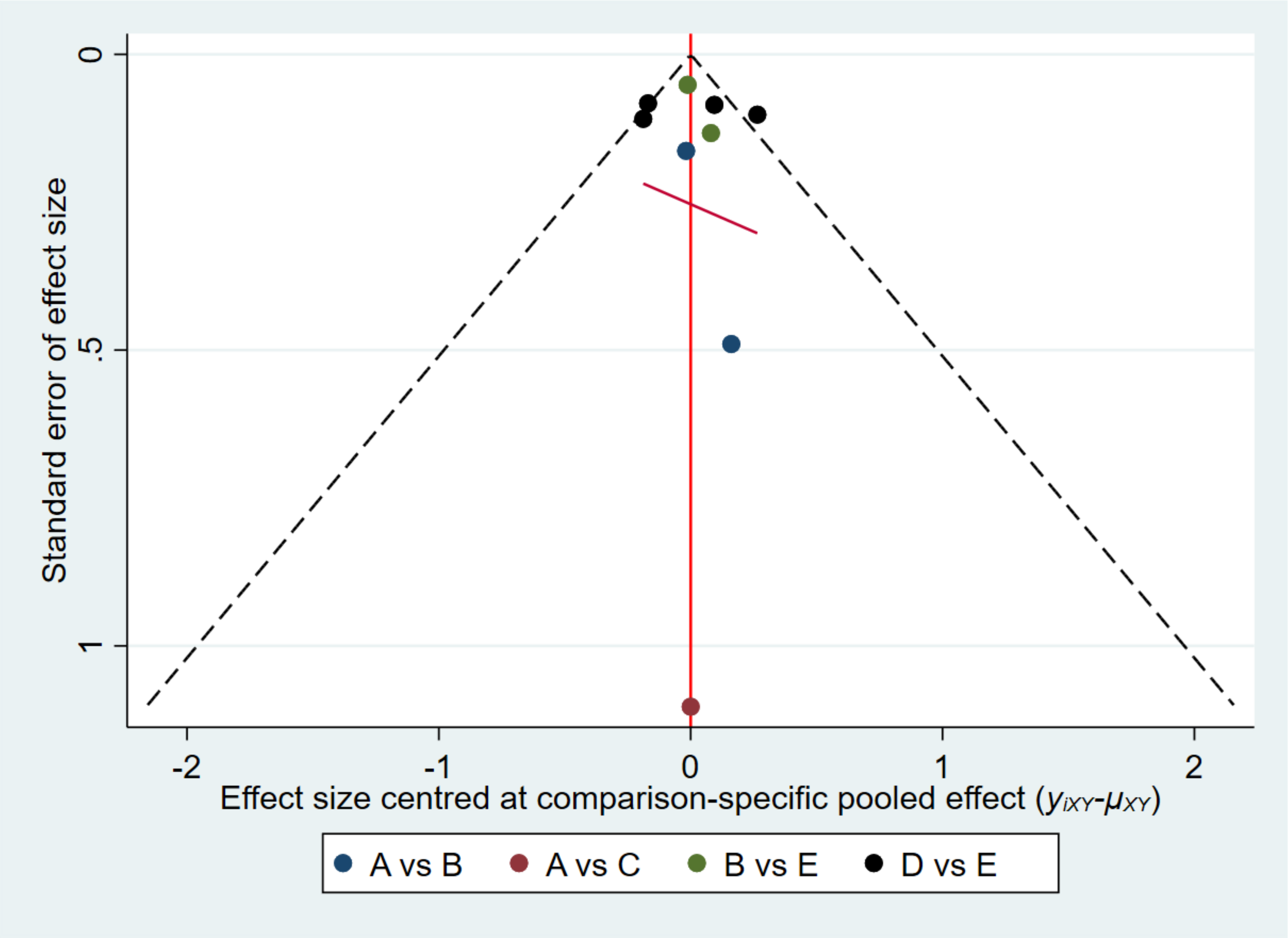


**Footnote: A:LMWH；B：NOACs；C：Placebo；D：TI；E：VKA**

Outcome: All-cause mortality during treatment

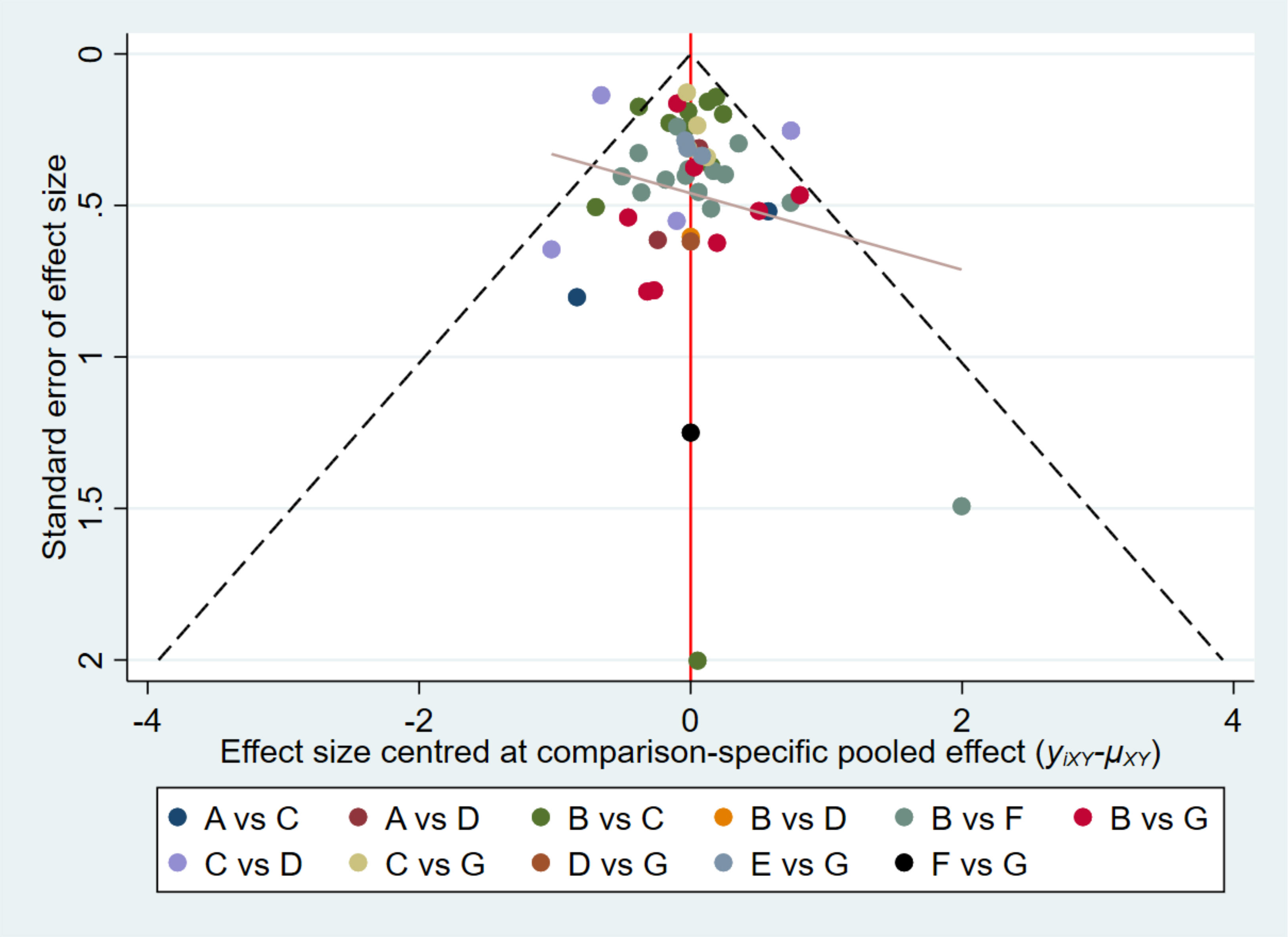

**Outcome: VTE during prophylaxis**

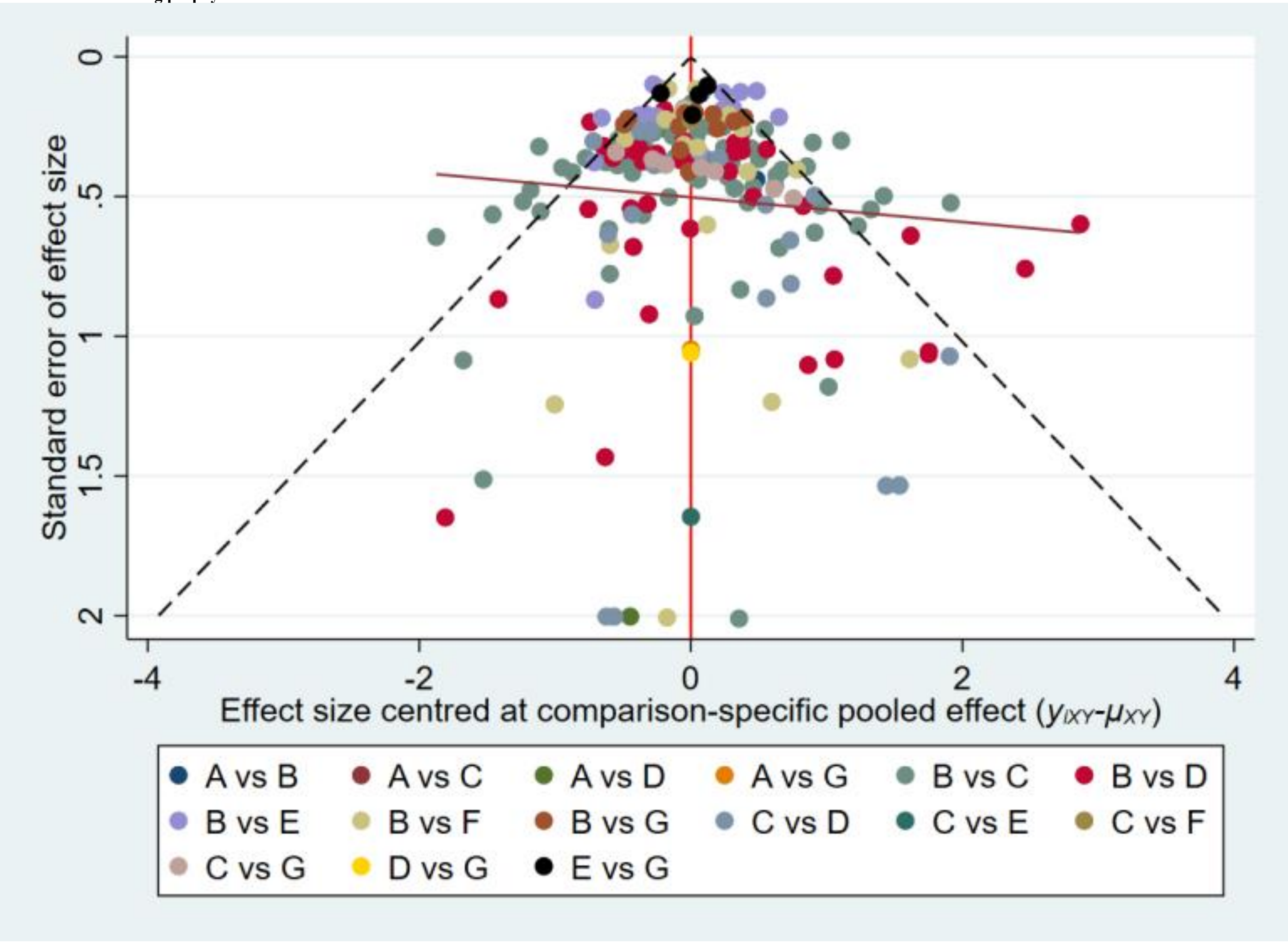

**Outcome: Major bleeding during prophylaxis**

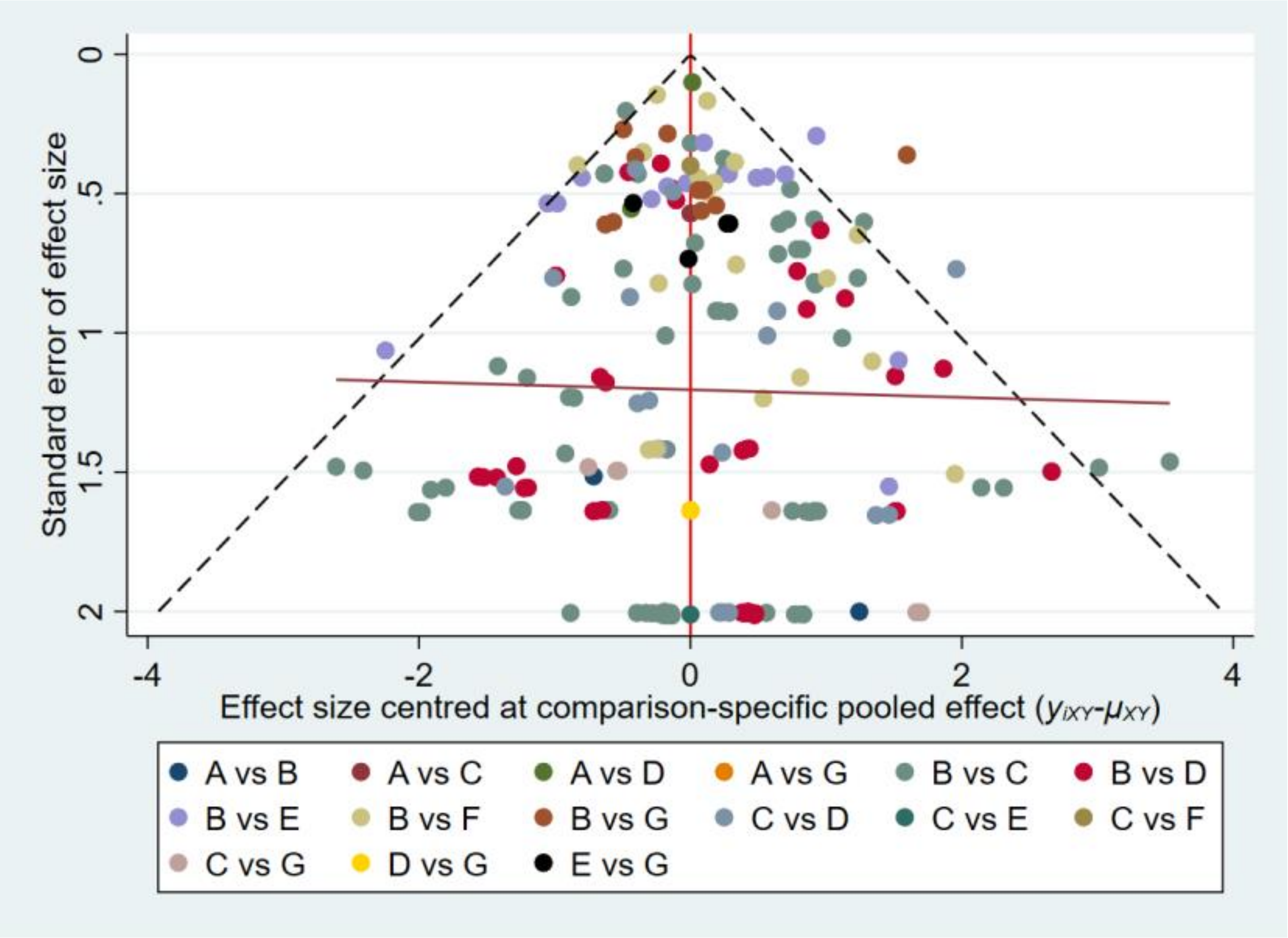

**Outcome: All-cause mortality during prophylaxis**

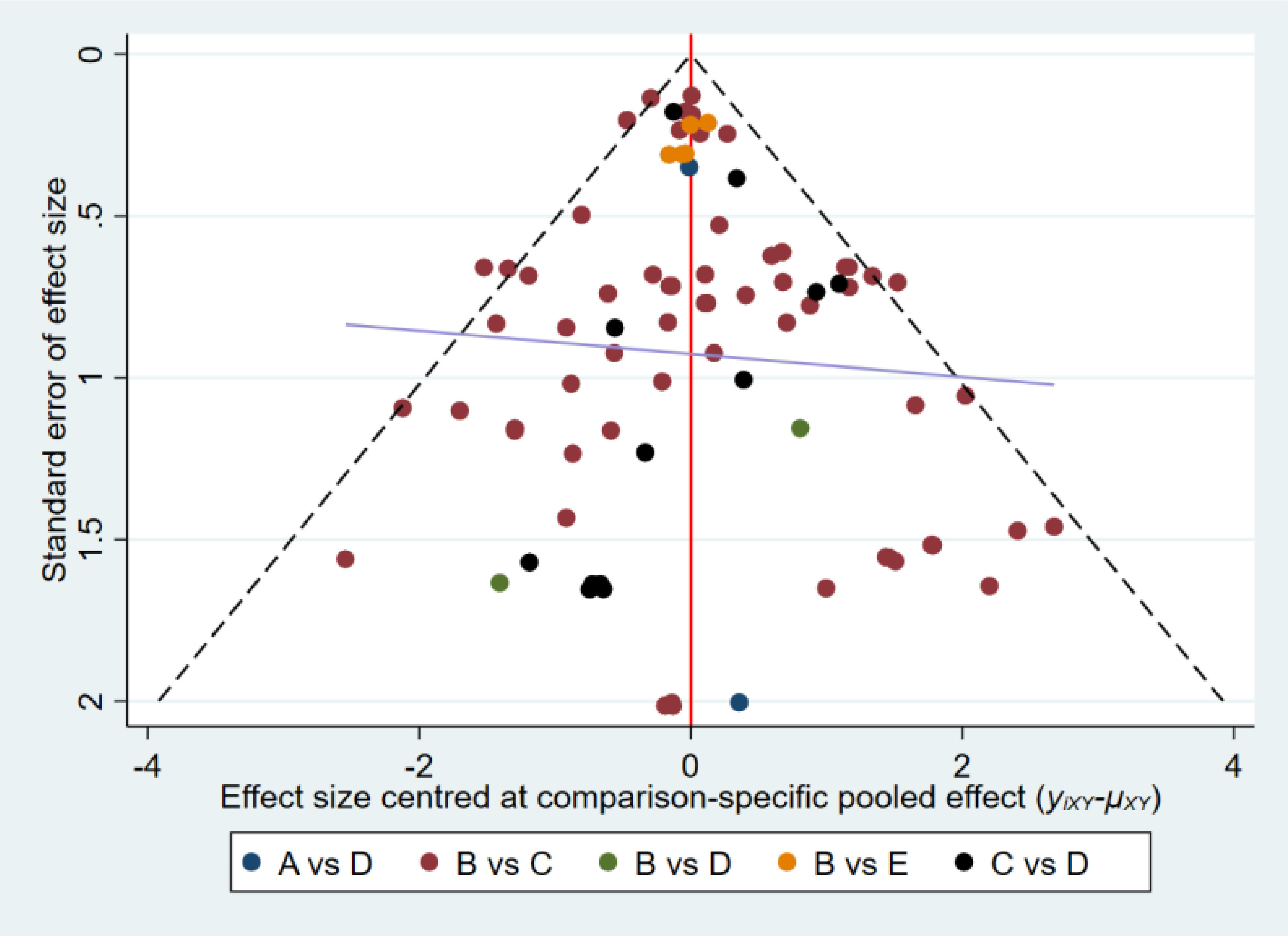

**Outcome: Clinical relevant non-major bleeding during prophylaxis**

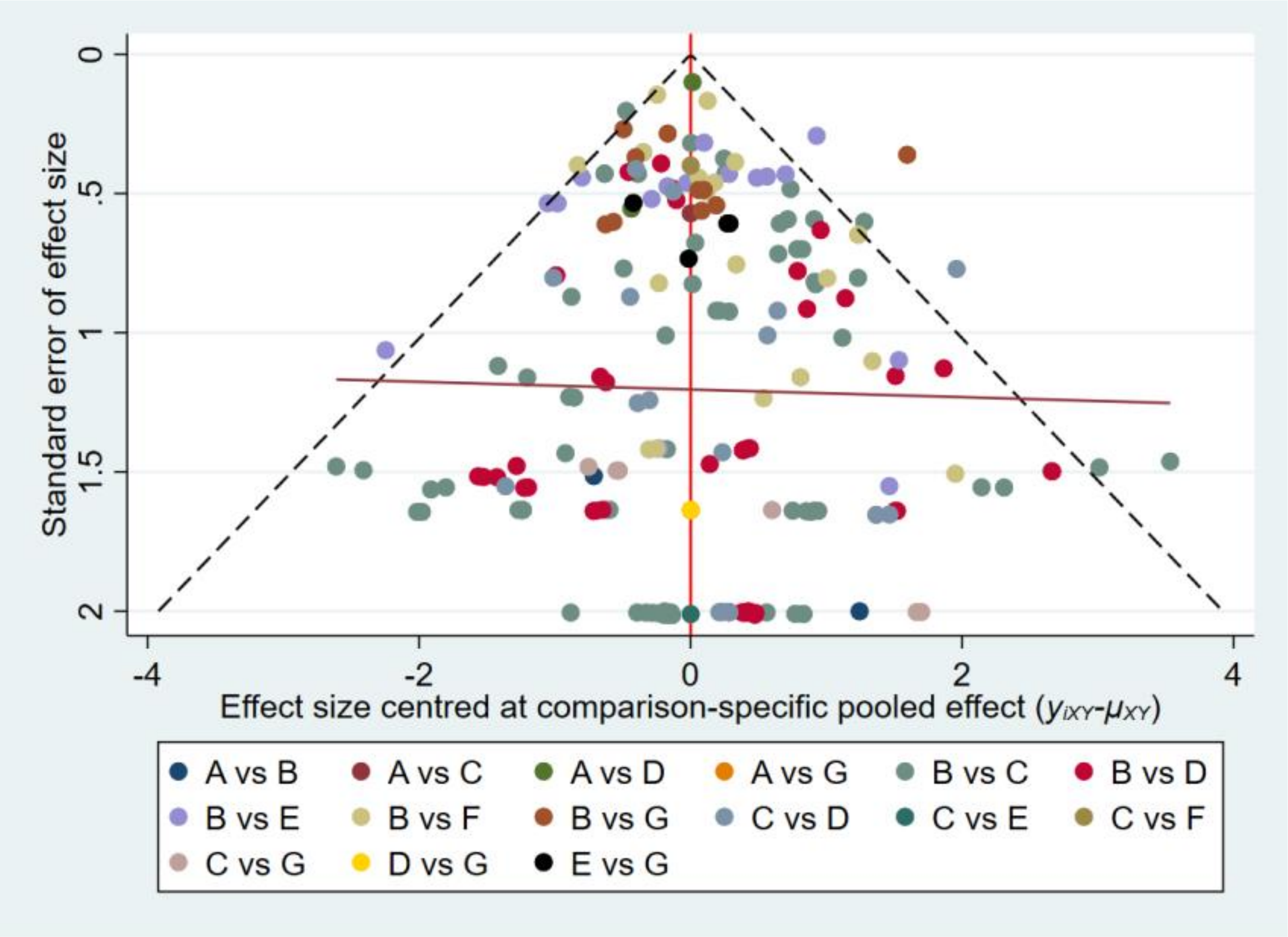

**Outcome：VTE related death during prophylaxis**

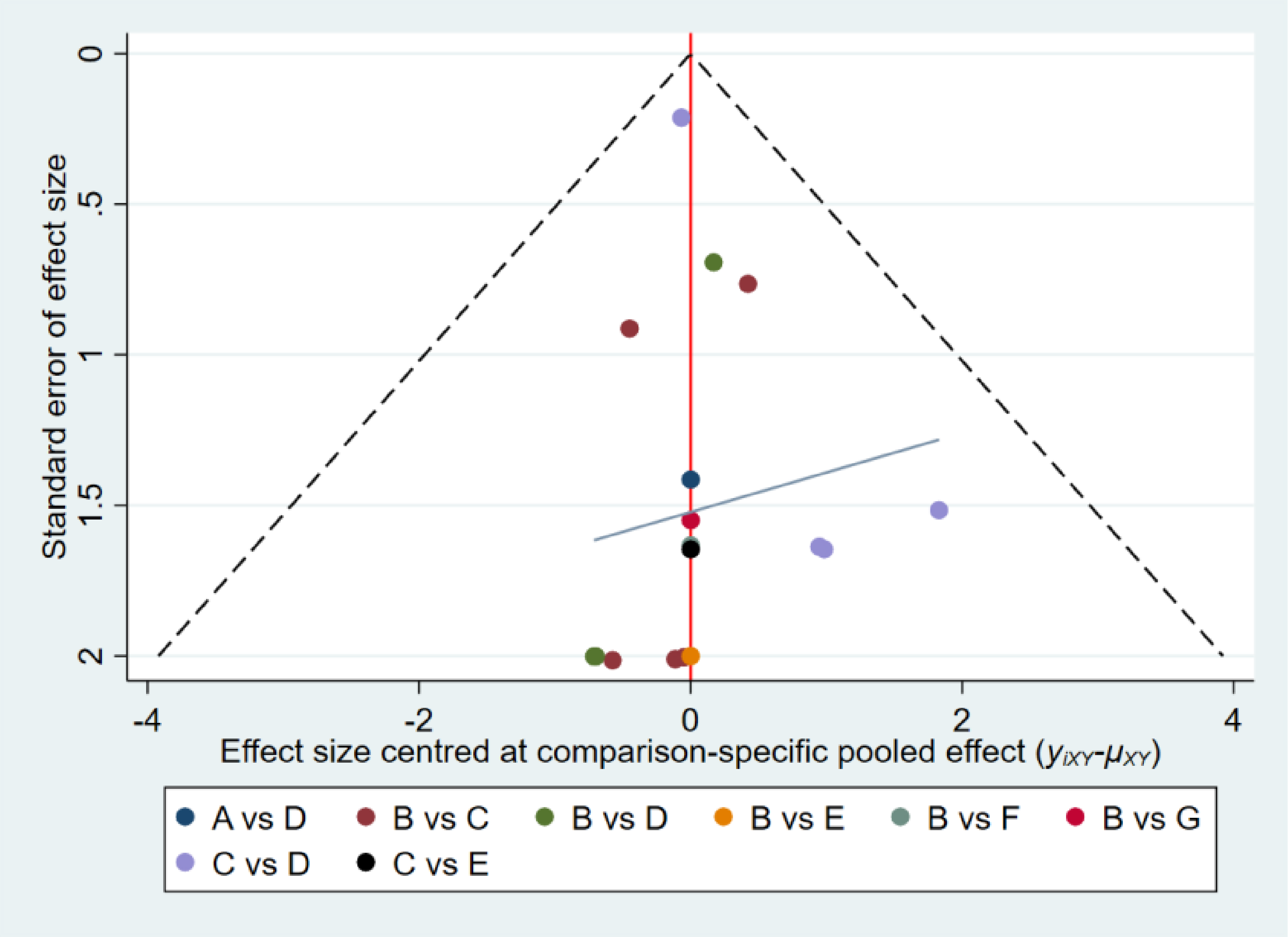

**Regression analysis**

| **Outcome** | **Egger's (P value)** | **Begg's (P value)** |
|---|---|---|
| **Recurrent VTEduring treatment** | **0.38** | **0.55** |
| **Major bleeding during treatment** | **0.16** | **0.75** |
| **Clinical relevant non-major bleeding during treatment** | **0.01** | **0.37** |
| **VTE related death during treatment** | **0.19** | **0.20** |
| **Fatal bleeding during treatment** | **0.51** | **0.18** |
| **Adverse events during treatment** | **0.67** | **0.58** |
| **All-cause mortality during treatment** | **0.94** | **0.96** |
| **VTE during prophylaxis** | **<0.001** | **0.013** |
| **Major bleeding during prophylaxis** | **0.18** | **0.89** |
| **All-cause mortality during prophylaxis** | **0.88** | **0.45** |
| **Clinical relevant non-major bleeding during prophylaxis** | **<0.05** | **0.29** |
| **VTE related death during prophylaxis** | **0.39** | **0.57** |

## 5.8 Subgroup analysis

**Outcome: Recurrent VTE during treatment**

| Study | Experimental Events | Experimental Total | Control Events | Control Total | RR | 95%-CI | Weight |
|---|---|---|---|---|---|---|---|
| t2 = VKA | | | | | | | |
| Raskob-2016 | 49 | 1076 | 48 | 1084 | 1.028 | [0.700; 1.511] | 4.1% |
| Raskob-2016 | 16 | 896 | 17 | 851 | 0.897 | [0.465; 1.729] | 3.5% |
| Raskob-2016 | 1 | 1661 | 2 | 1659 | 0.666 | [0.111; 3.980] | 1.4% |
| Raskob-2018 | 41 | 522 | 59 | 524 | 0.703 | [0.483; 1.023] | 4.1% |
| Medina-2017 | 18 | 601 | 21 | 582 | 0.836 | [0.458; 1.529] | 3.6% |
| Medina-2017 | 26 | 903 | 24 | 906 | 1.084 | [0.634; 1.852] | 3.8% |
| Raskob-2016 | 14 | 378 | 28 | 393 | 0.538 | [0.293; 0.987] | 3.6% |
| Raskob-2016 | 4 | 109 | 7 | 99 | 0.568 | [0.192; 1.680] | 2.5% |
| Raskob-2016 | 2 | 85 | 7 | 77 | 0.340 | [0.094; 1.237] | 2.1% |
| Nakamura-2015 | 16 | 563 | 24 | 538 | 0.650 | [0.355; 1.190] | 3.6% |
| Nakamura-2015 | 114 | 3555 | 122 | 3584 | 0.943 | [0.734; 1.210] | 4.3% |
| Random effects model | | 10349 | | 10297 | 0.840 | [0.723; 0.977] | 36.4% |
| Heterogeneity: $I^2 = 0\%$, $\tau^2 = 0.0011$, $p = 0.55$ | | | | | | | |
| t2 = Placebo | | | | | | | |
| Agnelli-2013 | 14 | 840 | 73 | 829 | 0.200 | [0.116; 0.346] | 3.7% |
| Agnelli-2013 | 14 | 840 | 73 | 829 | 0.200 | [0.116; 0.346] | 3.7% |
| Agnelli-2020 | 33 | 576 | 40 | 579 | 0.834 | [0.537; 1.294] | 4.0% |
| Prins-2018 | 8 | 598 | 42 | 590 | 0.207 | [0.102; 0.420] | 3.3% |
| Prins-2018 | 17 | 1107 | 42 | 590 | 0.223 | [0.130; 0.384] | 3.7% |
| Prins-2018 | 13 | 1127 | 42 | 590 | 0.171 | [0.094; 0.309] | 3.6% |
| Wells-2016 | 18 | 602 | 57 | 594 | 0.323 | [0.195; 0.536] | 3.8% |
| Random effects model | | 5690 | | 4601 | 0.268 | [0.174; 0.413] | 25.9% |
| Heterogeneity: $I^2 = 80\%$, $\tau^2 = 0.2593$, $p < 0.01$ | | | | | | | |
| t2 = LMWH | | | | | | | |
| Planquette-2022 | 4 | 74 | 6 | 84 | 0.810 | [0.268; 2.444] | 2.4% |
| Kang-2019 | 4 | 35 | 4 | 32 | 0.917 | [0.291; 2.884] | 2.3% |
| Schrag-2023 | 20 | 330 | 27 | 308 | 0.700 | [0.406; 1.206] | 3.7% |
| Nisio-2019 | 34 | 522 | 46 | 524 | 0.748 | [0.491; 1.138] | 4.0% |
| Nisio-2019 | 4 | 294 | 8 | 273 | 0.516 | [0.175; 1.521] | 2.5% |
| Wysokinski-2019 | 11 | 224 | 17 | 363 | 1.079 | [0.530; 2.196] | 3.3% |
| Wysokinski-2019 | 7 | 163 | 17 | 363 | 0.986 | [0.438; 2.222] | 3.1% |
| Beyer-2017 | 6 | 236 | 2 | 236 | 2.333 | [0.611; 8.915] | 2.0% |
| Lee-2003 | 37 | 336 | 14 | 336 | 2.533 | [1.421; 4.517] | 3.7% |
| Young-2018 | 8 | 203 | 18 | 203 | 0.474 | [0.220; 1.022] | 3.2% |
| Random effects model | | 2417 | | 2722 | 0.936 | [0.655; 1.339] | 30.3% |
| Heterogeneity: $I^2 = 54\%$, $\tau^2 = 0.1655$, $p = 0.02$ | | | | | | | |
| t2 = AP | | | | | | | |
| Weitz-2017 | 17 | 1107 | 50 | 1131 | 0.361 | [0.212; 0.613] | 3.8% |
| Weitz-2017 | 13 | 1127 | 50 | 1131 | 0.275 | [0.153; 0.495] | 3.6% |
| Random effects model | | 2234 | | 2262 | 0.319 | [0.215; 0.473] | 7.4% |
| Heterogeneity: $I^2 = 0\%$, $\tau^2 = 0$, $p = 0.50$ | | | | | | | |
| **Random effects model** | | **20690** | | **19882** | **0.577** | **[0.447; 0.744]** | **100.0%** |
| Heterogeneity: $I^2 = 81\%$, $\tau^2 = 0.3730$, $p < 0.01$ | | | | | | | |
| Test for subgroup differences: $\chi^2_3 = 42.46$, df = 3 ($p < 0.01$) | | | | | | | |

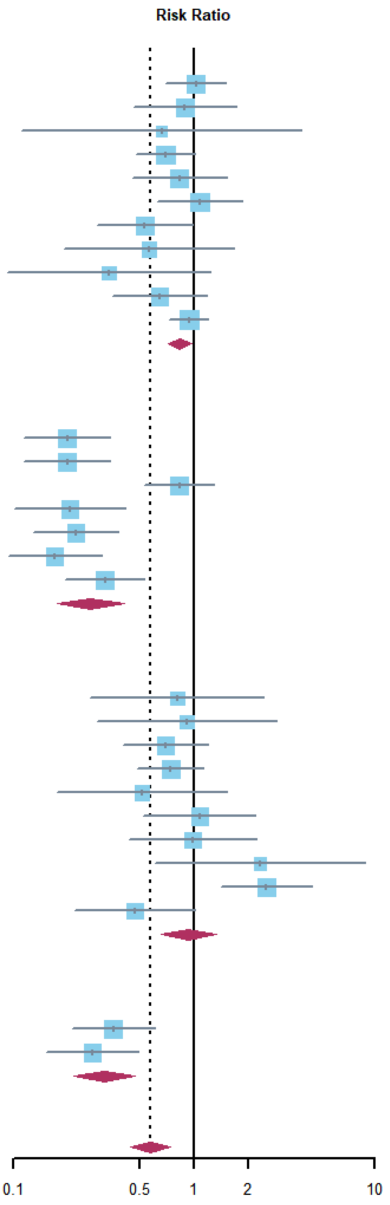

**Outcome：Major bleeding during treatment**

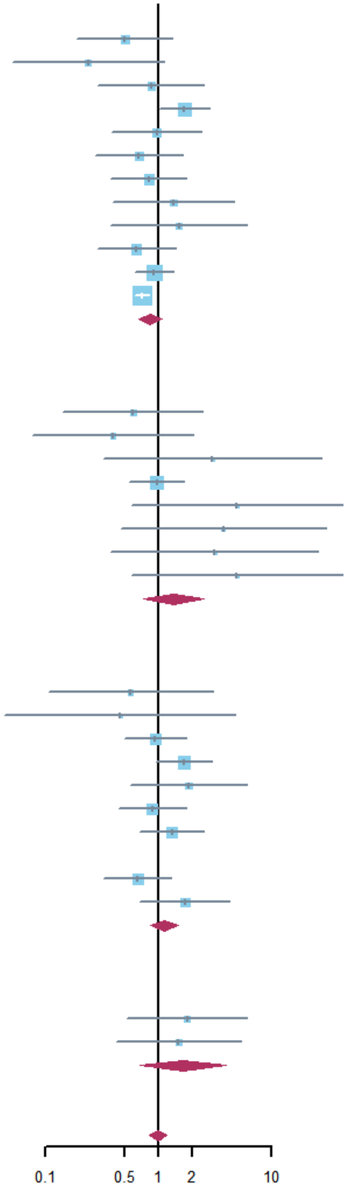


| Study | Experimental Events | Experimental Total | Control Events | Control Total | RR | 95%-CI | Weight |
|---|---|---|---|---|---|---|---|
| t2 = VKA | | | | | | | |
| Raskob-2016 | 5 | 1076 | 11 | 1084 | 0.504 | [0.190; 1.337] | 2.7% |
| Raskob-2016 | 1 | 896 | 7 | 851 | 0.237 | [0.051; 1.115] | 1.2% |
| Raskob-2016 | 5 | 1661 | 6 | 1659 | 0.856 | [0.288; 2.542] | 2.2% |
| Raskob-2018 | 36 | 522 | 21 | 524 | 1.688 | [1.010; 2.822] | 6.7% |
| Medina-2017 | 8 | 601 | 8 | 582 | 0.968 | [0.387; 2.423] | 3.0% |
| Medina-2017 | 7 | 903 | 11 | 906 | 0.669 | [0.275; 1.629] | 3.1% |
| Raskob-2016 | 10 | 378 | 13 | 393 | 0.817 | [0.376; 1.777] | 3.8% |
| Raskob-2016 | 5 | 109 | 3 | 99 | 1.364 | [0.396; 4.693] | 1.8% |
| Raskob-2016 | 4 | 85 | 2 | 77 | 1.512 | [0.373; 6.119] | 1.4% |
| Nakamura-2015 | 9 | 563 | 14 | 538 | 0.637 | [0.289; 1.406] | 3.7% |
| Nakamura-2015 | 47 | 3555 | 52 | 3584 | 0.913 | [0.620; 1.346] | 9.0% |
| Weycker-2018 | 295 | 17878 | 412 | 17878 | 0.717 | [0.618; 0.831] | 14.6% |
| Random effects model | | 28227 | | 28175 | 0.849 | [0.670; 1.076] | 53.2% |
| Heterogeneity: $I^2 = 31\%$, $\tau^2 = 0.0469$, $p = 0.15$ | | | | | | | |
| t2 = Placebo | | | | | | | |
| Agnelli-2013 | 2 | 840 | 4 | 829 | 0.592 | [0.142; 2.470] | 1.4% |
| Agnelli-2013 | 1 | 840 | 4 | 829 | 0.395 | [0.077; 2.029] | 1.1% |
| Marshall-2020 | 2 | 46 | 0 | 46 | 3.000 | [0.324; 27.809] | 0.6% |
| Agnelli-2020 | 22 | 576 | 23 | 579 | 0.963 | [0.550; 1.687] | 6.1% |
| Prins-2018 | 4 | 598 | 0 | 590 | 4.933 | [0.578; 42.099] | 0.6% |
| Prins-2018 | 6 | 1107 | 0 | 590 | 3.734 | [0.460; 30.275] | 0.7% |
| Prins-2018 | 5 | 1127 | 0 | 590 | 3.144 | [0.379; 26.050] | 0.7% |
| Wells-2016 | 4 | 602 | 0 | 594 | 4.934 | [0.578; 42.103] | 0.6% |
| Random effects model | | 5736 | | 4647 | 1.352 | [0.724; 2.523] | 11.7% |
| Heterogeneity: $I^2 = 24\%$, $\tau^2 = 0.1871$, $p = 0.24$ | | | | | | | |
| t2 = LMWH | | | | | | | |
| Planquette-2022 | 1 | 74 | 3 | 84 | 0.567 | [0.107; 3.006] | 1.0% |
| Kang-2019 | 0 | 35 | 1 | 32 | 0.458 | [0.044; 4.823] | 0.5% |
| Schrag-2023 | 17 | 330 | 17 | 308 | 0.934 | [0.495; 1.761] | 5.1% |
| Nisio-2019 | 29 | 522 | 17 | 524 | 1.673 | [0.945; 2.963] | 5.9% |
| Nisio-2019 | 7 | 294 | 3 | 273 | 1.858 | [0.566; 6.100] | 1.9% |
| Wysokinski-2019 | 11 | 224 | 21 | 363 | 0.882 | [0.446; 1.748] | 4.6% |
| Wysokinski-2019 | 12 | 163 | 21 | 363 | 1.312 | [0.678; 2.539] | 4.9% |
| Beyer-2017 | 0 | 236 | 0 | 236 | | | 0.0% |
| Lee-2003 | 12 | 336 | 19 | 336 | 0.650 | [0.329; 1.285] | 4.7% |
| Young-2018 | 11 | 203 | 6 | 203 | 1.714 | [0.689; 4.266] | 3.0% |
| Random effects model | | 2417 | | 2722 | 1.110 | [0.833; 1.478] | 31.6% |
| Heterogeneity: $I^2 = 1\%$, $\tau^2 = 0.0234$, $p = 0.43$ | | | | | | | |
| t2 = AP | | | | | | | |
| Weitz-2017 | 6 | 1107 | 3 | 1131 | 1.788 | [0.525; 6.090] | 1.8% |
| Weitz-2017 | 5 | 1127 | 3 | 1131 | 1.505 | [0.426; 5.320] | 1.7% |
| Random effects model | | 2234 | | 2262 | 1.645 | [0.683; 3.963] | 3.5% |
| Heterogeneity: $I^2 = 0\%$, $\tau^2 = 0$, $p = 0.85$ | | | | | | | |
| **Random effects model** | | **38614** | | **37806** | **0.986** | **[0.829; 1.174]** | **100.0%** |

Heterogeneity: $I^2 = 31\%$, $\tau^2 = 0.0483$, $p = 0.05$
Test for subgroup differences: $\chi^2_3 = 4.45$, df = 3 ($p = 0.22$)

**Outcome：Clinical relevant non-major bleeding during treatment**

| Study | Experimental Events | Experimental Total | Control Events | Control Total | RR | 95%-CI | Weight |
|---|---|---|---|---|---|---|---|
| t2 = VKA | | | | | | | |
| Raskob-2016 | 26 | 1076 | 24 | 1084 | 1.088 | [0.636; 1.862] | 4.0% |
| Raskob-2016 | 37 | 896 | 39 | 851 | 0.902 | [0.585; 1.393] | 4.7% |
| Raskob-2016 | 69 | 1661 | 61 | 1659 | 1.128 | [0.807; 1.576] | 5.4% |
| Raskob-2018 | 76 | 522 | 58 | 524 | 1.310 | [0.954; 1.798] | 5.5% |
| Medina-2017 | 38 | 601 | 42 | 582 | 0.878 | [0.578; 1.334] | 4.8% |
| Medina-2017 | 56 | 903 | 69 | 906 | 0.817 | [0.583; 1.145] | 5.4% |
| Raskob-2016 | 39 | 378 | 64 | 393 | 0.640 | [0.443; 0.924] | 5.2% |
| Raskob-2016 | 16 | 109 | 23 | 99 | 0.644 | [0.368; 1.126] | 3.9% |
| Raskob-2016 | 12 | 85 | 18 | 77 | 0.621 | [0.329; 1.172] | 3.4% |
| Nakamura-2015 | 47 | 563 | 83 | 538 | 0.546 | [0.391; 0.763] | 5.4% |
| Nakamura-2015 | 251 | 3555 | 285 | 3584 | 0.888 | [0.755; 1.045] | 6.5% |
| Random effects model | | 10349 | | 10297 | 0.847 | [0.715; 1.003] | 54.3% |
| Heterogeneity: $I^2 = 54\%$, $\tau^2 = 0.0430$, $p = 0.02$ | | | | | | | |
| t2 = Placebo | | | | | | | |
| Agnelli-2013 | 25 | 840 | 19 | 829 | 1.283 | [0.722; 2.280] | 3.8% |
| Agnelli-2013 | 34 | 813 | 19 | 829 | 1.784 | [1.039; 3.065] | 4.0% |
| Marshall-2020 | 2 | 46 | 0 | 46 | 3.000 | [0.324; 27.809] | 0.5% |
| Agnelli-2020 | 52 | 576 | 35 | 579 | 1.480 | [0.985; 2.224] | 4.9% |
| Wells-2016 | 46 | 608 | 34 | 594 | 1.312 | [0.860; 2.002] | 4.8% |
| Random effects model | | 2883 | | 2877 | 1.454 | [1.151; 1.837] | 17.9% |
| Heterogeneity: $I^2 = 0\%$, $\tau^2 = 0$, $p = 0.85$ | | | | | | | |
| t2 = LMWH | | | | | | | |
| Planquette-2022 | 8 | 74 | 5 | 84 | 1.700 | [0.635; 4.553] | 2.0% |
| Kang-2019 | 1 | 35 | 2 | 32 | 0.611 | [0.109; 3.432] | 0.8% |
| Beyer-2017 | 6 | 236 | 2 | 236 | 2.333 | [0.611; 8.915] | 1.2% |
| Nisio-2019 | 83 | 522 | 56 | 524 | 1.479 | [1.081; 2.025] | 5.6% |
| Nisio-2019 | 14 | 294 | 13 | 273 | 0.995 | [0.489; 2.023] | 3.0% |
| Wysokinski-2019 | 5 | 224 | 10 | 363 | 0.882 | [0.331; 2.353] | 2.0% |
| Wysokinski-2019 | 13 | 163 | 10 | 363 | 2.825 | [1.311; 6.088] | 2.8% |
| Young-2018 | 25 | 203 | 7 | 203 | 3.250 | [1.507; 7.007] | 2.8% |
| Random effects model | | 1751 | | 2078 | 1.621 | [1.152; 2.279] | 20.2% |
| Heterogeneity: $I^2 = 32\%$, $\tau^2 = 0.0732$, $p = 0.17$ | | | | | | | |
| t2 = AP | | | | | | | |
| Weitz-2017 | 30 | 1107 | 20 | 1131 | 1.508 | [0.872; 2.608] | 3.9% |
| Weitz-2017 | 22 | 1127 | 20 | 1131 | 1.099 | [0.612; 1.974] | 3.7% |
| Random effects model | | 2234 | | 2262 | 1.301 | [0.872; 1.941] | 7.6% |
| Heterogeneity: $I^2 = 0\%$, $\tau^2 = 0$, $p = 0.44$ | | | | | | | |
| **Random effects model** | | **17217** | | **17514** | **1.098** | **[0.931; 1.294]** | **100.0%** |

Heterogeneity: $I^2 = 64\%$, $\tau^2 = 0.1005$, $p < 0.01$

Test for subgroup differences: $\chi^2_3 = 20.27$, df = 3 ($p < 0.01$)

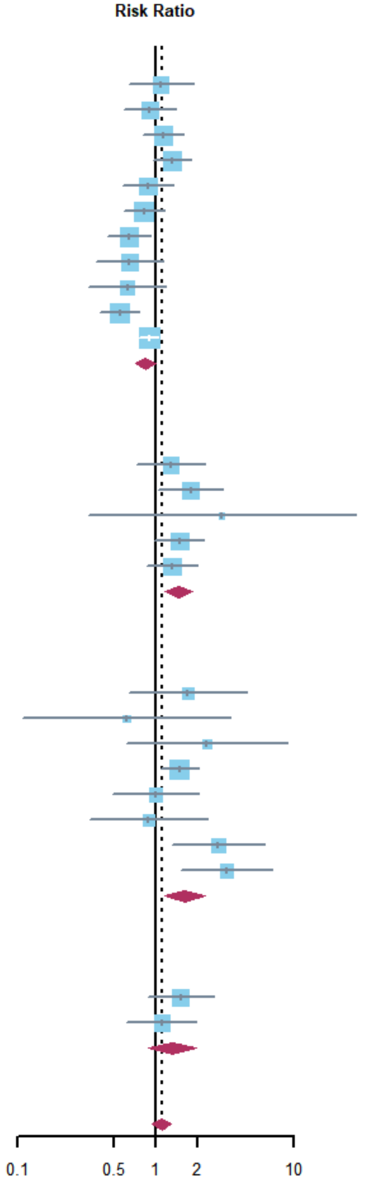

**Outcome: VTE related death during treatment**

| Study | Experimental Events | Experimental Total | Control Events | Control Total | RR | 95%-CI | Weight |
|---|---|---|---|---|---|---|---|
| t2 = VKA | | | | | | | |
| Raskob-2016 | 10 | 3594 | 9 | 3594 | 1.11 | [0.45; 2.73] | 19.1% |
| Nakamura-2015 | 1 | 563 | 1 | 538 | 0.96 | [0.06; 15.24] | 2.2% |
| Nakamura-2015 | 3 | 3555 | 2 | 3584 | 1.51 | [0.25; 9.04] | 4.2% |
| Common effect model | | 7712 | | 7716 | 1.16 | [0.54; 2.51] | 25.5% |
| Heterogeneity: $I^2 = 0\%$, $\tau^2 = 0$, $p = 0.95$ | | | | | | | |
| t2 = Placebo | | | | | | | |
| Agnelli-2013 | 4 | 840 | 11 | 829 | 0.36 | [0.11; 1.12] | 23.5% |
| Agnelli-2013 | 5 | 840 | 11 | 829 | 0.45 | [0.16; 1.29] | 23.5% |
| Agnelli-2020 | 4 | 576 | 3 | 579 | 1.34 | [0.30; 5.96] | 6.3% |
| Common effect model | | 2256 | | 2237 | 0.52 | [0.26; 1.00] | 53.3% |
| Heterogeneity: $I^2 = 1\%$, $\tau^2 = < 0.0001$, $p = 0.36$ | | | | | | | |
| t2 = AP | | | | | | | |
| Weitz-2017 | 4 | 1107 | 2 | 1131 | 2.04 | [0.38; 11.13] | 4.2% |
| Weitz-2017 | 1 | 1127 | 2 | 1131 | 0.50 | [0.05; 5.53] | 4.2% |
| Common effect model | | 2234 | | 2262 | 1.27 | [0.34; 4.71] | 8.4% |
| Heterogeneity: $I^2 = 0\%$, $\tau^2 = 0$, $p = 0.35$ | | | | | | | |
| t2 = LMWH | | | | | | | |
| Lee-2003 | 7 | 336 | 5 | 336 | 1.40 | [0.45; 4.37] | 10.6% |
| Young-2018 | 1 | 203 | 1 | 203 | 1.00 | [0.06; 15.88] | 2.1% |
| Common effect model | | 539 | | 539 | 1.33 | [0.47; 3.81] | 12.7% |
| Heterogeneity: $I^2 = 0\%$, $\tau^2 = 0$, $p = 0.83$ | | | | | | | |
| **Common effect model** | | **12741** | | **12754** | **0.85** | **[0.56; 1.29]** | **100.0%** |
| Heterogeneity: $I^2 = 0\%$, $\tau^2 = 0.0138$, $p = 0.67$ | | | | | | | |
| Test for subgroup differences: $\chi^2_3 = 3.87$, df = 3 ($p = 0.28$) | | | | | | | |

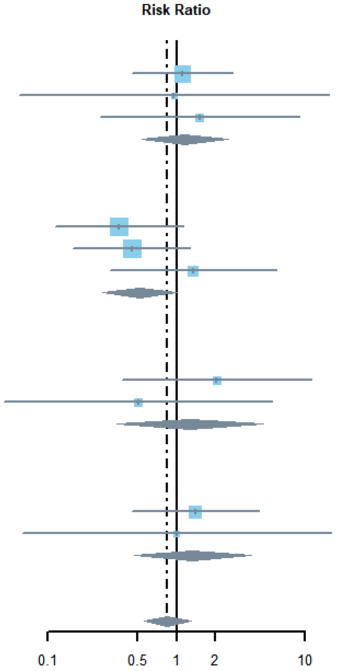


**Outcome: Fatal bleeding during treatment**

| Study | Experimental Events | Experimental Total | Control Events | Control Total | RR | 95%-CI | Weight |
|---|---|---|---|---|---|---|---|
| t2 = VKA | | | | | | | |
| Raskob-2016 | 0 | 1076 | 2 | 1084 | 0.20 | [0.01; 4.19] | 10.0% |
| Raskob-2016 | 0 | 896 | 0 | 851 | | | 0.0% |
| Raskob-2016 | 0 | 1661 | 2 | 1659 | 0.20 | [0.01; 4.16] | 10.0% |
| Medina-2017 | 0 | 601 | 1 | 582 | 0.32 | [0.01; 7.91] | 6.1% |
| Medina-2017 | 0 | 903 | 0 | 906 | | | 0.0% |
| Nakamura-2015 | 0 | 563 | 2 | 538 | 0.19 | [0.01; 3.97] | 10.3% |
| Nakamura-2015 | 2 | 3555 | 8 | 3584 | 0.25 | [0.05; 1.19] | 32.0% |
| Common effect model | | 9255 | | 9204 | 0.23 | [0.08; 0.70] | 68.4% |
| Heterogeneity: $I^2 = 0\%$, $\tau^2 = 0$, $p = 1.00$ | | | | | | | |
| t2 = LMWH | | | | | | | |
| Planquette-2022 | 0 | 74 | 2 | 84 | 0.23 | [0.01; 4.65] | 9.4% |
| Buller-2011 | 1 | 385 | 3 | 370 | 0.32 | [0.03; 3.07] | 12.3% |
| Common effect model | | 459 | | 454 | 0.28 | [0.05; 1.70] | 21.7% |
| Heterogeneity: $I^2 = 0\%$, $\tau^2 = 0$, $p = 0.86$ | | | | | | | |
| t2 = Placebo | | | | | | | |
| Wells-2016 | 0 | 602 | 0 | 594 | | | 0.0% |
| t2 = AP | | | | | | | |
| Weitz-2017 | 1 | 1107 | 1 | 1131 | 1.02 | [0.06; 16.31] | 4.0% |
| Weitz-2017 | 0 | 1127 | 1 | 1131 | 0.33 | [0.01; 8.20] | 6.0% |
| Common effect model | | 2234 | | 2262 | 0.61 | [0.08; 4.59] | 10.0% |
| Heterogeneity: $I^2 = 0\%$, $\tau^2 = 0$, $p = 0.61$ | | | | | | | |
| **Common effect model** | | **12550** | | **12514** | **0.28** | **[0.12; 0.65]** | **100.0%** |
| Heterogeneity: $I^2 = 0\%$, $\tau^2 = 0$, $p = 1.00$ | | | | | | | |
| Test for subgroup differences: $\chi^2_2 = 0.66$, df = 2 ($p = 0.72$) | | | | | | | |

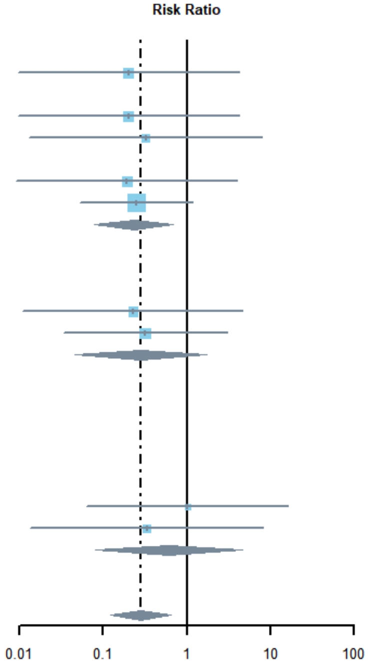

**Outcome： Adverse events during treatment**

| Study | Experimental Events | Experimental Total | Control Events | Control Total | RR | 95%-CI | Weight |
|---|---|---|---|---|---|---|---|
| t2 = LMWH | | | | | | | |
| Kang-2019 | 16 | 35 | 14 | 32 | 1.04 | [0.61; 1.78] | 0.5% |
| Schrag-2023 | 120 | 330 | 120 | 308 | 0.93 | [0.76; 1.14] | 4.0% |
| Common effect model | | 365 | | 340 | 0.95 | [0.78; 1.14] | 4.5% |
| Heterogeneity: $I^2 = 0\%$, $\tau^2 = 0$, $p = 0.70$ | | | | | | | |
| t2 = VKA | | | | | | | |
| Nakamura-2015 | 387 | 563 | 392 | 538 | 0.94 | [0.87; 1.02] | 13.1% |
| Nakamura-2015 | 2434 | 3555 | 2536 | 3584 | 0.97 | [0.94; 1.00] | 82.4% |
| Common effect model | | 4118 | | 4122 | 0.96 | [0.94; 0.99] | 95.5% |
| Heterogeneity: $I^2 = 0\%$, $\tau^2 = 0$, $p = 0.54$ | | | | | | | |
| **Common effect model** | | **4483** | | **4462** | **0.96** | **[0.94; 0.99]** | **100.0%** |
| Heterogeneity: $I^2 = 0\%$, $\tau^2 = 0$, $p = 0.91$ | | | | | | | |
| Test for subgroup differences: $\chi^2_1 = 0.04$, df = 1 ($p = 0.84$) | | | | | | | |

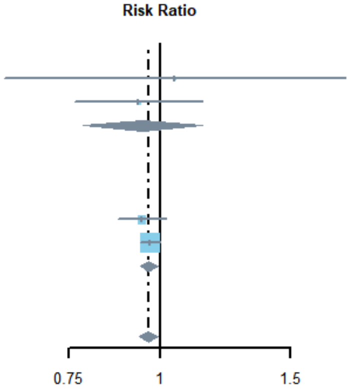


**Outcome: All-cause mortality during treatment**

| Study | Experimental Events | Experimental Total | Control Events | Control Total | RR | 95%-CI | Weight |
|---|---|---|---|---|---|---|---|
| t2 = VKA | | | | | | | |
| Raskob-2018 | 206 | 522 | 192 | 524 | 1.077 | [0.923; 1.256] | 6.6% |
| Raskob-2016 | 40 | 378 | 40 | 393 | 1.040 | [0.690; 1.565] | 5.4% |
| Raskob-2016 | 31 | 109 | 26 | 99 | 1.077 | [0.698; 1.664] | 5.3% |
| Raskob-2016 | 26 | 85 | 24 | 77 | 0.980 | [0.625; 1.535] | 5.2% |
| Random effects model | | 1094 | | 1093 | 1.064 | [0.934; 1.213] | 22.5% |
| Heterogeneity: $I^2 = 0\%$, $\tau^2 = 0$, $p = 0.98$ | | | | | | | |
| t2 = Placebo | | | | | | | |
| Agnelli-2013 | 20 | 840 | 86 | 829 | 0.238 | [0.149; 0.380] | 5.1% |
| Agnelli-2013 | 20 | 840 | 86 | 829 | 0.238 | [0.149; 0.380] | 5.1% |
| Marshall-2020 | 6 | 46 | 5 | 46 | 1.167 | [0.424; 3.212] | 2.6% |
| Agnelli-2020 | 135 | 576 | 153 | 579 | 0.888 | [0.727; 1.084] | 6.4% |
| Wells-2016 | 5 | 602 | 10 | 594 | 0.538 | [0.200; 1.446] | 2.7% |
| Random effects model | | 2904 | | 2877 | 0.479 | [0.248; 0.925] | 22.0% |
| Heterogeneity: $I^2 = 91\%$, $\tau^2 = 0.4553$, $p < 0.01$ | | | | | | | |
| t2 = LMWH | | | | | | | |
| Planquette-2022 | 19 | 74 | 20 | 84 | 1.079 | [0.637; 1.830] | 4.8% |
| Schrag-2023 | 71 | 330 | 57 | 308 | 1.159 | [0.851; 1.579] | 6.0% |
| Nisio-2019 | 140 | 522 | 127 | 524 | 1.106 | [0.900; 1.359] | 6.4% |
| Nisio-2019 | 39 | 294 | 39 | 273 | 0.929 | [0.619; 1.394] | 5.4% |
| Buller-2011 | 6 | 385 | 12 | 370 | 0.518 | [0.209; 1.283] | 3.0% |
| Wysokinski-2019 | 82 | 224 | 171 | 363 | 0.781 | [0.638; 0.956] | 6.4% |
| Wysokinski-2019 | 74 | 163 | 171 | 363 | 0.968 | [0.793; 1.181] | 6.4% |
| Beyer-2017 | 0 | 236 | 0 | 236 | | | 0.0% |
| Lee-2003 | 136 | 336 | 130 | 336 | 1.046 | [0.868; 1.259] | 6.5% |
| Young-2018 | 48 | 203 | 56 | 203 | 0.860 | [0.619; 1.194] | 5.9% |
| Random effects model | | 2767 | | 3060 | 0.968 | [0.869; 1.078] | 50.8% |
| Heterogeneity: $I^2 = 23\%$, $\tau^2 = 0.0069$, $p = 0.24$ | | | | | | | |
| t2 = AP | | | | | | | |
| Weitz-2017 | 8 | 1107 | 7 | 1131 | 1.149 | [0.445; 2.968] | 2.9% |
| Weitz-2017 | 2 | 1127 | 7 | 1131 | 0.376 | [0.100; 1.415] | 1.8% |
| Random effects model | | 2234 | | 2262 | 0.727 | [0.248; 2.132] | 4.7% |
| Heterogeneity: $I^2 = 45\%$, $\tau^2 = 0.2778$, $p = 0.18$ | | | | | | | |
| **Random effects model** | | **8999** | | **9292** | **0.819** | **[0.663; 1.010]** | **100.0%** |
| Heterogeneity: $I^2 = 78\%$, $\tau^2 = 0.1687$, $p < 0.01$ | | | | | | | |
| Test for subgroup differences: $\chi^2_3 = 6.33$, df = 3 ($p = 0.10$) | | | | | | | |

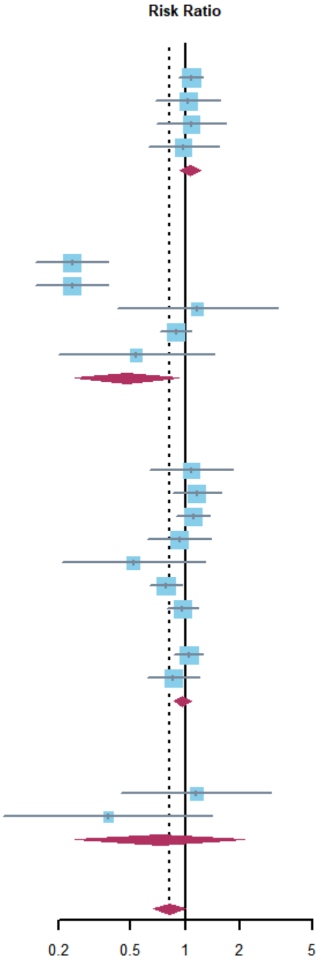

**Outcome: VTE during prophylaxis**

| Study | Experimental Events | Experimental Total | Control Events | Control Total | RR | 95%-CI | Weight |
|---|---|---|---|---|---|---|---|
| t2 = Placebo | | | | | | | |
| Wang-2023 | 5 | 610 | 5 | 607 | 0.995 | [0.323; 3.068] | 0.6% |
| Vadhan-2020 | 13 | 135 | 18 | 138 | 0.753 | [0.394; 1.441] | 1.2% |
| Becattini-2022 | 3 | 287 | 11 | 282 | 0.328 | [0.107; 1.004] | 0.6% |
| Ramacciotti-2022 | 5 | 159 | 15 | 159 | 0.375 | [0.151; 0.934] | 0.8% |
| Ageno-2021 | 25 | 3853 | 32 | 3872 | 0.792 | [0.474; 1.321] | 1.4% |
| Ageno-2021 | 25 | 2154 | 34 | 2140 | 0.738 | [0.446; 1.222] | 1.4% |
| Levine-2012 | 0 | 32 | 3 | 29 | 0.227 | [0.027; 1.922] | 0.2% |
| Levine-2012 | 0 | 29 | 3 | 29 | 0.250 | [0.030; 2.108] | 0.2% |
| Levine-2012 | 0 | 32 | 3 | 29 | 0.227 | [0.027; 1.922] | 0.2% |
| Eriksson-2008 | 33 | 109 | 29 | 103 | 1.072 | [0.710; 1.617] | 1.6% |
| Spyropoulos-2018 | 11 | 6007 | 25 | 6012 | 0.462 | [0.233; 0.915] | 1.1% |
| Zhang-2021 | 6 | 237 | 7 | 232 | 0.857 | [0.316; 2.324] | 0.7% |
| Zhang-2021 | 2 | 51 | 5 | 43 | 0.423 | [0.112; 1.594] | 0.5% |
| Brandt-2022 | 6 | 126 | 17 | 91 | 0.282 | [0.123; 0.647] | 0.9% |
| Connors-2021 | 0 | 135 | 0 | 136 | | | 0.0% |
| Connors-2021 | 0 | 143 | 0 | 136 | | | 0.0% |
| Camporese-2016 | 2 | 120 | 7 | 114 | 0.356 | [0.097; 1.311] | 0.5% |
| Random effects model | | 14219 | | 14152 | 0.602 | [0.459; 0.789] | 11.8% |
| Heterogeneity: $I^2 = 26\%$, $\tau^2 = 0.0832$, $p = 0.17$ | | | | | | | |
| t2 = LMWH | | | | | | | |
| Turpie-2009 | 20 | 1526 | 34 | 1508 | 0.593 | [0.347; 1.014] | 1.3% |
| Eriksson-2010 | 32 | 117 | 24 | 127 | 1.432 | [0.908; 2.258] | 1.5% |
| Eriksson-2010 | 38 | 120 | 24 | 127 | 1.650 | [1.067; 2.553] | 1.5% |
| Eriksson-2010 | 22 | 114 | 24 | 127 | 1.024 | [0.617; 1.701] | 1.4% |
| Eriksson-2010 | 16 | 120 | 24 | 127 | 0.719 | [0.409; 1.264] | 1.3% |
| Eriksson-2010 | 15 | 110 | 24 | 127 | 0.738 | [0.416; 1.310] | 1.3% |
| Fisher-2007 | 36 | 167 | 49 | 176 | 0.780 | [0.539; 1.127] | 1.7% |
| Fisher-2007 | 38 | 166 | 49 | 176 | 0.827 | [0.576; 1.187] | 1.7% |
| Fisher-2007 | 26 | 161 | 49 | 176 | 0.590 | [0.389; 0.895] | 1.6% |
| Fisher-2007 | 38 | 156 | 49 | 176 | 0.879 | [0.614; 1.260] | 1.7% |
| Fisher-2007 | 17 | 88 | 49 | 176 | 0.716 | [0.445; 1.151] | 1.5% |
| Eriksson-2007 | 13 | 63 | 18 | 107 | 1.243 | [0.671; 2.305] | 1.2% |
| Eriksson-2007 | 15 | 63 | 18 | 107 | 1.421 | [0.789; 2.560] | 1.3% |
| Eriksson-2007 | 11 | 55 | 18 | 107 | 1.218 | [0.638; 2.325] | 1.2% |
| Eriksson-2007 | 9 | 73 | 18 | 107 | 0.768 | [0.379; 1.557] | 1.1% |
| Eriksson-2007 | 6 | 59 | 18 | 107 | 0.663 | [0.296; 1.486] | 0.9% |
| Eriksson-2007 | 8 | 46 | 18 | 107 | 1.088 | [0.532; 2.226] | 1.0% |
| Agnelli-2007 | 11 | 26 | 14 | 66 | 1.985 | [1.075; 3.667] | 1.2% |
| Agnelli-2007 | 8 | 20 | 14 | 66 | 1.914 | [0.984; 3.724] | 1.1% |
| Agnelli-2007 | 12 | 22 | 14 | 66 | 2.525 | [1.425; 4.473] | 1.3% |
| Agnelli-2007 | 13 | 69 | 14 | 66 | 0.893 | [0.468; 1.706] | 1.2% |
| Agnelli-2007 | 15 | 80 | 14 | 66 | 0.882 | [0.472; 1.649] | 1.2% |
| Agnelli-2007 | 12 | 77 | 14 | 66 | 0.744 | [0.382; 1.451] | 1.1% |
| Fuji-2014 | 3 | 78 | 3 | 74 | 0.949 | [0.246; 3.660] | 0.5% |
| Fuji-2014 | 2 | 72 | 3 | 74 | 0.771 | [0.179; 3.324] | 0.4% |
| Eriksson-2006 | 16 | 104 | 18 | 106 | 0.912 | [0.502; 1.655] | 1.2% |
| Eriksson-2006 | 15 | 109 | 18 | 106 | 0.819 | [0.445; 1.507] | 1.2% |
| Eriksson-2006 | 12 | 101 | 18 | 106 | 0.718 | [0.374; 1.377] | 1.1% |
| Eriksson-2006 | 18 | 99 | 18 | 106 | 1.070 | [0.602; 1.901] | 1.3% |
| Eriksson-2006 | 2 | 29 | 18 | 106 | 0.563 | [0.179; 1.776] | 0.6% |
| Samama-2020 | 4 | 1661 | 18 | 1640 | 0.260 | [0.097; 0.694] | 0.7% |
| Lassen-2010 | 22 | 1194 | 68 | 1911 | 0.533 | [0.335; 0.850] | 1.5% |
| Zhao-2023 | 25 | 200 | 35 | 203 | 0.733 | [0.460; 1.167] | 1.5% |
| Raskob-2010 | 21 | 1195 | 51 | 1199 | 0.424 | [0.260; 0.694] | 1.4% |
| Raskob-2010 | 16 | 2199 | 41 | 2195 | 0.404 | [0.231; 0.708] | 1.3% |
| Turpie-2005 | 20 | 63 | 31 | 70 | 0.728 | [0.471; 1.124] | 1.5% |
| Turpie-2005 | 21 | 57 | 31 | 70 | 0.842 | [0.554; 1.278] | 1.6% |
| Turpie-2005 | 14 | 60 | 31 | 70 | 0.546 | [0.328; 0.908] | 1.4% |
| Turpie-2005 | 20 | 57 | 31 | 70 | 0.803 | [0.524; 1.232] | 1.6% |
| Turpie-2005 | 15 | 59 | 31 | 70 | 0.592 | [0.362; 0.968] | 1.4% |
| Eriksson-2008 | 12 | 1595 | 53 | 1558 | 0.235 | [0.129; 0.429] | 1.2% |
| Goldhaber-2011 | 58 | 2211 | 67 | 2284 | 0.896 | [0.636; 1.264] | 1.7% |
| Lassen-2010 | 142 | 971 | 243 | 997 | 0.602 | [0.499; 0.725] | 2.0% |
| Eriksson-2007 | 14 | 27 | 12 | 31 | 1.319 | [0.767; 2.269] | 1.3% |
| Eriksson-2007 | 12 | 31 | 12 | 31 | 1.000 | [0.553; 1.808] | 1.2% |
| Eriksson-2007 | 7 | 31 | 12 | 31 | 0.615 | [0.296; 1.279] | 1.0% |
| Eriksson-2007 | 5 | 27 | 12 | 31 | 0.527 | [0.231; 1.202] | 0.9% |
| Lassen-2008 | 67 | 965 | 97 | 959 | 0.690 | [0.513; 0.927] | 1.8% |
| Piazza-2023 | 0 | 641 | 3 | 643 | 0.251 | [0.028; 2.238] | 0.2% |
| Long-2014 | 30 | 608 | 62 | 717 | 0.580 | [0.383; 0.880] | 1.6% |
| Fuji-2014 | 3 | 46 | 1 | 27 | 1.191 | [0.233; 6.091] | 0.3% |
| Eriksson-2009 | 10 | 4657 | 20 | 4692 | 0.528 | [0.255; 1.093] | 1.0% |
| Turpie-2001 | 14 | 119 | 16 | 171 | 1.265 | [0.658; 2.432] | 1.1% |
| Turpie-2001 | 8 | 120 | 16 | 171 | 0.753 | [0.347; 1.631] | 1.0% |
| Turpie-2001 | 2 | 115 | 16 | 171 | 0.262 | [0.078; 0.873] | 0.5% |
| Turpie-2001 | 2 | 45 | 16 | 171 | 0.660 | [0.202; 2.155] | 0.6% |
| Turpie-2001 | 0 | 23 | 16 | 171 | 0.422 | [0.059; 3.026] | 0.2% |
| Turpie-2009 | 14 | 70 | 4 | 40 | 1.732 | [0.679; 4.418] | 0.8% |
| Turpie-2009 | 10 | 65 | 4 | 40 | 1.367 | [0.512; 3.651] | 0.7% |
| Eriksson-2006 | 14 | 94 | 27 | 107 | 0.609 | [0.347; 1.070] | 1.3% |
| Eriksson-2006 | 12 | 113 | 27 | 107 | 0.440 | [0.241; 0.804] | 1.2% |
| Eriksson-2006 | 9 | 106 | 27 | 107 | 0.360 | [0.184; 0.705] | 1.1% |
| Eriksson-2006 | 14 | 104 | 27 | 107 | 0.551 | [0.313; 0.971] | 1.3% |
| Eriksson-2006 | 6 | 94 | 27 | 107 | 0.284 | [0.130; 0.621] | 1.0% |
| Oliveira-2022 | 5 | 114 | 4 | 114 | 1.200 | [0.377; 3.822] | 0.6% |
| Veen-2021 | 0 | 48 | 0 | 45 | | | 0.0% |
| Verhamme-2013 | 9 | 193 | 10 | 195 | 0.918 | [0.399; 2.113] | 0.9% |
| Verhamme-2013 | 9 | 193 | 10 | 196 | 0.923 | [0.401; 2.124] | 0.9% |
| Weitz-2020 | 11 | 83 | 20 | 76 | 0.524 | [0.277; 0.992] | 1.2% |
| Random effects model | | 24344 | | 26334 | 0.770 | [0.690; 0.860] | 79.1% |
| Heterogeneity: $I^2 = 55\%$, $\tau^2 = 0.1090$, $p < 0.01$ | | | | | | | |
| t2 = VKA | | | | | | | |
| Lassen-2007 | 10 | 111 | 29 | 109 | 0.360 | [0.190; 0.682] | 1.2% |
| Lassen-2007 | 11 | 97 | 29 | 109 | 0.449 | [0.244; 0.828] | 1.2% |
| Lassen-2007 | 5 | 105 | 29 | 109 | 0.208 | [0.090; 0.478] | 0.9% |
| Lassen-2007 | 13 | 105 | 29 | 109 | 0.484 | [0.272; 0.861] | 1.3% |
| Lassen-2007 | 6 | 110 | 29 | 109 | 0.231 | [0.106; 0.504] | 1.0% |
| Lassen-2007 | 9 | 110 | 29 | 109 | 0.330 | [0.170; 0.643] | 1.1% |
| Lassen-2007 | 17 | 109 | 29 | 109 | 0.600 | [0.356; 1.010] | 1.4% |
| Random effects model | | 747 | | 763 | 0.389 | [0.297; 0.510] | 8.0% |
| Heterogeneity: $I^2 = 21\%$, $\tau^2 = 0.0251$, $p = 0.27$ | | | | | | | |
| t2 = AP | | | | | | | |
| Anderson-2018 | 12 | 1717 | 11 | 1707 | 1.077 | [0.493; 2.354] | 1.0% |
| t2 = TI | | | | | | | |
| Veen-2021 | 0 | 48 | 1 | 45 | 0.469 | [0.044; 5.004] | 0.2% |
| **Random effects model** | | **41075** | | **43001** | **0.706** | **[0.637; 0.782]** | **100.0%** |

Heterogeneity: $I^2 = 55\%$, $\tau^2 = 0.1279$, $p < 0.01$
Test for subgroup differences: $\chi^2_4 = 23.42$, df = 4 ($p < 0.01$)

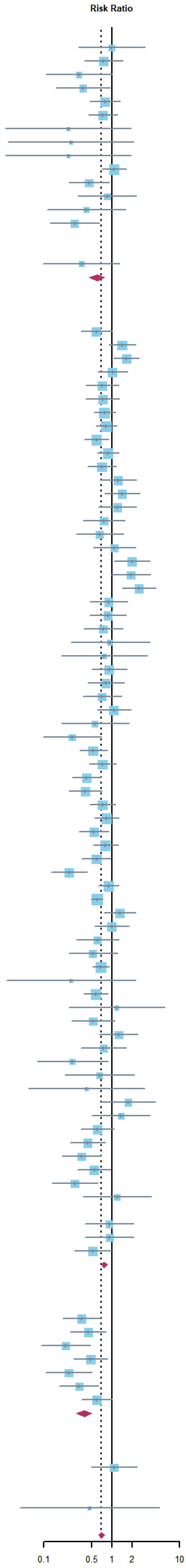

**Outcome: Major bleeding during prophylaxis**

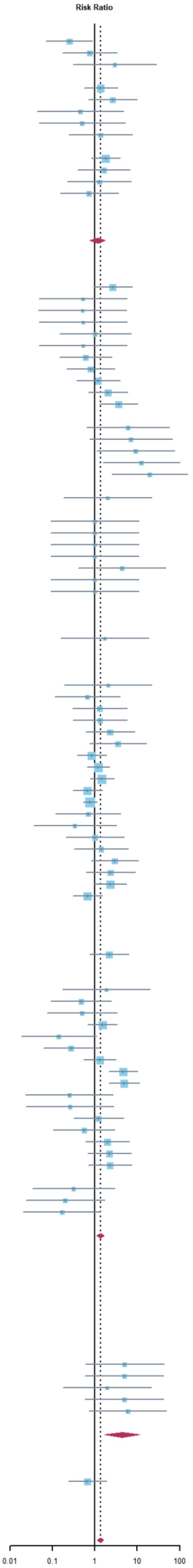


| Study | Experimental Events | Experimental Total | Control Events | Control Total | RR | 95%-CI | Weight |
|---|---|---|---|---|---|---|---|
| t2 = Placebo | | | | | | | |
| Wang-2023 | 2 | 610 | 11 | 607 | 0.249 | [0.071; 0.877] | 1.5% |
| Vadhan-2020 | 2 | 130 | 3 | 131 | 0.756 | [0.172; 3.311] | 1.2% |
| Becattini-2022 | 2 | 287 | 0 | 282 | 2.948 | [0.308; 28.172] | 0.6% |
| Ramacciotti-2022 | 0 | 159 | 0 | 159 | | | 0.0% |
| Ageno-2021 | 10 | 3837 | 7 | 3855 | 1.381 | [0.556; 3.431] | 2.2% |
| Ageno-2021 | 7 | 2145 | 2 | 2125 | 2.642 | [0.702; 9.944] | 1.4% |
| Levine-2012 | 0 | 32 | 1 | 29 | 0.455 | [0.043; 4.761] | 0.6% |
| Levine-2012 | 0 | 29 | 1 | 29 | 0.500 | [0.048; 5.224] | 0.6% |
| Levine-2012 | 2 | 32 | 1 | 29 | 1.364 | [0.244; 7.612] | 0.9% |
| Eriksson-2008 | 0 | 129 | 0 | 132 | | | 0.0% |
| Spyropoulos-2018 | 17 | 5982 | 9 | 5980 | 1.799 | [0.831; 3.895] | 2.6% |
| Zhang-2021 | 4 | 237 | 2 | 232 | 1.632 | [0.394; 6.750] | 1.3% |
| Zhang-2021 | 2 | 51 | 1 | 43 | 1.269 | [0.222; 7.257] | 0.9% |
| Brandt-2022 | 2 | 126 | 2 | 91 | 0.724 | [0.150; 3.509] | 1.1% |
| Connors-2021 | 0 | 135 | 0 | 136 | | | 0.0% |
| Connors-2021 | 0 | 143 | 0 | 136 | | | 0.0% |
| Camporese-2016 | 0 | 120 | 0 | 114 | | | 0.0% |
| Random effects model | | 14184 | | 14110 | 1.153 | [0.746; 1.783] | 14.8% |
| Heterogeneity: $I^2 = 2\%$, $\tau^2 = 0.0853$, $p = 0.42$ | | | | | | | |
| t2 = LMWH | | | | | | | |
| Turpie-2009 | 10 | 1256 | 4 | 1508 | 2.641 | [0.920; 7.581] | 1.9% |
| Eriksson-2010 | 0 | 158 | 1 | 166 | 0.525 | [0.048; 5.735] | 0.5% |
| Eriksson-2010 | 0 | 161 | 1 | 166 | 0.515 | [0.047; 5.629] | 0.5% |
| Eriksson-2010 | 0 | 156 | 1 | 166 | 0.532 | [0.049; 5.807] | 0.5% |
| Eriksson-2010 | 1 | 163 | 1 | 166 | 1.018 | [0.145; 7.144] | 0.8% |
| Eriksson-2010 | 0 | 156 | 1 | 166 | 0.532 | [0.049; 5.807] | 0.5% |
| Fisher-2007 | 2 | 232 | 4 | 236 | 0.610 | [0.148; 2.525] | 1.3% |
| Fisher-2007 | 3 | 238 | 4 | 236 | 0.793 | [0.216; 2.918] | 1.4% |
| Fisher-2007 | 5 | 236 | 4 | 236 | 1.200 | [0.371; 3.878] | 1.6% |
| Fisher-2007 | 9 | 232 | 4 | 236 | 2.034 | [0.706; 5.861] | 1.9% |
| Fisher-2007 | 10 | 143 | 4 | 236 | 3.621 | [1.284; 10.210] | 1.9% |
| Eriksson-2007 | 0 | 76 | 0 | 162 | | | 0.0% |
| Eriksson-2007 | 2 | 80 | 0 | 162 | 6.037 | [0.638; 57.131] | 0.6% |
| Eriksson-2007 | 2 | 68 | 0 | 162 | 7.087 | [0.750; 66.944] | 0.6% |
| Eriksson-2007 | 4 | 88 | 0 | 162 | 9.157 | [1.087; 77.168] | 0.7% |
| Eriksson-2007 | 5 | 77 | 0 | 162 | 12.538 | [1.536; 102.364] | 0.7% |
| Eriksson-2007 | 8 | 74 | 0 | 162 | 19.560 | [2.524; 151.607] | 0.7% |
| Verhamme-2021 | 0 | 102 | 0 | 104 | | | 0.0% |
| Verhamme-2021 | 1 | 104 | 0 | 104 | 2.000 | [0.184; 21.722] | 0.5% |
| Verhamme-2021 | 0 | 99 | 0 | 104 | | | 0.0% |
| Weitz-2021 | 0 | 148 | 1 | 296 | 0.997 | [0.091; 10.903] | 0.5% |
| Weitz-2021 | 0 | 148 | 1 | 296 | 0.997 | [0.091; 10.903] | 0.5% |
| Weitz-2021 | 0 | 149 | 1 | 296 | 0.990 | [0.090; 10.830] | 0.5% |
| Weitz-2021 | 0 | 148 | 1 | 296 | 0.997 | [0.091; 10.903] | 0.5% |
| Weitz-2021 | 0 | 33 | 1 | 296 | 4.368 | [0.407; 46.914] | 0.6% |
| Weitz-2021 | 0 | 150 | 1 | 296 | 0.983 | [0.090; 10.759] | 0.5% |
| Weitz-2021 | 0 | 147 | 1 | 296 | 1.003 | [0.092; 10.976] | 0.5% |
| Agnelli-2007 | 0 | 32 | 0 | 90 | | | 0.0% |
| Agnelli-2007 | 0 | 34 | 0 | 90 | | | 0.0% |
| Agnelli-2007 | 0 | 32 | 0 | 90 | | | 0.0% |
| Agnelli-2007 | 1 | 106 | 0 | 90 | 1.701 | [0.157; 18.454] | 0.5% |
| Agnelli-2007 | 0 | 110 | 0 | 90 | | | 0.0% |
| Agnelli-2007 | 0 | 103 | 0 | 90 | | | 0.0% |
| Fuji-2014 | 0 | 89 | 0 | 87 | | | 0.0% |
| Fuji-2014 | 1 | 85 | 0 | 87 | 2.047 | [0.189; 22.156] | 0.5% |
| Eriksson-2006 | 1 | 132 | 2 | 132 | 0.667 | [0.113; 3.926] | 0.9% |
| Eriksson-2006 | 3 | 136 | 2 | 132 | 1.294 | [0.295; 5.674] | 1.2% |
| Eriksson-2006 | 3 | 133 | 2 | 132 | 1.323 | [0.302; 5.800] | 1.2% |
| Eriksson-2006 | 6 | 134 | 2 | 132 | 2.299 | [0.607; 8.702] | 1.4% |
| Eriksson-2006 | 2 | 37 | 2 | 132 | 3.500 | [0.736; 16.642] | 1.1% |
| Samama-2020 | 10 | 1757 | 12 | 1739 | 0.837 | [0.376; 1.864] | 2.5% |
| Lassen-2010 | 22 | 2673 | 18 | 2659 | 1.204 | [0.657; 2.206] | 3.1% |
| Zhao-2023 | 19 | 196 | 13 | 201 | 1.465 | [0.762; 2.818] | 3.0% |
| Raskob-2010 | 9 | 1501 | 14 | 1508 | 0.670 | [0.302; 1.486] | 2.5% |
| Raskob-2010 | 44 | 1501 | 58 | 1508 | 0.766 | [0.523; 1.122] | 3.9% |
| Turpie-2005 | 1 | 100 | 2 | 104 | 0.693 | [0.118; 4.062] | 0.9% |
| Turpie-2005 | 0 | 102 | 2 | 104 | 0.340 | [0.036; 3.214] | 0.6% |
| Turpie-2005 | 2 | 103 | 2 | 104 | 1.010 | [0.209; 4.888] | 1.1% |
| Turpie-2005 | 3 | 98 | 2 | 104 | 1.414 | [0.325; 6.160] | 1.2% |
| Turpie-2005 | 8 | 106 | 2 | 104 | 2.944 | [0.820; 10.573] | 1.5% |
| Eriksson-2008 | 6 | 2209 | 2 | 2224 | 2.349 | [0.608; 9.073] | 1.4% |
| Goldhaber-2011 | 15 | 3184 | 6 | 3217 | 2.309 | [0.951; 5.606] | 2.3% |
| Lassen-2010 | 9 | 1501 | 14 | 1508 | 0.670 | [0.302; 1.486] | 2.5% |
| Eriksson-2007 | 0 | 34 | 0 | 36 | | | 0.0% |
| Eriksson-2007 | 0 | 35 | 0 | 36 | | | 0.0% |
| Eriksson-2007 | 0 | 36 | 0 | 36 | | | 0.0% |
| Eriksson-2007 | 0 | 34 | 0 | 36 | | | 0.0% |
| Lassen-2008 | 10 | 1526 | 4 | 1508 | 2.174 | [0.757; 6.242] | 1.9% |
| Piazza-2023 | 0 | 641 | 0 | 643 | | | 0.0% |
| Bueller-2015 | 0 | 144 | 0 | 72 | | | 0.0% |
| Bueller-2015 | 1 | 77 | 0 | 72 | 1.872 | [0.173; 20.207] | 0.5% |
| Long-2014 | 1 | 608 | 4 | 717 | 0.472 | [0.092; 2.422] | 1.0% |
| Fuji-2014 | 1 | 59 | 1 | 29 | 0.500 | [0.074; 3.378] | 0.8% |
| Eriksson-2009 | 14 | 4657 | 9 | 4692 | 1.511 | [0.680; 3.360] | 2.5% |
| Turpie-2001 | 0 | 184 | 9 | 260 | 0.141 | [0.018; 1.093] | 0.7% |
| Turpie-2001 | 1 | 188 | 9 | 260 | 0.276 | [0.061; 1.246] | 1.2% |
| Turpie-2001 | 8 | 177 | 9 | 260 | 1.320 | [0.547; 3.182] | 2.3% |
| Turpie-2001 | 12 | 72 | 9 | 260 | 4.648 | [2.125; 10.165] | 2.6% |
| Turpie-2001 | 9 | 52 | 9 | 260 | 4.925 | [2.157; 11.240] | 2.4% |
| Turpie-2009 | 0 | 87 | 1 | 43 | 0.250 | [0.023; 2.682] | 0.6% |
| Turpie-2009 | 0 | 84 | 1 | 43 | 0.259 | [0.024; 2.776] | 0.6% |
| Eriksson-2006 | 3 | 128 | 3 | 157 | 1.225 | [0.312; 4.802] | 1.3% |
| Eriksson-2006 | 1 | 142 | 3 | 157 | 0.552 | [0.103; 2.971] | 1.0% |
| Eriksson-2006 | 6 | 139 | 3 | 157 | 1.975 | [0.591; 6.605] | 1.6% |
| Eriksson-2006 | 7 | 142 | 3 | 157 | 2.210 | [0.680; 7.182] | 1.6% |
| Eriksson-2006 | 7 | 137 | 3 | 157 | 2.290 | [0.705; 7.439] | 1.6% |
| Oliveira-2022 | 0 | 114 | 0 | 114 | | | 0.0% |
| Veen-2021 | 0 | 48 | 2 | 45 | 0.313 | [0.034; 2.902] | 0.6% |
| Verhamme-2013 | 0 | 207 | 4 | 207 | 0.200 | [0.024; 1.697] | 0.7% |
| Verhamme-2013 | 0 | 207 | 5 | 208 | 0.167 | [0.020; 1.379] | 0.7% |
| Weitz-2020 | 0 | 83 | 0 | 76 | | | 0.0% |
| Random effects model | | 31058 | | 34030 | 1.350 | [1.090; 1.673] | 80.0% |
| Heterogeneity: $I^2 = 37\%$, $\tau^2 = 0.2222$, $p < 0.01$ | | | | | | | |
| t2 = NOACs | | | | | | | |
| Weitz-2019 | 0 | 38 | 0 | 63 | | | 0.0% |
| Weitz-2019 | 0 | 40 | 0 | 63 | | | 0.0% |
| Weitz-2019 | 0 | 42 | 0 | 63 | | | 0.0% |
| Weitz-2019 | 0 | 122 | 0 | 63 | | | 0.0% |
| t2 = VKA | | | | | | | |
| Lassen-2007 | 0 | 154 | 0 | 151 | | | 0.0% |
| Lassen-2007 | 4 | 151 | 0 | 151 | 5.000 | [0.591; 42.295] | 0.7% |
| Lassen-2007 | 4 | 153 | 0 | 151 | 4.935 | [0.583; 41.749] | 0.7% |
| Lassen-2007 | 1 | 155 | 0 | 151 | 1.949 | [0.179; 21.268] | 0.5% |
| Lassen-2007 | 4 | 153 | 0 | 151 | 4.935 | [0.583; 41.749] | 0.7% |
| Lassen-2007 | 5 | 151 | 0 | 151 | 6.000 | [0.731; 49.244] | 0.7% |
| Lassen-2007 | 0 | 149 | 0 | 151 | | | 0.0% |
| Random effects model | | 1066 | | 1057 | 4.424 | [1.674; 11.693] | 3.2% |
| Heterogeneity: $I^2 = 0\%$, $\tau^2 = 0$, $p = 0.97$ | | | | | | | |
| t2 = AP | | | | | | | |
| Anderson-2018 | 5 | 1717 | 8 | 1707 | 0.663 | [0.236; 1.858] | 1.9% |
| t2 = TI | | | | | | | |
| Veen-2021 | 0 | 48 | 0 | 45 | | | 0.0% |
| **Random effects model** | | **48315** | | **51201** | **1.348** | **[1.118; 1.627]** | **100.0%** |

Heterogeneity: $I^2 = 32\%$, $\tau^2 = 0.1981$, $p < 0.01$

Test for subgroup differences: $\chi^2_3 = 8.05$, df = 3 ($p = 0.04$)

**Outcome: All-cause mortality during prophylaxis**

| Study | Experimental Events | Experimental Total | Control Events | Control Total | RR | 95%-CI | Weight |
|---|---|---|---|---|---|---|---|
| t2 = Placebo | | | | | | | |
| Wang-1 | 8 | 610 | 9 | 607 | 0.88 | [0.34; 2.28] | 4.7% |
| Becattini-24 | 0 | 287 | 1 | 282 | 0.33 | [0.01; 8.01] | 0.8% |
| Carrier-25 | 35 | 288 | 27 | 275 | 1.24 | [0.77; 1.99] | 14.4% |
| Eriksson-43 | 2 | 129 | 0 | 131 | 5.08 | [0.25; 104.73] | 0.3% |
| Spyropoulos-45 | 71 | 6007 | 89 | 6012 | 0.80 | [0.59; 1.09] | 46.4% |
| Zhang-59 | 1 | 237 | 4 | 232 | 0.24 | [0.03; 2.17] | 2.1% |
| Zhang-60 | 0 | 51 | 1 | 43 | 0.28 | [0.01; 6.74] | 0.8% |
| Piazza-69 | 2 | 641 | 2 | 643 | 1.00 | [0.14; 7.10] | 1.0% |
| Brandt-73 | 1 | 126 | 2 | 91 | 0.36 | [0.03; 3.92] | 1.2% |
| Connors-82 | 0 | 135 | 0 | 136 | | | 0.0% |
| Connors-83 | 0 | 143 | 0 | 136 | | | 0.0% |
| Common effect model | | 8654 | | 8588 | 0.88 | [0.69; 1.11] | 71.7% |
| Heterogeneity: $I^2 = 0\%$, $\tau^2 = 0.0180$, $p = 0.60$ | | | | | | | |
| t2 = LMWH | | | | | | | |
| Eriksson-2 | 1 | 110 | 0 | 127 | 3.46 | [0.14; 84.11] | 0.2% |
| Fisher-3 | 0 | 167 | 0 | 176 | | | 0.0% |
| Fisher-4 | 0 | 166 | 0 | 176 | | | 0.0% |
| Fisher-5 | 0 | 161 | 0 | 176 | | | 0.0% |
| Fisher-6 | 0 | 156 | 0 | 176 | | | 0.0% |
| Fisher-7 | 0 | 88 | 0 | 176 | | | 0.0% |
| Eriksson-8 | 1 | 63 | 0 | 107 | 5.08 | [0.21; 122.81] | 0.2% |
| Eriksson-9 | 0 | 63 | 0 | 107 | | | 0.0% |
| Eriksson-10 | 0 | 55 | 0 | 107 | | | 0.0% |
| Eriksson-11 | 1 | 73 | 0 | 107 | 4.39 | [0.18; 106.23] | 0.2% |
| Eriksson-12 | 0 | 59 | 0 | 107 | | | 0.0% |
| Eriksson-13 | 0 | 46 | 0 | 107 | | | 0.0% |
| Eriksson-15 | 1 | 675 | 1 | 685 | 1.01 | [0.06; 16.19] | 0.5% |
| Eriksson-16 | 1 | 696 | 1 | 685 | 0.98 | [0.06; 15.70] | 0.5% |
| Weitz-17 | 0 | 129 | 1 | 252 | 0.65 | [0.03; 15.84] | 0.5% |
| Weitz-18 | 0 | 124 | 1 | 252 | 0.68 | [0.03; 16.48] | 0.5% |
| Weitz-19 | 0 | 134 | 1 | 252 | 0.63 | [0.03; 15.26] | 0.5% |
| Weitz-20 | 0 | 131 | 1 | 252 | 0.64 | [0.03; 15.60] | 0.5% |
| Weitz-21 | 0 | 28 | 1 | 252 | 2.95 | [0.12; 70.81] | 0.2% |
| Weitz-22 | 0 | 127 | 1 | 252 | 0.66 | [0.03; 16.09] | 0.5% |
| Weitz-23 | 0 | 123 | 1 | 252 | 0.68 | [0.03; 16.61] | 0.5% |
| Fuji-27 | 0 | 78 | 0 | 74 | | | 0.0% |
| Fuji-28 | 0 | 72 | 0 | 74 | | | 0.0% |
| Samama-34 | 0 | 1756 | 1 | 1737 | 0.33 | [0.01; 8.09] | 0.8% |
| Lassen-37 | 3 | 2708 | 1 | 2699 | 2.99 | [0.31; 28.73] | 0.5% |
| Eriksson-58 | 5 | 1595 | 4 | 1558 | 1.22 | [0.33; 4.54] | 2.1% |
| Lassen-65 | 2 | 1528 | 0 | 1529 | 5.00 | [0.24; 104.13] | 0.3% |
| Lassen-68 | 11 | 1526 | 18 | 1508 | 0.60 | [0.29; 1.27] | 9.4% |
| Fuji-70 | 0 | 46 | 0 | 27 | | | 0.0% |
| Eriksson-71 | 7 | 4657 | 19 | 4692 | 0.37 | [0.16; 0.88] | 9.9% |
| Eriksson-76 | 0 | 94 | 0 | 107 | | | 0.0% |
| Eriksson-77 | 0 | 113 | 0 | 107 | | | 0.0% |
| Eriksson-78 | 0 | 106 | 0 | 107 | | | 0.0% |
| Eriksson-79 | 0 | 104 | 0 | 107 | | | 0.0% |
| Eriksson-80 | 0 | 94 | 0 | 107 | | | 0.0% |
| Common effect model | | 17851 | | 19216 | 0.76 | [0.51; 1.14] | 28.0% |
| Heterogeneity: $I^2 = 0\%$, $\tau^2 = 0.0687$, $p = 0.86$ | | | | | | | |
| t2 = VKA | | | | | | | |
| Lassen-49 | 1 | 111 | 0 | 109 | 2.95 | [0.12; 71.54] | 0.3% |
| Lassen-50 | 0 | 97 | 0 | 109 | | | 0.0% |
| Lassen-51 | 0 | 105 | 0 | 109 | | | 0.0% |
| Lassen-52 | 0 | 105 | 0 | 109 | | | 0.0% |
| Lassen-53 | 0 | 110 | 0 | 109 | | | 0.0% |
| Lassen-54 | 0 | 110 | 0 | 109 | | | 0.0% |
| Lassen-55 | 0 | 109 | 0 | 109 | | | 0.0% |
| **Common effect model** | | **27252** | | **28567** | **0.85** | **[0.69; 1.04]** | **100.0%** |

Heterogeneity: $I^2 = 0\%$, $\tau^2 = 0.0197$, $p = 0.90$

Test for subgroup differences: $\chi^2_2 = 0.91$, df = 2 ($p = 0.63$)

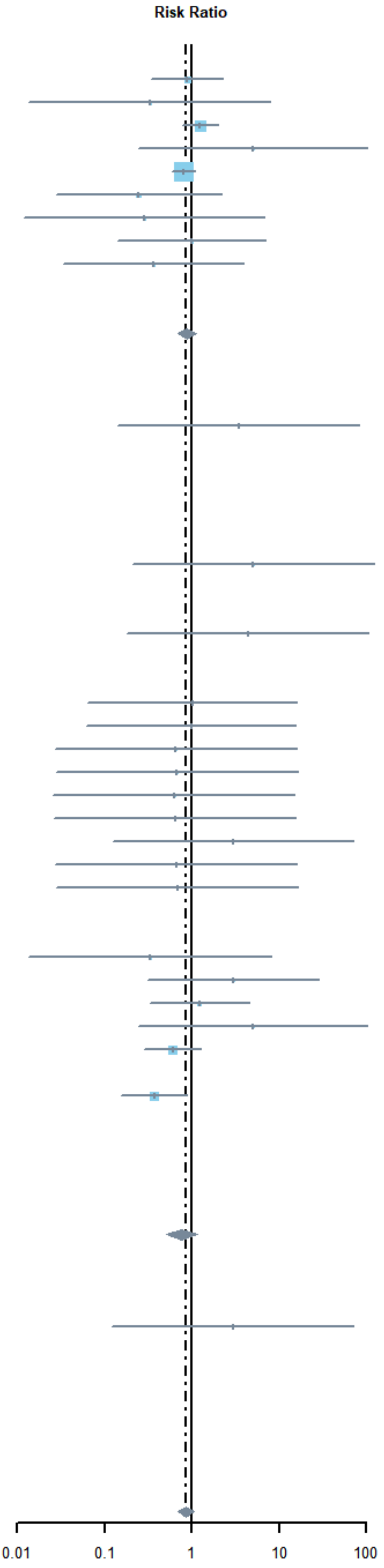

**Outcome: Clinical relevant non-major bleeding during prophylaxis**

| Study | Experimental Events | Experimental Total | Control Events | Control Total | RR | 95%-CI | Weight |
|---|---|---|---|---|---|---|---|
| t2 = Placebo | | | | | | | |
| Wang-2023 | 3 | 610 | 6 | 607 | 0.569 | [0.167; 1.932] | 0.9% |
| Vadhan-2020 | 5 | 130 | 2 | 131 | 2.015 | [0.515; 7.889] | 0.7% |
| Becattini-2022 | 3 | 287 | 5 | 282 | 0.655 | [0.187; 2.297] | 0.8% |
| Ramacciotti-2022 | 2 | 159 | 2 | 159 | 1.000 | [0.205; 4.880] | 0.5% |
| Levine-2012 | 1 | 32 | 0 | 29 | 1.818 | [0.174; 19.045] | 0.3% |
| Levine-2012 | 1 | 29 | 0 | 29 | 2.000 | [0.191; 20.898] | 0.3% |
| Levine-2012 | 2 | 32 | 0 | 29 | 2.727 | [0.300; 24.824] | 0.3% |
| Eriksson-2008 | 2 | 129 | 1 | 132 | 1.535 | [0.261; 9.035] | 0.4% |
| Spyropoulos-2018 | 85 | 5982 | 51 | 5980 | 1.653 | [1.174; 2.328] | 6.0% |
| Zhang-2021 | 15 | 237 | 14 | 232 | 1.044 | [0.529; 2.063] | 2.5% |
| Connors-2021 | 1 | 135 | 0 | 136 | 2.015 | [0.185; 21.959] | 0.2% |
| Connors-2021 | 1 | 143 | 0 | 136 | 1.903 | [0.175; 20.746] | 0.2% |
| Random effects model | | 7905 | | 7882 | 1.371 | [1.011; 1.858] | 13.2% |
| Heterogeneity: $I^2 = 0\%$, $\tau^2 = 0.0165$, $p = 0.86$ | | | | | | | |
| t2 = LMWH | | | | | | | |
| Turpie-2009 | 39 | 1256 | 30 | 1508 | 1.549 | [0.975; 2.461] | 4.3% |
| Eriksson-2010 | 4 | 158 | 4 | 166 | 1.050 | [0.310; 3.559] | 0.9% |
| Eriksson-2010 | 4 | 161 | 4 | 166 | 1.031 | [0.304; 3.494] | 0.9% |
| Eriksson-2010 | 5 | 156 | 4 | 166 | 1.276 | [0.397; 4.099] | 1.0% |
| Eriksson-2010 | 9 | 163 | 4 | 166 | 2.037 | [0.711; 5.830] | 1.2% |
| Eriksson-2010 | 8 | 156 | 4 | 166 | 1.915 | [0.656; 5.589] | 1.1% |
| Fisher-2007 | 4 | 232 | 3 | 236 | 1.271 | [0.346; 4.676] | 0.8% |
| Fisher-2007 | 11 | 238 | 3 | 236 | 2.975 | [0.973; 9.092] | 1.0% |
| Fisher-2007 | 4 | 236 | 3 | 236 | 1.250 | [0.340; 4.598] | 0.8% |
| Fisher-2007 | 11 | 232 | 3 | 236 | 3.052 | [0.999; 9.325] | 1.0% |
| Fisher-2007 | 8 | 143 | 3 | 236 | 3.703 | [1.162; 11.806] | 1.0% |
| Eriksson-2007 | 2 | 76 | 3 | 162 | 1.588 | [0.364; 6.920] | 0.6% |
| Eriksson-2007 | 1 | 80 | 3 | 162 | 1.006 | [0.188; 5.379] | 0.5% |
| Eriksson-2007 | 3 | 68 | 3 | 162 | 2.362 | [0.608; 9.177] | 0.7% |
| Eriksson-2007 | 6 | 88 | 3 | 162 | 3.205 | [0.964; 10.652] | 0.9% |
| Eriksson-2007 | 4 | 77 | 3 | 162 | 2.612 | [0.721; 9.459] | 0.8% |
| Eriksson-2007 | 7 | 74 | 3 | 162 | 4.347 | [1.351; 13.986] | 1.0% |
| Verhamme-2021 | 2 | 102 | 0 | 104 | 3.058 | [0.323; 28.923] | 0.3% |
| Verhamme-2021 | 2 | 104 | 0 | 104 | 3.000 | [0.317; 28.376] | 0.3% |
| Verhamme-2021 | 0 | 99 | 0 | 104 | | | 0.0% |
| Eriksson-2007 | 40 | 679 | 37 | 694 | 1.103 | [0.718; 1.693] | 4.7% |
| Eriksson-2007 | 48 | 703 | 37 | 694 | 1.273 | [0.844; 1.919] | 5.0% |
| Fuji-2014 | 2 | 89 | 2 | 87 | 0.978 | [0.203; 4.714] | 0.6% |
| Fuji-2014 | 1 | 85 | 2 | 87 | 0.682 | [0.117; 3.982] | 0.4% |
| Eriksson-2006 | 2 | 132 | 0 | 132 | 3.000 | [0.316; 28.474] | 0.3% |
| Eriksson-2006 | 8 | 136 | 0 | 132 | 8.737 | [1.122; 68.017] | 0.3% |
| Eriksson-2006 | 3 | 133 | 0 | 132 | 3.970 | [0.450; 35.056] | 0.3% |
| Eriksson-2006 | 6 | 134 | 0 | 132 | 6.896 | [0.860; 55.287] | 0.3% |
| Eriksson-2006 | 1 | 37 | 0 | 132 | 7.000 | [0.652; 75.124] | 0.2% |
| Samama-2020 | 9 | 1757 | 6 | 1739 | 1.414 | [0.539; 3.706] | 1.4% |
| Lassen-2010 | 109 | 2673 | 120 | 2659 | 0.904 | [0.702; 1.164] | 7.8% |
| Zhao-2023 | 6 | 196 | 1 | 201 | 3.589 | [0.755; 17.064] | 0.6% |
| Turpie-2005 | 2 | 100 | 3 | 104 | 0.780 | [0.179; 3.397] | 0.6% |
| Turpie-2005 | 3 | 102 | 3 | 104 | 1.019 | [0.262; 3.968] | 0.7% |
| Turpie-2005 | 1 | 103 | 3 | 104 | 0.505 | [0.094; 2.697] | 0.5% |
| Turpie-2005 | 5 | 98 | 3 | 104 | 1.591 | [0.463; 5.470] | 0.9% |
| Turpie-2005 | 7 | 106 | 3 | 104 | 1.963 | [0.609; 6.321] | 1.0% |
| Eriksson-2008 | 65 | 2209 | 54 | 2224 | 1.208 | [0.849; 1.720] | 5.8% |
| Goldhaber-2011 | 70 | 3184 | 61 | 3217 | 1.157 | [0.826; 1.621] | 6.1% |
| Lassen-2010 | 44 | 1501 | 58 | 1508 | 0.766 | [0.523; 1.122] | 5.4% |
| Eriksson-2007 | 1 | 34 | 0 | 36 | 2.114 | [0.201; 22.293] | 0.3% |
| Eriksson-2007 | 2 | 35 | 0 | 36 | 3.083 | [0.336; 28.281] | 0.3% |
| Eriksson-2007 | 0 | 36 | 0 | 36 | | | 0.0% |
| Eriksson-2007 | 0 | 34 | 0 | 36 | | | 0.0% |
| Lassen-2008 | 39 | 1526 | 30 | 1508 | 1.275 | [0.802; 2.027] | 4.3% |
| Piazza-2023 | 9 | 599 | 1 | 599 | 5.000 | [1.100; 22.723] | 0.6% |
| Bueller-2015 | 4 | 144 | 6 | 72 | 0.360 | [0.118; 1.094] | 1.1% |
| Bueller-2015 | 1 | 77 | 6 | 72 | 0.267 | [0.057; 1.246] | 0.6% |
| Long-2014 | 6 | 608 | 13 | 717 | 0.589 | [0.239; 1.451] | 1.5% |
| Fuji-2014 | 1 | 59 | 1 | 29 | 0.500 | [0.074; 3.378] | 0.4% |
| Eriksson-2009 | 138 | 4657 | 115 | 4692 | 1.207 | [0.947; 1.540] | 8.0% |
| Turpie-2009 | 0 | 87 | 2 | 43 | 0.167 | [0.018; 1.556] | 0.3% |
| Turpie-2009 | 2 | 84 | 2 | 43 | 0.518 | [0.109; 2.459] | 0.6% |
| Eriksson-2006 | 2 | 128 | 5 | 157 | 0.612 | [0.156; 2.401] | 0.7% |
| Eriksson-2006 | 3 | 142 | 5 | 157 | 0.737 | [0.212; 2.558] | 0.9% |
| Eriksson-2006 | 1 | 139 | 5 | 157 | 0.376 | [0.077; 1.834] | 0.5% |
| Eriksson-2006 | 3 | 142 | 5 | 157 | 0.737 | [0.212; 2.558] | 0.9% |
| Eriksson-2006 | 4 | 137 | 5 | 157 | 0.954 | [0.298; 3.058] | 1.0% |
| Oliveira-2022 | 3 | 114 | 0 | 114 | 4.000 | [0.454; 35.244] | 0.3% |
| Verhamme-2013 | 3 | 207 | 8 | 207 | 0.444 | [0.139; 1.421] | 1.0% |
| Verhamme-2013 | 3 | 207 | 11 | 208 | 0.335 | [0.110; 1.022] | 1.1% |
| Weitz-2020 | 2 | 83 | 6 | 76 | 0.393 | [0.105; 1.466] | 0.8% |
| Random effects model | | 27335 | | 28401 | 1.180 | [1.040; 1.339] | 86.8% |
| Heterogeneity: $I^2 = 31\%$, $\tau^2 = 0.0279$, $p = 0.02$ | | | | | | | |
| **Random effects model** | | **35240** | | **36283** | **1.203** | **[1.067; 1.356]** | **100.0%** |

Heterogeneity: $I^2 = 24\%$, $\tau^2 = 0.0316$, $p = 0.04$
Test for subgroup differences: $\chi^2_1 = 0.79$, df = 1 ($p = 0.37$)

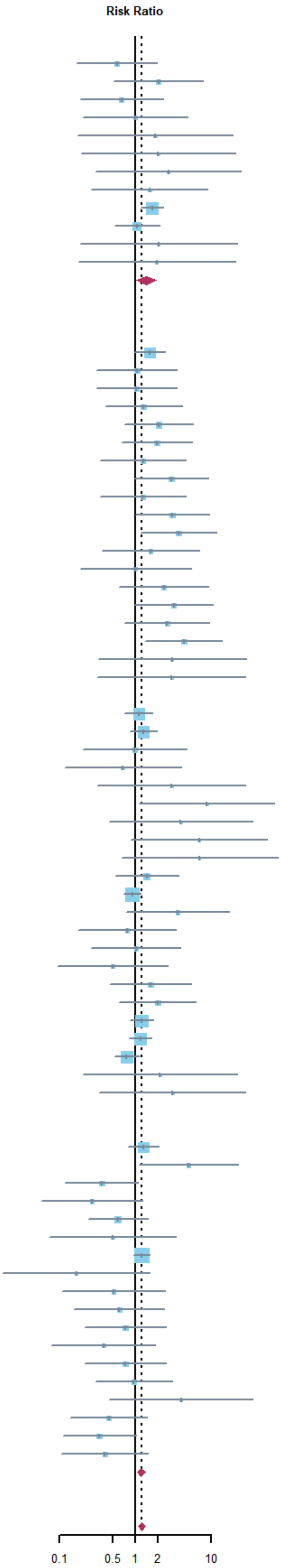

**Outcome: VTE related death during prophylaxis**

| Study | Experimental Events | Experimental Total | Control Events | Control Total | RR | 95%-CI | Weight |
|---|---|---|---|---|---|---|---|
| t2 = VKA | | | | | | | |
| Raskob-2016 | 0 | 1076 | 2 | 1084 | 0.20 | [0.01; 4.19] | 11.4% |
| Raskob-2016 | 0 | 896 | 0 | 851 | | | 0.0% |
| Raskob-2016 | 0 | 1661 | 2 | 1659 | 0.20 | [0.01; 4.16] | 11.4% |
| Medina-2017 | 0 | 601 | 1 | 582 | 0.32 | [0.01; 7.91] | 7.0% |
| Medina-2017 | 0 | 903 | 0 | 906 | | | 0.0% |
| Nakamura-2015 | 0 | 563 | 2 | 538 | 0.19 | [0.01; 3.97] | 11.7% |
| Nakamura-2015 | 2 | 3555 | 8 | 3584 | 0.25 | [0.05; 1.19] | 36.4% |
| Common effect model | | 9255 | | 9204 | 0.23 | [0.08; 0.70] | 77.9% |
| Heterogeneity: $I^2 = 0\%$, $\tau^2 = 0$, $p = 1.00$ | | | | | | | |
| t2 = LMWH | | | | | | | |
| Planquette-2022 | 0 | 74 | 2 | 84 | 0.23 | [0.01; 4.65] | 10.7% |
| t2 = Placebo | | | | | | | |
| Wells-2016 | 0 | 602 | 0 | 594 | | | 0.0% |
| t2 = AP | | | | | | | |
| Weitz-2017 | 1 | 1107 | 1 | 1131 | 1.02 | [0.06; 16.31] | 4.5% |
| Weitz-2017 | 0 | 1127 | 1 | 1131 | 0.33 | [0.01; 8.20] | 6.8% |
| Common effect model | | 2234 | | 2262 | 0.61 | [0.08; 4.59] | 11.4% |
| Heterogeneity: $I^2 = 0\%$, $\tau^2 = 0$, $p = 0.61$ | | | | | | | |
| **Common effect model** | | **12165** | | **12144** | **0.28** | **[0.11; 0.68]** | **100.0%** |

Heterogeneity: $I^2 = 0\%$, $\tau^2 = 0$, $p = 0.99$

Test for subgroup differences: $\chi^2_2 = 0.69$, df = 2 ($p = 0.71$)

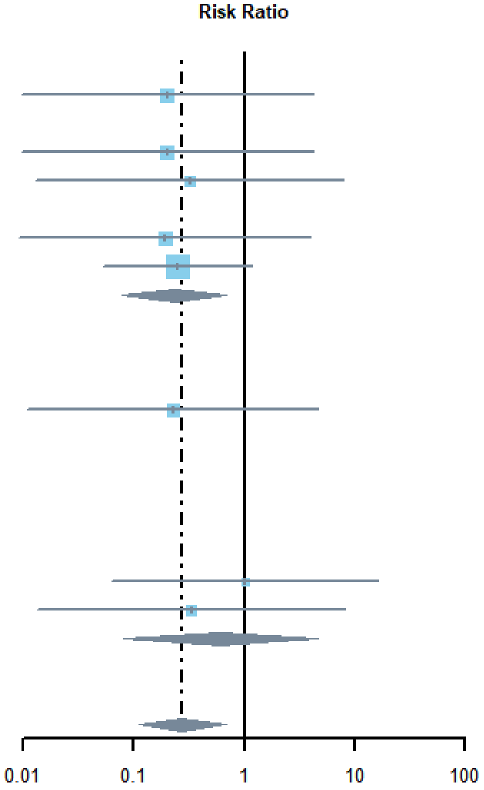

## 5.9 Single-drug analysis

**Outcome: Recurrent VTE during treatment**

| Study | Experimental Events | Experimental Total | Control Events | Control Total | RR | 95%-CI | Weight |
|---|---|---|---|---|---|---|---|
| t1 = Apixaban | | | | | | | |
| Agnelli-2013 | 14 | 840 | 73 | 829 | 0.20 | [0.12; 0.35] | 17.8% |
| Agnelli-2013 | 14 | 840 | 73 | 829 | 0.20 | [0.12; 0.35] | 17.8% |
| Random effects model | | 1680 | | 1658 | 0.20 | [0.14; 0.29] | 35.5% |
| Heterogeneity: $I^2 = 0\%$, $\tau^2 = 0$, $p = 1.00$ | | | | | | | |
| t1 = Rivaroxaban | | | | | | | |
| Prins-2018 | 8 | 598 | 42 | 590 | 0.21 | [0.10; 0.42] | 10.6% |
| Prins-2018 | 17 | 1107 | 42 | 590 | 0.22 | [0.13; 0.38] | 18.1% |
| Prins-2018 | 13 | 1127 | 42 | 590 | 0.17 | [0.09; 0.31] | 15.0% |
| Wells-2016 | 18 | 602 | 57 | 594 | 0.32 | [0.19; 0.54] | 20.8% |
| Random effects model | | 3434 | | 2364 | 0.23 | [0.17; 0.31] | 64.5% |
| Heterogeneity: $I^2 = 0\%$, $\tau^2 = 0.0049$, $p = 0.42$ | | | | | | | |
| **Random effects model** | | **5114** | | **4022** | **0.22** | **[0.18; 0.28]** | **100.0%** |
| Heterogeneity: $I^2 = 0\%$, $\tau^2 = 0$, $p = 0.67$ | | | | | | | |
| Test for subgroup differences: $\chi^2_1 = 0.37$, df = 1 ($p = 0.54$) | | | | | | | |

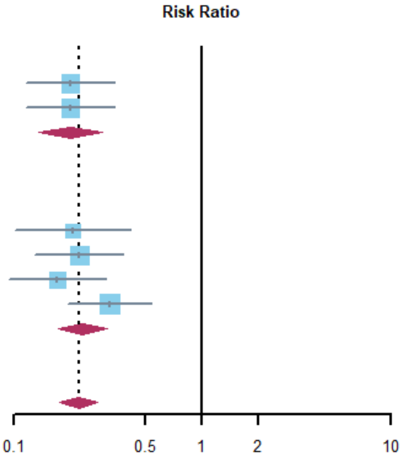


**Outcome: Major bleeding during treatment**

| Study | Experimental Events | Experimental Total | Control Events | Control Total | RR | 95%-CI | Weight |
|---|---|---|---|---|---|---|---|
| t1 = Apixaban | | | | | | | |
| Agnelli-2013 | 2 | 840 | 4 | 829 | 0.59 | [0.14; 2.47] | 20.7% |
| Agnelli-2013 | 1 | 840 | 4 | 829 | 0.39 | [0.08; 2.03] | 17.7% |
| Random effects model | | 1680 | | 1658 | 0.50 | [0.17; 1.46] | 38.4% |
| Heterogeneity: $I^2 = 0\%$, $\tau^2 = 0$, $p = 0.71$ | | | | | | | |
| t1 = Rivaroxaban | | | | | | | |
| Marshall-2020 | 2 | 46 | 0 | 46 | 3.00 | [0.32; 27.81] | 11.6% |
| Prins-2018 | 4 | 598 | 0 | 590 | 4.93 | [0.58; 42.10] | 12.3% |
| Prins-2018 | 6 | 1107 | 0 | 590 | 3.73 | [0.46; 30.28] | 12.7% |
| Prins-2018 | 5 | 1127 | 0 | 590 | 3.14 | [0.38; 26.05] | 12.6% |
| Wells-2016 | 4 | 602 | 0 | 594 | 4.93 | [0.58; 42.10] | 12.3% |
| Random effects model | | 3480 | | 2410 | 3.87 | [1.48; 10.09] | 61.6% |
| Heterogeneity: $I^2 = 0\%$, $\tau^2 = 0$, $p = 1.00$ | | | | | | | |
| **Random effects model** | | **5160** | | **4068** | **1.75** | **[0.73; 4.22]** | **100.0%** |
| Heterogeneity: $I^2 = 26\%$, $\tau^2 = 0.4403$, $p = 0.23$ | | | | | | | |
| Test for subgroup differences: $\chi^2_1 = 7.79$, df = 1 ($p < 0.01$) | | | | | | | |

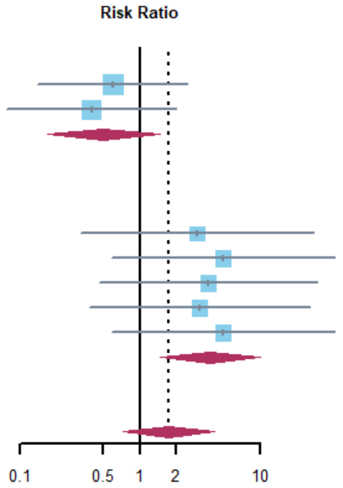

**Outcome: Clinical relevant non-major bleeding during treatment**

| Study | Experimental Events | Experimental Total | Control Events | Control Total | RR | 95%-CI | Weight |
|---|---|---|---|---|---|---|---|
| t1 = Apixaban | | | | | | | |
| Agnelli-2013 | 25 | 840 | 19 | 829 | 1.28 | [0.72; 2.28] | 24.7% |
| Agnelli-2013 | 34 | 813 | 19 | 829 | 1.78 | [1.04; 3.06] | 27.9% |
| Random effects model | | 1653 | | 1658 | 1.53 | [1.03; 2.27] | 52.6% |
| Heterogeneity: $I^2 = 0\%$, $\tau^2 = 0$, $p = 0.41$ | | | | | | | |
| t1 = Rivaroxaban | | | | | | | |
| Marshall-2020 | 2 | 46 | 0 | 46 | 3.00 | [0.32; 27.81] | 1.6% |
| Wells-2016 | 46 | 608 | 34 | 594 | 1.31 | [0.86; 2.00] | 45.7% |
| Random effects model | | 654 | | 640 | 1.35 | [0.89; 2.05] | 47.4% |
| Heterogeneity: $I^2 = 0\%$, $\tau^2 = 0$, $p = 0.47$ | | | | | | | |
| **Random effects model** | | **2307** | | **2298** | **1.44** | **[1.08; 1.92]** | **100.0%** |

Heterogeneity: $I^2 = 0\%$, $\tau^2 = 0$, $p = 0.71$

Test for subgroup differences: $\chi^2_1 = 0.18$, df = 1 ($p = 0.67$)

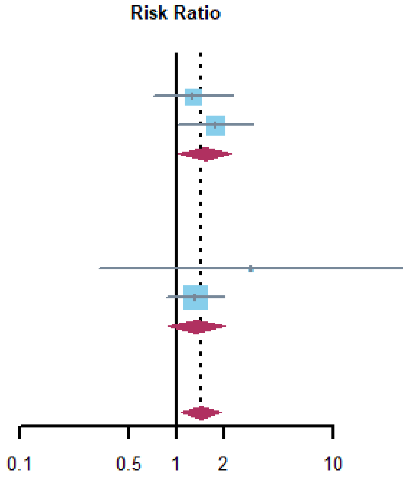


**Outcome: All-cause mortality during treatment**

| Study | Experimental Events | Experimental Total | Control Events | Control Total | RR | 95%-CI | Weight |
|---|---|---|---|---|---|---|---|
| t1 = Apixaban | | | | | | | |
| Agnelli-2013 | 20 | 840 | 86 | 829 | 0.238 | [0.149; 0.380] | 29.8% |
| Agnelli-2013 | 20 | 840 | 86 | 829 | 0.238 | [0.149; 0.380] | 29.8% |
| Random effects model | | 1680 | | 1658 | 0.238 | [0.171; 0.331] | 59.6% |
| Heterogeneity: $I^2 = 0\%$, $\tau^2 = 0$, $p = 1.00$ | | | | | | | |
| t1 = Rivaroxaban | | | | | | | |
| Marshall-2020 | 6 | 46 | 5 | 46 | 1.167 | [0.424; 3.212] | 20.0% |
| Wells-2016 | 5 | 602 | 10 | 594 | 0.538 | [0.200; 1.446] | 20.4% |
| Random effects model | | 648 | | 640 | 0.786 | [0.368; 1.677] | 40.4% |
| Heterogeneity: $I^2 = 13\%$, $\tau^2 = 0.0387$, $p = 0.28$ | | | | | | | |
| **Random effects model** | | **2328** | | **2298** | **0.387** | **[0.192; 0.780]** | **100.0%** |

Heterogeneity: $I^2 = 70\%$, $\tau^2 = 0.3740$, $p = 0.02$

Test for subgroup differences: $\chi^2_1 = 8.01$, df = 1 ($p < 0.01$)

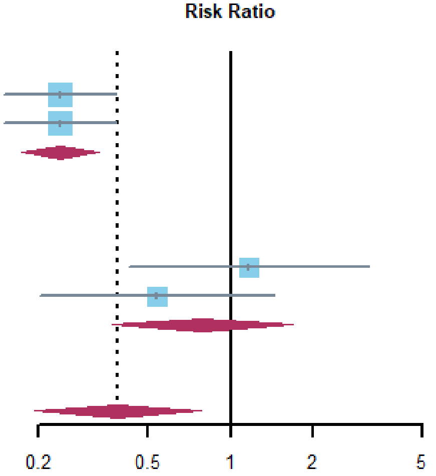

**Outcome: VTE during prophylaxis**

| Study | Experimental Events | Experimental Total | Control Events | Control Total | RR | 95%-CI | Weight |
|---|---|---|---|---|---|---|---|
| t1 = Apixaban | | | | | | | |
| Wang-2023 | 5 | 610 | 5 | 607 | 0.995 | [0.323; 3.068] | 4.6% |
| Levine-2012 | 0 | 32 | 3 | 29 | 0.227 | [0.027; 1.922] | 1.5% |
| Levine-2012 | 0 | 29 | 3 | 29 | 0.250 | [0.030; 2.108] | 1.5% |
| Levine-2012 | 0 | 32 | 3 | 29 | 0.227 | [0.027; 1.922] | 1.5% |
| Zhang-2021 | 6 | 237 | 7 | 232 | 0.857 | [0.316; 2.324] | 5.6% |
| Zhang-2021 | 2 | 51 | 5 | 43 | 0.423 | [0.112; 1.594] | 3.5% |
| Brandt-2022 | 6 | 126 | 17 | 91 | 0.282 | [0.123; 0.647] | 7.3% |
| Connors-2021 | 0 | 135 | 0 | 136 | | | 0.0% |
| Connors-2021 | 0 | 143 | 0 | 136 | | | 0.0% |
| Random effects model | | 1395 | | 1332 | 0.462 | [0.269; 0.795] | 25.5% |
| Heterogeneity: $I^2 = 0\%$, $\tau^2 = 0.0920$, $p = 0.45$ | | | | | | | |
| t1 = Rivaroxaban | | | | | | | |
| Vadhan-2020 | 13 | 135 | 18 | 138 | 0.753 | [0.394; 1.441] | 9.9% |
| Becattini-2022 | 3 | 287 | 11 | 282 | 0.328 | [0.107; 1.004] | 4.7% |
| Ramacciotti-2022 | 5 | 159 | 15 | 159 | 0.375 | [0.151; 0.934] | 6.4% |
| Ageno-2021 | 25 | 3853 | 32 | 3872 | 0.792 | [0.474; 1.321] | 12.6% |
| Ageno-2021 | 25 | 2154 | 34 | 2140 | 0.738 | [0.446; 1.222] | 12.8% |
| Spyropoulos-2018 | 11 | 6007 | 25 | 6012 | 0.462 | [0.233; 0.915] | 9.3% |
| Camporese-2016 | 2 | 120 | 7 | 114 | 0.356 | [0.097; 1.311] | 3.6% |
| Random effects model | | 12715 | | 12717 | 0.620 | [0.479; 0.804] | 59.4% |
| Heterogeneity: $I^2 = 0\%$, $\tau^2 = 0$, $p = 0.48$ | | | | | | | |
| t1 = TTP889 | | | | | | | |
| Eriksson-2008 | 33 | 109 | 29 | 103 | 1.072 | [0.710; 1.617] | 15.0% |
| **Random effects model** | | **14219** | | **14152** | **0.602** | **[0.459; 0.789]** | **100.0%** |

Heterogeneity: $I^2 = 26\%$, $\tau^2 = 0.0832$, $p = 0.17$
Test for subgroup differences: $\chi^2_2 = 7.12$, df = 2 ($p = 0.03$)

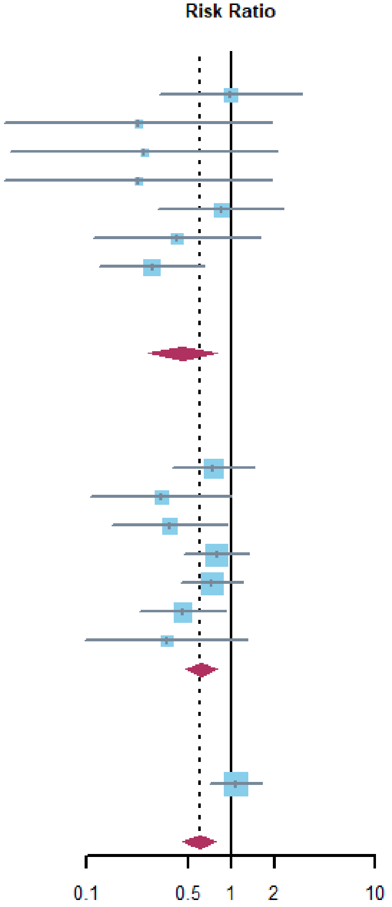

**Outcome: Major bleeding during prophylaxis**

| Study | Experimental Events | Experimental Total | Control Events | Control Total | RR | 95%-CI | Weight |
|---|---|---|---|---|---|---|---|
| t1 = Apixaban | | | | | | | |
| Wang-2023 | 2 | 610 | 11 | 607 | 0.25 | [0.07; 0.88] | 9.9% |
| Levine-2012 | 0 | 32 | 1 | 29 | 0.45 | [0.04; 4.76] | 3.3% |
| Levine-2012 | 0 | 29 | 1 | 29 | 0.50 | [0.05; 5.22] | 3.3% |
| Levine-2012 | 2 | 32 | 1 | 29 | 1.36 | [0.24; 7.61] | 5.8% |
| Zhang-2021 | 4 | 237 | 2 | 232 | 1.63 | [0.39; 6.75] | 8.1% |
| Zhang-2021 | 2 | 51 | 1 | 43 | 1.27 | [0.22; 7.26] | 5.6% |
| Brandt-2022 | 2 | 126 | 2 | 91 | 0.72 | [0.15; 3.51] | 6.8% |
| Connors-2021 | 0 | 135 | 0 | 136 | | | 0.0% |
| Connors-2021 | 0 | 143 | 0 | 136 | | | 0.0% |
| Random effects model | | 1395 | | 1332 | 0.73 | [0.37; 1.43] | 42.7% |
| Heterogeneity: $I^2 = 0\%$, $\tau^2 = 0.1008$, $p = 0.52$ | | | | | | | |
| t1 = Rivaroxaban | | | | | | | |
| Vadhan-2020 | 2 | 130 | 3 | 131 | 0.76 | [0.17; 3.31] | 7.6% |
| Becattini-2022 | 2 | 287 | 0 | 282 | 2.95 | [0.31; 28.17] | 3.5% |
| Ramacciotti-2022 | 0 | 159 | 0 | 159 | | | 0.0% |
| Ageno-2021 | 10 | 3837 | 7 | 3855 | 1.38 | [0.56; 3.43] | 16.5% |
| Ageno-2021 | 7 | 2145 | 2 | 2125 | 2.64 | [0.70; 9.94] | 9.1% |
| Spyropoulos-2018 | 17 | 5982 | 9 | 5980 | 1.80 | [0.83; 3.89] | 20.6% |
| Camporese-2016 | 0 | 120 | 0 | 114 | | | 0.0% |
| Random effects model | | 12660 | | 12646 | 1.63 | [1.00; 2.67] | 57.3% |
| Heterogeneity: $I^2 = 0\%$, $\tau^2 = 0$, $p = 0.73$ | | | | | | | |
| t1 = TTP889 | | | | | | | |
| Eriksson-2008 | 0 | 129 | 0 | 132 | | | 0.0% |
| **Random effects model** | | **14184** | | **14110** | **1.15** | **[0.75; 1.78]** | **100.0%** |

Heterogeneity: $I^2 = 2\%$, $\tau^2 = 0.0853$, $p = 0.42$

Test for subgroup differences: $\chi^2_1 = 3.60$, df = 1 ($p = 0.06$)

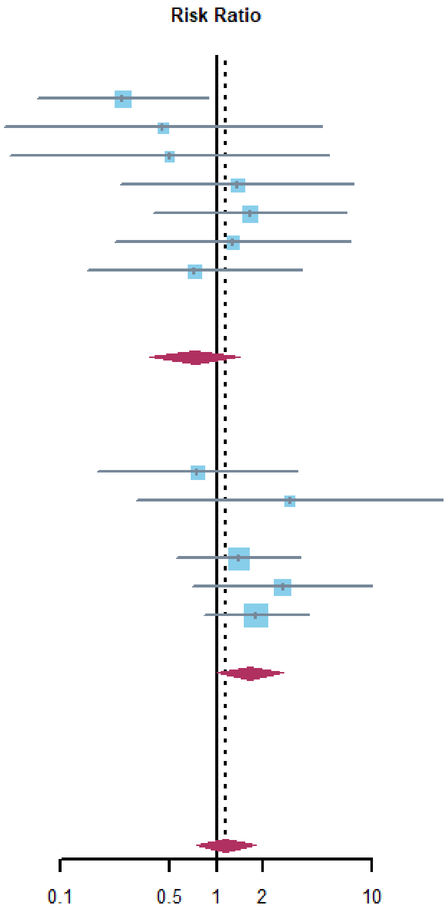

**Outcome: All-cause mortality during prophylaxis**

| Study | Experimental Events | Experimental Total | Control Events | Control Total | RR | 95%-CI | Weight |
|---|---|---|---|---|---|---|---|
| t1 = Apixaban | | | | | | | |
| Wang-1 | 8 | 610 | 9 | 607 | 0.90 | [0.37; 2.19] | 8.6% |
| Carrier-25 | 35 | 288 | 27 | 275 | 1.23 | [0.77; 1.96] | 27.7% |
| Zhang-59 | 1 | 237 | 4 | 232 | 0.39 | [0.08; 2.00] | 2.7% |
| Zhang-60 | 0 | 51 | 1 | 43 | 0.42 | [0.04; 4.51] | 1.3% |
| Brandt-73 | 1 | 126 | 2 | 91 | 0.48 | [0.08; 2.83] | 2.3% |
| Connors-82 | 0 | 135 | 0 | 136 | | | 0.0% |
| Connors-83 | 0 | 143 | 0 | 136 | | | 0.0% |
| Random effects model | | 1590 | | 1520 | 0.96 | [0.61; 1.50] | 42.6% |
| Heterogeneity: $I^2 = 0\%$, $\tau^2 = 0.0302$, $p = 0.52$ | | | | | | | |
| t1 = Rivaroxaban | | | | | | | |
| Becattini-24 | 0 | 287 | 1 | 282 | 0.49 | [0.04; 5.39] | 1.3% |
| Spyropoulos-45 | 71 | 6007 | 89 | 6012 | 0.80 | [0.59; 1.09] | 51.9% |
| Piazza-69 | 2 | 641 | 2 | 643 | 1.00 | [0.20; 4.95] | 2.8% |
| Random effects model | | 6935 | | 6937 | 0.80 | [0.59; 1.08] | 56.0% |
| Heterogeneity: $I^2 = 0\%$, $\tau^2 = 0$, $p = 0.89$ | | | | | | | |
| t1 = TTP889 | | | | | | | |
| Eriksson-43 | 2 | 129 | 0 | 131 | 3.05 | [0.32; 28.91] | 1.4% |
| **Random effects model** | | **8654** | | **8588** | **0.89** | **[0.68; 1.17]** | **100.0%** |

Heterogeneity: $I^2 = 0\%$, $\tau^2 = 0.0117$, $p = 0.70$

Test for subgroup differences: $\chi^2_2 = 1.65$, df = 2 ($p = 0.44$)

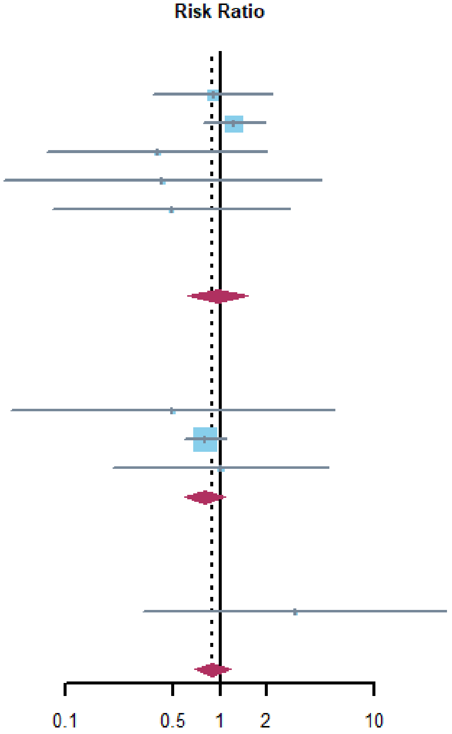

**Outcome: Clinical relevant non-major bleeding during prophylaxis**

| Study | Experimental Events | Experimental Total | Control Events | Control Total | RR | 95%-CI | Weight |
|---|---|---|---|---|---|---|---|
| t1 = Apixaban | | | | | | | |
| Wang-2023 | 3 | 610 | 6 | 607 | 0.57 | [0.17; 1.93] | 5.9% |
| Levine-2012 | 1 | 32 | 0 | 29 | 1.82 | [0.17; 19.05] | 1.7% |
| Levine-2012 | 1 | 29 | 0 | 29 | 2.00 | [0.19; 20.90] | 1.7% |
| Levine-2012 | 2 | 32 | 0 | 29 | 2.73 | [0.30; 24.82] | 1.9% |
| Zhang-2021 | 15 | 237 | 14 | 232 | 1.04 | [0.53; 2.06] | 17.6% |
| Connors-2021 | 1 | 135 | 0 | 136 | 2.01 | [0.18; 21.96] | 1.6% |
| Connors-2021 | 1 | 143 | 0 | 136 | 1.90 | [0.17; 20.75] | 1.6% |
| Random effects model | | 1218 | | 1198 | 1.11 | [0.66; 1.86] | 31.9% |
| Heterogeneity: $I^2 = 0\%$, $\tau^2 = 0$, $p = 0.85$ | | | | | | | |
| t1 = Rivaroxaban | | | | | | | |
| Vadhan-2020 | 5 | 130 | 2 | 131 | 2.02 | [0.51; 7.89] | 4.8% |
| Becattini-2022 | 3 | 287 | 5 | 282 | 0.66 | [0.19; 2.30] | 5.6% |
| Ramacciotti-2022 | 2 | 159 | 2 | 159 | 1.00 | [0.20; 4.88] | 3.6% |
| Spyropoulos-2018 | 85 | 5982 | 51 | 5980 | 1.65 | [1.17; 2.33] | 51.2% |
| Random effects model | | 6558 | | 6552 | 1.55 | [1.13; 2.12] | 65.2% |
| Heterogeneity: $I^2 = 0\%$, $\tau^2 = 0$, $p = 0.50$ | | | | | | | |
| t1 = TTP889 | | | | | | | |
| Eriksson-2008 | 2 | 129 | 1 | 132 | 1.53 | [0.26; 9.03] | 2.9% |
| **Random effects model** | | **7905** | | **7882** | **1.37** | **[1.01; 1.86]** | **100.0%** |

Heterogeneity: $I^2 = 0\%$, $\tau^2 = 0.0165$, $p = 0.86$

Test for subgroup differences: $\chi^2_2 = 1.16$, df = 2 ($p = 0.56$)

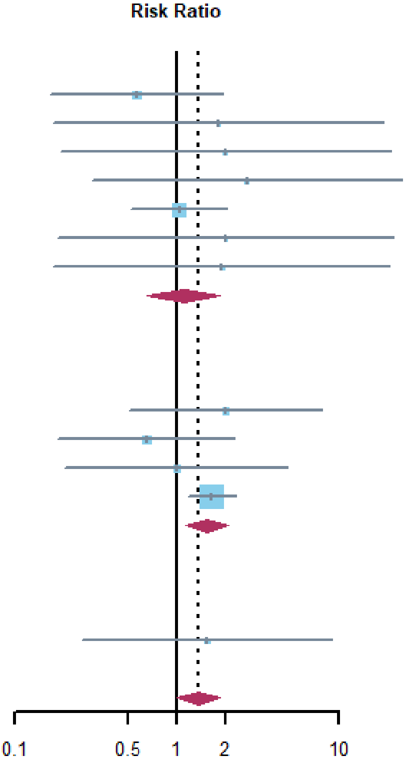

**5.10 Sensitive analysis**
**Outcome：Recurrent VTE during treatment**

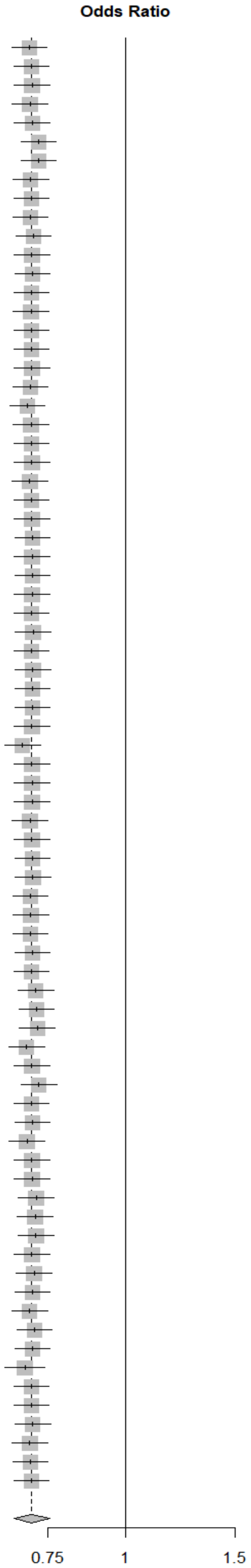


| Study | OR | 95%-CI | P-value | Tau2 | Tau | I2 |
|---|---|---|---|---|---|---|
| Omitting Raskob-2016 | 0.70 | [0.66; 0.75] | < 0.01 | 0.4018 | 0.6339 | 76% |
| Omitting Raskob-2016 | 0.71 | [0.66; 0.75] | < 0.01 | 0.4029 | 0.6347 | 76% |
| Omitting Raskob-2016 | 0.71 | [0.66; 0.76] | < 0.01 | 0.3976 | 0.6306 | 76% |
| Omitting Schulman-2009 | 0.70 | [0.66; 0.75] | < 0.01 | 0.4012 | 0.6334 | 76% |
| Omitting Raskob-2018 | 0.71 | [0.66; 0.76] | < 0.01 | 0.4054 | 0.6367 | 76% |
| Omitting Agnelli-2013 | 0.73 | [0.68; 0.77] | < 0.01 | 0.3678 | 0.6064 | 74% |
| Omitting Agnelli-2013 | 0.73 | [0.68; 0.77] | < 0.01 | 0.3678 | 0.6064 | 74% |
| Omitting Koopman-1996 | 0.70 | [0.66; 0.75] | < 0.01 | 0.3980 | 0.6309 | 76% |
| Omitting Medina-2017 | 0.71 | [0.66; 0.75] | < 0.01 | 0.4037 | 0.6354 | 76% |
| Omitting Medina-2017 | 0.70 | [0.66; 0.75] | < 0.01 | 0.4006 | 0.6329 | 76% |
| Omitting Buller-2007 | 0.71 | [0.67; 0.76] | < 0.01 | 0.3916 | 0.6258 | 75% |
| Omitting Planquette-2022 | 0.71 | [0.66; 0.76] | < 0.01 | 0.4007 | 0.6330 | 76% |
| Omitting Righini-2016 | 0.71 | [0.66; 0.76] | < 0.01 | 0.4005 | 0.6328 | 76% |
| Omitting Findik-2002 | 0.71 | [0.66; 0.75] | < 0.01 | 0.3948 | 0.6283 | 76% |
| Omitting Girolami-2004 | 0.71 | [0.66; 0.75] | < 0.01 | 0.4006 | 0.6329 | 76% |
| Omitting Belcaro-1999 | 0.71 | [0.66; 0.75] | < 0.01 | 0.4002 | 0.6326 | 76% |
| Omitting Belcaro-1999 | 0.71 | [0.66; 0.75] | < 0.01 | 0.3992 | 0.6318 | 76% |
| Omitting Levine-1996 | 0.71 | [0.66; 0.75] | < 0.01 | 0.4035 | 0.6352 | 76% |
| Omitting Romera-2009 | 0.70 | [0.66; 0.75] | < 0.01 | 0.3900 | 0.6245 | 76% |
| Omitting Ridker-2003 | 0.70 | [0.65; 0.74] | < 0.01 | 0.3663 | 0.6052 | 74% |
| Omitting Kearon-2006 | 0.71 | [0.66; 0.75] | < 0.01 | 0.4001 | 0.6325 | 76% |
| Omitting Monreal-1999 | 0.71 | [0.66; 0.75] | < 0.01 | 0.3956 | 0.6290 | 76% |
| Omitting Monreal-1999 | 0.71 | [0.66; 0.76] | < 0.01 | 0.4001 | 0.6325 | 76% |
| Omitting Riess-2003 | 0.70 | [0.66; 0.75] | < 0.01 | 0.3966 | 0.6297 | 76% |
| Omitting Simonneau-1997 | 0.71 | [0.66; 0.75] | < 0.01 | 0.3989 | 0.6316 | 76% |
| Omitting Kang-2019 | 0.71 | [0.66; 0.75] | < 0.01 | 0.3997 | 0.6322 | 76% |
| Omitting Ramacciotti-2004 | 0.71 | [0.66; 0.76] | < 0.01 | 0.3941 | 0.6278 | 76% |
| Omitting Bynum-1979 | 0.71 | [0.66; 0.76] | < 0.01 | 0.4009 | 0.6331 | 76% |
| Omitting Marshall-2020 | 0.71 | [0.66; 0.76] | < 0.01 | 0.3970 | 0.6301 | 76% |
| Omitting Merli-2001 | 0.71 | [0.66; 0.76] | < 0.01 | 0.4029 | 0.6347 | 76% |
| Omitting Merli-2001 | 0.71 | [0.66; 0.75] | < 0.01 | 0.4007 | 0.6330 | 76% |
| Omitting Gonzalez-1999 | 0.71 | [0.67; 0.76] | < 0.01 | 0.3937 | 0.6274 | 76% |
| Omitting Wells-2005 | 0.71 | [0.66; 0.75] | < 0.01 | 0.4018 | 0.6338 | 76% |
| Omitting Raskob-2016 | 0.71 | [0.67; 0.76] | < 0.01 | 0.4024 | 0.6343 | 76% |
| Omitting Raskob-2016 | 0.71 | [0.66; 0.76] | < 0.01 | 0.4002 | 0.6326 | 76% |
| Omitting Raskob-2016 | 0.71 | [0.66; 0.76] | < 0.01 | 0.3953 | 0.6287 | 76% |
| Omitting Lopaciuk-1999 | 0.71 | [0.66; 0.76] | < 0.01 | 0.4020 | 0.6340 | 76% |
| Omitting Simes-2014 | 0.68 | [0.64; 0.73] | < 0.01 | 0.3906 | 0.6250 | 74% |
| Omitting Meyer-2002 | 0.71 | [0.66; 0.76] | < 0.01 | 0.3990 | 0.6316 | 76% |
| Omitting Schrag-2023 | 0.71 | [0.66; 0.76] | < 0.01 | 0.4045 | 0.6360 | 76% |
| Omitting Agnelli-2020 | 0.71 | [0.66; 0.76] | < 0.01 | 0.4052 | 0.6366 | 76% |
| Omitting Schulman-2014 | 0.70 | [0.66; 0.75] | < 0.01 | 0.4008 | 0.6331 | 76% |
| Omitting Nisio-2019 | 0.71 | [0.66; 0.76] | < 0.01 | 0.4052 | 0.6366 | 76% |
| Omitting Nisio-2019 | 0.71 | [0.66; 0.76] | < 0.01 | 0.3999 | 0.6324 | 76% |
| Omitting Becattini-2011 | 0.71 | [0.67; 0.76] | < 0.01 | 0.4041 | 0.6357 | 76% |
| Omitting Breddin-2001 | 0.70 | [0.66; 0.75] | < 0.01 | 0.3848 | 0.6203 | 75% |
| Omitting Breddin-2001 | 0.70 | [0.66; 0.75] | < 0.01 | 0.3921 | 0.6261 | 76% |
| Omitting Buller-1997 | 0.70 | [0.66; 0.75] | < 0.01 | 0.4006 | 0.6330 | 76% |
| Omitting Bauersachs-2018 | 0.71 | [0.66; 0.76] | < 0.01 | 0.4046 | 0.6361 | 76% |
| Omitting Bauersachs-2018 | 0.71 | [0.66; 0.75] | < 0.01 | 0.4016 | 0.6337 | 76% |
| Omitting Prins-2018 | 0.72 | [0.67; 0.77] | < 0.01 | 0.3757 | 0.6129 | 75% |
| Omitting Prins-2018 | 0.72 | [0.67; 0.77] | < 0.01 | 0.3751 | 0.6125 | 74% |
| Omitting Prins-2018 | 0.72 | [0.68; 0.77] | < 0.01 | 0.3624 | 0.6020 | 74% |
| Omitting Prins-2018 | 0.69 | [0.65; 0.74] | < 0.01 | 0.3875 | 0.6225 | 75% |
| Omitting Jimenez-2007 | 0.71 | [0.66; 0.76] | < 0.01 | 0.4008 | 0.6331 | 76% |
| Omitting Eriksson-2005 | 0.73 | [0.68; 0.78] | < 0.01 | 0.3619 | 0.6016 | 74% |
| Omitting Veiga-2000 | 0.71 | [0.66; 0.75] | < 0.01 | 0.3973 | 0.6303 | 76% |
| Omitting Nakamura-2015 | 0.71 | [0.66; 0.76] | < 0.01 | 0.4041 | 0.6357 | 76% |
| Omitting Nakamura-2015 | 0.69 | [0.65; 0.74] | < 0.01 | 0.4036 | 0.6353 | 76% |
| Omitting Cirujeda-2006 | 0.71 | [0.66; 0.76] | < 0.01 | 0.3998 | 0.6323 | 76% |
| Omitting Perez-2010 | 0.71 | [0.66; 0.76] | < 0.01 | 0.3966 | 0.6298 | 76% |
| Omitting Wells-2016 | 0.72 | [0.67; 0.77] | < 0.01 | 0.3895 | 0.6241 | 75% |
| Omitting Weitz-2017 | 0.72 | [0.67; 0.76] | < 0.01 | 0.3944 | 0.6280 | 75% |
| Omitting Weitz-2017 | 0.72 | [0.67; 0.77] | < 0.01 | 0.3855 | 0.6209 | 75% |
| Omitting Buller-2011 | 0.71 | [0.66; 0.76] | < 0.01 | 0.4029 | 0.6347 | 76% |
| Omitting Lee-2003 | 0.71 | [0.67; 0.76] | < 0.01 | 0.3960 | 0.6293 | 75% |
| Omitting Harenberg-2000 | 0.71 | [0.66; 0.76] | < 0.01 | 0.3991 | 0.6317 | 76% |
| Omitting Schulman-2013 | 0.70 | [0.66; 0.75] | < 0.01 | 0.3937 | 0.6275 | 75% |
| Omitting Schulman-2013 | 0.71 | [0.67; 0.76] | < 0.01 | 0.3791 | 0.6157 | 75% |
| Omitting Harenberg-2001 | 0.71 | [0.66; 0.76] | < 0.01 | 0.3948 | 0.6283 | 76% |
| Omitting Weycker-2018 | 0.69 | [0.64; 0.74] | < 0.01 | 0.4062 | 0.6373 | 76% |
| Omitting Wysokinski-2019 | 0.71 | [0.66; 0.75] | < 0.01 | 0.4008 | 0.6331 | 76% |
| Omitting Wysokinski-2019 | 0.71 | [0.66; 0.75] | < 0.01 | 0.4018 | 0.6339 | 76% |
| Omitting Harenberg-2003 | 0.71 | [0.66; 0.76] | < 0.01 | 0.4047 | 0.6362 | 76% |
| Omitting Young-2018 | 0.70 | [0.66; 0.75] | < 0.01 | 0.3831 | 0.6190 | 75% |
| Omitting Hull-1979 | 0.70 | [0.66; 0.75] | < 0.01 | 0.3888 | 0.6236 | 75% |
| Omitting Beyer-2017 | 0.71 | [0.66; 0.75] | < 0.01 | 0.3910 | 0.6253 | 76% |
| **Common effect model** | **0.71** | **[0.66; 0.76]** | **< 0.01** | **0.3954** | **0.6288** | **76%** |

**Outcome：Major bleeding during treatment**

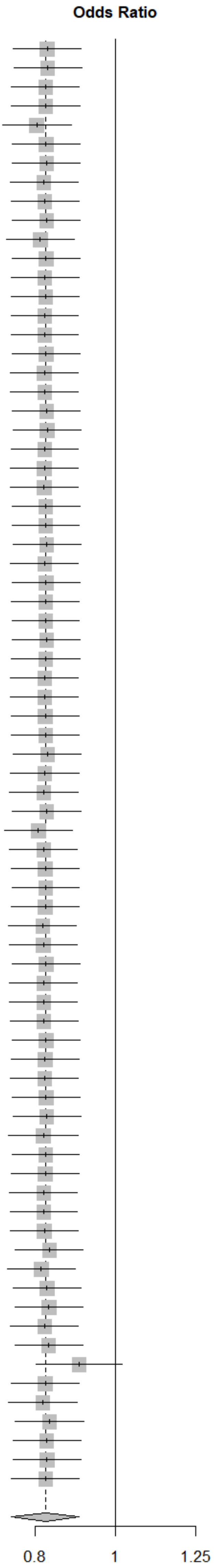


| Study | OR | 95%-CI | P-value | Tau2 | Tau | I2 |
|---|---|---|---|---|---|---|
| Omitting Raskob-2016 | 0.83 | [0.75; 0.91] | < 0.01 | 0.0450 | 0.2121 | 19% |
| Omitting Raskob-2016 | 0.83 | [0.75; 0.91] | < 0.01 | 0.0442 | 0.2102 | 17% |
| Omitting Raskob-2016 | 0.82 | [0.75; 0.91] | < 0.01 | 0.0471 | 0.2171 | 20% |
| Omitting Schulman-2009 | 0.82 | [0.75; 0.91] | < 0.01 | 0.0513 | 0.2265 | 20% |
| Omitting Raskob-2018 | 0.80 | [0.73; 0.88] | < 0.01 | 0.0205 | 0.1433 | 12% |
| Omitting Agnelli-2013 | 0.83 | [0.75; 0.91] | < 0.01 | 0.0459 | 0.2141 | 19% |
| Omitting Agnelli-2013 | 0.83 | [0.75; 0.91] | < 0.01 | 0.0452 | 0.2125 | 19% |
| Omitting Koopman-1996 | 0.82 | [0.75; 0.90] | < 0.01 | 0.0442 | 0.2102 | 18% |
| Omitting Medina-2017 | 0.82 | [0.75; 0.90] | < 0.01 | 0.0477 | 0.2185 | 20% |
| Omitting Medina-2017 | 0.83 | [0.75; 0.91] | < 0.01 | 0.0473 | 0.2175 | 20% |
| Omitting Buller-2007 | 0.81 | [0.74; 0.89] | < 0.01 | 0.0430 | 0.2075 | 14% |
| Omitting Planquette-2022 | 0.83 | [0.75; 0.91] | < 0.01 | 0.0455 | 0.2132 | 19% |
| Omitting Righini-2016 | 0.82 | [0.75; 0.90] | < 0.01 | 0.0450 | 0.2121 | 19% |
| Omitting Findik-2002 | 0.82 | [0.75; 0.90] | < 0.01 | 0.0450 | 0.2121 | 19% |
| Omitting Girolami-2004 | 0.82 | [0.75; 0.90] | < 0.01 | 0.0458 | 0.2140 | 19% |
| Omitting Belcaro-1999 | 0.82 | [0.75; 0.90] | < 0.01 | 0.0457 | 0.2139 | 19% |
| Omitting Belcaro-1999 | 0.83 | [0.75; 0.91] | < 0.01 | 0.0454 | 0.2131 | 19% |
| Omitting Levine-1996 | 0.82 | [0.75; 0.90] | < 0.01 | 0.0450 | 0.2122 | 19% |
| Omitting Romera-2009 | 0.82 | [0.75; 0.90] | < 0.01 | 0.0447 | 0.2115 | 19% |
| Omitting Ridker-2003 | 0.83 | [0.75; 0.91] | < 0.01 | 0.0455 | 0.2134 | 19% |
| Omitting Kearon-2006 | 0.83 | [0.75; 0.91] | < 0.01 | 0.0454 | 0.2132 | 19% |
| Omitting Monreal-1999 | 0.82 | [0.75; 0.90] | < 0.01 | 0.0453 | 0.2129 | 19% |
| Omitting Riess-2003 | 0.82 | [0.75; 0.90] | < 0.01 | 0.0463 | 0.2153 | 19% |
| Omitting Simonneau-1997 | 0.82 | [0.75; 0.90] | < 0.01 | 0.0459 | 0.2143 | 19% |
| Omitting Kang-2019 | 0.82 | [0.75; 0.91] | < 0.01 | 0.0453 | 0.2128 | 19% |
| Omitting Ramacciotti-2004 | 0.82 | [0.75; 0.91] | < 0.01 | 0.0459 | 0.2143 | 20% |
| Omitting Bynum-1979 | 0.83 | [0.75; 0.91] | < 0.01 | 0.0449 | 0.2119 | 18% |
| Omitting Marshall-2020 | 0.82 | [0.75; 0.90] | < 0.01 | 0.0447 | 0.2115 | 18% |
| Omitting Merli-2001 | 0.83 | [0.75; 0.91] | < 0.01 | 0.0466 | 0.2158 | 20% |
| Omitting Merli-2001 | 0.82 | [0.75; 0.91] | < 0.01 | 0.0471 | 0.2170 | 20% |
| Omitting Gonzalez-1999 | 0.82 | [0.75; 0.91] | < 0.01 | 0.0455 | 0.2134 | 20% |
| Omitting Wells-2005 | 0.83 | [0.75; 0.91] | < 0.01 | 0.0455 | 0.2134 | 19% |
| Omitting Raskob-2016 | 0.82 | [0.75; 0.91] | < 0.01 | 0.0488 | 0.2210 | 20% |
| Omitting Raskob-2016 | 0.82 | [0.75; 0.90] | < 0.01 | 0.0454 | 0.2131 | 19% |
| Omitting Raskob-2016 | 0.82 | [0.75; 0.90] | < 0.01 | 0.0451 | 0.2124 | 19% |
| Omitting Lopaciuk-1999 | 0.82 | [0.75; 0.90] | < 0.01 | 0.0454 | 0.2131 | 20% |
| Omitting Simes-2014 | 0.82 | [0.75; 0.91] | < 0.01 | 0.0479 | 0.2188 | 20% |
| Omitting Meyer-2002 | 0.83 | [0.75; 0.91] | < 0.01 | 0.0441 | 0.2099 | 18% |
| Omitting Schrag-2023 | 0.82 | [0.75; 0.90] | < 0.01 | 0.0500 | 0.2235 | 20% |
| Omitting Agnelli-2020 | 0.82 | [0.75; 0.90] | < 0.01 | 0.0507 | 0.2252 | 19% |
| Omitting Schulman-2014 | 0.83 | [0.75; 0.91] | < 0.01 | 0.0488 | 0.2209 | 19% |
| Omitting Nisio-2019 | 0.81 | [0.73; 0.89] | < 0.01 | 0.0281 | 0.1676 | 14% |
| Omitting Nisio-2019 | 0.82 | [0.75; 0.90] | < 0.01 | 0.0436 | 0.2088 | 18% |
| Omitting Becattini-2011 | 0.82 | [0.75; 0.90] | < 0.01 | 0.0454 | 0.2131 | 20% |
| Omitting Breddin-2001 | 0.82 | [0.75; 0.91] | < 0.01 | 0.0460 | 0.2145 | 20% |
| Omitting Breddin-2001 | 0.82 | [0.75; 0.90] | < 0.01 | 0.0458 | 0.2140 | 20% |
| Omitting Buller-1997 | 0.82 | [0.74; 0.90] | < 0.01 | 0.0444 | 0.2106 | 18% |
| Omitting Bauersachs-2018 | 0.82 | [0.75; 0.90] | < 0.01 | 0.0441 | 0.2100 | 18% |
| Omitting Bauersachs-2018 | 0.83 | [0.75; 0.91] | < 0.01 | 0.0460 | 0.2146 | 19% |
| Omitting Prins-2018 | 0.82 | [0.75; 0.90] | < 0.01 | 0.0443 | 0.2104 | 17% |
| Omitting Prins-2018 | 0.82 | [0.75; 0.90] | < 0.01 | 0.0444 | 0.2107 | 18% |
| Omitting Prins-2018 | 0.82 | [0.75; 0.90] | < 0.01 | 0.0446 | 0.2111 | 18% |
| Omitting Prins-2018 | 0.83 | [0.75; 0.91] | < 0.01 | 0.0453 | 0.2128 | 19% |
| Omitting Jimenez-2007 | 0.82 | [0.75; 0.90] | < 0.01 | 0.0484 | 0.2199 | 20% |
| Omitting Eriksson-2005 | 0.82 | [0.75; 0.90] | < 0.01 | 0.0464 | 0.2153 | 19% |
| Omitting Veiga-2000 | 0.83 | [0.75; 0.91] | < 0.01 | 0.0452 | 0.2126 | 19% |
| Omitting Nakamura-2015 | 0.83 | [0.75; 0.91] | < 0.01 | 0.0471 | 0.2170 | 19% |
| Omitting Nakamura-2015 | 0.82 | [0.74; 0.90] | < 0.01 | 0.0546 | 0.2336 | 19% |
| Omitting Cirujeda-2006 | 0.82 | [0.75; 0.91] | < 0.01 | 0.0453 | 0.2129 | 19% |
| Omitting Perez-2010 | 0.82 | [0.75; 0.90] | < 0.01 | 0.0454 | 0.2130 | 20% |
| Omitting Wells-2016 | 0.82 | [0.75; 0.90] | < 0.01 | 0.0443 | 0.2104 | 17% |
| Omitting Weitz-2017 | 0.82 | [0.75; 0.90] | < 0.01 | 0.0441 | 0.2100 | 18% |
| Omitting Weitz-2017 | 0.82 | [0.75; 0.90] | < 0.01 | 0.0451 | 0.2124 | 19% |
| Omitting Buller-2011 | 0.83 | [0.76; 0.92] | < 0.01 | 0.0399 | 0.1996 | 15% |
| Omitting Lee-2003 | 0.81 | [0.74; 0.90] | < 0.01 | 0.0390 | 0.1976 | 17% |
| Omitting Harenberg-2000 | 0.83 | [0.75; 0.91] | < 0.01 | 0.0461 | 0.2146 | 19% |
| Omitting Schulman-2013 | 0.83 | [0.76; 0.91] | < 0.01 | 0.0423 | 0.2056 | 18% |
| Omitting Schulman-2013 | 0.82 | [0.75; 0.90] | < 0.01 | 0.0448 | 0.2116 | 19% |
| Omitting Harenberg-2001 | 0.83 | [0.76; 0.91] | < 0.01 | 0.0411 | 0.2028 | 17% |
| Omitting Weycker-2018 | 0.91 | [0.80; 1.02] | 0.10 | 0.0465 | 0.2156 | 15% |
| Omitting Wysokinski-2019 | 0.82 | [0.75; 0.91] | < 0.01 | 0.0496 | 0.2228 | 20% |
| Omitting Wysokinski-2019 | 0.82 | [0.74; 0.90] | < 0.01 | 0.0450 | 0.2121 | 18% |
| Omitting Harenberg-2003 | 0.83 | [0.76; 0.92] | < 0.01 | 0.0419 | 0.2048 | 18% |
| Omitting Young-2018 | 0.83 | [0.75; 0.91] | < 0.01 | 0.0461 | 0.2146 | 19% |
| Omitting Hull-1979 | 0.83 | [0.75; 0.91] | < 0.01 | 0.0449 | 0.2119 | 18% |
| Omitting Beyer-2017 | 0.82 | [0.75; 0.90] | < 0.01 | 0.0450 | 0.2121 | 19% |
| **Common effect model** | **0.82** | **[0.75; 0.90]** | **< 0.01** | **0.0450** | **0.2121** | **19%** |

**Outcome：Clinical relevant non-major bleeding during treatment**

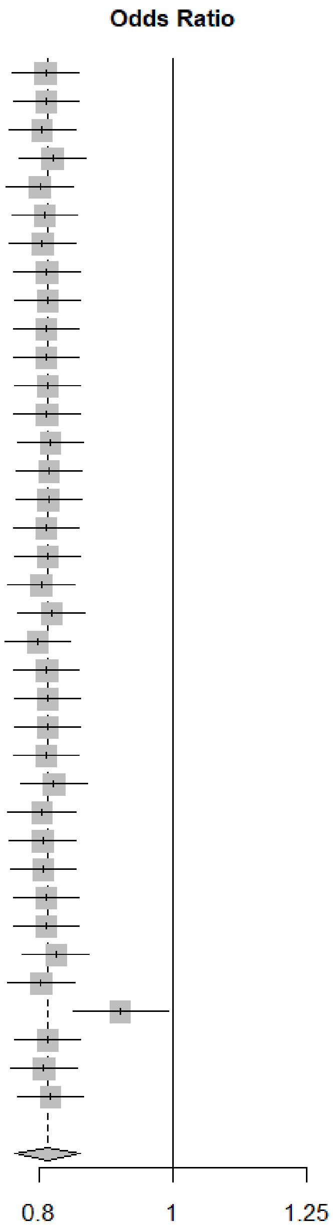


| Study | OR | 95%-CI | P-value | Tau2 | Tau | I2 |
|---|---|---|---|---|---|---|
| Omitting Raskob-2016 | 0.81 | [0.77; 0.85] | < 0.01 | 0.1780 | 0.4219 | 73% |
| Omitting Raskob-2016 | 0.81 | [0.77; 0.86] | < 0.01 | 0.1798 | 0.4240 | 73% |
| Omitting Raskob-2016 | 0.80 | [0.76; 0.85] | < 0.01 | 0.1792 | 0.4233 | 73% |
| Omitting Schulman-2009 | 0.82 | [0.77; 0.87] | < 0.01 | 0.1652 | 0.4064 | 73% |
| Omitting Raskob-2018 | 0.80 | [0.76; 0.85] | < 0.01 | 0.1731 | 0.4161 | 72% |
| Omitting Agnelli-2013 | 0.81 | [0.76; 0.85] | < 0.01 | 0.1747 | 0.4180 | 73% |
| Omitting Agnelli-2013 | 0.80 | [0.76; 0.85] | < 0.01 | 0.1616 | 0.4020 | 72% |
| Omitting Medina-2017 | 0.81 | [0.77; 0.86] | < 0.01 | 0.1796 | 0.4238 | 73% |
| Omitting Medina-2017 | 0.81 | [0.77; 0.86] | < 0.01 | 0.1793 | 0.4234 | 74% |
| Omitting Planquette-2022 | 0.81 | [0.77; 0.86] | < 0.01 | 0.1694 | 0.4116 | 73% |
| Omitting Righini-2016 | 0.81 | [0.77; 0.85] | < 0.01 | 0.1675 | 0.4092 | 73% |
| Omitting Kang-2019 | 0.81 | [0.77; 0.86] | < 0.01 | 0.1708 | 0.4133 | 73% |
| Omitting Marshall-2020 | 0.81 | [0.77; 0.86] | < 0.01 | 0.1692 | 0.4113 | 73% |
| Omitting Raskob-2016 | 0.82 | [0.77; 0.86] | < 0.01 | 0.1690 | 0.4112 | 73% |
| Omitting Raskob-2016 | 0.81 | [0.77; 0.86] | < 0.01 | 0.1710 | 0.4135 | 73% |
| Omitting Raskob-2016 | 0.81 | [0.77; 0.86] | < 0.01 | 0.1705 | 0.4130 | 73% |
| Omitting Beyer-2017 | 0.81 | [0.77; 0.86] | < 0.01 | 0.1675 | 0.4092 | 73% |
| Omitting Simes-2014 | 0.81 | [0.77; 0.86] | < 0.01 | 0.1722 | 0.4149 | 73% |
| Omitting Agnelli-2020 | 0.80 | [0.76; 0.85] | < 0.01 | 0.1683 | 0.4102 | 72% |
| Omitting Schulman-2014 | 0.82 | [0.77; 0.86] | < 0.01 | 0.1690 | 0.4111 | 73% |
| Omitting Nisio-2019 | 0.80 | [0.76; 0.84] | < 0.01 | 0.1656 | 0.4070 | 71% |
| Omitting Nisio-2019 | 0.81 | [0.77; 0.86] | < 0.01 | 0.1767 | 0.4204 | 73% |
| Omitting Becattini-2011 | 0.81 | [0.77; 0.86] | < 0.01 | 0.1724 | 0.4152 | 73% |
| Omitting Bauersachs-2018 | 0.81 | [0.77; 0.86] | < 0.01 | 0.1717 | 0.4144 | 73% |
| Omitting Bauersachs-2018 | 0.81 | [0.77; 0.86] | < 0.01 | 0.1704 | 0.4128 | 73% |
| Omitting Nakamura-2015 | 0.82 | [0.78; 0.87] | < 0.01 | 0.1576 | 0.3970 | 72% |
| Omitting Nakamura-2015 | 0.80 | [0.76; 0.85] | < 0.01 | 0.1820 | 0.4266 | 73% |
| Omitting Wells-2016 | 0.81 | [0.76; 0.85] | < 0.01 | 0.1740 | 0.4171 | 72% |
| Omitting Weitz-2017 | 0.81 | [0.76; 0.85] | < 0.01 | 0.1697 | 0.4119 | 72% |
| Omitting Weitz-2017 | 0.81 | [0.77; 0.85] | < 0.01 | 0.1775 | 0.4213 | 73% |
| Omitting Buller-2011 | 0.81 | [0.77; 0.85] | < 0.01 | 0.1748 | 0.4181 | 73% |
| Omitting Schulman-2013 | 0.82 | [0.78; 0.87] | < 0.01 | 0.1602 | 0.4002 | 72% |
| Omitting Schulman-2013 | 0.80 | [0.76; 0.85] | < 0.01 | 0.1430 | 0.3782 | 70% |
| Omitting Weycker-2018 | 0.92 | [0.85; 0.99] | 0.03 | 0.1768 | 0.4205 | 70% |
| Omitting Wysokinski-2019 | 0.81 | [0.77; 0.86] | < 0.01 | 0.1744 | 0.4176 | 74% |
| Omitting Wysokinski-2019 | 0.81 | [0.76; 0.85] | < 0.01 | 0.1512 | 0.3888 | 71% |
| Omitting Young-2018 | 0.82 | [0.77; 0.86] | < 0.01 | 0.1491 | 0.3861 | 72% |
| **Common effect model** | **0.81** | **[0.77; 0.86]** | **< 0.01** | **0.1696** | **0.4119** | **73%** |

**Outcome：VTE related death during treatment**

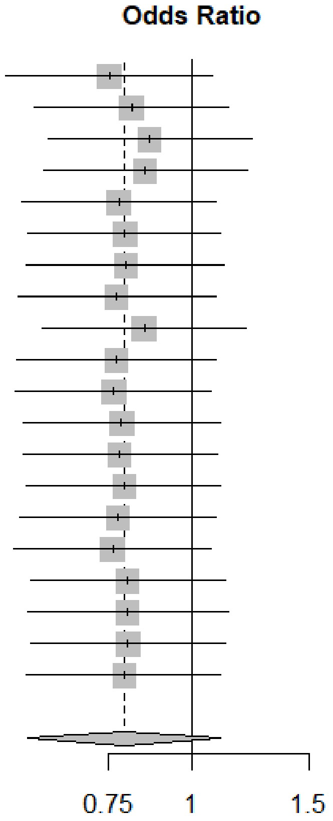


| Study | OR | 95%-CI | P-value | Tau2 | Tau | I2 |
|---|---|---|---|---|---|---|
| Omitting Raskob-2016 | 0.75 | [0.52; 1.08] | 0.12 | 0 | 0 | 0% |
| Omitting Schulman-2009 | 0.81 | [0.58; 1.14] | 0.23 | 0 | 0 | 0% |
| Omitting Agnelli-2013 | 0.86 | [0.61; 1.23] | 0.42 | 0 | 0 | 0% |
| Omitting Agnelli-2013 | 0.85 | [0.60; 1.21] | 0.37 | 0 | 0 | 0% |
| Omitting Buller-2007 | 0.78 | [0.55; 1.09] | 0.14 | 0 | 0 | 0% |
| Omitting Findik-2002 | 0.79 | [0.57; 1.11] | 0.17 | 0 | 0 | 0% |
| Omitting Girolami-2004 | 0.79 | [0.56; 1.12] | 0.19 | 0 | 0 | 0% |
| Omitting Agnelli-2020 | 0.77 | [0.55; 1.09] | 0.14 | 0 | 0 | 0% |
| Omitting Schulman-2014 | 0.85 | [0.59; 1.21] | 0.36 | 0 | 0 | 0% |
| Omitting Becattini-2011 | 0.77 | [0.54; 1.09] | 0.14 | 0 | 0 | 0% |
| Omitting Breddin-2001 | 0.76 | [0.54; 1.07] | 0.12 | 0 | 0 | 0% |
| Omitting Breddin-2001 | 0.78 | [0.56; 1.10] | 0.16 | 0 | 0 | 0% |
| Omitting Veiga-2000 | 0.78 | [0.56; 1.09] | 0.14 | 0 | 0 | 0% |
| Omitting Nakamura-2015 | 0.79 | [0.56; 1.11] | 0.17 | 0 | 0 | 0% |
| Omitting Nakamura-2015 | 0.77 | [0.55; 1.09] | 0.14 | 0 | 0 | 0% |
| Omitting Weitz-2017 | 0.76 | [0.54; 1.07] | 0.12 | 0 | 0 | 0% |
| Omitting Weitz-2017 | 0.80 | [0.57; 1.12] | 0.20 | 0 | 0 | 0% |
| Omitting Lee-2003 | 0.80 | [0.56; 1.14] | 0.21 | 0 | 0 | 0% |
| Omitting Harenberg-2000 | 0.80 | [0.57; 1.12] | 0.20 | 0 | 0 | 0% |
| Omitting Young-2018 | 0.79 | [0.56; 1.11] | 0.17 | 0 | 0 | 0% |
| **Common effect model** | **0.79** | **[0.57; 1.11]** | **0.17** | **0** | **0** | **0%** |

**Outcome: Fatal bleeding during treatment**

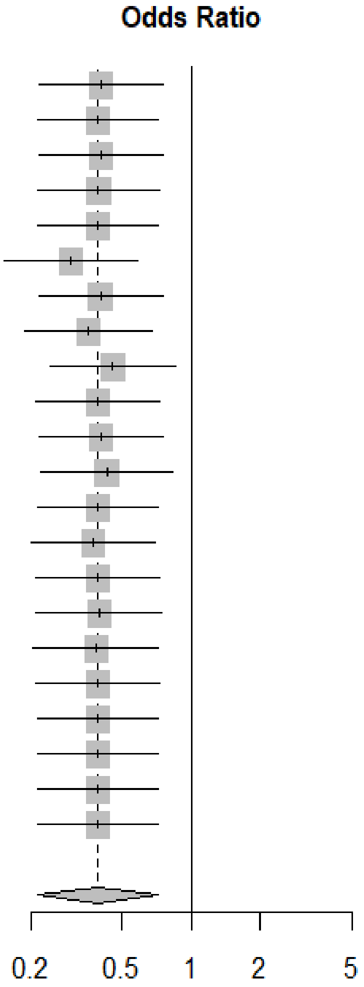


| Study | OR | 95%-CI | P-value | Tau2 | Tau | I2 |
|---|---|---|---|---|---|---|
| Omitting Raskob-2016 | 0.40 | [0.22; 0.75] | < 0.01 | 0 | 0 | 0% |
| Omitting Raskob-2016 | 0.39 | [0.21; 0.72] | < 0.01 | 0 | 0 | 0% |
| Omitting Raskob-2016 | 0.40 | [0.22; 0.75] | < 0.01 | 0 | 0 | 0% |
| Omitting Medina-2017 | 0.39 | [0.21; 0.73] | < 0.01 | 0 | 0 | 0% |
| Omitting Medina-2017 | 0.39 | [0.21; 0.72] | < 0.01 | 0 | 0 | 0% |
| Omitting Buller-2007 | 0.30 | [0.15; 0.59] | < 0.01 | 0 | 0 | 0% |
| Omitting Planquette-2022 | 0.40 | [0.22; 0.75] | < 0.01 | 0 | 0 | 0% |
| Omitting Girolami-2004 | 0.36 | [0.19; 0.67] | < 0.01 | 0 | 0 | 0% |
| Omitting Meyer-2002 | 0.46 | [0.24; 0.86] | 0.01 | 0 | 0 | 0% |
| Omitting Schulman-2014 | 0.39 | [0.21; 0.73] | < 0.01 | 0 | 0 | 0% |
| Omitting Nakamura-2015 | 0.41 | [0.22; 0.75] | < 0.01 | 0 | 0 | 0% |
| Omitting Nakamura-2015 | 0.43 | [0.22; 0.83] | 0.01 | 0 | 0 | 0% |
| Omitting Wells-2016 | 0.39 | [0.21; 0.72] | < 0.01 | 0 | 0 | 0% |
| Omitting Weitz-2017 | 0.37 | [0.20; 0.70] | < 0.01 | 0 | 0 | 0% |
| Omitting Weitz-2017 | 0.39 | [0.21; 0.73] | < 0.01 | 0 | 0 | 0% |
| Omitting Buller-2011 | 0.40 | [0.21; 0.75] | < 0.01 | 0 | 0 | 0% |
| Omitting Harenberg-2000 | 0.38 | [0.21; 0.72] | < 0.01 | 0 | 0 | 0% |
| Omitting Schulman-2013 | 0.39 | [0.21; 0.73] | < 0.01 | 0 | 0 | 0% |
| Omitting Agnelli-2007 | 0.39 | [0.21; 0.72] | < 0.01 | 0 | 0 | 0% |
| Omitting Agnelli-2007 | 0.39 | [0.21; 0.72] | < 0.01 | 0 | 0 | 0% |
| Omitting Agnelli-2007 | 0.39 | [0.21; 0.72] | < 0.01 | 0 | 0 | 0% |
| Omitting Agnelli-2007 | 0.39 | [0.21; 0.72] | < 0.01 | 0 | 0 | 0% |
| **Common effect model** | **0.39** | **[0.21; 0.72]** | **< 0.01** | **0** | **0** | **0%** |

**Outcome: Adverse events during treatment**

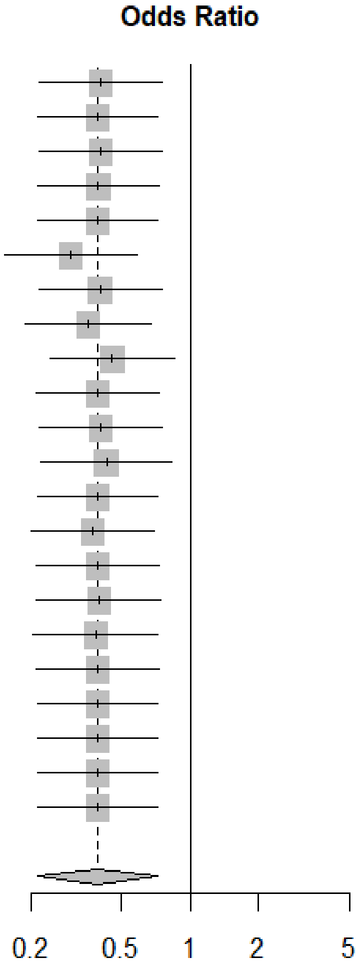


| Study | OR | 95%-CI | P-value | Tau2 | Tau | I2 |
|---|---|---|---|---|---|---|
| Omitting Raskob-2016 | 0.40 | [0.22; 0.75] | < 0.01 | 0 | 0 | 0% |
| Omitting Raskob-2016 | 0.39 | [0.21; 0.72] | < 0.01 | 0 | 0 | 0% |
| Omitting Raskob-2016 | 0.40 | [0.22; 0.75] | < 0.01 | 0 | 0 | 0% |
| Omitting Medina-2017 | 0.39 | [0.21; 0.73] | < 0.01 | 0 | 0 | 0% |
| Omitting Medina-2017 | 0.39 | [0.21; 0.72] | < 0.01 | 0 | 0 | 0% |
| Omitting Buller-2007 | 0.30 | [0.15; 0.59] | < 0.01 | 0 | 0 | 0% |
| Omitting Planquette-2022 | 0.40 | [0.22; 0.75] | < 0.01 | 0 | 0 | 0% |
| Omitting Girolami-2004 | 0.36 | [0.19; 0.67] | < 0.01 | 0 | 0 | 0% |
| Omitting Meyer-2002 | 0.46 | [0.24; 0.86] | 0.01 | 0 | 0 | 0% |
| Omitting Schulman-2014 | 0.39 | [0.21; 0.73] | < 0.01 | 0 | 0 | 0% |
| Omitting Nakamura-2015 | 0.41 | [0.22; 0.75] | < 0.01 | 0 | 0 | 0% |
| Omitting Nakamura-2015 | 0.43 | [0.22; 0.83] | 0.01 | 0 | 0 | 0% |
| Omitting Wells-2016 | 0.39 | [0.21; 0.72] | < 0.01 | 0 | 0 | 0% |
| Omitting Weitz-2017 | 0.37 | [0.20; 0.70] | < 0.01 | 0 | 0 | 0% |
| Omitting Weitz-2017 | 0.39 | [0.21; 0.73] | < 0.01 | 0 | 0 | 0% |
| Omitting Buller-2011 | 0.40 | [0.21; 0.75] | < 0.01 | 0 | 0 | 0% |
| Omitting Harenberg-2000 | 0.38 | [0.21; 0.72] | < 0.01 | 0 | 0 | 0% |
| Omitting Schulman-2013 | 0.39 | [0.21; 0.73] | < 0.01 | 0 | 0 | 0% |
| Omitting Agnelli-2007 | 0.39 | [0.21; 0.72] | < 0.01 | 0 | 0 | 0% |
| Omitting Agnelli-2007 | 0.39 | [0.21; 0.72] | < 0.01 | 0 | 0 | 0% |
| Omitting Agnelli-2007 | 0.39 | [0.21; 0.72] | < 0.01 | 0 | 0 | 0% |
| Omitting Agnelli-2007 | 0.39 | [0.21; 0.72] | < 0.01 | 0 | 0 | 0% |
| **Common effect model** | **0.39** | **[0.21; 0.72]** | **< 0.01** | **0** | **0** | **0%** |

**Outcome: All-cause mortality during treatment**

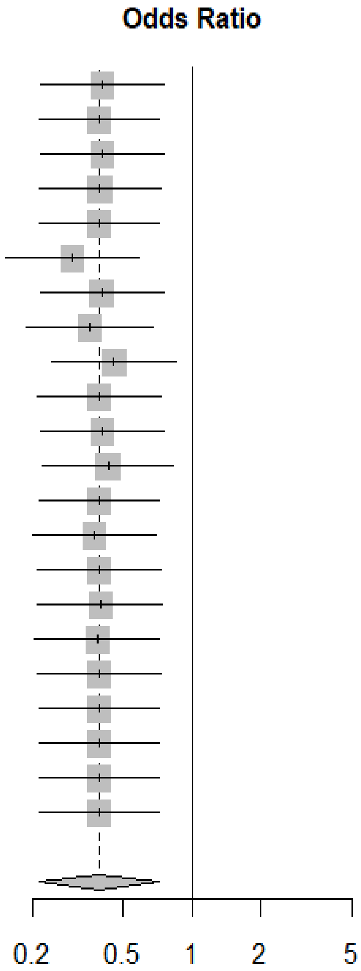


| Study | OR | 95%-CI | P-value | Tau2 | Tau | I2 |
|---|---|---|---|---|---|---|
| Omitting Raskob-2016 | 0.40 | [0.22; 0.75] | < 0.01 | 0 | 0 | 0% |
| Omitting Raskob-2016 | 0.39 | [0.21; 0.72] | < 0.01 | 0 | 0 | 0% |
| Omitting Raskob-2016 | 0.40 | [0.22; 0.75] | < 0.01 | 0 | 0 | 0% |
| Omitting Medina-2017 | 0.39 | [0.21; 0.73] | < 0.01 | 0 | 0 | 0% |
| Omitting Medina-2017 | 0.39 | [0.21; 0.72] | < 0.01 | 0 | 0 | 0% |
| Omitting Buller-2007 | 0.30 | [0.15; 0.59] | < 0.01 | 0 | 0 | 0% |
| Omitting Planquette-2022 | 0.40 | [0.22; 0.75] | < 0.01 | 0 | 0 | 0% |
| Omitting Girolami-2004 | 0.36 | [0.19; 0.67] | < 0.01 | 0 | 0 | 0% |
| Omitting Meyer-2002 | 0.46 | [0.24; 0.86] | 0.01 | 0 | 0 | 0% |
| Omitting Schulman-2014 | 0.39 | [0.21; 0.73] | < 0.01 | 0 | 0 | 0% |
| Omitting Nakamura-2015 | 0.41 | [0.22; 0.75] | < 0.01 | 0 | 0 | 0% |
| Omitting Nakamura-2015 | 0.43 | [0.22; 0.83] | 0.01 | 0 | 0 | 0% |
| Omitting Wells-2016 | 0.39 | [0.21; 0.72] | < 0.01 | 0 | 0 | 0% |
| Omitting Weitz-2017 | 0.37 | [0.20; 0.70] | < 0.01 | 0 | 0 | 0% |
| Omitting Weitz-2017 | 0.39 | [0.21; 0.73] | < 0.01 | 0 | 0 | 0% |
| Omitting Buller-2011 | 0.40 | [0.21; 0.75] | < 0.01 | 0 | 0 | 0% |
| Omitting Harenberg-2000 | 0.38 | [0.21; 0.72] | < 0.01 | 0 | 0 | 0% |
| Omitting Schulman-2013 | 0.39 | [0.21; 0.73] | < 0.01 | 0 | 0 | 0% |
| Omitting Agnelli-2007 | 0.39 | [0.21; 0.72] | < 0.01 | 0 | 0 | 0% |
| Omitting Agnelli-2007 | 0.39 | [0.21; 0.72] | < 0.01 | 0 | 0 | 0% |
| Omitting Agnelli-2007 | 0.39 | [0.21; 0.72] | < 0.01 | 0 | 0 | 0% |
| Omitting Agnelli-2007 | 0.39 | [0.21; 0.72] | < 0.01 | 0 | 0 | 0% |
| **Common effect model** | **0.39** | **[0.21; 0.72]** | **< 0.01** | **0** | **0** | **0%** |

**5.11 SUCRA ranking diagram**

**Outcome：Recurrent VTE during treatment**

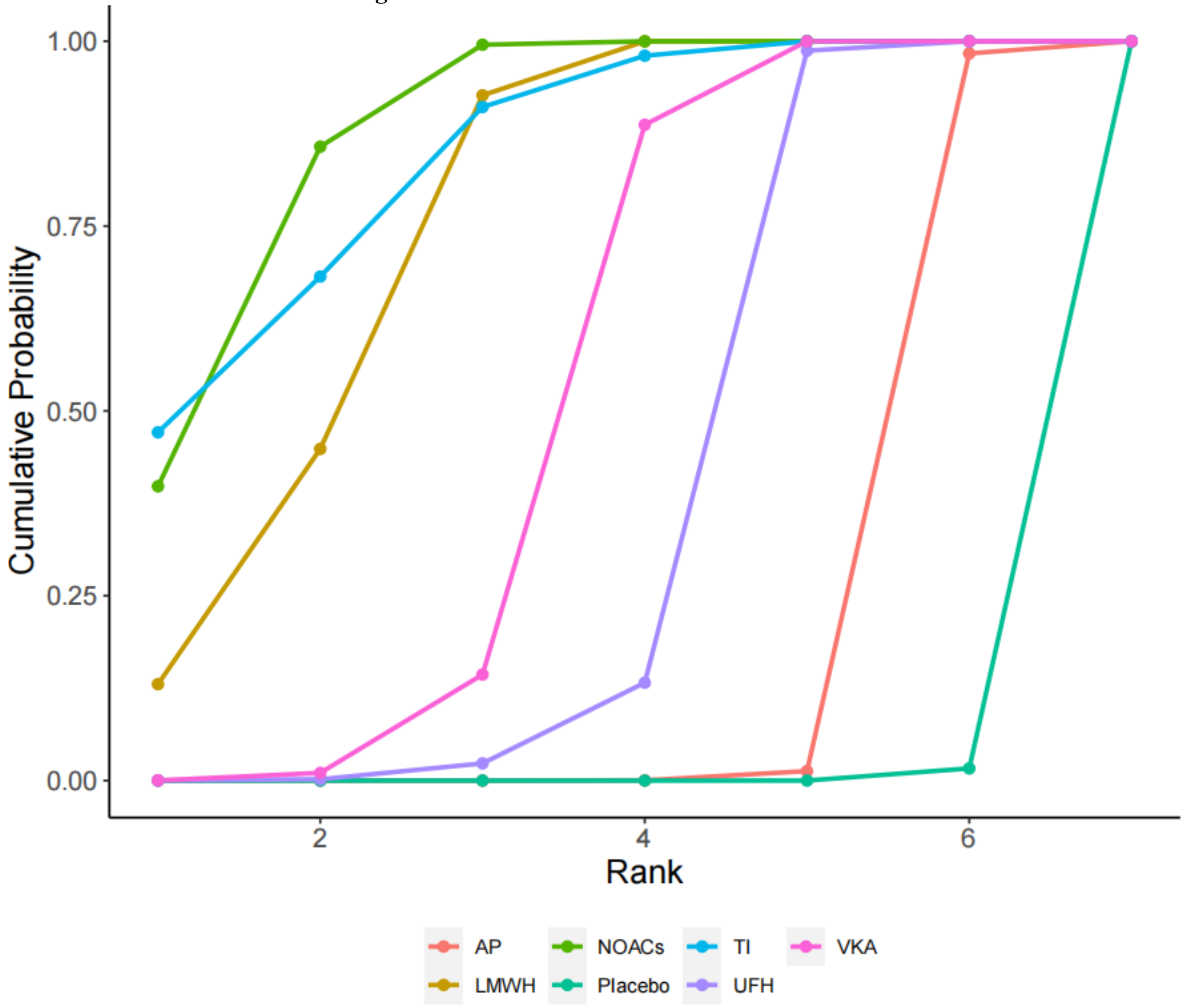


**Outcome：Major bleeding during treatment**

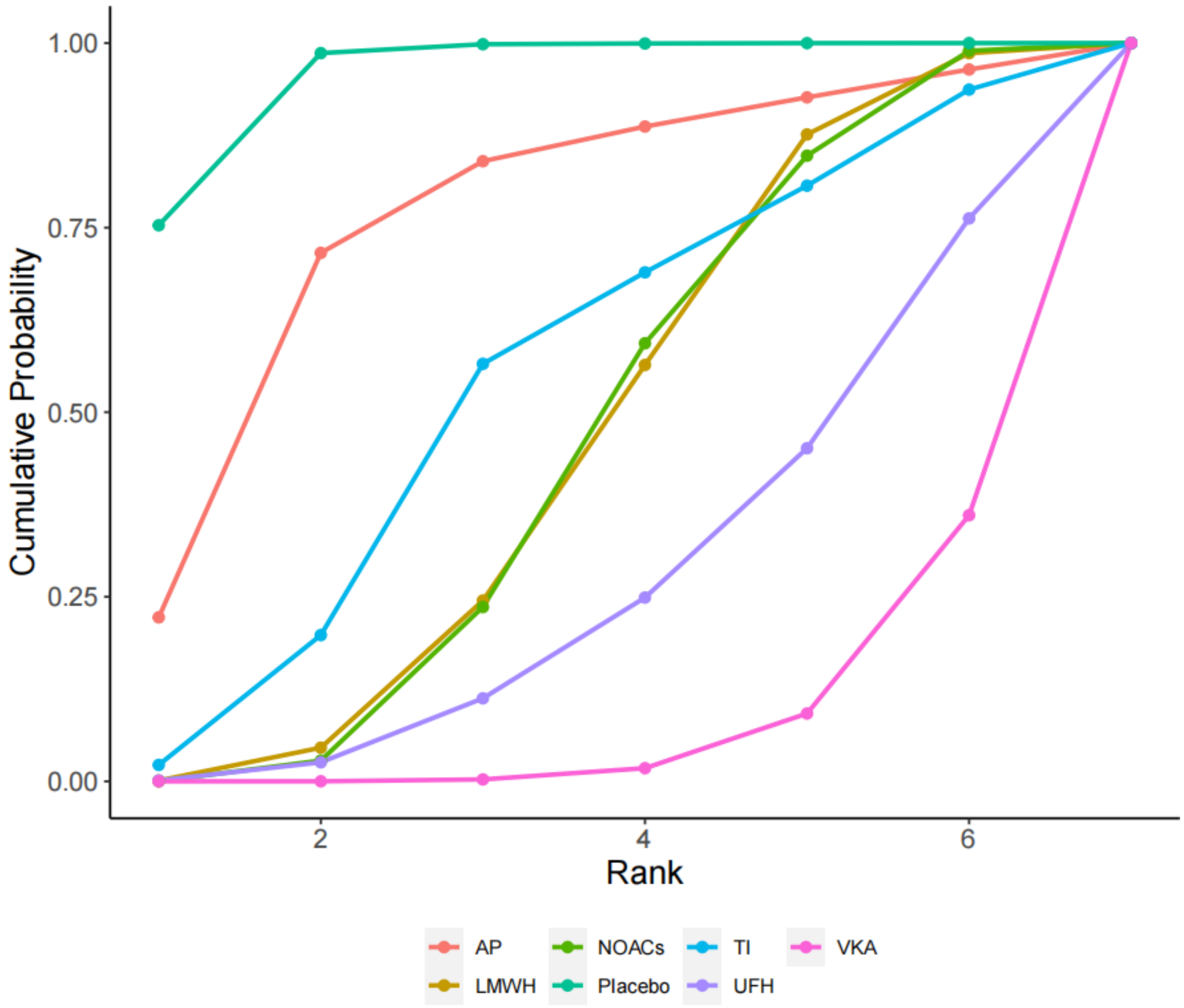

**Outcome：Clinical relevant non-major bleeding during treatment**

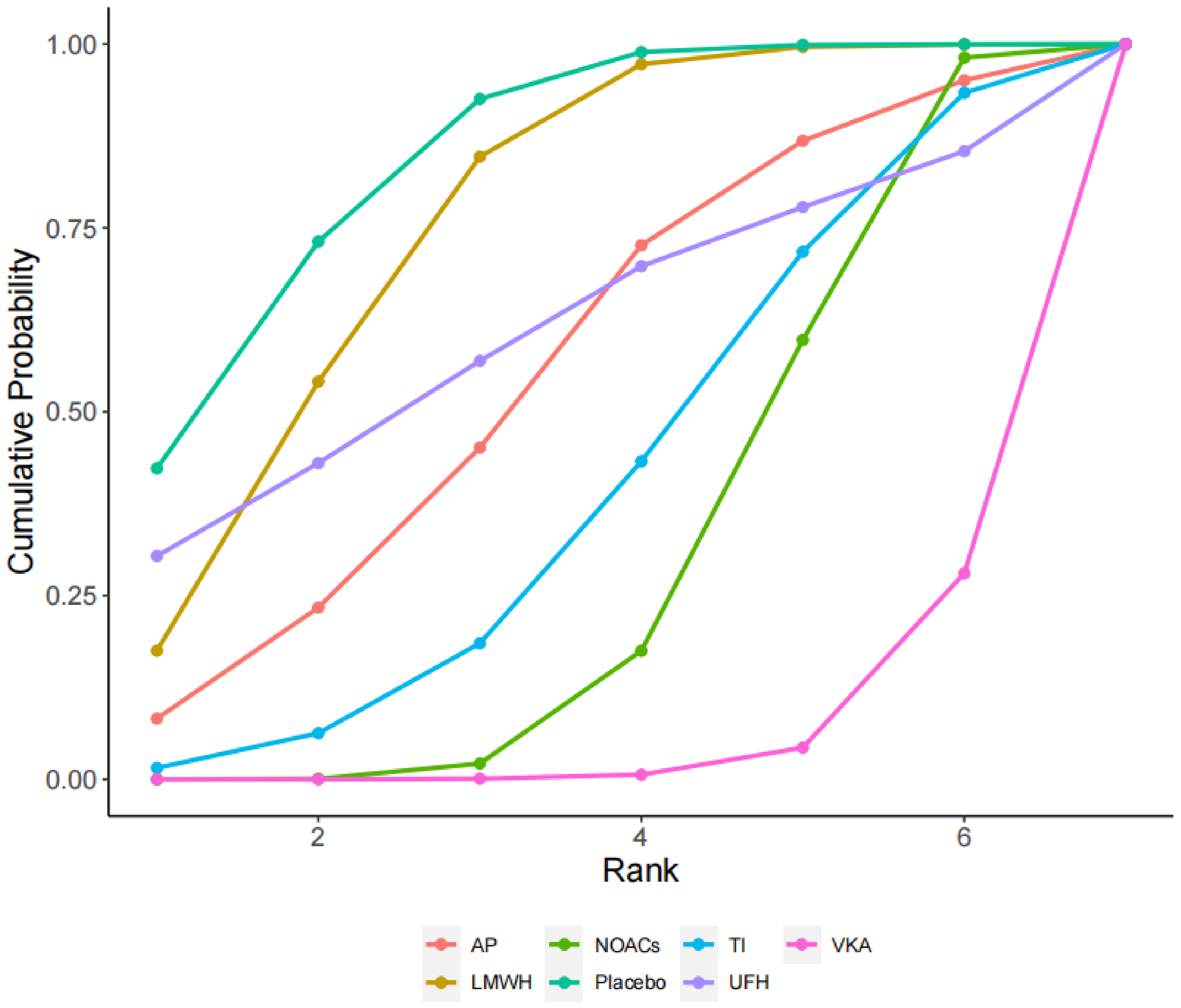


**Outcome：VTE related death during treatment**

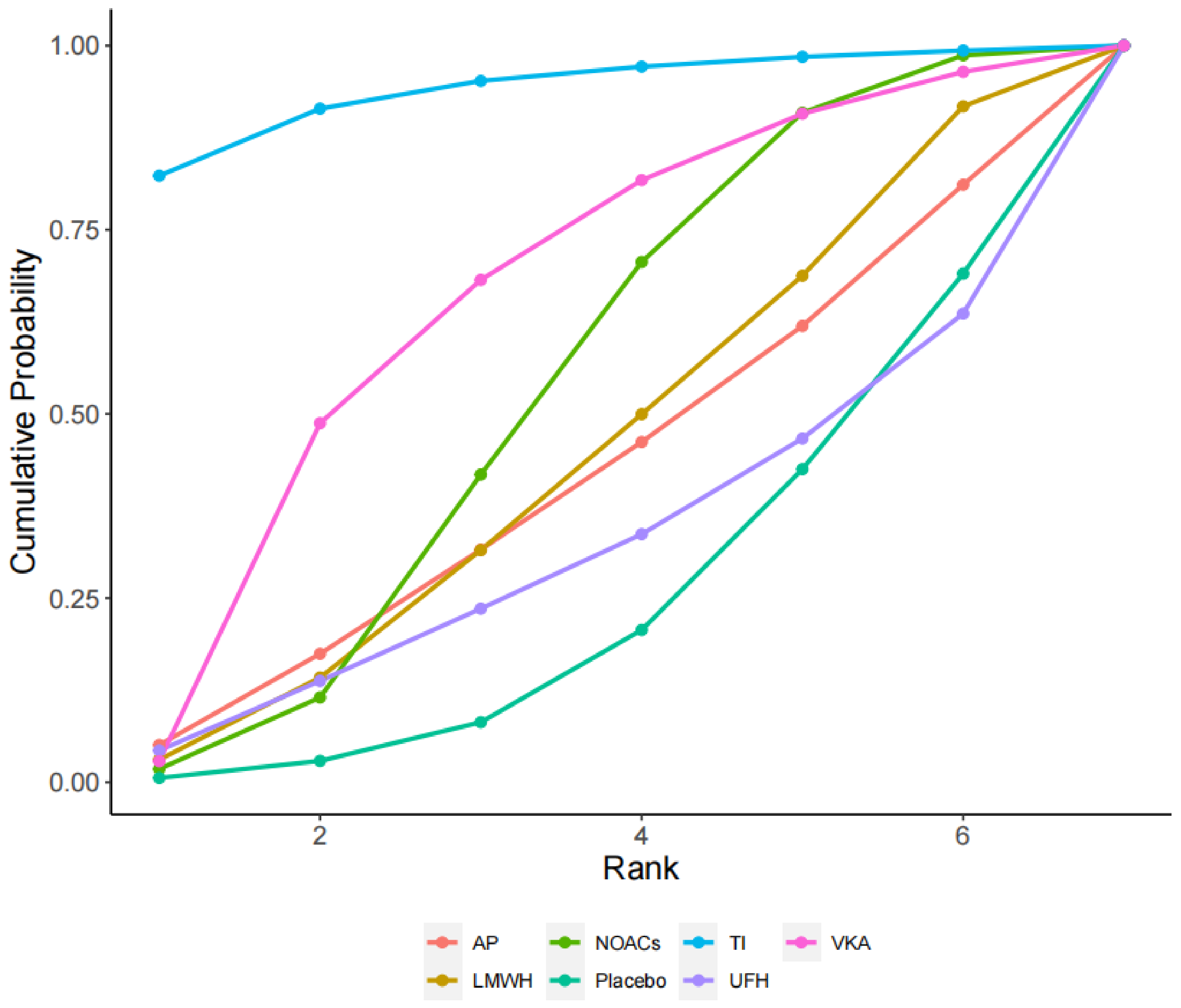

**Outcome：Fatal bleeding during treatment**

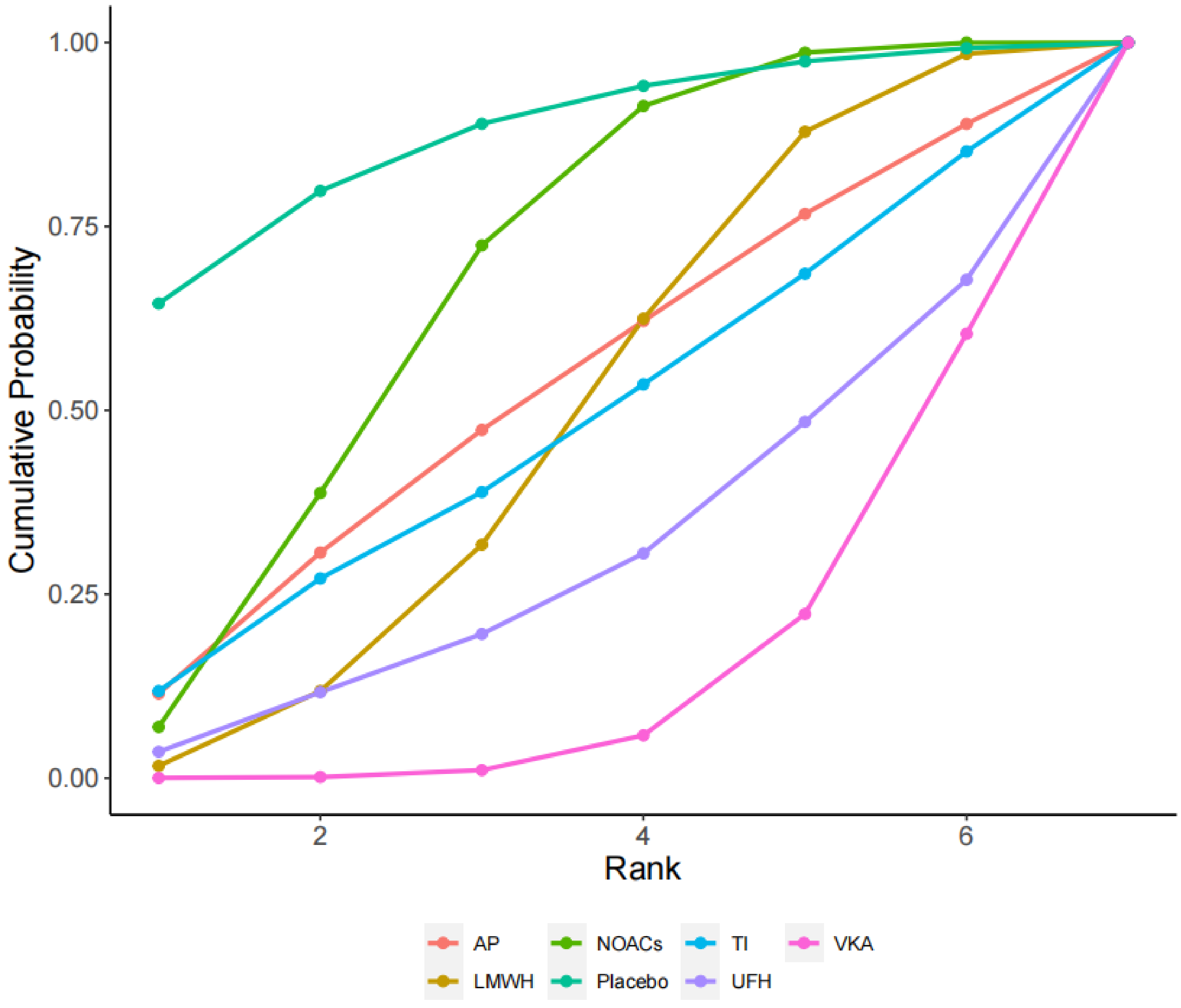


**Outcome：Adverse events during treatment**

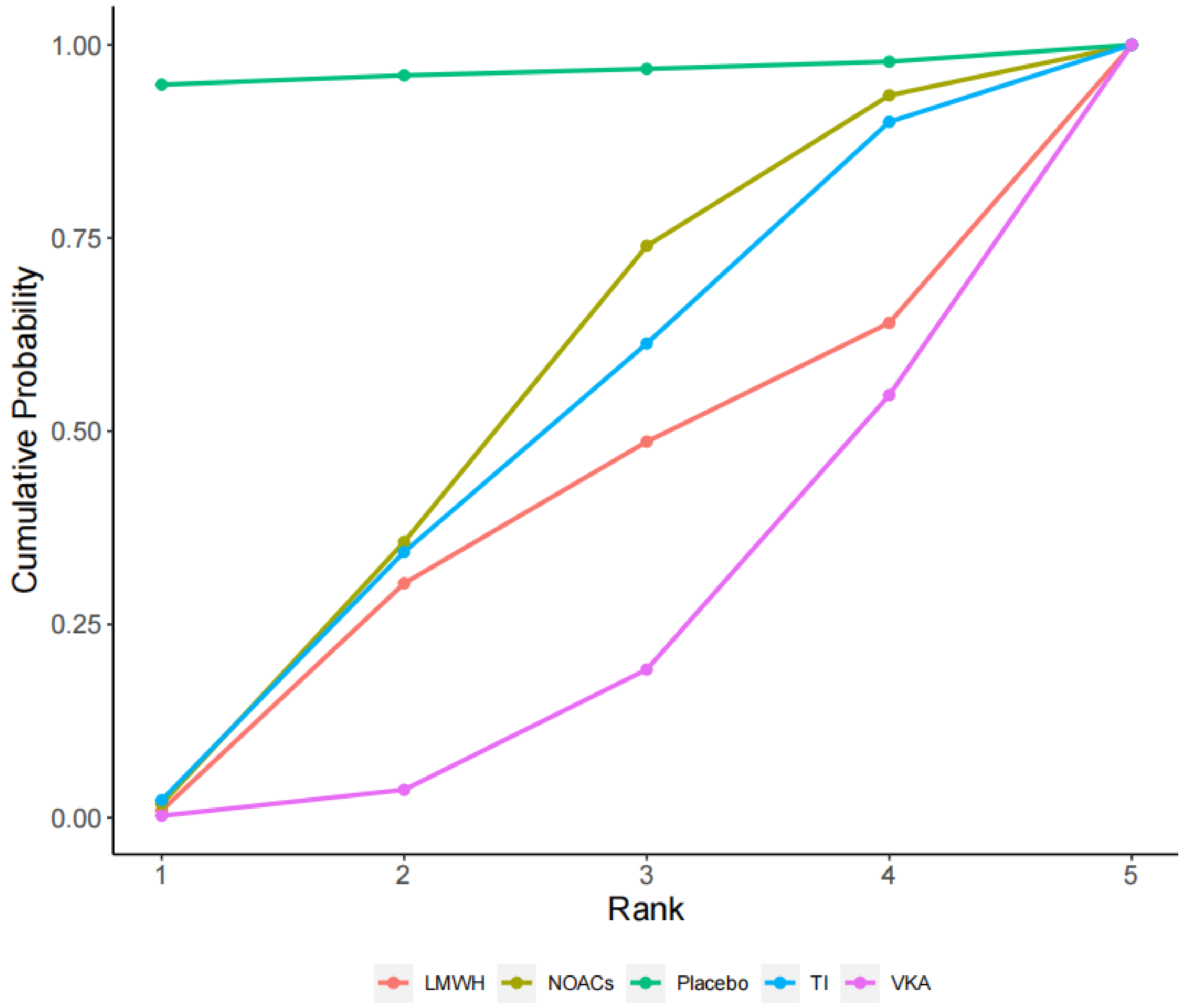

**Outcome: All-cause mortality during treatment**

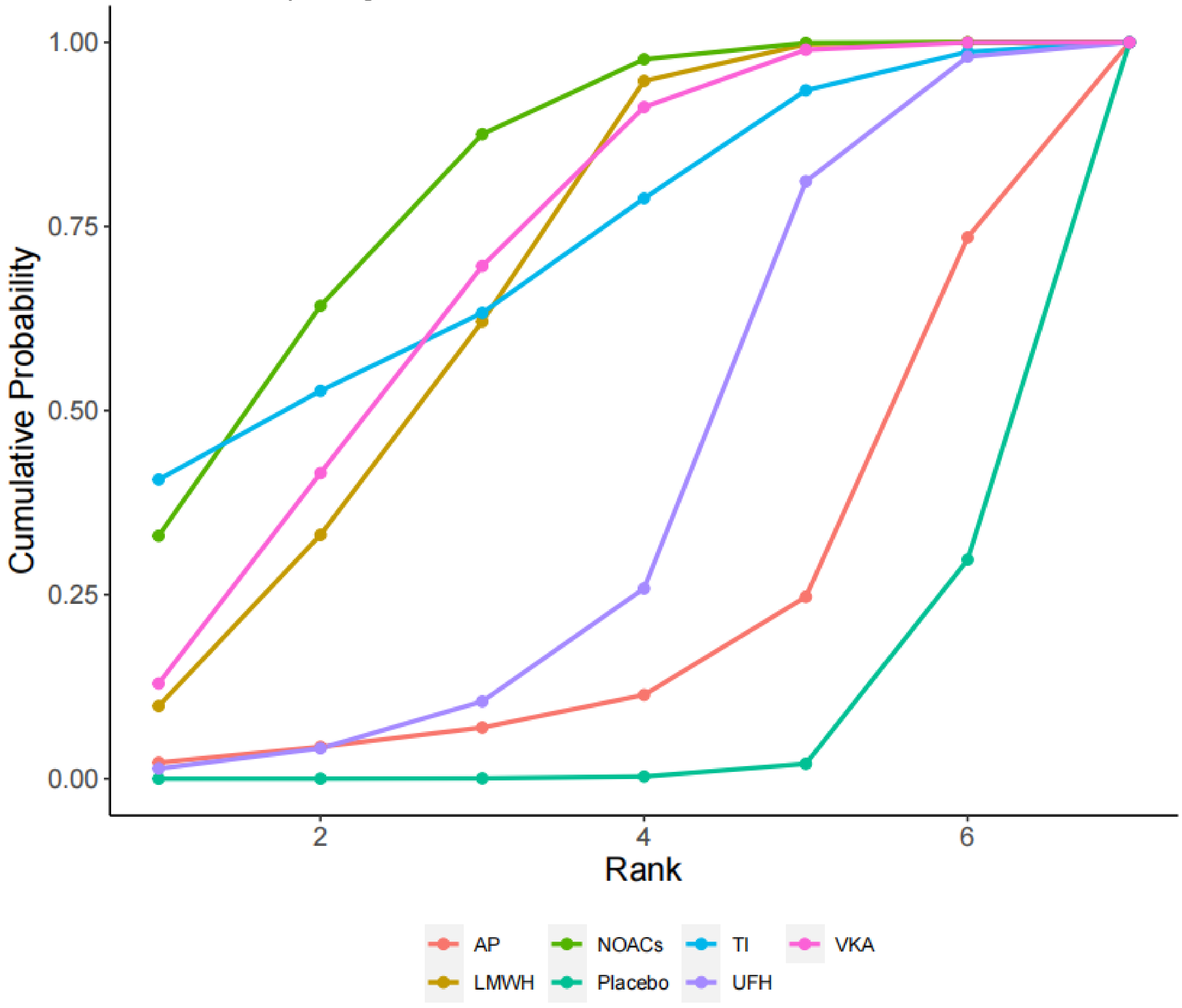


**Outcome: VTE during prophylaxis**

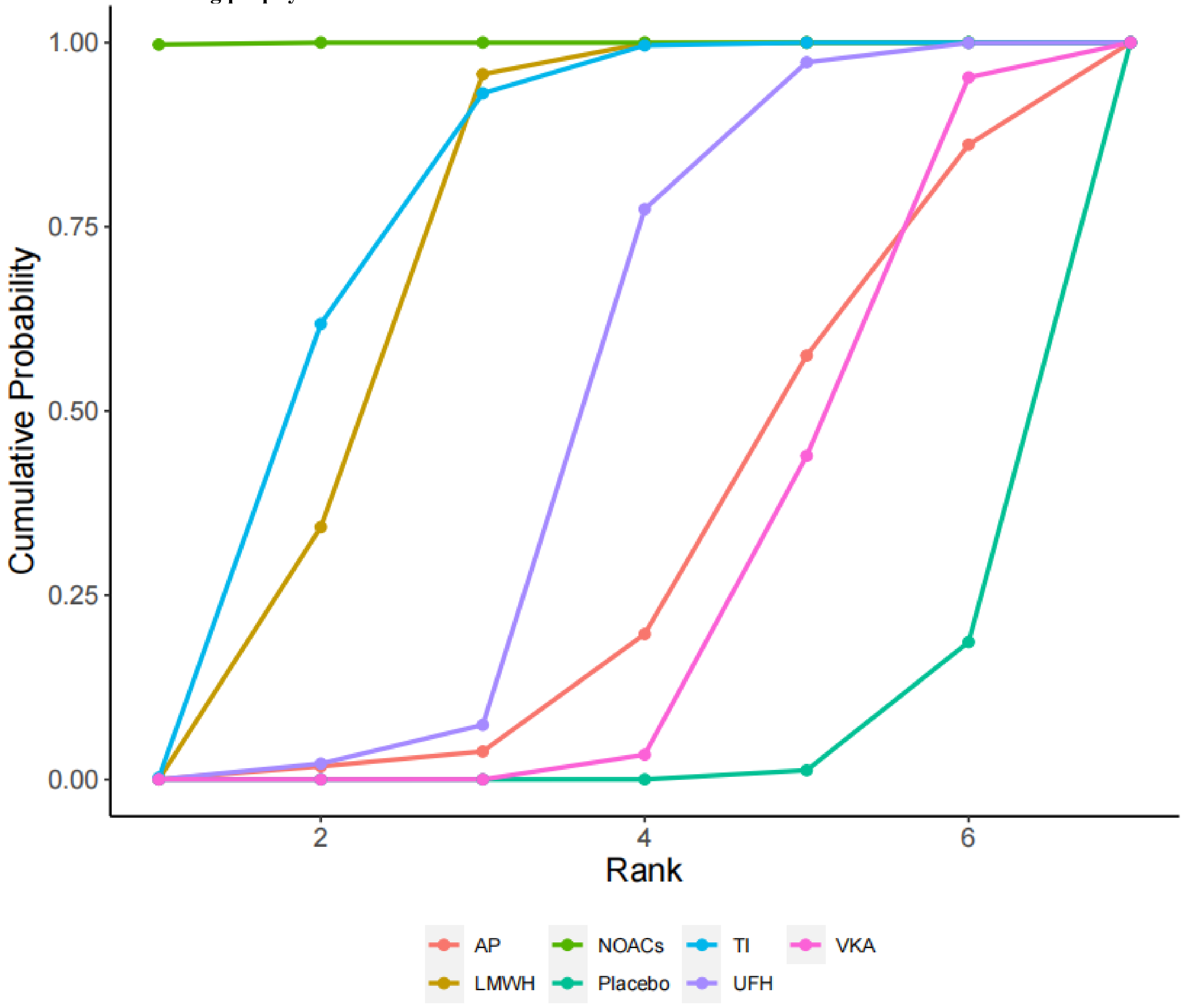

**Outcome: Major bleeding during prophylaxis**

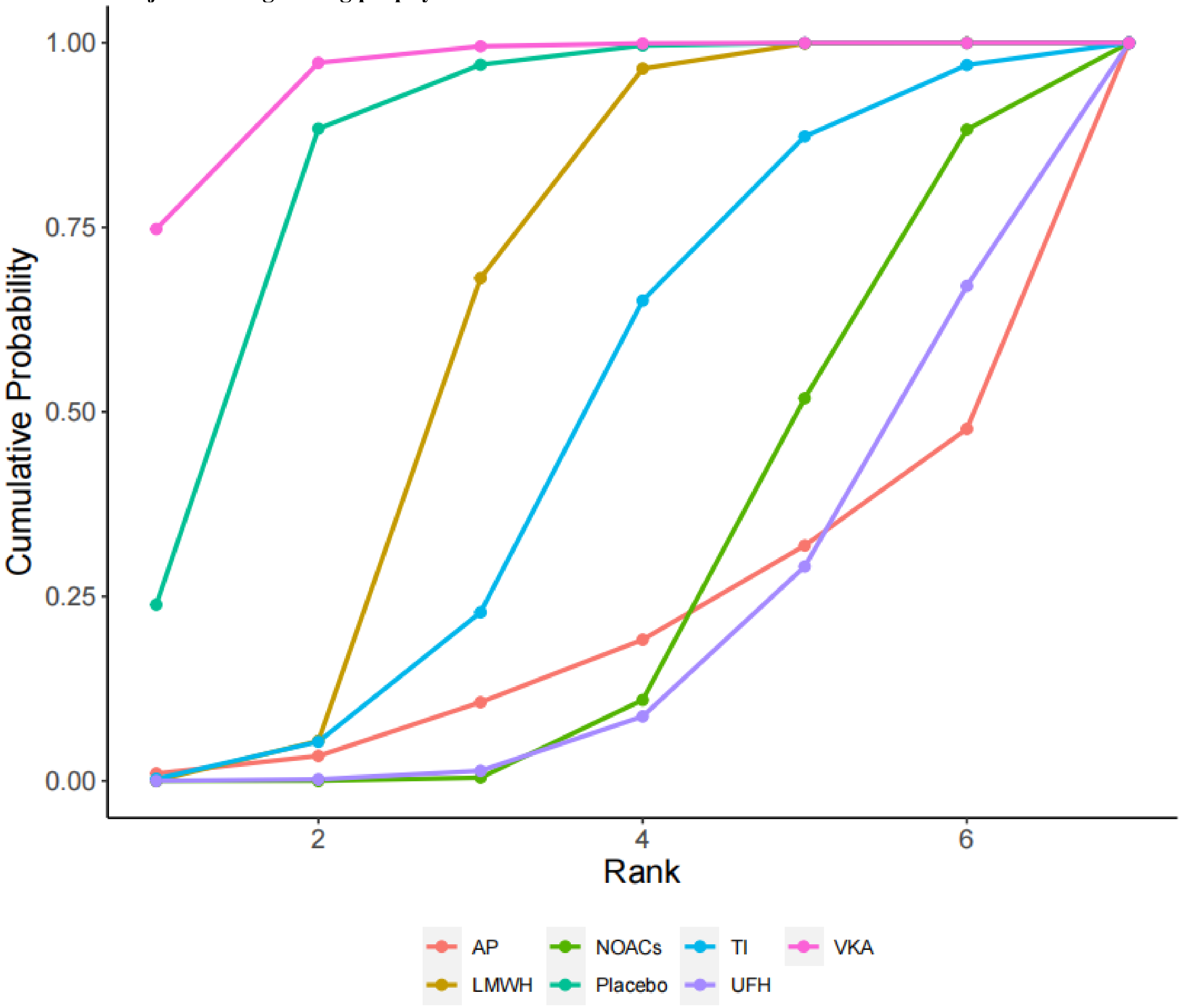


**Outcome: All-cause mortality during prophylaxis**

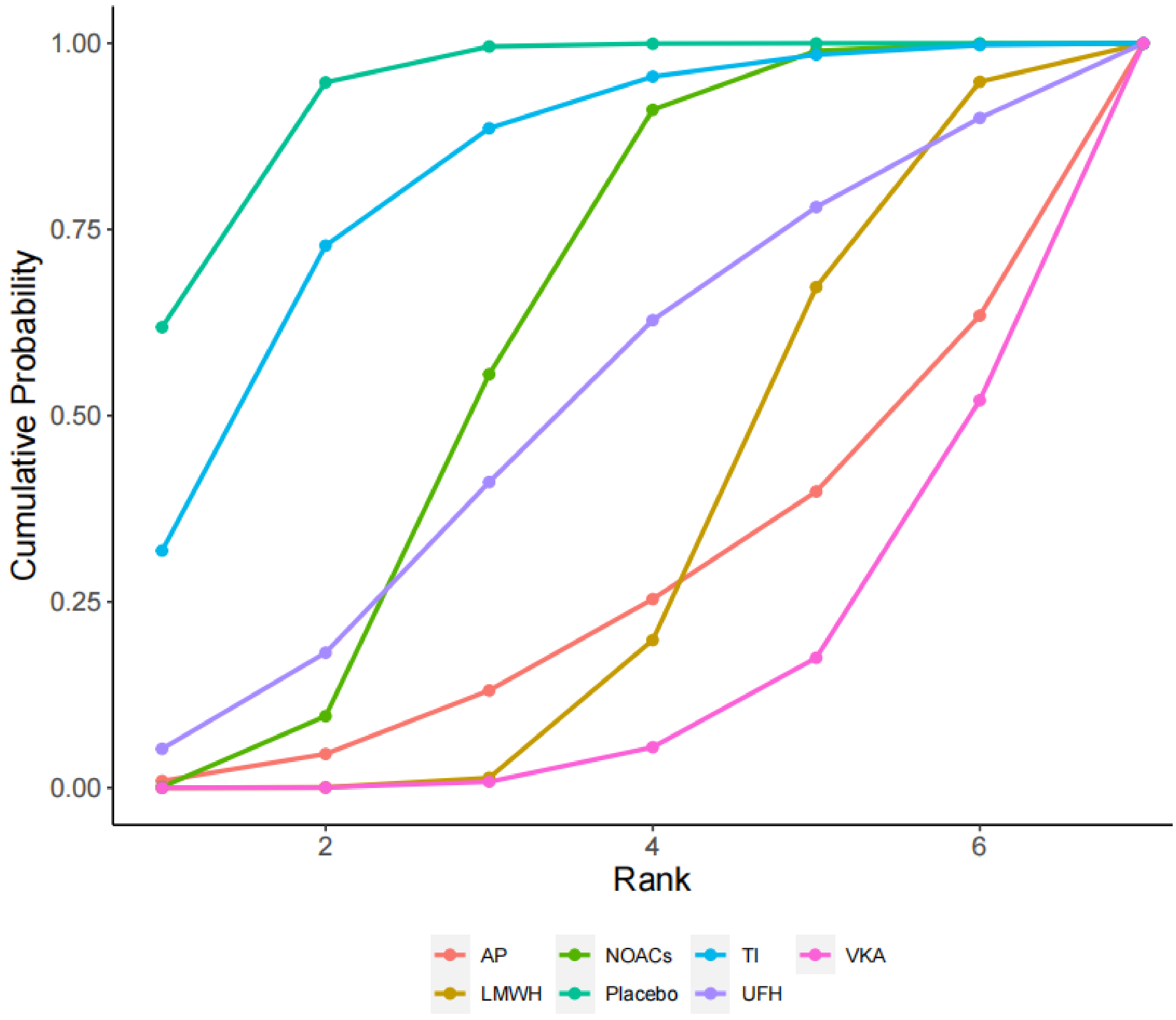

Outcome: Clinical relevant non-major bleeding during prophylaxis

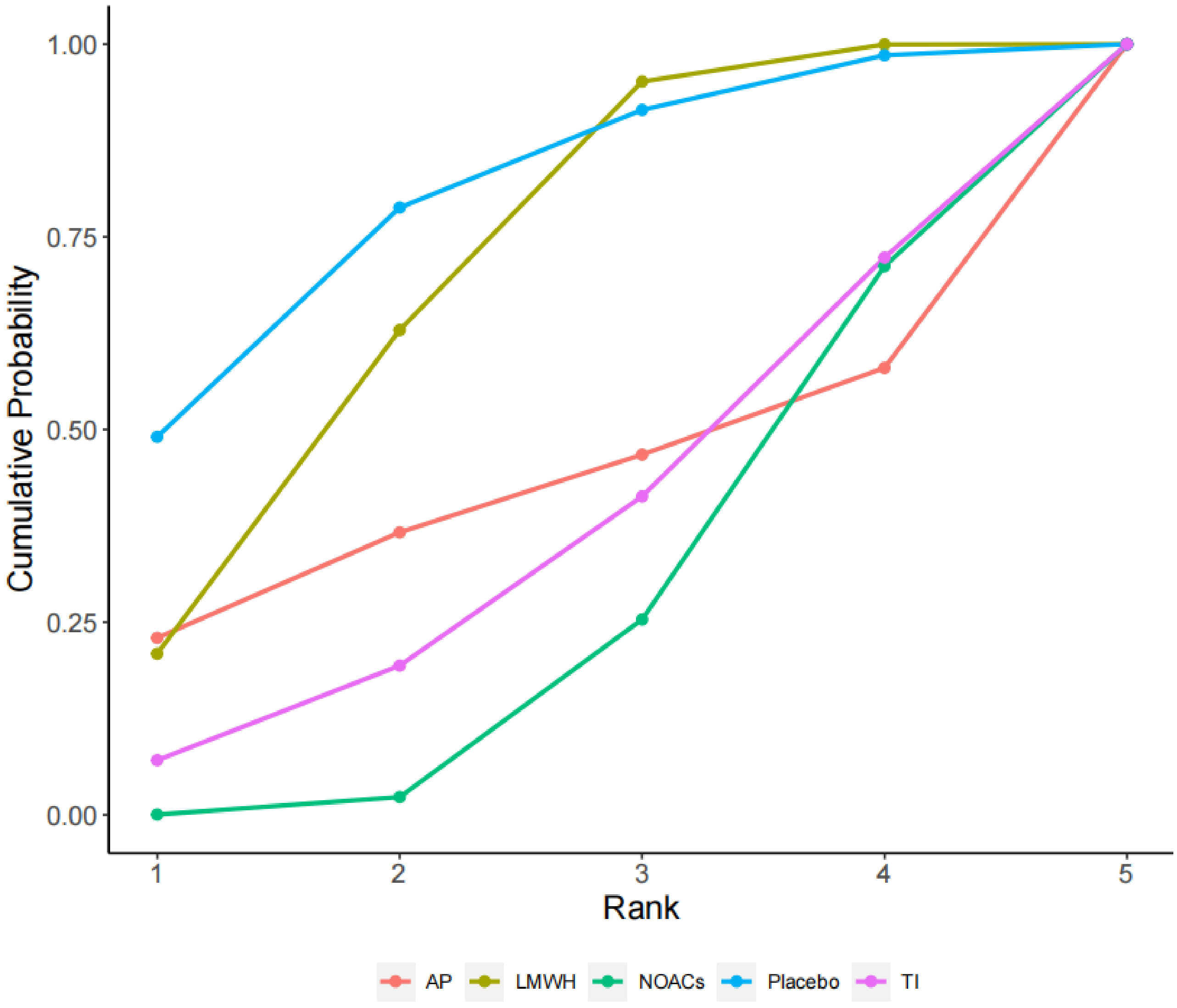


Outcome: VTE related death during prophylaxis

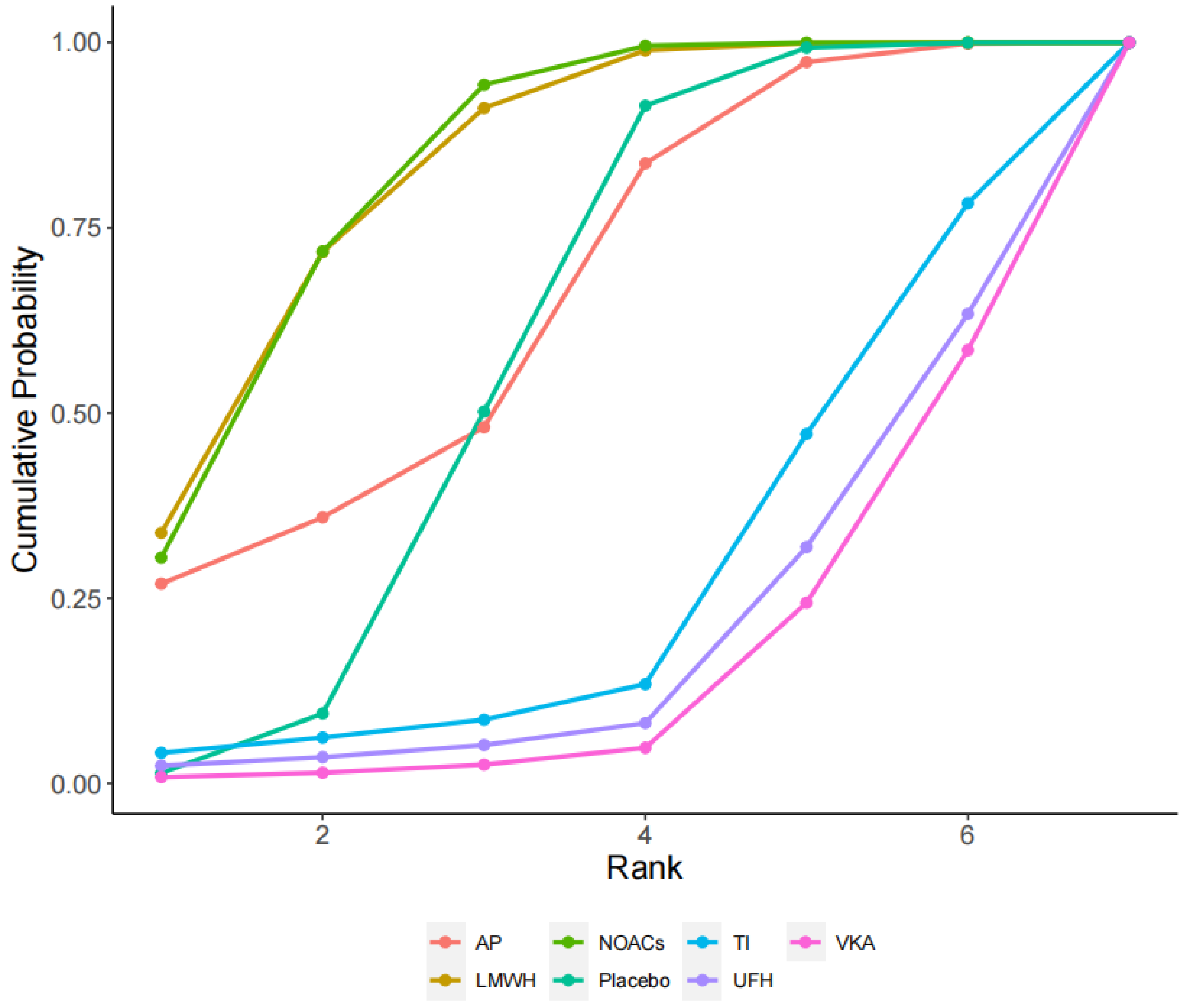

## Appendix 6 Reference

### 6.1 Treatment

[1] RASKOB G, AGENO W, COHEN A T, et al. Extended duration of anticoagulation with edoxaban in patients with venous thromboembolism: a post-hoc analysis of the Hokusai-VTE study [J]. The lancet haematology, 2016, 3(5): e228-e36.
[2] SCHULMAN S, KEARON C, KAKKAR A K, et al. Dabigatran versus Warfarin in the Treatment of Acute Venous Thromboembolism [J]. New England Journal of Medicine, 2009, 361(24): 2342-52.
[3] RASKOB G E, VAN ES N, VERHAMME P, et al. Edoxaban for the Treatment of Cancer-Associated Venous Thromboembolism [J]. New England Journal of Medicine, 2018, 378(7): 615-24.
[4] AGNELLI G, BULLER H R, COHEN A, et al. Apixaban for Extended Treatment of Venous Thromboembolism [J]. New England Journal of Medicine, 2013, 368(8): 699-708.
[5] KOOPMAN M M, PRANDONI P, PIOVELLA F, et al. Treatment of venous thrombosis with intravenous unfractionated heparin administered in the hospital as compared with subcutaneous low-molecular-weight heparin administered at home. The Tasman Study Group [J]. The New England journal of medicine, 1996, 334(11): 682-7.
[6] MEDINA A, RASKOB G, AGENO W, et al. Outpatient Management in Patients with Venous Thromboembolism with Edoxaban: A Post Hoc Analysis of the Hokusai-VTE Study [J]. Thrombosis and Haemostasis, 2017, 117(12): 2406-14.
[7] BULLER H R, COHEN A T, DAVIDSON B, et al. Extended prophylaxis of venous thromboembolism with idraparinux [J]. New England Journal of Medicine, 2007, 357(11): 1105-12.
[8] PLANQUETTE B, BERTOLETTI L, CHARLES-NELSON A, et al. Rivaroxaban vs Dalteparin in Cancer-Associated Thromboembolism: a Randomized Trial [J]. Chest, 2022, 161(3): 781-90.
[9] RIGHINI M, GALANAUD J-P, GUENNEGUEZ H, et al. Anticoagulant therapy for symptomatic calf deep vein thrombosis (CACTUS): a randomised, double-blind, placebo-controlled trial [J]. Lancet Haematology, 2016, 3(12): E556-E62.
[10] HULL R, DELMORE T, GENTON E, et al. Warfarin sodium versus low-dose heparin in the long-term treatment of venous thrombosis [J]. The New England journal of medicine, 1979, 301(16): 855-8.
[11] FINDIK S, ERKAN M L, SELCUK M B, et al. Low-molecular-weight heparin versus unfractionated heparin in the treatment of patients with acute pulmonary thromboembolism [J]. Respiration, 2002, 69(5): 440-4.
[12] GIROLAMI A, PRANDONI P, IACOBELLI M, et al. Subcutaneous adjusted-dose unfractionated heparin vs fixed-dose low-molecular-weight heparin in the initial treatment of venous thromboembolism [J]. Archives of Internal Medicine, 2004, 164(10): 1077-83.
[13] BELCARO G, NICOLAIDES A N, CESARONE M R, et al. Comparison of low-molecular-weight heparin, administered primarily at home, with unfractionated heparin, administered in hospital, and subcutaneous heparin, administered at home for deep-vein thrombosis [J]. Angiology, 1999, 50(10): 781-7.
[14] LEVINE M, GENT M, HIRSH J, et al. A comparison of low-molecular-weight heparin administered primarily at home with unfractionated heparin administered in the hospital for proximal deep-vein thrombosis [J]. The New England journal of medicine, 1996, 334(11): 677-81.
[15] ROMERA A, CAIROLS M A, VILA-COLL R, et al. A Randomised Open-Label Trial Comparing Long-term Sub-Cutaneous Low-Molecular-weight Heparin Compared with Oral-Anticoagulant Therapy in the Treatment of Deep Venous Thrombosis [J]. European Journal of Vascular and Endovascular Surgery, 2009, 37(3): 349-56.
[16] RIDKER P M, GOLDHABER S Z, DANIELSON E, et al. Long-term, low-intensity warfarin therapy for the prevention of recurrent venous thromboembolism [J]. The New England journal of medicine, 2003, 348(15): 1425-34.
[17] KEARON C, GINSBERG J S, JULIAN J A, et al. Comparison of fixed-dose weight-adjusted unfractionated heparin and low-molecular-weight heparin for acute treatment of venous thromboembolism [J]. Jama-Journal of the American Medical Association, 2006, 296(8): 935-42.
[18] MONREAL M, RONCALES F J, RUIZ J, et al. Secondary prevention of venous thromboembolism: A role for low-molecular-weight heparin [J]. Haemostasis, 1998, 28(5): 236-43.
[19] RIESS H, KOPPENHAGEN K, TOLLE A, et al. Fixed-dose, body weight-independent subcutaneous low molecular weight heparin Certoparin compared with adjusted-dose intravenous unfractionated heparin in patients with proximal deep venous thrombosis [J]. Thrombosis and Haemostasis, 2003, 90(2): 252-9.
[20] SIMONNEAU G, SORS H, CHARBONNIER B, et al. A comparison of low-molecular-weight heparin with unfractionated heparin for acute pulmonary embolism. The THESEE Study Group. Tinzaparine ou Heparine Standard: Evaluations dans l'Embolie Pulmonaire [J]. The New England journal of medicine, 1997, 337(10): 663-9.
[21] KANG J M, PARK K-H, AHN S, et al. Rivaroxaban after Thrombolysis in Acute Iliofemoral Venous Thrombosis: A Randomized, Open-labeled, Multicenter Trial [J]. Scientific Reports, 2019, 9.
[22] RAMACCIOTTI E, ARAUJO G R, LASTORIA S, et al. An open-label, comparative study of the efficacy and safety of once-daily dose of enoxaparin versus unfractionated heparin in the treatment of proximal lower limb deep-vein thrombosis [J]. Thrombosis Research, 2004, 114(3): 149-53.
[23] BYNUM L J, WILSON J E, 3RD. Low-dose heparin therapy in the long-term management of venous thromboembolism [J]. The American journal of medicine, 1979, 67(4): 553-6.
[24] MARSHALL A, LEVINE M, HILL C, et al. Treatment of cancer-associated venous thromboembolism: 12-month outcomes of the placebo versus rivaroxaban randomization of the SELECT-D Trial (SELECT-D: 12m) [J]. Journal of thrombosis and haemostasis : JTH, 2020, 18(4): 905-15.
[25] MERLI G, SPIRO T E, OLSSON C G, et al. Subcutaneous enoxaparin once or twice daily compared with intravenous unfractionated heparin for treatment of venous thromboembolic disease [J]. Annals of internal medicine, 2001, 134(3): 191-202.
[26] GONZALEZ-FAJARDO J A, ARREBA E, CASTRODEZA J, et al. Venographic comparison of subcutaneous low-molecular weight heparin with oral anticoagulant therapy in the long-term treatment of deep venous thrombosis [J]. Journal of vascular surgery, 1999, 30(2): 283-92.
[27] WELLS P S, ANDERSON D R, RODGER M A, et al. A Randomized trial comparing 2 low-molecular-weight heparins for the outpatient treatment of deep vein thrombosis and pulmonary embolism [J]. Archives of Internal Medicine, 2005, 165(7): 733-8.
[28] RASKOB G E, VAN ES N, SEGERS A, et al. Edoxaban for venous thromboembolism in patients with cancer: results from a non-inferiority subgroup analysis of the Hokusai-VTE randomised, double-blind, double-dummy trial [J]. Lancet Haematol, 2016, 3(8): e379-87.
[29] BEYER-WESTENDORF J, SCHELLONG S M, GERLACH H, et al. Prevention of thromboembolic complications in patients with superficial-vein thrombosis given rivaroxaban or fondaparinux: the open-label, randomised, non-inferiority SURPRISE phase 3b trial [J]. The Lancet Haematology, 2017, 4(3): e105-e13.
[30] LOPACIUK S, BIELSKA-FALDA H, NOSZCZYK W, et al. Low molecular weight heparin versus acenocoumarol in the secondary prophylaxis of deep vein thrombosis [J]. Thrombosis and haemostasis, 1999, 81(1): 26-31.
[31] SIMES J, BECATTINI C, AGNELLI G, et al. Aspirin for the Prevention of Recurrent Venous Thromboembolism The INSPIRE Collaboration [J]. Circulation, 2014, 130(13): 1062-+.
[32] MEYER G, MARJANOVIC Z, VALCKE J, et al. Comparison of low-molecular-weight heparin and warfarin for the secondary prevention of venous thromboembolism in patients with cancer: a randomized controlled study [J]. Archives of internal medicine, 2002, 162(15): 1729-35.
[33] SCHRAG D, UNO H, ROSOVSKY R, et al. Direct Oral Anticoagulants vs Low-Molecular-Weight Heparin and Recurrent VTE in Patients With Cancer A Randomized Clinical Trial [J]. Jama-Journal of the American Medical Association, 2023.
[34] AGNELLI G, BECATTINI C, MEYER G, et al. Apixaban for the Treatment of Venous Thromboembolism Associated with Cancer [J]. New England Journal of Medicine, 2020, 382(17): 1599-607.
[35] SCHULMAN S, KAKKAR A K, GOLDHABER S Z, et al. Treatment of acute venous thromboembolism with dabigatran or warfarin and pooled analysis [J]. Circulation, 2014, 129(7): 764-72.
[36] DI NISIO M, VAN ES N, CARRIER M, et al. Extended treatment with edoxaban in cancer patients with venous thromboembolism: A post-hoc analysis of the Hokusai-VTE Cancer study [J]. Journal of thrombosis and haemostasis : JTH, 2019, 17(11): 1866-74.
[37] BECATTINI C, AGNELLI G, POGGIO R, et al. Aspirin after oral anticoagulants for prevention of recurrence in patients with unprovoked venous thromboembolism. the warfasa study [J]. Blood, 2011, 118(21).
[38] BREDDIN H K, HACH-WUNDERLE V, NAKOV R, et al. Effects of a low-molecular-weight heparin on thrombus regression and recurrent thromboembolism in patients with deep-vein thrombosis [J]. The New England journal of medicine, 2001, 344(9): 626-31.
[39] BULLER H R, GENT M, GALLUS A S, et al. Low-molecular-weight heparin in the treatment of patients with venous thromboembolism [J]. The New England journal of medicine, 1997, 337(10): 657-62.
[40] BAUERSACHS R, LEE A Y Y, KAMPHUISEN P W, et al. Renal Impairment, Recurrent Venous Thromboembolism and Bleeding in Cancer Patients with Acute Venous ThromboembolismAnalysis of the CATCH Study [J]. Thrombosis and Haemostasis, 2018, 118(5): 914-21.
[41] PRINS M H, LENSING A W A, PRANDONI P, et al. Risk of recurrent venous thromboembolism according to baseline risk factor profiles [J]. Blood advances, 2018, 2(7): 788-96.
[42] JIMENEZ CASTRO D, DIAZ G, MARTI D, et al. Monotherapy with enoxaparin for the prevention of recurrent venous thromboembolism [J]. Blood Coagulation & Fibrinolysis, 2007, 18(2): 173-7.
[43] ERIKSSON H, LUNDSTRöM T, WåHLANDER K, et al. Prognostic factors for recurrence of venous thromboembolism (VTE) or bleeding during long-term secondary prevention of VTE with ximelagatran [J]. Thrombosis and haemostasis, 2005, 94(3): 522-7.
[44] VEIGA F, ESCRIBá A, MALUENDA M P, et al. Low molecular weight heparin (enoxaparin) versus oral anticoagulant therapy (acenocoumarol) in the

long-term treatment of deep venous thrombosis in the elderly: a randomized trial [J]. Thrombosis and haemostasis, 2000, 84(4): 559-64.
[45] NAKAMURA M, WANG Y Q, WANG C, et al. Efficacy and safety of edoxaban for treatment of venous thromboembolism: a subanalysis of East Asian patients in the Hokusai-VTE trial [J]. Journal of thrombosis and haemostasis : JTH, 2015, 13(9): 1606-14.
[46] CIRUJEDA J L, GRANADO P C. A study on the safety, efficacy, and efficiency of sulodexide compared with acenocoumarol in secondary prophylaxis in patients with deep venous thrombosis [J]. Angiology, 2006, 57(1): 53-64.
[47] PéREZ-DE-LLANO L A, LEIRO-FERNáNDEZ V, GOLPE R, et al. Comparison of tinzaparin and acenocoumarol for the secondary prevention of venous thromboembolism: a multicentre, randomized study [J]. Blood coagulation & fibrinolysis : an international journal in haemostasis and thrombosis, 2010, 21(8): 744-9.
[48] WELLS P S, PRINS M H, LEVITAN B, et al. Long-term Anticoagulation With Rivaroxaban for Preventing Recurrent VTE: A Benefit-Risk Analysis of EINSTEIN-Extension [J]. Chest, 2016, 150(5): 1059-68.
[49] WEITZ J I, LENSING A W A, PRINS M H, et al. Rivaroxaban or Aspirin for Extended Treatment of Venous Thromboembolism [J]. New England Journal of Medicine, 2017, 376(13): 1211-22.
[50] BULLER H R, DESTORS J-M, GALLUS A S, et al. Efficacy and safety of once weekly subcutaneous idrabiotaparinux in the treatment of patients with symptomatic deep venous thrombosis [J]. Journal of Thrombosis and Haemostasis, 2011, 9(1): 92-9.
[51] LEE A Y, LEVINE M N, BAKER R I, et al. Low-molecular-weight heparin versus a coumarin for the prevention of recurrent venous thromboembolism in patients with cancer [J]. The New England journal of medicine, 2003, 349(2): 146-53.
[52] HARENBERG J, SCHMIDT J A, KOPPENHAGEN K, et al. Fixed-dose, body weight-independent subcutaneous LMW heparin versus adjusted dose unfractionated intravenous heparin in the initial treatment of proximal venous thrombosis [J]. Thrombosis and haemostasis, 2000, 83(5): 652-6.
[53] SCHULMAN S, KEARON C, KAKKAR A K, et al. Extended Use of Dabigatran, Warfarin, or Placebo in Venous Thromboembolism [J]. New England Journal of Medicine, 2013, 368(8): 709-18.
[54] HARENBERG J, HUISMAN M V, TOLLE A R, et al. Reduction in thrombus extension and clinical end points in patients after initial treatment for deep vein thrombosis with the fixed-dose body weight-independent low molecular weight heparin certoparin [J]. Seminars in thrombosis and hemostasis, 2001, 27(5): 513-8.
[55] WEYCKER D, LI X, WYGANT G D, et al. Effectiveness and Safety of Apixaban versus Warfarin as Outpatient Treatment of Venous Thromboembolism in US Clinical Practice [J]. Thrombosis and Haemostasis, 2018, 118(11): 1951-61.
[56] WYSOKINSKI W E, HOUGHTON D E, CASANEGRA A I, et al. Comparison of apixaban to rivaroxaban and enoxaparin in acute cancer-associated venous thromboembolism [J]. American Journal of Hematology, 2019, 94(11): 1185-92.
[57] HARENBERG J, RIESS H, BULLER H R, et al. Comparison of six-month outcome of patients initially treated for acute deep vein thrombosis with a low molecular weight heparin Certoparin at a fixed, body-weight-independent dosage or unfractionated heparin [J]. Haematologica, 2003, 88(10): 1157-62.
[58] YOUNG A M, MARSHALL A, THIRLWALL J, et al. Comparison of an Oral Factor Xa Inhibitor With Low Molecular Weight Heparin in Patients With Cancer With Venous Thromboembolism: Results of a Randomized Trial (SELECT-D) [J]. Journal of clinical oncology : official journal of the American Society of Clinical Oncology, 2018, 36(20): 2017-23.

## 6.2 Prevention

[1] ABBAS M S. Bemiparin versus Enoxaparin in the Prevention of Venous Thromboembolism among Intensive Care Unit Patients [J]. Indian J Crit Care Med, 2017, 21(7): 419-23.
[2] WANG T Y, WAHED A S, MORRIS A, et al. Effect of Thromboprophylaxis on Clinical Outcomes After COVID-19 Hospitalization [J]. Annals of internal medicine, 2023, 176(4): 515-23.
[3] VADHAN-RAJ S, MCNAMARA M G, VENERITO M, et al. Rivaroxaban thromboprophylaxis in ambulatory patients with pancreatic cancer: Results from a pre-specified subgroup analysis of the randomized CASSINI study [J]. Cancer medicine, 2020, 9(17): 6196-204.
[4] TURPIE A G, LASSEN M R, DAVIDSON B L, et al. Rivaroxaban versus enoxaparin for thromboprophylaxis after total knee arthroplasty (RECORD4): a randomised trial [J]. Lancet (london, england), 2009, 373(9676): 1673-80.
[5] ERIKSSON B I, TURPIE A G G, LASSEN M R, et al. Prevention of venous thromboembolism with an oral factor Xa inhibitor, YM150, after total hip arthroplasty. A dose finding study (ONYX-2) [J]. Journal of Thrombosis and Haemostasis, 2010, 8(4): 714-21.
[6] FISHER W D, ERIKSSON B I, BAUER K A, et al. Rivaroxaban for thromboprophylaxis after orthopaedic surgery: pooled analysis of two studies [J]. Thrombosis and haemostasis, 2007, 97(6): 931-7.
[7] KOLLURI R, PLESSA A L, SANDERS M C, et al. A randomized study of the safety and efficacy of fondaparinux versus placebo in the prevention of venous thromboembolism after coronary artery bypass graft surgery [J]. American heart journal, 2016, 171(1): 1-6.
[8] BERGQVIST D, BENONI G, BJORGELL O, et al. Low-molecular-weight heparin (enoxaparin) as prophylaxis against venous thromboembolism after total hip replacement [J]. The New England journal of medicine, 1996, 335(10): 696-700.
[9] KAKKAR A K, LEVINE M N, KADZIOLA Z, et al. Low molecular weight heparin, therapy with dalteparin, and survival in advanced cancer: The fragmin advanced malignancy outcome study (FAMOUS) [J]. Journal of Clinical Oncology, 2004, 22(10): 1944-8.
[10] ERIKSSON B I, BORRIS L C, DAHL O E, et al. Dose-escalation study of rivaroxaban (BAY 59-7939) - an oral, direct Factor Xa inhibitor - for the prevention of venous thromboembolism in patients undergoing total hip replacement [J]. Thrombosis Research, 2007, 120(5): 685-93.
[11] FUJI T, OCHI T, NIWA S, et al. Prevention of postoperative venous thromboembolism in Japanese patients undergoing total hip or knee arthroplasty: two randomized, double-blind, placebo-controlled studies with three dosage regimens of enoxaparin [J]. Journal of orthopaedic science : official journal of the Japanese Orthopaedic Association, 2008, 13(5): 442-51.
[12] ERIKSSON B I. Improvements in the prevention of postoperative venous thromboembolism in hip fracture patients [J]. Orthopedics, 2003, 26(8 Suppl): s851-8.
[13] HAAS S K, FREUND M, HEIGENER D, et al. Low-Molecular-Weight Heparin Versus Placebo for the Prevention of Venous Thromboembolism in Metastatic Breast Cancer or Stage III/IV Lung Cancer [J]. Clinical and Applied Thrombosis-Hemostasis, 2012, 18(2): 159-65.
[14] VERHAMME P, YI B A, SEGERS A, et al. Abelacimab for Prevention of Venous Thromboembolism [J]. New England Journal of Medicine, 2021, 385(7): 609-17.
[15] SELBY R, GEERTS W H, KREDER H J, et al. A double-blind, randomized controlled trial of the prevention of clinically important venous thromboembolism after isolated lower leg fractures [J]. Journal of orthopaedic trauma, 2015, 29(5): 224-30.
[16] EIKELBOOM J W, KEARON C, GUYATT G, et al. Perioperative Aspirin for Prevention of Venous Thromboembolism: The PeriOperative ISchemia Evaluation-2 Trial and a Pooled Analysis of the Randomized Trials [J]. Anesthesiology, 2016, 125(6): 1121-9.
[17] ERIKSSON B I, DAHL O E, ROSENCHER N, et al. Oral dabigatran etexilate vs. subcutaneous enoxaparin for the prevention of venous thromboembolism after total knee replacement: the RE-MODEL randomized trial [J]. Journal of Thrombosis and Haemostasis, 2007, 5(11): 2178-85.
[18] WEITZ J I, STRONY J, AGENO W, et al. Milvexian for the Prevention of Venous Thromboembolism [J]. New England Journal of Medicine, 2021, 385(23): 2161-72.
[19] BECATTINI C, PACE U, PIROZZI F, et al. Rivaroxaban vs placebo for extended antithrombotic prophylaxis after laparoscopic surgery for colorectal cancer [J]. Blood, 2022, 140(8): 900-8.
[20] AGNELLI G, HAAS S, GINSBERG J S, et al. A phase II study of the oral factor Xa inhibitor LY517717 for the prevention of venous thromboembolism after hip or knee replacement [J]. Journal of thrombosis and haemostasis : JTH, 2007, 5(4): 746-53.
[21] CARRIER M, ABOU-NASSAR K, MALLICK R, et al. Apixaban to Prevent Venous Thromboembolism in Patients with Cancer [J]. The New England journal of medicine, 2019, 380(8): 711-9.
[22] PLANES A, SAMAMA M M, LENSING A W, et al. Prevention of deep vein thrombosis after hip replacement--comparison between two low-molecular heparins, tinzaparin and enoxaparin [J]. Thrombosis and haemostasis, 1999, 81(1): 22-5.
[23] WOLLER S C, BERTIN K C, STEVENS S M, et al. A Prospective Comparison of Warfarin to Aspirin for Thromboprophylaxis in Total Hip and Total Knee Arthroplasty [J]. Journal of Arthroplasty, 2012, 27(1): 1-9.
[24] FUJI T, WANG C J, FUJITA S, et al. Safety and efficacy of edoxaban, an oral factor xa inhibitor, for thromboprophylaxis after total hip arthroplasty in Japan and Taiwan [J]. The Journal of arthroplasty, 2014, 29(12): 2439-46.
[25] ERIKSSON B I, BORRIS L, DAHL O E, et al. Oral, direct Factor Xa inhibition with BAY 59-7939 for the prevention of venous thromboembolism after total hip replacement [J]. Journal of thrombosis and haemostasis : JTH, 2006, 4(1): 121-8.
[26] PERRY J R, JULIAN J A, LAPERRIERE N J, et al. PRODIGE: a randomized placebo-controlled trial of dalteparin low-molecular-weight heparin thromboprophylaxis in patients with newly diagnosed malignant glioma [J]. Journal of Thrombosis and Haemostasis, 2010, 8(9): 1959-65.
[27] COLWELL C W, BERKOWITZ S D, LIEBERMAN J R, et al. Oral direct thrombin inhibitor ximelagatran compared with warfarin for the prevention of venous thromboembolism after total knee arthroplasty [J]. Journal of Bone and Joint Surgery-American Volume, 2005, 87A(10): 2169-77.
[28] RAMACCIOTTI E, BARILE AGATI L, CALDERARO D, et al. Rivaroxaban versus no anticoagulation for post-discharge thromboprophylaxis after hospitalisation for COVID-19 (MICHELLE): an open-label, multicentre, randomised, controlled trial [J]. Lancet, 2022, 399(10319): 50-9.
[29] AGENO W, LOPES R D, GOLDIN M, et al. Rivaroxaban for extended thromboprophylaxis in acutely ill medical patients 75 years of age or older [J]. Journal of thrombosis and haemostasis : JTH, 2021, 19(11): 2772-80.
[30] LEVINE M N, GU C, LIEBMAN H A, et al. A randomized phase II trial of apixaban for the prevention of thromboembolism in patients with metastatic cancer [J]. Journal of thrombosis and haemostasis : JTH, 2012, 10(5): 807-14.

[31] DI CARLO V, AGNELLI G, PRANDONI P, et al. Dermatan sulphate for the prevention of postoperative venous thromboembolism in patients with cancer. DOS (Dermatan sulphate in Oncologic Surgery) Study Group [J]. Thrombosis and haemostasis, 1999, 82(1): 30-4.
[32] ERIKSSON B I, DAHL O E, HUO M H, et al. Oral dabigatran versus enoxaparin for thromboprophylaxis after primary total hip arthroplasty (RE-NOVATE II) A randomised, double-blind, non-inferiority trial [J]. Thrombosis and Haemostasis, 2011, 105(4): 721-9.
[33] FRANCIS C W, BERKOWITZ S D, COMP P C, et al. Comparison of ximelagatran with warfarin for the prevention of venous thromboembolism after total knee replacement [J]. The New England journal of medicine, 2003, 349(18): 1703-12.
[34] SAMAMA C M, LAPORTE S, ROSENCHER N, et al. Rivaroxaban or Enoxaparin in Nonmajor Orthopedic Surgery [J]. New England Journal of Medicine, 2020, 382(20): 1916-25.
[35] HEIT J A, BERKOWITZ S D, BONA R, et al. Efficacy and safety of low molecular weight heparin (ardeparin sodium) compared to warfarin for the prevention of venous thromboembolism after total knee replacement surgery: a double-blind, dose-ranging study. Ardeparin Arthroplasty Study Group [J]. Thrombosis and haemostasis, 1997, 77(1): 32-8.
[36] ERIKSSON B I, AGNELLI G, COHEN A T, et al. The direct thrombin inhibitor melagatran followed by oral ximelagatran compared with enoxaparin for the prevention of venous thromboembolism after total hip or knee replacement: the EXPRESS study [J]. Journal of thrombosis and haemostasis : JTH, 2003, 1(12): 2490-6.
[37] COLWELL C W, JR., SPIRO T E, TROWBRIDGE A A, et al. Efficacy and safety of enoxaparin versus unfractionated heparin for prevention of deep venous thrombosis after elective knee arthroplasty. Enoxaparin Clinical Trial Group [J]. Clinical orthopaedics and related research, 1995, (321): 19-27.
[38] LASSEN M R, FISHER W, MOURET P, et al. Semuloparin for prevention of venous thromboembolism after major orthopedic surgery: results from three randomized clinical trials, SAVE-HIP1, SAVE-HIP2 and SAVE-KNEE [J]. Journal of Thrombosis and Haemostasis, 2012, 10(5): 822-32.
[39] LASSEN M R, GALLUS A, RASKOB G E, et al. Apixaban versus Enoxaparin for Thromboprophylaxis after Hip Replacement [J]. New England Journal of Medicine, 2010, 363(26): 2487-98.
[40] SAMAMA C M, VRAY M, BARRé J, et al. Extended venous thromboembolism prophylaxis after total hip replacement: a comparison of low-molecular-weight heparin with oral anticoagulant [J]. Archives of internal medicine, 2002, 162(19): 2191-6.
[41] MENZIN J, RICHNER R, HUSE D, et al. Prevention of deep-vein thrombosis following total hip replacement surgery with enoxaparin versus unfractionated heparin: a pharmacoeconomic evaluation [J]. The Annals of pharmacotherapy, 1994, 28(2): 271-5.
[42] WIRTH T, SCHNEIDER B, MISSELWITZ F, et al. Prevention of venous thromboembolism after knee arthroscopy with low-molecular weight heparin (reviparin): Results of a randomized controlled trial [J]. Arthroscopy : the journal of arthroscopic & related surgery : official publication of the Arthroscopy Association of North America and the International Arthroscopy Association, 2001, 17(4): 393-9.
[43] BURROWS R F, GAN E T, GALLUS A S, et al. A randomised double-blind placebo controlled trial of low molecular weight heparin as prophylaxis in preventing venous thrombolic events after caesarean section: a pilot study [J]. BJOG : an international journal of obstetrics and gynaecology, 2001, 108(8): 835-9.
[44] LEIZOROVICZ A, COHEN A T, TURPIE A G, et al. Randomized, placebo-controlled trial of dalteparin for the prevention of venous thromboembolism in acutely ill medical patients [J]. Circulation, 2004, 110(7): 874-9.
[45] KLEBER F X, WITT C, VOGEL G, et al. Randomized comparison of enoxaparin with unfractionated heparin for the prevention of venous thromboembolism in medical patients with heart failure or severe respiratory disease [J]. American heart journal, 2003, 145(4): 614-21.
[46] HEIT J A, ELLIOTT C G, TROWBRIDGE A A, et al. Ardeparin sodium for extended out-of-hospital prophylaxis against venous thromboembolism after total hip or knee replacement. A randomized, double-blind, placebo-controlled trial [J]. Annals of internal medicine, 2000, 132(11): 853-61.
[47] CHO K Y, KIM K I, KHURANA S, et al. Is routine chemoprophylaxis necessary for prevention of venous thromboembolism following knee arthroplasty in a low incidence population? [J]. Archives of orthopaedic and trauma surgery, 2013, 133(4): 551-9.
[48] GINSBERG J S, DAVIDSON B L, COMP P C, et al. Oral Thrombin Inhibitor Dabigatran Etexilate vs North American Enoxaparin Regimen for Prevention of Venous Thromboembolism After Knee Arthroplasty Surgery [J]. Journal of Arthroplasty, 2009, 24(1): 1-9.
[49] SONG J, XUAN L, WU W, et al. Fondaparinux versus nadroparin for thromboprophylaxis following minimally invasive esophagectomy: A randomized controlled trial [J]. Thromb Res, 2018, 166: 22-7.
[50] FUJI T, FUJITA S, OCHI T. Fondaparinux prevents venous thromboembolism after joint replacement surgery in Japanese patients [J]. International orthopaedics, 2008, 32(4): 443-51.
[51] ERIKSSON B I, DAHL O E, ROSENCHER N, et al. Dabigatran etexilate versus enoxaparin for prevention of venous thromboembolism after total hip replacement: a randomised, double-blind, non-inferiority trial [J]. Lancet, 2007, 370(9591): 949-56.
[52] ERIKSSON B I, DAHL O E, BULLER H R, et al. A new oral direct thrombin inhibitor, dabigatran etexilate, compared with enoxaparin for prevention of thromboembolic events following total hip or knee replacement: the BISTRO II randomized trial [J]. Journal of Thrombosis and Haemostasis, 2005, 3(1): 103-11.
[53] SCHELLONG S M, HAAS S, GREINACHER A, et al. An open-label comparison of the efficacy and safety of certoparin versus unfractionated heparin for the prevention of thromboembolic complications in acutely ill medical patients: CERTAIN [J]. Expert opinion on pharmacotherapy, 2010, 11(18): 2953-61.
[54] ERIKSSON B I, DAHL O E, LASSEN M R, et al. Partial factor IXa inhibition with TTP889 for prevention of venous thromboembolism: an exploratory study [J]. Journal of thrombosis and haemostasis : JTH, 2008, 6(3): 457-63.
[55] ERIKSSON B I, LASSEN M R, COLWELL C W. Efficacy of fondaparinux for thromboprophylaxis in hip fracture patients [J]. Journal of Arthroplasty, 2004, 19(7): 78-81.
[56] WEITZ J I, SEGERS A, RASKOB G, et al. Randomized phase 2 trial comparing JNJ-9375, a thrombin-directed antibody, with apixaban for prevention of venous thrombosis [J]. Journal of thrombosis and haemostasis : JTH, 2019, 17(12): 2081-8.
[57] SPYROPOULOS A C, AGENO W, ALBERS G W, et al. Rivaroxaban for Thromboprophylaxis after Hospitalization for Medical Illness [J]. The New England journal of medicine, 2018, 379(12): 1118-27.
[58] AGNELLI G, GUSSONI G, BIANCHINI C, et al. Nadroparin for the prevention of thromboembolic events in ambulatory patients with metastatic or locally advanced solid cancer receiving chemotherapy: a randomised, placebo-controlled, double-blind study [J]. The Lancet Oncology, 2009, 10(10): 943-9.
[59] MARLOVITS S, STRIESSNIG G, SCHUSTER R, et al. Extended-duration thromboprophylaxis with enoxaparin after arthroscopic surgery of the anterior cruciate ligament: A prospective, randomized, placebo-controlled study [J]. Arthroscopy-the Journal of Arthroscopic and Related Surgery, 2007, 23(7): 696-702.
[60] DE OLIVEIRA A L M L, DE OLIVEIRA PEREIRA R F, AGATI L B, et al. Rivaroxaban Versus Enoxaparin for Thromboprophylaxis After major Gynecological Cancer Surgery: The VALERIA Trial Venous thromboembolism prophylAxis after gynecoLogical pElvic cancer surgery with RIvaroxaban versus enoxAparin (VALERIA trial) [J]. Clinical and Applied Thrombosis-Hemostasis, 2022, 28.
[61] LECHLER E, SCHRAMM W, FLOSBACH C W. The venous thrombotic risk in non-surgical patients: epidemiological data and efficacy/safety profile of a low-molecular-weight heparin (enoxaparin). The Prime Study Group [J]. Haemostasis, 1996, 26 Suppl 2: 49-56.
[62] BRUNTINK M M, GROUTARS Y M E, SCHIPPER I B, et al. Nadroparin or fondaparinux versus no thromboprophylaxis in patients immobilised in a below-knee plaster cast (PROTECT): A randomised controlled trial [J]. Injury-International Journal of the Care of the Injured, 2017, 48(4): 936-40.
[63] SAMAMA M M, COHEN A T, DARMON J Y, et al. A comparison of enoxaparin with placebo for the prevention of venous thromboembolism in acutely ill medical patients. Prophylaxis in Medical Patients with Enoxaparin Study Group [J]. The New England journal of medicine, 1999, 341(11): 793-800.
[64] AGNELLI G, ERIKSSON E L, COHEN A T, et al. Safety assessment of new antithrombotic agents: Lessons from the EXTEND study on ximelagatran [J]. Thrombosis Research, 2009, 123(3): 488-97.
[65] ZHAO M, BAO Y, JIANG C, et al. Rivaroxaban versus nadroparin for thromboprophylaxis following thoracic surgery for lung cancer: A randomized, noninferiority trial [J]. American journal of hematology, 2023, 98(8): 1185-95.
[66] KAKKAR V V, COHEN A T, EDMONSON R A, et al. Low molecular weight versus standard heparin for prevention of venous thromboembolism after major abdominal surgery. The Thromboprophylaxis Collaborative Group [J]. Lancet (London, England), 1993, 341(8840): 259-65.
[67] MICHOT M, CONEN D, HOLTZ D, et al. Prevention of deep-vein thrombosis in ambulatory arthroscopic knee surgery: a randomized trial of prophylaxis with low--molecular weight heparin [J]. Arthroscopy, 2002, 18(3): 257-63.
[68] SHORR A F, ERIKSSON B I, JAFFER A K, et al. Impact of stage 3B chronic kidney disease on thrombosis and bleeding outcomes after orthopedic surgery in patients treated with desirudin or enoxaparin: insights from a randomized trial [J]. Journal of thrombosis and haemostasis : JTH, 2012, 10(8): 1515-20.
[69] FITZGERALD R H, JR., SPIRO T E, TROWBRIDGE A A, et al. Prevention of venous thromboembolic disease following primary total knee arthroplasty. A randomized, multicenter, open-label, parallel-group comparison of enoxaparin and warfarin [J]. The Journal of bone and joint surgery American volume, 2001, 83(6): 900-6.
[70] FRANCIS C W, DAVIDSON B L, BERKOWITZ S D, et al. Ximelagatran versus warfarin for the prevention of venous thromboembolism after total knee arthroplasty - A randomized, double-blind trial [J]. Annals of Internal Medicine, 2002, 137(8): 648-55.
[71] RASKOB G E, GALLUS A S, PINEO G F, et al. Apixaban versus enoxaparin for thromboprophylaxis after joint replacement surgery: pooled analysis of major venous thromboembolism and bleeding in 8,464 patients from the ADVANCE 2 and 3 trials [J]. Blood, 2010, 116(21).
[72] VAN DER VEEN L, SEGERS M, VAN RAAY J J A M, et al. Bleeding complications of thromboprophylaxis with dabigatran, nadroparin or rivaroxaban for 6 weeks after total knee arthroplasty surgery: a randomised pilot study [J]. Bmj Open, 2021, 11(1).
[73] HAAS S, BREYER H G, BACHER H P, et al. Prevention of major venous thromboembolism following total hip or knee replacement: a randomized comparison of low-molecular-weight heparin with unfractionated heparin (ECHOS Trial) [J]. International angiology : a journal of the International Union of Angiology, 2006, 25(4): 335-42.

[74] WANG Z Y, WAN Y D, LIU X Z, et al. A Single-Center, Randomized, Double-Blind Study of 94 Patients Undergoing Surgery for Cerebral Glioma to Compare Postoperative Thromboprophylaxis with and without Rivaroxaban [J]. Medical science monitor : international medical journal of experimental and clinical research, 2022, 28: e934341.
[75] COLWELL C W, JR., COLLIS D K, PAULSON R, et al. Comparison of enoxaparin and warfarin for the prevention of venous thromboembolic disease after total hip arthroplasty. Evaluation during hospitalization and three months after discharge [J]. The Journal of bone and joint surgery American volume, 1999, 81(7): 932-40.
[76] PRANDONI P, BRUCHI O, SABBION P, et al. Prolonged thromboprophylaxis with oral anticoagulants after total hip arthroplasty: a prospective controlled randomized study [J]. Archives of internal medicine, 2002, 162(17): 1966-71.
[77] VAN ADRICHEM R A, NEMETH B, ALGRA A, et al. Thromboprophylaxis after Knee Arthroscopy and Lower-Leg Casting [J]. New England Journal of Medicine, 2017, 376(6): 515-25.
[78] COHEN A T, DAVIDSON B L, GALLUS A S, et al. Efficacy and safety of fondaparinux for the prevention of venous thromboembolism in older acute medical patients: randomised placebo controlled trial [J]. Bmj-British Medical Journal, 2006, 332(7537): 325-7.
[79] LASSEN M R, DAVIDSON B L, GALLUS A, et al. The efficacy and safety of apixaban, an oral, direct factor Xa inhibitor, as thromboprophylaxis in patients following total knee replacement [J]. Journal of thrombosis and haemostasis : JTH, 2007, 5(12): 2368-75.
[80] MAHé I, BERGMANN J F, D'AZéMAR P, et al. Lack of effect of a low-molecular-weight heparin (nadroparin) on mortality in bedridden medical in-patients: a prospective randomised double-blind study [J]. European journal of clinical pharmacology, 2005, 61(5-6): 347-51.
[81] ISHI S V, LAKSHMI M, KAKDE S T, et al. Randomised controlled trial for efficacy of unfractionated heparin (UFH) versus low molecular weight heparin (LMWH) in thrombo-prophylaxis [J]. The Journal of the Association of Physicians of India, 2013, 61(12): 882-6.
[82] VERHAMME P, GUNN S, SONESSON E, et al. Single-dose TB-402 or rivaroxaban for the prevention of venous thromboembolism after total hip replacement. A randomised, controlled trial [J]. Thrombosis and haemostasis, 2013, 109(6): 1091-8.
[83] RIESS H, HAAS S, TEBBE U, et al. A randomized, double-blind study of certoparin vs. unfractionated heparin to prevent venous thromboembolic events in acutely ill, non-surgical patients: CERTIFY Study [J]. Journal of thrombosis and haemostasis : JTH, 2010, 8(6): 1209-15.
[84] TURPIE A G G, FISHER W D, BAUER K A, et al. BAY 59-7939: an oral, direct Factor Xa inhibitor for the prevention of venous thromboembolism in patients after total knee replacement. A phase II dose-ranging study [J]. Journal of Thrombosis and Haemostasis, 2005, 3(11): 2479-86.
[85] TURPIE A G G, LENSING A W A, FUJI T, et al. Pharmacokinetic and clinical data supporting the use of fondaparinux 1.5 mg once daily in the prevention of venous thromboembolism in renally impaired patients [J]. Blood Coagulation & Fibrinolysis, 2009, 20(2): 114-21.
[86] MCLEOD R S, GEERTS W H, SNIDERMAN K W, et al. Subcutaneous heparin versus low-molecular-weight heparin as thromboprophylaxis in patients undergoing colorectal surgery: results of the canadian colorectal DVT prophylaxis trial: a randomized, double-blind trial [J]. Annals of surgery, 2001, 233(3): 438-44.
[87] ERIKSSON B I, BORRIS L C, FRIEDMAN R J, et al. Rivaroxaban versus enoxaparin for thromboprophylaxis after hip arthroplasty [J]. The New England journal of medicine, 2008, 358(26): 2765-75.
[88] LECLERC J R, GEERTS W H, DESJARDINS L, et al. Prevention of venous thromboembolism after knee arthroplasty. A randomized, double-blind trial comparing enoxaparin with warfarin [J]. Annals of internal medicine, 1996, 124(7): 619-26.
[89] ZHANG J, ATALLA M, MALLICK R, et al. Thromboprophylaxis for patients with newly diagnosed vs. recurrent cancers: a post-hoc analysis of the avert trial [J]. Journal of thrombosis and thrombolysis, 2021, 51(3): 720-4.
[90] DOBESH P P. Novel concepts: Emerging data and the role of extended prophylaxis following hip fracture surgery [J]. American Journal of Health-System Pharmacy, 2003, 60(22): S15-S9.
[91] HULL R D, PINEO G F, FRANCIS C, et al. Low-molecular-weight heparin prophylaxis using dalteparin in close proximity to surgery vs warfarin in hip arthroplasty patients: a double-blind, randomized comparison. The North American Fragmin Trial Investigators [J]. Archives of internal medicine, 2000, 160(14): 2199-207.
[92] COLWELL C W, JR., BERKOWITZ S D, DAVIDSON B L, et al. Comparison of ximelagatran, an oral direct thrombin inhibitor, with enoxaparin for the prevention of venous thromboembolism following total hip replacement. A randomized, double-blind study [J]. Journal of thrombosis and haemostasis : JTH, 2003, 1(10): 2119-30.
[93] HAAS S, SCHELLONG S M, TEBBE U, et al. Heparin based prophylaxis to prevent venous thromboembolic events and death in patients with cancer - a subgroup analysis of CERTIFY [J]. BMC cancer, 2011, 11: 316.
[94] GOLDHABER S Z, LEIZOROVICZ A, KAKKAR A K, et al. Apixaban versus Enoxaparin for Thromboprophylaxis in Medically Ill Patients [J]. New England Journal of Medicine, 2011, 365(23): 2167-77.
[95] COMP P C, VOEGELI T, MCCUTCHEN J W, et al. A comparison of danaparoid and warfarin for prophylaxis against deep vein thrombosis after total hip replacement: The Danaparoid Hip Arthroplasty Investigators Group [J]. Orthopedics, 1998, 21(10): 1123-8.
[96] HULL R D, SCHELLONG S M, TAPSON V F, et al. Extended-Duration Venous Thromboembolism Prophylaxis in Acutely III Medical Patients With Recently Reduced Mobility [J]. Annals of Internal Medicine, 2010, 153(1): 8-+.
[97] PLANES A, VOCHELLE N. The post-hospital discharge venous thrombosis risk of the orthopedic patient [J]. Orthopedics, 1997, 20 Suppl: 18-21.
[98] LASSEN M R, RASKOB G E, GALLUS A, et al. Apixaban versus enoxaparin for thromboprophylaxis after knee replacement (ADVANCE-2): a randomised double-blind trial [J]. Lancet, 2010, 375(9717): 807-15.
[99] BORSTAD E, URDAL K, HANDELAND G, et al. Comparison of low molecular weight heparin vs. unfractionated heparin in gynecological surgery. II: Reduced dose of low molecular weight heparin [J]. Acta obstetricia et gynecologica Scandinavica, 1992, 71(6): 471-5.
[100] SAMAMA C M, LECOULES N, KIERZEK G, et al. Comparison of Fondaparinux with Low-Molecular-Weight Heparin for Venous Thromboembolism Prevention in Patients Requiring Rigid or Semi-Rigid Immobilization for Isolated Non-Surgical Below-Knee Injury [J]. Annales francaises de medecine d'urgence, 2014, 4(3): 153-66.
[101] ERIKSSON B I, TURPIE A G G, LASSEN M R, et al. A dose escalation study of YM150, an oral direct factor Xa inhibitor, in the prevention of venous thromboembolism in elective primary hip replacement surgery [J]. Journal of Thrombosis and Haemostasis, 2007, 5(8): 1660-5.
[102] LASSEN M R, AGENO W, BORRIS L C, et al. Rivaroxaban versus enoxaparin for thromboprophylaxis after total knee arthroplasty [J]. New England Journal of Medicine, 2008, 358(26): 2776-86.
[103] PIAZZA G C, SPYROPOULOS A, HSIA J, et al. Rivaroxaban for Prevention of Thrombotic Events, Hospitalization, and Death in Outpatients With COVID-19: A Randomized Clinical Trial [J]. Circulation, 2023, 147(25): 1891-901.
[104] BUELLER H R, BETHUNE C, BHANOT S, et al. Factor XI Antisense Oligonucleotide for Prevention of Venous Thrombosis [J]. New England Journal of Medicine, 2015, 372(3): 232-40.
[105] BERGQVIST D, AGNELLI G, COHEN A T, et al. Duration of prophylaxis against venous thromboembolism with enoxaparin after surgery for cancer [J]. The New England journal of medicine, 2002, 346(13): 975-80.
[106] LONG A, ZHANG L, ZHANG Y, et al. Efficacy and safety of rivaroxaban versus low-molecular-weight heparin therapy in patients with lower limb fractures [J]. Journal of Thrombosis and Thrombolysis, 2014, 38(3): 299-305.
[107] WEITZ J I, BAUERSACHS R, BECKER B, et al. Effect of Osocimab in Preventing Venous Thromboembolism Among Patients Undergoing Knee Arthroplasty: The FOXTROT Randomized Clinical Trial [J]. Jama, 2020, 323(2): 130-9.
[108] ANDERSON D R, DUNBAR M, MURNAGHAN J, et al. Aspirin or Rivaroxaban for VTE Prophylaxis after Hip or Knee Arthroplasty [J]. The New England journal of medicine, 2018, 378(8): 699-707.
[109] FUJI T, FUJITA S, KAWAI Y, et al. Safety and efficacy of edoxaban in patients undergoing hip fracture surgery [J]. Thromb Res, 2014, 133(6): 1016-22.
[110] GEERTS W H, JAY R M, CODE K I, et al. A comparison of low-dose heparin with low-molecular-weight heparin as prophylaxis against venous thromboembolism after major trauma [J]. The New England journal of medicine, 1996, 335(10): 701-7.
[111] ERIKSSON B I, KAKKAR A K, TURPIE A G G, et al. Oral rivaroxaban for the prevention of symptomatic venous thromboembolism after elective hip and knee replacement [J]. Journal of Bone and Joint Surgery-British Volume, 2009, 91B(5): 636-44.
[112] ABBAS M S. Bemiparin versus enoxaparin in the prevention of venous thromboembolism among intensive care unit patients [J]. Indian journal of critical care medicine, 2017, 21(7): 419-23.
[113] PAI M, ADHIKARI N K J, OSTERMANN M, et al. Low-molecular-weight heparin venous thromboprophylaxis in critically ill patients with renal dysfunction: A subgroup analysis of the PROTECT trial [J]. Plos One, 2018, 13(6).
[114] TURPIE A G, GALLUS A S, HOEK J A. A synthetic pentasaccharide for the prevention of deep-vein thrombosis after total hip replacement [J]. The New England journal of medicine, 2001, 344(9): 619-25.
[115] TURPIE A G, BAUER K A, DAVIDSON B L, et al. A randomized evaluation of betrixaban, an oral factor Xa inhibitor, for prevention of thromboembolic events after total knee replacement (EXPERT) [J]. Thrombosis and haemostasis, 2009, 101(1): 68-76.
[116] LEVINE M N, HIRSH J, GENT M, et al. Prevention of deep vein thrombosis after elective hip surgery. A randomized trial comparing low molecular weight heparin with standard unfractionated heparin [J]. Annals of internal medicine, 1991, 114(7): 545-51.
[117] BRANDT W, BROWN C, WANG T-F, et al. Efficacy and safety of apixaban for primary prevention of thromboembolism in patients with cancer and a central venous catheter: A subgroup analysis of the AVERT Trial [J]. Thrombosis Research, 2022, 216: 8-10.
[118] YI X, LIN J, WANG C, et al. Low-molecular-weight heparin is more effective than aspirin in preventing early neurologic deterioration and improving six-month outcome [J]. Journal of stroke and cerebrovascular diseases : the official journal of National Stroke Association, 2014, 23(6): 1537-44.
[119] AGNELLI G, BERGQVIST D, COHEN A T, et al. Randomized clinical trial of postoperative fondaparinux versus perioperative dalteparin for prevention of venous thromboembolism in high-risk abdominal surgery [J]. The British journal of surgery, 2005, 92(10): 1212-20.

[120] ERIKSSON B I, BORRIS L C, DAHL O E, et al. A once-daily, oral, direct Factor Xa inhibitor, rivaroxaban (BAY 59-7939), for thromboprophylaxis after total hip replacement [J]. Circulation, 2006, 114(22): 2374-81.
[121] CONNORS J M, BROOKS M M, SCIURBA F C, et al. Effect of Antithrombotic Therapy on Clinical Outcomes in Outpatients With Clinically Stable Symptomatic COVID-19 The ACTIV-4B Randomized Clinical Trial [J]. Jama-Journal of the American Medical Association, 2021, 326(17): 1703-12.
[122] PLANES A, VOCHELLE N, FAGOLA M, et al. Comparison of two low-molecular-weight heparins for the prevention of postoperative venous thromboembolism after elective hip surgery. Reviparin Study Group [J]. Blood coagulation & fibrinolysis : an international journal in haemostasis and thrombosis, 1998, 9(6): 499-505.
[123] PALUMBO A, CAVO M, BRINGHEN S, et al. Aspirin, warfarin, or enoxaparin thromboprophylaxis in patients with multiple myeloma treated with thalidomide: a phase III, open-label, randomized trial [J]. Journal of clinical oncology : official journal of the American Society of Clinical Oncology, 2011, 29(8): 986-93.
[124] CAMPORESE G, BERNARDI E, NOVENTA F, et al. Efficacy of Rivaroxaban for thromboprophylaxis after Knee Arthroscopy (ERIKA). A phase II, multicentre, double-blind, placebo-controlled randomised study [J]. Thrombosis and haemostasis, 2016, 116(2): 349-55.
[125] DE A, ROY P, GARG V K, et al. Low-molecular-weight heparin and unfractionated heparin in prophylaxis against deep vein thrombosis in critically ill patients undergoing major surgery [J]. Blood Coagulation & Fibrinolysis, 2010, 21(1): 57-61.
